# Supplementary material for: Primary health care during the COVID-19 pandemic: A qualitative exploration of the challenges and changes in practice experienced by GPs and GP trainees
Source: PLoS One. 2023 Feb 9;18(2):e0280733. doi: 10.1371/journal.pone.0280733 (PMC9910752; doi:10.1371/journal.pone.0280733)
Supplement: S1 Appendix — (PDF) [file pone.0280733.s001.pdf]

Topic Guide

With the aim of establishing a good rapport (Reinharz, 1993) (45), *and a funnel towards the questions with more potential to be distressing* (Kyale & Brinkmann, 2009) (45).

Introduction

- Introduce self, nature and outline of research
- Gain informed verbal consent – confirm understanding of the topic guide
- We expect this to take 45 minutes, but how long works for you etc?

Interview

INTRODUCTION QUESTIONS

1. Could you tell me a little about your experience in GP care?
    a. Please can you describe your **practice population** and **role in the practice?**

EXPERIENCE OF THE PANDEMIC PROFESSIONALLY

2. Can you tell me about your professional experience of the COVID-19 pandemic?
    a. How **prepared** did you feel for the pandemic as a GP?
        i. How informed did you feel about the risk of COVID-19, and how to reduce your own risk?
        ii. Tell me about the PPE (ie. EPI) that was available…
        iii. What support was offered to you? (*Counselling and peer support groups)*
    b. How **well informed** did you feel your patients were about the pandemic? Tell me about this…
        i. How understanding were they of the new safety procedures? *less visitors, mask wearing*
    c. How did you feel **making decisions** with the guidance you had? *explore morality problems*
        i. How did you feel in the role of a decision maker?

3. In what ways did common practice change for you?
    a. Tell me about any changes to clinical guidelines…have you found any of these effective in adjusting to COVID-19?
    b. In your experience, how have GPs been utilised, and have they had to take on any new roles? (*eg. vaccination*)
    c. What change, if any, has it had on interactions between GP staff/ between GP and hospital staff?
    d. If not mentioned: *telemedicine, hot hubs, emotional debriefs*
    e. Has it changed your relationships with your patients? *Ability to ask unwell patients to leave, wear PPE correctly, etc.*

GOVERNMENT

4. What is your opinion of the government response to COVID-19?
    a. How effective have they been in controlling the pandemic?
    b. What is your opinion of the public health messages/policies?

EXPERIENCE OF THE PANDEMIC PERSONALLY

5. *Sign-post – more sensitive question* – has COVID-19 had any impact for you personally?
    a. Are you in an at-risk group for COVID? Is any of your family? What effect has this had for you as a general practitioner?
    b. What protective measures have you taken for yourself? *How do you look after your physical and mental health, if you have the time?*
    c. If COVID-19 has had any mental or physical impacts for you, do you feel this has influenced your practice, and how?

FUTURE OF GENERAL PRACTICE

6. Are there any changes which you think should be carried on in the future? Why and how?
    a. Are there any that should *not* be?
    b. Can you describe the future of general practice in recovering from this pandemic?
    c. What do you think we can learn from the pandemic thus far, to inform future GP care?

QUESTIONS SPECIFIC TO GP TRAINEE

7. How do you think the pandemic has influenced your training?
    a. Has it had any effect on your views of general practice?
    b. Has it informed your specialty choice?

Summative Questions

8. Is there anything else you would like to tell me about working as a GP during the pandemic?

Thank participant and ask if they have any questions. Summarise back to them main findings from interview to confirm most salient topics have been covered.

Notes for interviewer:

Numbers indicate questions. Letters indicate prompts. Italics are notes for interviewer.
