# Supplementary material for: Primary health care during the COVID-19 pandemic: A qualitative exploration of the challenges and changes in practice experienced by GPs and GP trainees
Source: PLoS One. 2023 Feb 9;18(2):e0280733. doi: 10.1371/journal.pone.0280733 (PMC9910752; doi:10.1371/journal.pone.0280733)
Supplement: S3 Appendix — (PDF) [file pone.0280733.s003.pdf]

**Supplementary Material 3**

Unpublished / unavailable work referenced

a) Russell A, de Wildt G, Grut M, Greenfield S, Clarke J. What can general practice learn from primary care nurses' and healthcare assistants' experiences of the COVID-19 pandemic? Challenges, opportunities, and lessons for resilience. A qualitative study. [unpublished study]. 2021.

b) Dillon R, Kearney G, Donnelly M, Stockman N. Pursuing flourishing general practice: Understanding the pressures affecting practices at the 'coal face'. [unpublished study]. 2021.

c) Burn E, Locock L, Smith J, Fisher B. Narrative accounts of primary care practitioners in a time of COVID-19. [unpublished study]. 2021
