# Supplementary material for: Primary health care during the COVID-19 pandemic: A qualitative exploration of the challenges and changes in practice experienced by GPs and GP trainees
Source: PLoS One. 2023 Feb 9;18(2):e0280733. doi: 10.1371/journal.pone.0280733 (PMC9910752; doi:10.1371/journal.pone.0280733)
Supplement: S4 Appendix — (PDF) [file pone.0280733.s004.pdf]

GP AND GP TRAINEE VOLUNTEERS NEEDED FOR INTERVIEWS

STUDY: Primary Health Care during the COVID-19 pandemic: a qualitative exploration of the challenges and changes in practice experienced by GPs and GP trainees

We are looking for **GP and GP trainees** to take part in a study between February and March.

We would like to interview you about your perceptions and experiences of the pandemic in General Practice.

Our aim is to gain an understanding of the perceptions and experiences of Primary Health Care workers during the COVID-19 pandemic. We hope that through this, we can understand the impact it has had on GPs, if it has affected their clinical practice, and how we can learn from this in future.

If you are a GP or GP trainee that worked before *and* during the pandemic in the UK, we would be very grateful for your participation. You will be required to take part in a 45-minute interview carried out by video call or phone call, with the lead researcher.

We would like to offer a **£30 AMAZON VOUCHER** as thanks for your participation.

If you are interested, or would like further details, please contact the researcher by phone or email.

Contact Details:

Lead Researcher: Minka Grut, University of Birmingham

Email: mkg757@student.bham.ac.uk

Number: 07587140920

UNIVERSITY OF BIRMINGHAM

Thank you for your interest in participating in our study.
