# Supplementary material for: Primary health care during the COVID-19 pandemic: A qualitative exploration of the challenges and changes in practice experienced by GPs and GP trainees
Source: PLoS One. 2023 Feb 9;18(2):e0280733. doi: 10.1371/journal.pone.0280733 (PMC9910752; doi:10.1371/journal.pone.0280733)
Supplement: S1 Data — (ZIP) [file pone.0280733.s005.zip › GP1 Transcript.pdf]

GP1 Transcript

Interviewer: So to start, could you tell me a little bit about your experience in GP care?

GP1: So, I've been a GP principle for, um, well over- well- 15, 17 years, I am a trainer, I'm an appraiser and I train medical students, foundation doctors, and registrars.

Interviewer: OK.

GP1: I have an interest in diabetes, I lead on the vaccination program on our site, which is the one that provides- It's a PCN site that we have.

Interviewer: OK.

GP1: Um, it's an inner-city practice… uh, we cater to a very low deprivation index.

Interviewer: OK.

GP1: Anything else you need to know?

Interviewer: Um, what's your practice population? Is it- is there many patients?

GP1: 5,000 patients?

Interviewer: 5,000, OK, thank you. Could you tell me about your experiences of the COVID-19 pandemic, as a GP?

GP1: Personally, or professionally?

Interviewer: Professionally.

GP1: Um… So… It has been a challenge. It has been a challenge both positive and negative. So, we have learnt to adapt… adapt and sustain. Um, it's been a year where resilience has been created and scaled up in so many ways. We have moved to accepting digitalisation…

Interviewer: OK?

GP1: …looked at alternate methods of consultations. We have developed a lot of in-house, um, team… dependence… taking care of one another, being kind to one another, making sure that person-help staff is paramount

Interviewer: Hmmm.

GP1: Um, we have, um, challenges in terms of maintaining mainstream general practice in addition to vaccination more recently.

Interviewer: OK, what do you mean by that?

GP1: So, I hold the role of a lead GP principle and I hold the role of the lead vaccinator. And… with the vaccination ramp-up we've needed to deliver up to about 8,000-odd vaccines in 5 weeks, which means mainstream work – there's no time or capacity for them.

Interviewer: Right, OK. And how has that been for you, having such a role of responsibility in the vaccination program?

GP1: Personally, it has been um… a very good challenge.

Interviewer: OK.

GP1: Um, I feel quite… quite a buzz, quite grateful that I'm able to deliver this at the time of a pandemic, it's like I signed up for my- something useful for my life!

Interviewer: Sure!

Both laugh

Interviewer: Um, it's a huge feat, 8,000 vaccinations.

GP1: Do I see…?

Interviewer: Sorry – I said it's a huge achievement, 8,000 vaccinations.

GP1: Absolutely, we're a very small practice as you can see.

Interviewer: Yeah, no, it's really exciting. Um-

GP1: -Yes, it's a great teamwork.

Interviewer: How prepared did you feel for the pandemic as a GP? And that could be in terms of… information from the government, uh, PPE supplies, uh, counsel support, uh, anything really.

GP1: Um… well, I think it would be unfair for me to say… um, I think we did too little too late generally.

Interviewer: Right.

GP1: From a political perspective.

Interviewer: Sure.

GP1: Um… But… From a vaccine perspective, I think the program has been pretty impressive. Um, I think that general practice has responded astonishingly well to the challenge, um… And I think… we… for PPE I think it was… initially very little… and … there has never been a huge infrastructure for PPE.

Interviewer: Sure.

GP1: Um, and some protection although theoretically was paramount, I don't think we were always protected. Before this now, and learning more about the illusive nature of the virus, um… I think it's become more… at the end of the year we'll have kind of learnt how to adapt a bit more… but PPE is a let-down.

Interviewer: Yeah. Did you receive PPE at all at the beginning, or was this a…?

GP1: Yeah, we had some. Um… but it was more because we sourced a lot of it ourselves.

Interviewer: Right.

GP1: We also… Uh, information was… I think there was- there was a bombardment of information, there was an overload initially, because A) we needed it, it was a new pandemic, unprecedented life

Interviewer: Sure

GP1: Um… I think… We've got… We had… varying standards. For example, the Royal College had a covid section to it, there was huge adaptations to Covid-related needs, uh, in all forms of information, everywhere, and I think that I was quite satisfied with that.

Interviewer: So you spoke about how as a team, it was strengthening, um, in terms of supporting each other. Do you feel that you had peer support or emotional support during the pandemic?

GP1: Personally? Um… yes, but I think, um… it's more to do with the fact that in our status as carers, um, we are a lot more resilient, I don't think, um… there's been a huge amount of support. As a- when you're a GP principle, and you're leading the program -

Interviewer: Uh-huh

GP1: -of general practice, (*unintelligible*), you need to be taking care of others and there was rarely a good time that they would pause and ask if I was OK.

Interviewer: Right, OK, um, thank you very much. As you were in a role of responsibility – I touched on this earlier – how did you feel making decisions with the guidance you had been given by um… the Royal College of GPs or whoever you were receiving your guidance from.

GP1: Um… There's never going to be a perfect evidence-based answer, there's are lots and lots of variables in this -

Interviewer: - Sure

GP1: But I think… um… the tool kits that have been thrown our way is something to work with and I'm quite happy to carry on with what I have… um… fully well accommodating for the fact that we would not be in a position to ask the Royal questions.

Interviewer: Right, OK. So I'm going to ask more about in general practice, um, in what ways has contact changed for you? You've spoken a bit about telemedicine and new formats for consultations, could you tell me more about that?

GP1: Uh, in terms of telemedicine coming into practice?

Interviewer: Sure, any changes that you've experienced really in practice.

GP1: Yeah, so we have um… here, triage first like the rest of the world in terms of general practice delivery. So… it's always telephone triage first, and then we've moved on to… I think about 25-30% video consultations.

Interviewer: OK.

GP1: And… quite comfortable. We have (*unintelligible*) a tool called AccuRx, which merges with our IT system in terms of… um… giving patients leaflets… um… texting patients, getting photographs from them, sending sicknotes. Uh- an incredible tool that has come into the practice, from an IT perspective.

Interviewer: Had you, um, had you used that before the pandemic? The AccuRx?

GP1: Nope, never.

Interviewer: Oh, OK – how was that adjusting to a new, um, programme?

GP1: Very exciting.

Interviewer: Really? OK! That's great.

GP1: Really positive.

Interviewer: And do you think you would continue using it?

GP1: Yes

Interviewer: OK, um so you've said you had, was it 25-30% were video consultations. Are the rest in-person consultations?

GP1: No, we do very little face-to-face, we do majority as telephones, a few videos, and I think, again, that's… uh- I don't see myself going back to my old times, I would only bring in relevant patients.

Interviewer: OK. So could you tell me about any changes in the workforce, for example have you noticed a change in the number of hours that you have to work, or in the staff that you've interacted with?

GP1: It's more working from home, really, then um… then the other way around.

Interviewer: OK. Has that been the majority of your work now, is that based at home?

GP1: Very possible, but it's difficult to sustain that kind of work in general practice. Um, front desk for example cannot log onto (*unintelligible)*. Um, some clinical options have been given, particularly in sickness, um… but… um… it is an option that's opened up. Um, we are not able to move all of it into remote working, but… managers… some of the clinical staff, we have tried to move there.

Interviewer: OK, did you say… I think you have mentioned patient notes, but I didn't hear what you said, are you able to access patient notes from home?

GP1: Yes, yes.

Interviewer: OK, and that transition's been fine?

GP1: Yes.

Interviewer: How have you found that GPs have been utilised? So I know you spoke about vaccinations, are there any other new roles you've taken on?

GP1: No.

Interviewer: No? Or have there been any differences in your interactions with other GP staff or hospital staff?

GP1: Pardon?

Interviewer: Have you had any new interactions with hospital staff, or other GPs?

GP1: No.

Interviewer: OK. Uh… I've read a bit about a concept called hot hubs. Has that been something, um, in your practice?

GP1: No.

Interviewer: No, OK. Could you… um… I'm that sure your patients have been speaking to NHS 111. Have you experienced that at all?

GP1: NHS 111 *(unintelligible)*.

Interviewer: Sure, because they have might have taken on some new roles that might not have been for them previously?

GP1: Yes, they have taken on a lot of COVID-related queries and, um, diagnosing… which has been very useful. But… then actually um… sending off, um… patients for us to be seen after, and that has been rather cumbersome, because I'd rather they didn't keep using up our slots for it?

Interviewer: OK, how does that work normally, in terms of the slots?

GP1: So uh, we have given them, um, for every x number of patients they're allowed to… directly book these slots in for us.

Interviewer: OK

GP1: Which means that we lose an appointment to them for face-to-face, sometimes they might- they may have been managed by video or phone call.

Interviewer: Right, OK. Would you say the pandemic has changed your relationship with your patients?

GP1: Yes. I think so, but I'm not able to quantify, or take it as a positive or a negative one. Um… at the moment, the… um… the press and the negativity that comes up from the fact that GPs are not opening up for patients has been very demoralising and unnecessary, but from a patient perspective, I think soon we'll see their patience is beginning to wear out, which we are beginning to see quite a bit anyway.

Interviewer: What is their impatience towards?

GP1: I think this acute work that has been delayed and postponed in terms of referrals to secondary care?

Interviewer: Sure

GP1: So that has been delayed significantly.

Interviewer: How have you found that it has affected your care of chronic management, has that changed at all?

GP1: Um… a lot of it has become virtual, in terms of, um… of sharing… um… lots of it has to be adapted – diabetic care, COPD care… has been affected.

Interviewer: When you say adapted?

GP1: Um… so chronic reviews and follow ups have been done virtually on the phone.

Interviewer: Okay. So, my next questions is about your opinion of the government response to COVID-19, in how effective they have been in controlling the pandemic?

GP1: In terms of lockdown?

Interviewer: Sure, in terms of controlling the spread of the pandemic and informing people like your patients.

GP1: Um… it's hugely politically driven in terms of giving information about the pandemic and the vaccination programme. I would say that we've had a fair amount of support with the vaccination programme compared to the actual disease process?

Interviewer: Sorry, did you say the disease process? What's that, sorry?

GP1: The amount of information given for the disease process? And for the vaccination programme. I was talking about actual conditions and the pandemic…um, initially it was very chaotic, but then the vaccination programme- I think that it's been a bit more structured.

Interviewer: OK, have you had any experience with the Track and Trace system, or the Testing programmes?

GP1: Um, it was, as all of us would agree, it was a major disaster. Um, I think it's one of the worst, you know, it's (*unintelligible)*.

Interviewer: Why do you say it was a disaster?

GP1: It didn't work, did it (*laughs)*. No, it didn't work in any way

Interviewer: Um, did you find GPs were utilised well in that scenario?

GP1: Did I have anybody use it?

Interviewer: Did you find GPs had a role in the Track and Trace system at all?

GP1: Nope, no.

Interviewer: OK. Do you have any thoughts on that?

(*Both laugh*)

Interviewer: Ok, so, this is a more personal question, I was wondering what your experience of the pandemic was personally. For example, you know, if you were in an at-risk group, or something like this.

GP1: Yeah, I'm BAME. I'm at-risk. But… I… I, uh you know, if you ask me what my personal experience was, I haven't been personally affected by COVID. But I live with someone who had a positive test and was positive for antibodies.

Interviewer: Oh, okay, how did that go for you as a GP? Were you still able to work?

GP1: Uh, I worked from home?

Interviewer: Alright, thank you. Have you had to take any protective measures for yourself in the pandemic?

GP1: I've been vaccinated.

Interviewer: Oh, OK, that's good! Good news. From what we've spoken about today, are there any changes which you think could be carried on in the future?

GP1: Well, adapting to technology and digitalisation is the way forward in many ways. Um… but… looking at… it is a huge amount of political drive into this, and it becomes harder to make some rational clinical decisions. So there are umpteen number of examples I can give. The last one is, I'm not sure if you're aware, we have gestational diabetes now added to cohort number 4- which, you know, has very little evidence behind it and we've got such mixed messages. Um… public have been told something, and we have been informed later. Similarly, opening our vaccinations at **REDACTED location**, when to the same cohorts that we are doing as at GPs, there's quite a few mixed messages from the government. Um, I suppose it is a pandemic, but it would be useful to sing out of one hymn sheet.

Interviewer: Uh-huh. I see what you mean. In um, in terms of cohort 4, I'm not familiar with that system, is that how they organise who's vaccinated?

GP1: So the government have got their own methods of creating who goes to the vulnerable risk groups, and some of them don't tally with what we think is the clinically derived risk groups.

Interviewer: Right, I see. How's your experience of that been with patients who are – I assume you have patients who are trying to get vaccinations….

GP1: Yeah, correct. So it is that we've been giving mixed messages to public and patients, so we need to honour what they have been told, because it is safety at the end of the day.

Interviewer: It must be difficult for you navigating, um, the conversations with patients, around who gets a vaccination when.

GP1: Yes, and also justifying reasons for why something is against a guideline that's been provided to them, so communication, doctor-patient relationship, breakdown of certain barriers, all these things happen.

Interviewer: Has it bought you closer to your patients in any way?

GP1: Hmmm… no I don't think so! (*laughs*)

Interviewer: Ok, that's fair enough, it's worth asking! So, a more broad question – can you describe the future of general practice, in recovering from this pandemic, how do you see general practice, um, sort of coming out of the pandemic?

GP1: Um… it's going to take time, it's going to be unsupported in many ways, and we're going to be left taking the crumbs out of normal day-to-day work, including acute and chronic care, um… and we're hoping that we wouldn't… we would have a good infrastructure towards this.

Interviewer: OK, would you have any advice to people making policies, if you were to use your own experience to inform?

GP1: Having clinical representation in the policy making.

Interviewer: Oh sure, is there much representation at the moment, for GPs?

GP1: I don't know.

Interviewer: Ok, well that's good to know. Um… and then… I've asked most of the main- the salient questions I have, so, what do you think we could learn from the pandemic, so far?

GP1: What do you think we have learnt?

Interviewer: Yeah. Or yeah, can learn.

GP1: Um… the value of life, I suppose

Interviewer: That’s a very nice answer!

(*Both laugh*)

Interviewer: Um, is there anything else you would like to tell me about working as a GP during the pandemic that I haven’t touched on? Anything important to you or practice in general?

GP1: It has been satisfying.

Interviewer: OK.

GP1: Despite having had the challenge thrown to us, I think it’s been… um, it’s been… you go back home with a buzz.

Interviewer: That’s really nice to hear. It sounds like… um, yeah – it sounds like you particularly have had quite a big challenge with the vaccination programme and your role as a teacher, but it’s good to hear that it’s been, you know, a manageable experience. From this interview, I feel that the main topics we’ve covered are regarding telemedicine, um, the switch to it being mainly effective, and then potentially the PPE being less effective- in terms of it being supplied to you, um, and you mentioned that it did strengthen your working environment with other GPs. Is that correct?

GP1: Hmmm.

Recording ends.
