# Supplementary material for: Primary health care during the COVID-19 pandemic: A qualitative exploration of the challenges and changes in practice experienced by GPs and GP trainees
Source: PLoS One. 2023 Feb 9;18(2):e0280733. doi: 10.1371/journal.pone.0280733 (PMC9910752; doi:10.1371/journal.pone.0280733)
Supplement: S1 Data — (ZIP) [file pone.0280733.s005.zip › GP2 Transcript.pdf]

GP2 Transcript

Interviewer: So, firstly, could you tell me a little about your experience in GP care?

GP2: Yeah, certainly, so do you mean sort of during the pandemic or before?

Interviewer: Um, more generally, your training and so forth.

GP2: Yeah, so um… I've- I've worked in *REDACTED location* since I finished my training so I-I went to *REDACTED university*, in fact, as well, um… trained in *REDACTED location*, so foundation training was pretty much around *REDACTED regions* and then subsequently, um… I've been locuming for about… just under four years now. Um, and I, I just constantly move around, so I will go to regular uh, reg-, uh, practices regularly. Um… but generally it can be wherever the work takes me.

Interviewer: OK

GP2: Yep… experience overall, it's-it's been, uh, good overall, uh, it's, obviously it's challenging as I'm sure you've- you've heard, um… and during the pandemic obviously there's been increased challenges with that, obviously I'm sure we'll come to that, but, uh, yeah, on the whole, uh… challenging but enjoyable, I would say.

Interviewer: Alright, thank you. Um… well, I mean, I would ask then- what has been your professional experience of the pandemic?

GP2: Yeah, so I think the key things have been obviously the um… the change, in, uh, in the way we carry out the consultations with patients. Um… so one of the key things obviously is that um, we're not able to routinely perform a… examination, uh… so that obviously takes away a central part, if you like, of the consultation, for… for any doctor, and it-it makes us obviously rely more heavily than ever before on-one the history element, um… but obviously there's- there's ways and means to… to try, and um, you know, get around that. So, you know, using, uh, video, or, uh, photos where- where appropriate, um… So there's been an increased reliance on that to aid- uh, diagnoses and management. So I'd say that's the main, fundamental difference, in how the… in how… at least GP anyway, we've

been carrying out a consultation. Um… but that initially was, was quite difficult. So, um, early on in the pandemic, obviously, there might not have been provision for carrying out a video call in some practices, now I would say there is, pretty much everywhere? Um… so it was, it was tricky, that was just some of the challenges, early on, you know, sort of March, April last year.

Interviewer: Are you finding now that it's balanced between video and phone calls, or in-face consultations?

GP2: Yeah, now, um…. I'd say, because the public generally have got used to the mode of consultation as well, most patients will have the means, elderly included, of… uh… probably not video, but of getting a photograph to us, if that is indicated. So generally speaking, it's… very rarely it's… I personally will face a situation where it's not feasible at all.

Interviewer: That's good to hear, so it stays accessible for patients.

GP2: Yeah.

Interviewer: How do they, um, how do they send things like photos to you?

GP2: So generally, there's um… two means that are used in primary care, so one is just straight-forward email, so that will be the patient emailing it to the practice, uh, email address? Or there's another software called uh, AccuRx? I'm not sure if you've heard of that. Uh… that's very useful, to be honest, and I can see that sticking around for many years to come. Um… so that allows both video uh… as well as a patient to send photos through… uh… and you can even text them as well. So, uh…

Interviewer: Oh great. Had you had any experience with that before the pandemic started?

GP2: No, I hadn't, so it was completely new. Um, prior to the pandemic, in terms of telephone triaging, the only exposure I had to it was, uh, as a GP trainee, so um, as things stand at the moment, or they did stand when I was training, is you had to do a certain amount of hours, in out-of-hours, during your, uh, final year as a GP trainee, and

as part of that, uh, you did telephone triage. So some- some sessions were entirely out of hours telephone triage. Uh, but that- you know- as a proportion of your overall training that was very, very small, so you had very limited exposure to that. Uh… probably did the odd one in my regular ST3 practise as well. So, um… so yeah, pre-pre-pandemic, no- hardly any exposure to it and the software I- I had personally never used as I imagine most GPs hadn't really.

Interviewer: Yeah, you're one of my early interviews, but um, so far, very similar experience. It seems to have been a very escalated learning process.

GP2: Exactly, yeah, but fortunately, um…uh- it's something very easy to- to learn, especially with me, you know, being young, with- with technology, can get your head around it very quicky.

Interviewer: Yeah, you've got the advantage of understanding.

GP2: Yeah, whereas for older GPs, or sort ones nearing retirement, it's, I think it can be tricky getting a hold of the technology aspects of it.

Interviewer: Sure. Um… so you have had the shift to telemedicine then. How informed when the pandemic started did you feel about the risk to yourself personally, and the risk to your patients?

GP2: Yeah, so… at the start of the pandemic, sort of March last year, late March, early April, I think it, generally it was quite a frightening time for everyone, both doctors and patients included, so, uh, one of the issues early on was, there were no clear guidelines or instruction on how to go about, um, arranging to see patients? And that varied quite heavily from practice to practice. Uh, so being a locum and moving around, I had the luxury, of, kind of, seeing how different practices were dealing with it, early on. Now I would say it's fairly consistent across the board, so you triage patients, so if you, as the triaging doctor, feel that the patient needs to be seen, then you just book them into your clinic at that surgery, um, provided they don't have any Covid symptoms obviously. Whereas early on, I felt there was generally a… a pressure to perhaps not see any patients at all, but obviously, if uh- if a case arose where

you did need to see one, then you um, you sort of had to make the provisions either to get them booked in, or, um… there were these red sites that were being set up early on as well.

Interviewer: Red sites?

GP2: Three or four different practices in the area would nominate a single practice, where any patients that needed face-to-face appointments were being sent to.

Interviewer: Is this similar to the hot hub concept, or is that more Covid-centric?

GP2: Yeah similar, so I- I think that- the way that stands now is it's more for suspected Covid, so ones that obviously don't warrant, uh, admission? Whereas early on, it was for, um, for any patient that needed face-to-face consult. Um, but that-that varied from practice to practice, equally there were some early on where it was a- you were allowed to see patients in that surgery as well, if you needed to. Um… but it just varied. So that was one of the challenges I found early on, was that, they weren't, it wasn't that, sort of, clear instructional guideline across the board.

Interviewer: Yeah. How did you find navigating the variation between the practices, because it sounds like you were seeing quite a lot of different, um, circumstances? Which is really interesting because I've been speaking to somebody who has seen one practice, so it's great to hear a comparative, um, opinion.

GP2: Yeah, so you mean just, sort of, establishing what the protocol was in each practice?

Interviewer: Yeah, in each case, and sort of-

GP2: Yeah, it- I just check with the practice manager what- what was being done by the regular GPs on the ground by the partners and the salaried GPs, but, shortly after the pandemic, uh, started, I was fortunate to do a long-term locum at a single practice, so that obviously- which- which I would say was quite opportunistic, and…. beneficial to me, because it allowed me to get used to the norms of the practice, um…whereas uh, when things were a bit

uncertain early on, it was difficult constantly moving around, and knowing OK what's this practice doing, what's that one- whereas normal, that obviously wouldn't be an issue.

Interviewer: Yeah, no, it must've been, um, very- a slightly confusing time going from place to place!

GP2: Yeah definitely.

Interviewer: Were you generally working in the practice when you were there, or did you do any at-home work?

GP2: So it's all been in-practice.

Interviewer: OK. Is that because of your role as a locum?

GP2: Exactly, so it's, it's- so some of the partners in certain practices do- do remote working? Um… I've personally not done it, I think you need, there's just some technical issues around that. So you need, um the appropriate software, um… installed at home, and I think the CCG can provide you with a laptop as well, if needed.

Interviewer: Oh, OK.

GP2: But- but yeah, I've personally not done that though.

Interviewer: OK, fair enough. Did you find that in your practices you were protected, in terms of um… PPE, so like physical protection, and sort of social support? Was there anything offered to you to navigate the pandemic?

GP2: *(laughs)* Yeah, so I think Matt Hancock's been uh – you know… criticised recently hasn't he about this in the press about PPE, early on, um, I think there was a shortage, as we know, nationally of uh PPE so, um… I'm just trying to think the practices I was working at when it started. They… I think from my personal experience, there were always the, kind of, basic, you know, level 1 facemask available. Gloves are always available anyway in any- any- any

surgery, as well as the basic plastic aprons, so, those were always available, if needed. I can't ever recall a situation where I was about to see a person- a patient face to face and that wasn't available to me.

Interviewer: Right, OK. So, was that appropriate PPE?

GP2: Well- for a suspected Covid patient that wouldn't be sufficient, but obviously I was seeing the ones were they had been appropriately triaged, and didn't have any Covid symptoms, so appropriate for the patients in question.

Interviewer: How did the um, triaging process actually work, for the practices you saw?

GP2: Uh, yeah, so um… in terms of variation, so um, the main variation has been… so most practices just simply book the patient into your list, so reception book them in if they request an appointment. You can request one either by ringing up or emailing surgeries as well. And then you just call the patient back, obviously take a history and decide if, uh, you can resolve the issue over the phone, close the call, or whether you need to see them in person, or whether you need to send them to A&E.

Interviewer: Yeah.

GP2: There are some practices that are using other means, uh, what's it called again? It just escapes my mind, briefly. But the patient basically goes on the practice website, they can fill out a, um, a form themselves, which just outlines why they need to speak to a doctor, what the issue is, what their expected, um, uh- hope is ectara, just got through all of that, and that's essentially put- put into the patients, um, notes, which the GP will read, before calling the patient? So it gives the doctor, you know, a much better idea- insight into why they're calling them. And I think if the issue is, you know, is fairly basic, like a medication request, or they just want some advice, some GPs actually just reply by texting them back, using AccuRx, if they feel a call isn't necessary. But uh, so yeah, I've worked at one or two practices where they have used that, um… I think it has its pros and cons, it's slightly more time-consuming because for each patient you have to firstly read that through, and you end up calling them anyway, and in most cases the patient just repeats themselves, and you end up going- taking the- the history anyway, it's just like you're a bit more well informed…

Interviewer: You know what's coming a little bit.

(Both laugh)

GP2: Exactly, yeah, so… I probably just prefer where you just call them and… just take a history yourself.

Interviewer: Fair enough, yeah, so, um, I- I was going to ask, you talked about the guidance being not so clear at the beginning, how did you feel in the role- as a decision-maker, um, with the guidance that you had or did not have?

GP2: Yeah, it was tricky… um… I think having the limited exposure to telephone triaging pre-pandemic, during, as a GP trainee, it meant that you felt certain consultations were, you were being asked to take on more risk as it were. You know, the unwell child or an elderly patient, who, really anything could be going on, on the other end of the phone, I think in those cases it was quite tricky early on, uh, knowing how to risk-stratify that, and you know, determining whether the patient needs to be seen face to face, um… but fortunately I think in those early days, where I felt there was a need, I just discussed it with the partners in the surgery, and-and just made sure that the patients managed safely.

Interviewer: That's a really… That's a great answer thank you, and also, it makes me wonder what is your- did it change your relationship with your colleagues, or your team in the practice?

GP2: Yeah absolutely, the key thing is that everyone was in the same boat. Um, so…

Interviewer: Yeah, learning!

GP2: Exactly. All of a sudden we've gone from seeing 18 patients in a three-hour clinic face to face, um, and all of a sudden, you're making decisions, which you would otherwise- by seeing a patient in person, examining them, without uh any of that really, um… But having said that, uh, at the same time obviously it's shown that- how much you can do through just telephone consultations alone. Uh… you know, as we're taught in medical school, um, 90%

is in the history and this pandemic has obviously been a great testament to that, because most of the time, especially now, as we've got used to it, you can- find the majority of calls, you can safely manage the patient with just telephone alone, no photos, no video, um, appropriate safety net advice. I would say, kind of, as a ball-park figure, in a consultation of 18 patients, 18 telephone calls, I might only see… two in person?

Interviewer: OK, and why would you choose to see those two patients?

GP2: Just, just as a pure statistic, there will be two in any given list, on average who are unwell enough, or- or examination is needed, for example abdominal pain, or- or there's something else that requires a face-to-face consultation. And I've found speaking to other GPs, that they would agree roughly with that figure? So they would find one or two from a list of 18, roughly.

Interviewer: Alright. Um, as a locum GP, have you observed any changes in your working hours, or sort of in your working lifestyle during the pandemic?

GP2: Absolutely. Um, so uh, there was, uh, quite a dramatic decline in the need for locums early on in the pandemic so, from April onwards, the… so pre-pandemic there was a surplus of locum work, uh, at least in **REDACTED location** and **REDACTED location** anyway, but a- from sort of April onwards, so I usually pre-book about roughly a month in advance? So April was OK, but, come May there were- there were hardly any locums going around, um. But, uh…. but, fortunately, as I mentioned, that one practice were looking for a longer term locum, so I was able to work there, uh, more of the week, for-for a good you know 4, 5 months or so, and just top that up with any work that came about, but generally locum work did dry up quite significantly as a result of the pandemic.

Interviewer: Were you personally affected by that? Or as you had the 4-to-5-month placement, was it…

GP2: Yeah, I, uh- I would say I was affected. Um, so, in terms of the number of hours I was doing was… less, through no choice of my own, if I could, I would have worked the same amount as I was before.

Interviewer: Yeah, that's tough, yeah.

GP2: So, um… there was- I was fortunate in that, I know some locums, or some doctors who do- do, most of the week, they do locum work, were hit quite hard by it, but I was fortunate with than one practice, that- that did still make up about 75, 80% of my week's worth of work. So slightly affected by it.

Interviewer: Sure, I see, yeah. It's good to know, um, your… not only your experience but I'm glad that you're telling me about what you know of other locum doctors experiencing. Obviously, I'm glad to hear your experiences more, but…

GP2: Yeah, sure, and as a result of it, um… I've heard that a lot of doctors have taken up salaried roles as a result of it, so… you know, everyone's circumstances are different, some people have got mortgages to pay, got children etc. things that like that, so you know they've had no choice but to take up salaried uh, jobs, or at least a part-time one, that will provide that steady basic flow of income.

Interviewer: And are you still happy as a locum GP, has it changed the course of your career would you say, or are you…

GP2: Yeah I would say I- I personally am very happy with it, um, so this- this was sort of early on in the pandemic across the summer last year, but from about sort of autumn, uh, onwards of 2020, things have slightly picked up in terms of locum work. Um… and now I'm- currently I'm working back to pre-pandemic levels, in terms of the number of hours I'm doing per week.

Interviewer: OK, that's good then. I assume?

(Both laugh)

GP2: It is, yeah, pleased about that. I'm happy with how locum is going, uh, it is obviously not for everyone, because um, as I said, depending on your circumstances, and um, I guess your personality as well, some people like to have…

knowing there is that secure solid source of work, every week without question, and then topping it up with locums, but I- I will carry on for the time being unless things completely dry up. *(laughs)*

Interviewer: Fair enough. So, I was going to ask, so you've spoken a bit about the changes between GP staff, have you noticed any changes between GP and hospital staff? Or any shift in responsibilities between the two?

GP2: Yeah, in terms of, sort of, the nature of the work we might be expected to do?

Interviewer: Yeah, um, what you pass on to each other, and yeah, what your changing roles are, I guess?

GP2: Yeah, um, it's been difficult, because obviously referrals to secondary care have massively been affected, at least routine referrals anyway, I think in some specialities and clinics there's up to a year, a year's wait before the patient will even be seen in out-patients, uh, I think two week waits for cancer referrals are obviously still open, but once again, delayed, so most of them aren't being seen within two weeks, but might be three, four weeks, five, six weeks, etcetera. So in terms of um… uh, work we might be expected to do, I've found that secondary care might, more so than before, like I couldn't reliably say on this matter, because I think maybe a regular doctor at a single practice would be in a better position to- to say, but there might be a bit more… they might request a GP to do a few more tests, like you know, ECGs, or prescribing medication or initiating or monitoring it, and then letting a specialist know, so things like that would I say, have been a change.

Interviewer: What you're saying reflects what I've heard from salaried GPs.

GP2: Really.

Interviewer: Yeah, so yeah it's interesting to see as I get more voices, that definitely…

GP2: Yeah, what I've personally done though, with regards to the question of routine referrals, is if there is a year's wait before a patient is seen, there is advice and guidance from speciality consultants as well, where you just rather than referring the patient, you give them the clinical scenario, and just ask what should we do next, if there's

anything… I think that's been very useful, because there's been- the consultants reply to that within a week or two, um, and… it might turn out you don't even need to refer them, you can do certain tests, x, y and z, they just- they just, say if this shows such and such just refer the patient

Interviewer: Yeah, I didn't realise that was happening. That sounds like you are taking on quite a few more responsibilities then in that case.

GP2: Yeah, so that we- we could've done normally pre-pandemic as well, it's just that now, I personally am using that more than I did before.

Interviewer: Yeah, fair enough. So you've spoken a bit about, obviously telemedicine is a very big change, triaging and so forth. Have you had any experience with NHS 111, or the whole Track & Trace system?

GP2: So I, I have personally not had anything to do with that, as such. Patients obviously sometimes, I'd say more early on in the pandemic, were confused with regards to that, so they'd often contact the GP and you'd signpost them to 111 directly. I otherwise have not been directly involved.

Interviewer: Has 111 been helpful in that sense, in taking that burden?

GP2: I think early on, there was a bit of, sort of confusion with them as well… so sometimes the patient, despite having fairly, or, symptoms suggestive of COVID, being bounced back to the GP, uh… this is a bug-bearer of general practice, generally, even you know pre-pandemic, sort of general practice and 111, but if as a (*unintelligible)* for that they did need a test, or it could be covid, then you just, you had to ask the patient to call 111, because there was no provision for, you know, carrying out Covid testing in primary care.

Interviewer: Have you had any, um, interaction with the vaccination programmes, or have any of your practices been?

GP2: I don't know, I'm afraid.

Interviewer: Ok, no that's fine. I- I mean, you've sort of touched on it, but what is your response to the government response to Covid-19, in terms of public health messages and policies, like clarity, or measures I guess?

GP2: Yeah, I think, early on, there was obviously not enough, not enough clarity, on… on various aspects. I think the major issue was with the supply of PPE, which was, um, inadequate at the start. And I know a few doctors who took the responsibility upon themselves in, you know, fundraising and buying PPE to distribute across hospitals in **REDACTED location** and GP surgeries as well.

Interviewer: Really?

GP2: Yeah. So I think in terms of that, uh, it could've been handled better, but again it was a- an unprecedented time-

Interviewer: Sure, yeah.

GP2: -like no other. Um, in terms of hospital, I probably couldn't comment much on that, as far as, um, as far as that's concerned. But in primary care, apart from PPE, when you know, you come into contact face to face in GP, it's just been business as usual really, um, dealing with routine problems, urgent problems, all sorts really,

Interviewer: Yeah. Thank you, um, that's a great answer, thank you. So this is a more sensitive question, you don't have to answer it, but are you in an at-risk group, or have you experienced any issues as a result of the pandemic?

GP2: No fortunately I have not experienced anything. I, uh, I have had the first jab now…

Interviewer: Oh that's good news!

GP2: So I'm just waiting on the second one. I had that in, uh, January.

Interviewer: Me too

(*Both laugh)*

GP2: Being, at-at-risk, I mean um… I'm fit and well (*laughs)*. So I've heard that the BAME community does- does have a higher risk, but no I personally am in good health and have not had any issues.

Interviewer: That's good to hear. I'd like to ask if there's any changes, um… you personally think should be carried on in the future, or have been positive, and equally any that you would not advise?

GP2: In terms, of, sorry…?

Interviewer: In terms of, um… new policies, or changing responsibilities, is there anything you would say has been particularly positive or negative?

GP2: So I think in terms of moving forward uh, you mean how we might…?

Interviewer: Yeah that's exactly it, once we're out of the pandemic really.

GP2: I think generally, yeah, the whole pandemic has shown us how we can deal with majority of issues through telephone consultations, and I think uh, moving forward, we might see a move to triaging more, so… how exactly that will work I don't think anyone knows yet, but perhaps part of the clinic for every GP is reserved for telephone appointments, um, and then the other is for the uh, you know, routine face-to-face ones. And it all boils down to patient preferences as well, some patients, especially now, are quite happy to have a telephone consultation, it's convenient for them, uh… especially with work, busy lifestyles, etcetera, um… the ones who are savvy with technology don't mind sending in photos or doing video calls etcetera, so I think that-that aspect of that will, for the better, will change, whereas I think for some patients, the way they perceive consultations is that, from a patient- as I mentioned earlier, we know, as doctors, that history is the main element to a consultation, you can get the diagnosis most of the time from that, the examination probably is just to check one thing or another, or rule out

something, but from a patient perspective, or in the public eye, I'd say it's the exact opposite – it's either the examination element as the fundamental component of the doctor checking you, as it were, and I think when you remove that from the consultation, as has been the case, they feel- they feel that the consultation is… somehow substandard?

Interviewer: Incomplete?

GP2: Incomplete, exactly.

Interviewer: That's really interesting, yeah.

GP2: So that's more of a- from the psyche of the patient, so they feel that during the pandemic the consultation might not be- it's substandard, or it's not a complete one, um…. whereas, from a doctor's point of view, you are-you are perfectly happy with it and the diagnosis, you think you've safely managed the patient. So maybe, changing that perception in the long run? But I think with a certain proportion of patients, that will always persist, and you- you will just have to see them face-to-face to, you know, um, satisfy their expectations.

Interviewer: That's interesting to hear about doctor satisfaction versus patient satisfaction with the calls, I'd almost expected it to be the other way around, so.

GP2: Exactly, so it's the classic ideas, concerns, expectations, I suppose.

Interviewer: Yeah, a bit of ICE (*laughs*).

GP2: ICE, yeah.

Interviewer: How is it for you, being at the end of the phone line, rather than facing patients? Has that shift been OK?

GP2: Yeah so I'd say at the start I didn't like it so much, because obviously it's a big change.

Interviewer: Yeah, huge change!

GP2: Huge change, it's in our nature to not like change at the start, but as time's gone on, I- I actually prefer it I would say, it's… far more time-efficient, um… I am, as I said, very happy with using technology where needed, um… so I think there's been a shift yeah. Early on I didn't like it so much, but now I'm at a point where… and I've spoken to some other GPs as well, who feel similarly, with a thought of reverting to a full, um, session of seeing 18 patients face to face, just seems so difficult, and we think uh how did we ever do that? *(laughs)*

Interviewer: I guess it was always far too tight for time, at the time.

GP2: Exactly yeah.

Interviewer: OK, great! Um, well, is there anything else you would like to tell me about working as a GP during the pandemic? Any topics I haven't touched on that are important to you?

GP2: I think we've sort of touched on most things, I wrote a few things down earlier on, just before you came on I was brainstorming, but uh technology, risk management, examination, red sites, um… yeah I'd say the other thing, just related to what we were just talking about, is that um, looking ahead to the future, um… I think there will be a change to doing more telephone triaging, but, it also boils down to the patient demographics as well, so I think even during the pandemic it's worked relatively well in certain areas, whereas in others, it's…. not so great- telephone triaging that is.

Interviewer: Which areas do you think it works better in?

GP2: So I'd say areas where there is, the patient demographics are largely of a lower social class, or generally there is more comorbidities amongst the patients, or it's an older population group, then it's difficult, telephone triaging is difficult in those areas.

Interviewer: It sounds obvious, but what are the barriers? Sorry you're probably about to say.

GP2: The barriers might include, so they might include language sometimes, and telephone triaging is tricky as- because there's a heavy reliance in telephone triaging on communication, and getting the history as I was mentioning, uh, if you take that, or if that's affected significantly, then it, it just makes telephone consultations very, very difficult, and that's where, even if that same patient is sitting in front of you, it-it obviously hugely helps, because, um, verbal cues, body language, things like that, can um, often, um, guide you.

Interviewer: Yeah.

GP2: So, um, language would be one. Um… The other would be as, you know, sort of lower social class or lower levels of education might relate back or feedback to that idea of the patient thinking it's a substandard form of consultation, or they just insist on a face-to-face consultation, they uh, they feel they've not been properly assessed until they see you, you know, face to face, even though you might not do much, anything, different.

Interviewer: So I guess, uh, as you've said, the shift to telemedicine would probably have to come with some sort of population education about what you want to get out of a GP call.

GP2: Exactly, yeah. I suppose that's… it's always been the case, isn't it, so um… the change is not- not welcomed.

Interviewer: Yeah! I can't believe it's been a year now, you do hear the expression 'the new normal' so much now…

GP2: I know, exactly year, it's almost coming to a year… It's flown by to be honest, um…

Interviewer: It does sound like that first month particularly for you was a lot of changes happening, which probably would've happened slower- probably would've happened, just a lot slower, um… the shift to technology I guess is…

GP2: Yeah absolutely, yeah. I think they key thing was, um, the sudden change, and then the lack of clarity as well, about how to go about things, um… that sort of first month or two were very difficult.

Interviewer: Yeah. Well, this has been so informative, thank you so much. And I really appreciate you writing notes to prepare, because you raised so many important topics that I-

GP2: Well, that's OK, you're welcome!

Interviewer: Great, I'll stop the recording now.

Recordings ends.
