# Supplementary material for: Primary health care during the COVID-19 pandemic: A qualitative exploration of the challenges and changes in practice experienced by GPs and GP trainees
Source: PLoS One. 2023 Feb 9;18(2):e0280733. doi: 10.1371/journal.pone.0280733 (PMC9910752; doi:10.1371/journal.pone.0280733)
Supplement: S1 Data — (ZIP) [file pone.0280733.s005.zip › GP3 Transcript.pdf]

GP3 Transcript

Interviewer: OK, so just to start, can you please tell me a bit about your experience in GP care, and about your practice?

GP3: Um, so I only joined the practice in November, I moved there, throughout the- so I was a locum at the beginning of the pandemic…

Interviewer: OK

GP3: And then, uh… and then, there wasn't very much locum work about, as everyone kind of shut up shop (*laughs*) um, so- and I just happened to get a telehealth job, I don't know if you've heard of Livi, actually they're based- there's some **REDACTED location** practices-

Interviewer: Who are they?

GP3: Livi are like this big telehealth company, um, originated in Sweden, and… so they've got some practices in Sweden, but in the UK they're just telehealth, so basically, um… they've got various contracts, with various CCGs, which are groups of GPs, um, and the CCGs are like, if we pay you so much, however much, can you do telephone- um, video consultations for us between, so the hours are like 7am to 10pm, all the week days, and 8 to 4 on weekends, um… and so… and so… you can basically- as a Livi GP you can basically pick your hours, and so I was just doing quite a lot of shifts on there, and it's basically about as well-paid as a salaried job, um, but obviously, a lot, well- quite a bit easier, as you work from home, and there aren't any time pressures so they just pop up and it's like a **REDACTED** version of A&E where the patients sort of pop in, talk to you and go away (*laughs)*. Um, but you can prescribe…

Interviewer: How is it?

GP3: It works really well, and a lot of, because obviously a lot of patients struggle to get appointments during Covid, we had quite a lot of Covid, especially during the first wave, um… of, a lot of Covid patients, but ordinarily I think it's like a lot of skin complaints, and um, you know little things basically, little UTIs and sinusitis and skin complaints, contraception are the main- probably the main things that I see but yes, nowadays, um-

[Wifi break]

Resumed.

GP3: It was Covid-based and now it's kind of petered out back to normal presentations, I don't know what normal is! So yes, that telehealth job, and I did a lot of that in lockdown, we… moved to **REDACTED country**, me and my other half, and moved around some practices, before eventually taking this salaried job in **REDACTED city**.

Interviewer: Ok, and how's that been?

GP3: Yeah, good, really good, it's nice- it's a sort of small practice, so 5,000 patients so, it's really tight-knit, and there's not really very many staff, so you get to know a small team quite well, and they all know the patients very well, so it's been really nice.

Interviewer: OK, it sounds like a positive change then.

GP3: Yeah definitely

Interviewer: What's the practice demographic, so population demographic like?

GP3: Um, it's very- it's very mixed, **REDACTED city** is a medium-sized town for **REDACTED country** actually, and it's quite- it's probably, for **REDACTED country**, a buzzing metropolis (*laughs*) so it's quite nice, there's like, probably, an average of one visit a day between two doctors, and, and then otherwise so they manage quite well in terms of like, the extra patients who're spoken to, they're kind of shared out between the… two doctors, on average, that are there all day most days, and one nurse, so it's a small team but it works really well.

Interviewer: OK, that's a really great introduction to your experience and also your practice, I feel like I've got a really good picture of it, thanks very much. Um, could you tell me about your experience of the Covid-19 pandemic, professionally?

GP3: Um…

Interviewer: It's quite a big question, I appreciate that.

GP3: Yeah, it is quite a big question! I guess at the beginning it was, uh- there was a lot more- 'cause I'm on all these, like, groups, I don't know if you've heard of them, like 'Resilient GP' and 'Keep Close (*?*) GP Group'.

Interviewer: Oh, I haven't actually.

GP3: That's, I think that's where my husband got your little advert from maybe, I can't remember, but yes, that's probably why you've had such a big response because it was forwarded so quickly to this group of like 5,000 GPS! (*laughs*)

Interviewer: I got over 100 retweets on twitter and yeah, it got a bit out of my control (*both laugh*). But it's great, I'm really happy because I get people actually happy to do it rather than just people because they've been begged to do it, so it's really nice, yeah, it's a great thing.

Both laugh

GP3: Bless you. So yeah, professionally it was, I suppose, just like a respiratory virus initially, and uh there were like signs going up, you know, have you, just ask everyone, you know, where they've been. But we've had similar back when MERS was a big thing, so I was in my last year of- I think it was 2016… um, I was in my last year, and I was like there's thing MERS thing, so I was like maybe I'll not really, do much, as MERS did, um… and then… uh… and then yeah, it started spreading, and so everyone kind of locked down, and everyone kind of- and there was a lot of talk of people like showering fully everyday after getting home, and boil washing their clothes, and wiping down everything, and, I was just like wow, these people (*laughs*), like there's gonna be a limit to how much of this I'm gonna be able to sustainably do for the rest of my working life, so (*laughs*), so, the one- one of the things I did at the beginning was getting my practice big old respiratory masks, like FFP3, and they were properly fit-tested, like kind of do you want one too, come here for the fit testing. So they spray the little smelly thing- do you know how they fit test masks?

Interviewer: I haven't actually been fit tested, I haven't been on wards this year because of the research year.

GP3: Oh, um yeah, so I don't know what kind of treatment you would get, but the company was a cleaning company, so the cleaners had them first (*laughs*), and then- and then the GPs were like where the hell did you get these, can we get them too? And so the- the little company were like doing them, they were super-overrun because they were like properly doing it properly. So you put the mask on, and it's kind of adjusted to your face etcetera, and then they… there's like a hood, and the hood has a little window and they spray the smelly thing, so like, if you can taste a bitter tase in your mouth then the thing's getting through the seal and it's not fit-tested basically.

Interviewer: I didn't know it such an unpleasant experience!

Both laugh

GP3: Exactly, but the thing is, I got that, and I probably wore it for maybe two or three patients right at the beginning, and the problem with it is that you cannot communicate at all whilst you've got it on. They can't hear what you're saying, so there's almost no point… you're better off seeing them, like, in a car park in a well-ventilated area, if you, if you think that they've got Covid and you have to see them. Otherwise, it- it varies across the country, I know, like, with Livi and some places had hot hubs where you could send patients to get assessed.

Interviewer: Yeah. Did you have hot hubs?

GP3: No… I don't think I ever really had access to that… but then… yeah… but then people were seeing each other in the car park just for like saturations, and just minimising the chest examinations right at the beginning, um, and…. yeah that was it really.

Interviewer: So did you have much in the way of PPE then? So you had the proper masks at the beginning, so did you-

GP3: So yeah it's because we all bought our own personal proper masks, and they were supplying like, you know, I don't know if you've heard about those masks that were like out of date (*laughs*), the expiry date was covered, a few boxes of those and you're like 'yeah thanks, mate' and a few plastic pinnies, and yeah, and gloves, but, uh, I think- I don't know, it is difficult, because I was coming at it like I don't have any vulnerable, particularly vulnerable, like, household members, I don't, you know, I'm quite young, I didn't have that much of a risk, I wasn't as terrified as a lot of my colleagues clearly were about it, um, but yeah.

Interviewer: I was going to ask about that, if you were- I was going to ask later, if you were at-risk, but you're saying you weren't?

GP3: No, no, no, no. But like, I know family members, certain family members, were wiping down bags of their shopping, do you know what I mean, and because they were at risk, and you know, it's a scary thing.

Interviewer: Completely, and you don't have much information, so…

GP3: Exactly, exactly. Yeah.

Interviewer: Um, how well informed did you feel you were about the pandemic, in terms of guidance and new information?

GP3: Um, not through, like, any of the proper channels, um, but there was a lot of good collaborative chat on the social media actually.

Interviewer: OK. I haven't actually heard much about that yet, so that's good to know.

GP3: Yeah, the two main groups, you should probably join them actually, they're called 'Resilient GP' and 'TIKO GP Group', those are the two main ones, and Resilient GP used to be like a venting space for GPs that wasn't allowed clinical information, but I think with Covid, it kind of, everything kind of got spilled over, and they were fine about it, let's just do it. And there were a lot of guidelines flying about on there, and then I followed a group from my GP training group, and um, they- they- they're all London based, pretty much, and they were sharing a lot of information on there as well.

Interviewer: Great, OK. Through those, and through your own colleagues, did you feel there was sufficient social support, or sort of, peer support, really?

GP3: Yeah, I guess so, I mean GP's quite a lonely thing anyway, to be honest (*laughs*), and if you're a locum GP, it's not like you have a regular team, um… so I don't know about social support, but like, it's not, you've got your family, you've got your friends, and I think they're more supportive, than like, yeah.

Interviewer: Yeah, fair enough. Um, how did you feel making decisions with the guidance you had, it sounds like you weren't getting much mainstream guidance? How did you feel informing patients about things you were still learning about?

GP3: Hmmm, yeah, that's fine, that's part and parcel of GP really, is sharing uncertainty with patients and so I think, I, you know, that wasn't that difficult (*laughs*), we were basically chucking everyone antibiotics left right and centre.

Both laugh

Interviewer: Doing whatever you can, I guess!

GP3: Exactly, it's only when it gets to like now, a year down the line, we're like well… probably should stop that now (*laughs*) but it's very much depends on the relative incidences of the- what's- what's about, doesn't it.

Interviewer: Yeah, yeah. So, I can hear a few ways you've mentioned already, but in what ways did common practice change for you as a GP during your time in the pandemic?

GP3: Um, I think- I think I'm just a lot less nervous about prescribing remotely in general (*laughs*), um… yeah, and I think- I think you've just got to rely on patients to come back if what you do doesn't work, and I think that's like the same for a skin complaint you need a cream for… you know, you've only seen a photo, um… but yeah, you've just got to leave it with them really. Unless they're vulnerable, that's fine to do I think.

Interviewer: Yeah. Was there a lot of remote consultations then?

GP3: Mmm, I mean there still is, and I don't think that's gonna go back.

Interviewer: OK, is that a good thing?

GP3: Yes, it is (*laughs*). Because I don't think there's any point dragging people in, to sit in a waiting room, to be told 'you need a blood test', essentially, (*laughs*), whereas they could have that first, and then you can examine them with the results and actually come up with a bit of a better plan.

Interviewer: Do you- do you use in your practise, is it phone calls, video calls, or…?

GP3: Um, phone in the practice, but I still do like a day or two a week of the Livi, like the telehealth stuff, and that works quite well too.

Interviewer: OK, great. Do you- are you able to do it from home? Or is it a practice-based-

GP3: Yeah, so that's from home, and at my practice I do two full days at the practice, and then I do half a day phone from home.

Interviewer: How have you found that quite significant change, how have you found that, switching to telemedicine?

GP3: Yeah… (*sighs*), it's alright, I mean, it's not the same, but it's also not as tiring and draining actually by the end of the day, you're not as (*unintelligible*) glasses on my face all the time, which is- which is good, although um, it's difficult to get people to stop talking!

Both laugh

GP3: But then, that's true with face to face as well, sometimes.

Interviewer: Yeah. Do you think it's still accessible for your patients?

GP3: Yeah I do, and I think that's probably because it's quite a small practice and there's quite good access in general with it. And definitely, my husband works on the 111 C-KAS line, I don't know if you've heard of it?

Interviewer: Is that with NHS 111?

GP3: Yeah it's via NHS 111, but if basically you call 111 and the 111 clinicians aren't quite sure if it's Covid or not, it's the Covid assessment line, and a lot of the clinicians have signed up to work on that line during the pandemic, and a lot of GPs have ended up doing that stuff, because-

Interviewer: Oh sorry.

GP3: Because the locum situation has kind of dried up? Um, there are like 4-hour shifts on this telephone line, which is, quite good.

Interviewer: Oh actually I was going to ask about NHS 111, if you've found that you've been, um, having to, well because I know in some places they've had to take on some responsibilities, or I know you've been taking on responsibilities for them, being sent patients, have you had any experiences with 111?

GP3: No… no, only via my husband who sits on the line, maybe four hours most days, and that's it. It's quite boring-sounding, to be honest. (*laughs*)

Interviewer: Have you had interactions with Track & Trace testing systems?

GP3: No… just rubbish experiences really, via patients.

Interviewer: What do you mean by rubbish experiences with them?

GP3: Um, you know, just poor… poor tracking and tracing (*laughs*), poor advice, you know, conflicting advice, difficult to understand advice, um, it's difficult, that combined with patients not really wanting to follow advice is, is not a good recipe.

Interviewer: It's a perfect storm for frustrations I imagine. Um, do you think, in your experience, has your role as a GP changed, have you had to take on different responsibilities, or, potentially from secondary care, or have you delegated different things?

GP3: No I don't think I have.

Interviewer: OK.

GP3: No I think secondary care are gonna- always gonna try and get away with not doing stud (*laughs*), but then so are primary care, we're all a bit lazy sometimes, so… yeah, it's difficult, and there's always gonna be that divide, and uh… and I think it's fine to just write a letter back saying, you know, 'I don't think it's reasonable', or you know, that kind of thing, but I don't think it's happening any more or less because of the pandemic, certainly at the beginning they were more, not running clinics, and that caused a lot of frustrations, but I think patients were all quite understanding of, you know, the hospital doctors in the big shiny building, you know, saving lives, let them get on with it (*laughs*). But now that the waiting queues are like, a year long, they're not so haps…

Interviewer: Yeah, has that been- have there been longer waiting times?

GP3: Uh, yeah, I mean, it's out of our hands, and it's out of their hands, and a lot of people have been getting a lot more dependent on really strong painkillers because of it, but…

Interviewer: Really.

GP3: Yeah… but there's nothing else, you know what I mean, what's the other option?

Interviewer: No, course, yeah. That's a very tough consequence of very long waits, yeah. Um, do your patients come back to you if they can't get referrals, or are they more patient?

GP3: The, the thing is, mostly people have already been referred, they're just waiting for…

Interviewer: Right, yeah I understand. Have you had, um, any experience with vaccination- with vaccination programmes?

GP3: Hmmm yeah, we've both had vaccinations at our local vaccination centres, and then our practice is running, um, vaccination clinics as well, so yeah, it's working quite well.

Interviewer: OK, has that been successful?

GP3: I'd say so, yeah.

Interviewer: Have you had any role in running that?

GP3: No, I've given a few. It's not- it's mostly the nurses and the admin team that are really on the ball with it.

Interviewer: Cool! So, it's a bit, um- it's a contentious question so feel free to answer it how you wish, but what's your opinion of the government response to Covid-19, in terms of their public health policies, and in, also controlling the pandemic.

GP3: Ah, it's useless, it's just been useless, that's all I have to say. (*laughs*)

Interviewer: Ok, yeah.

GP3: Has anyone answered that question, saying 'Oh yeah I think they've done a really great job'?

Interviewer: One person! One person.

GP3: Noooo.

Interviewer: Yeah, I think, um, it's the patient- I yeah. I was going to ask if you had any advice in terms of the public health messages or policies? From your experience, in the actual clinical workforce, what would you…

GP3: I think they just needed clarity and firmness at the beginning, um… to-to just be clear, and be firm, and support people financially, and it would've been just so easy I think (*laughs*), but- but, um, they let it get out of control, really.

Interviewer: Yeah, sure, thank you. Yeah, I appreciate it's not an easy question to answer, it's a bit of a loaded one, but thank you. It's a more sensitive question, but has Covid had any impact for you personally, outside of GP care?

GP3: Um… no, I've got several clinician- well, I've got one main clinician friend who's suffering with long Covid now, so that's really rotten, lots of GP partners have had to hand back GP partnerships because of it…

Interviewer: Oh really?

GP3: Yeah, lots of clinicians have been affected, um… but the ones that I know have mostly been hospital-based. Which- which I think is a good thing that GPs have actually- I know, I know we're gonna suffer for it in terms of our PR or whatever, you know, the section that we've shut all the doors and aren't seeing anyone, um, but actually, the workforce is not significantly depleted, because of it, um… it was worth it.

Interviewer: Yeah. How has it been for you, having colleagues, um, getting ill from Covid.

GP3: Um, it's- it was actually mainly my… uh… family members, 'cause I've got a lot of family healthcare workers, so they- cousins and uncles

Interviewer: Oh, I'm sorry to hear that.

GP3: No, no, it's fine, they're over it! It was a flu-y illness for most of them thank god, but I was quite concerned- my parents, who um, run a pharmacy and opticians, and obviously they're like, in their 60s, so… I think, at the beginning I was just like 'Dad, man, get a locum', (*laughs*) so that's exactly what he did, and um, you know, for both the peaks, and then when we had this winter peak, I was just like, you know, 'do it again, like, what have you actually got to lose here, your whole health, versus, you know you're an ethnic minority, older, and you can afford it, so just do it'

Interviewer: Yeah, fair enough. It just brings it closer to home when you have family members also working in healthcare.

GP3: Particularly pharmacists, who think because they are kind of… they don't- they can't really control their exposure in the same way GPs can, um… with all our remote prescribing, they're on the other end of the prescribing.

Interviewer: Yeah, I hadn't actually considered that as much, completely. Yeah I guess they still have to have their doors open at the end of the day.

GP3: Exactly, and you know, people say they're obeying the self-isolation, but you know, guidelines, they're actually still going to go and pick up their doxycycline that you prescribed them, to cover for secondary infection, and it's just a real hassle, even though you've threatened them with a £5,000 fine.

Both laugh

Interviewer: I didn't know that was a thing!

GP3: Yeah no, it is a thing, no one knows it's a thing! They brought it in right at the beginning!

Interviewer: Is that if you know you have an infection?

GP3: Yeah, you know, if you and your whole household have symptoms, stay at home, while you wait for your test result.

Interviewer: Oh right, OK, I thought you said a fine for getting prescriptions, um, yeah I was aware of that one!

Both laugh

Interviewer: I haven't been breaking that rule! Bit suspicious of me!

Both laugh

Interviewer: OK, so from, like, talking about these changes, are there any changes which you think should be carried on into the future? Or… or you know, also not carried on into the future.

GP3: Uh, yeah you know, I definitely think the scrubs-wearing, and the telephone triaging, are all good things that will stay, for sure. It gives us a little more power back, you know before, I was getting- I was probably getting one febrile illness every month, particularly throughout winter.

Interviewer: Really!?

GP3: Yeah, I… yeah probably about, exactly-probably a 5-day febrile illness every month. Just from, just from snotty kids, I'd just not wear PPE for them, and it's just, you know, you're just gonna get it in this line of work. But, you know, I've not had, literally not had a single one.

Interviewer: That's amazing!

GP3: Yeah, it's amazing.

Interviewer: I hadn't actually considered that much either.

GP3: And like, what have you actually achieved by seeing that kid, not a lot.

Interviewer: Maybe in future, if there's more in-person consultations, it's a good thing, yeah –

GP3: -and I've got a friend in paeds and the bronchiolitis and RSV and all of that- all those infections have just gone way down.

Interviewer: Really.

GP3: Yeah.

Interviewer: Um do you think it's, sort of, affected your views on general practice, um in general?

GP3: Yeah, I think it's changed a lot, and… I think it's certainly difficult for the older generation of GPs, I think a lot, like, certainly the senior partner at my practice, is like, gagging to retire now, because he's just like 'what is the point, I just come in and sit in my room all day and go home, sit all day in the evening'. Because you lose that, don't you, you lose the face to face value….

Interviewer: Yeah, have you been alright with that? How's it been seeing less people?

GP3: Yeah I'm fine with that! Because I (*laughs*), I've never actually developed that, you know GP used to be all about the long-term relationship with patients, long-term care, relationship with the family, all of that, I like trained in **REDACTED location**, then worked in **REDACTED location**, then locumed around the shop, then, you know, and now, I'm like salaried here, I barely know anyone, let alone like who's a family, you know what I mean? (*laughs*) And yeah, I think that's going to be the case for our generation of GPs, you're going to lose that quite a bit, but I'm glad you know, I'm pleased I'm in a small practice now, so at least I get the chance, you know, it's a massive time-saver, and you know – my senior partner he can pick a patient off a massive list and tell you everything about them, whereas I… have to go onto EMIS, and click on my consultations to see if I've met them before, and you know (*laughs*).

Interviewer: Yeah, well, when I shadowed GPs, they'd grown up together with their patients, and it's crazy but yeah very nice, just um, different- different times. So I wanted to ask, you're doing the telemedicine quite a lot, how- how is the um, process of risk stratification gone for you?

GP3: What do you mean by that?

Interviewer: Have you done any triaging, or had to work out which patients need to be seen in in-person consultations?

GP3: Oh yeah, so I kind of- it's a difficult one, because I think they're probably triaged prior to me seeing them… um, and there's probably quite a bit of self-triage with that, because it's like an app, and the patient downloads it, and they-

Interviewer:-Oh, there's an app for it?

GP3: Yeah, exactly, so if you're registered with a Livi surgery that has a contact with Livi, you basically download this app called Livi, and you're like I want to speak to a doctor about this, and then your wait-time is like such a circloid, free at this appointment, at this time, and it's all kind of on the day stuff, so it's rare that you see a patient that needs to go to hospital straight away, do you know what I mean, because people do kind of self-select. I know we're kind of arsey about it then they don't, but they mostly self-select the right place to go for the right thing (*laughs*).

Interviewer: Well, I'm glad that worked out. So, from what we've talked about, obviously there's been a big shift to telemedicine, and you're in a smaller practice which I think brings unique experiences with it, is there anything that we haven't talked about that you think would be important to talk about, from your experience?

GP3: No, I don't think so… No I don't think so, just yeah, I think it's been really important to get on with your colleagues where you work, and, just to have that team spirit wherever you are, because I suppose lots of GPs will be working from home, and shielding, and trying to manage childcare, stuff like that, so it's really, it's really easy to lose that team spirit, and definitely, when we were getting our masks fitted in, definitely some of the nurses and reception staff were probably the most exposed, were like, uh, this is just so unfair (*laughs)* there's nothing to protect us particularly, and we have to deal with them just as much as you guys, kind of.

Interviewer: How did you navigate that?

GP3: I was just a locum, so I was like 'it's not up to me' (*laughs*) – step back from it, just step back from it. I was also a bit stir-y, I was like, 'yeah, you guys should get something as well!'

Interviewer: Well fair, in a situation like that, they're not wrong.

GP3: Exactly! And they're all like older women, mostly, receptionists, um, so, you know, it is difficult.

Interviewer: Completely. I'll be sure to ask future participants about that to see if they've had a similar experience. But yeah! Thank you so much, I've asked everything I've wanted to ask, and you've raised a few extra things I didn't know about, so that's really useful. This has been really good for me, thank you so much.

GP3: No, not at all! I look forwards to seeing the results! Nice to speak with you. Cool, alright then.

Recording ends.
