# Supplementary material for: Primary health care during the COVID-19 pandemic: A qualitative exploration of the challenges and changes in practice experienced by GPs and GP trainees
Source: PLoS One. 2023 Feb 9;18(2):e0280733. doi: 10.1371/journal.pone.0280733 (PMC9910752; doi:10.1371/journal.pone.0280733)
Supplement: S1 Data — (ZIP) [file pone.0280733.s005.zip › GP4 Transcript.pdf]

GP4 Transcript

Interviewer: Okay, so to start could you tell me a little bit about your experience in GP care pre-pandemic, so um, the practice that you work at, the demographic, things like that?

GP4: Um so before the pandemic, I was a GP trainee at the same practice. So I finished my training in the middle of the pandemic, and then became a salaried GP in the same practice during the pandemic so, um, before... before the pandemic, I was sort of seeing patients face-to-face in clinics as an ST3 registrar, um, and um... yeah, my- my- my I my day typically looked like, I suppose a morning of face-to-face patients and then usually, um, one or two home visits and then an afternoon of face-to-face patient appointments.

Interviewer: What does your practice demographic look like?

GP4: Practice demographic is- is mainly- mainly white British, uh, sort of working class, um... and sort of uh, a sort of low socioeconomic population and that tends to work, and this- I think, quite a lot are unemployed, but also they tend to, um, the employed, um,, patients tend to work in the local factory and also the local supermarket and retail parks.

Interviewer: Right. Thank you that's good picture of the practice demographic. Um, so, could you tell me a bit about your experience of the pandemic professionally? I appreciate it's a big question, but if you had to pick at the big changes I guess?

GP4: Um... so yeah I suppose professionally the way that we are practicing is very different, and I think it's... the changes that have been made, um, that I think, are going to stick, um, long-term into the future? Particularly as I say in my sort of working day before face-to-face patients, home visits, face-to-face patients is very much changed now, I'm spending most of the day, sort of, on the telephone and every patient that gets a telephone consultation beforehand, um... all sort of phone triaged?

Interviewer: Yeah Okay.

GP4: Then I bring in sort of a few patients, like, for example today I've seen, uh, in the morning I saw only two patients face to face, but after doing sort of roughly 15 to 20 phone calls. Yeah that's my day at the moment, and any- any home visits are kind of from the telephone list as well, so where those patients just requested to book in to see face-to-face it's now, they're all- they all get a telephone- telephone call beforehand. That's a big change.

Interviewer: What do you have... in terms of protection, have you got PPE and measures in place to cover seeing patients face-to-face?

GP4: Um yes, we have at the moment, so what we use is a sort of, the- the aprons and the face mask and gloves and I wear that for every face-to-face contact that I have with a patient, including if I'll go out on a home visit, I'll wear that. There are also visors and goggles available but I don't think anyone in the practice actually wears them? And our supply's been quite good, actually, but I do know- we got some supplies initially in the pandemic from our local factories? The Jaguar Land rover factory, they helped produce them, also a local school helped produce some PPE, and we got so- so one of our partners, um, had, um, some friends from med school who are in China, and we got some PPE sent from China as well, so we had a bit of a mix but... (*Laughs*).

Interviewer: Nice okay, I'm glad you've got that protection then. Do you have anything in the form of red zones or, um, policy for when patients come into the practice?

GP4: Um yes we do, and actually I did an audit fairly early on sort of when I a trainee at the practice, about um, sort of PPE and what we should be wearing? And I did some quizzes for the practice staff and we did, um, so I did some demos and did some acting with one of the nurses, and the nurse pretended to be a patient who had Covid symptoms and we had a separate, um, so one room we kept completely free, so one consultation room completely free and we trained our reception staff to triage, um, in a sense, of asking if they had a cough, fever or anosmia, and if they had any of those we either ask them to leave the practice if safe to do so, or if they were very unwell, we'd direct them to that, um, that one consultation room. And- and we'd call them in in the room, like telephone them in the room um... and.. . and that was sort of our- our procedure, but everyone... that was just if a patient just walked into the practice without contacting us beforehand. And that happened twice, I think, and we actually, uh... We actually had to use that protocol, but everyone else gets a telephone call beforehand, and we ask everyone, we ask to come to the practice, those questions.

Interviewer: OK. How prepared did you feel for the pandemic, um, the shift to telemedicine, in terms of guidance and for doing this?

GP4: Ummm…

Interviewer: Did you have any guidance?

GP4: We did it- we felt it was a bit- a bit patchy and it changed quite frequently early on in the pandemic which I felt was... um, it was- it was unclear at times and it was, I felt, a bit vague at times? But then I think we kind of understood that, because obviously this is all quite new and everything was coming out was sort of fairly new, and so I think we were- we were quite cautious in our practice and kind of erring on yeah, erring on the sort of safer side, I think. Umm, but yeah.

Interviewer: Um, how did you feel making decisions for the patients with the guidance that you had been given, with what you knew about Covid? Which I can imagine was a quickly changing situation?

GP4: Sorry, I missed that last bit there.

Interviewer: Um, how was that responsibility?

GP4: Um, yeah, that was- that was challenging and I think speaking to patients over the phone I think was a really big learning curve as well and I felt, like, quite lucky, because as part of GP training you- you do out-of-hours training and how it works in **REDACTED city name** is we work with **REDACTED organisation name*,* the **REDACTED organisation name** team do a lot of telephone triage and a lot of telephone triage training, like, before- before the pandemic, so I found that helpful. But it's- but in terms of figuring out, and assessing on the phone, who's at- who's at risk and who's... and which patients are really unwell, um... so... but I found that really challenging and, especially as I was- as I was still a trainee, and I think just suddenly switching and that responsibility, um, that was quite a lot of- that did feel like quite a lot of pressure? Um... and there were, I think there were times, where I was worried for the patients and they had like, in the sort of, the first peak of the pandemic, I was speaking to quite a few patients on the phone who had Covid symptoms and who had tested positive for Covid, and it was quite scary, um, talking to them, because knowing that there wasn't too much you could do other than call an ambulance and- and um, just give the- sort of the standard guidance in terms of paracetamol, fluids, rest, isolate.

Interviewer: You must've felt a little bit helpless.

GP4: Yeah and there were- there were a few patients I spoke to who passed away, um, who passed away at home um, with it, and that was- and particularly as- particularly as a trainee, that was quite hard to takes and it was- it was- it felt strange that they had passed away at home and I'd spoke to them on the phone and no doctor had seen them, and I hadn't gone out to see them and that's quite, yeah, that's- that's difficult, it's not something that plagues me but, and I know that, um, all that was done, um you know, was what could have been done at the time, and was safe, um, but difficult and I'm sure perhaps lots of other GPs will be in the same position as well.

Interviewer: Yeah, that, um so much of that does sound really difficult. Have you found that the presentation of patients has changed, since moving to telephone calls? Is it telephone and video or telephone only?

GP4: So we- there is the option to do video with us, and I think we've done- we've done a few videos but I think we all find it a bit too clunky at the moment and we... I think it takes a bit more to get the patients sort of on board with- with that, and I think because it's a little bit more of a faff I think we try and avoid it? But we do a few telephone video calls, but we tend to do a telephone call beforehand to say, would you be okay doing it as a video, and explaining how that works, so it's mainly telephone. I've only done probably, less than 10 video consultations, to be honest. Throughout the pandemic. But there is that option! I think we just choose not to. (*laughs*)

Interviewer: Ok, and how are patients presenting on the telephone?

GP4: Yeah I see what you mean. I think we definitely- at the start of the pandemic, we were really quiet, because people weren't calling us up and they did- in the first wave, and the first panic, it was quite, um, it was quite quiet our end? But now everyone- everywhere all our sort of standard patients that we would expect to be consulting are calling, are calling up. I've definitely seen, you know, the calls, for Covid symptoms have definitely mirrored the peaks and troughs that we've had nationally, in terms of the numbers of Covid patients. So a few weeks ago, um, I suppose there were lots of patients calling up with Covid symptoms and it was a lot of, um, I've always remembered a lot of like parents of schoolchildren, just anecdotally that's just what I tend to- I tend to see. Now I'm not really speaking to many patients who've got Covid symptoms, it's a mix of different presentations.

Interviewer: Great, thank you, that's a really useful answer. How would you say that that your role as a GP has changed during the pandemic. Have you had to take on any new responsibilities from secondary care or vaccinations, just as examples, has there been any change for you?

GP4: Um yeah I think there's more of a responsibility, I feel like with the telephone you- we are more... we're triaging a lot more than consulting? And I think it is, yeah, kind of making quick decisions of where patients need to go, rather than a full consultation, which is, I think a big change through the pandemic, I'm doing more triaging than consulting. Um... and... in terms of... yeah, we are doing some vaccinations, we have- in our primary care network another practice is the hub for the vaccinations, but we get- we get a very sporadic delivery from them, uh, a vial or two, uh, every week or two? So it's all- it's all a bit last minute when we get them so it's a bit panicky- but it's very ad hoc and trying to explain that to patients is tricky. We get a lot of questions from patients about the vaccinations and when they're due them, and yeah so a lot of- there's been peaks and

troughs of patients calling up and asking you know, what are the risks for the vaccination, when is it my turn, for example, and then get through onto the doctor's triage list, so that's been I think, sort of a few of us have found that quite frustrating. Um, and it's kind of, yeah.

Interviewer: Having to explain the government- having to explain the NHS decisions when they're not your decisions must be a bit difficult. I guess it depends on how much they make sense each time!

GP4: Yeah, that's true

Interviewer: Um... how has it changed your relationship with your patients then? Having to navigate these conversations?

GP4: Uh... I think it's, uh, I think patients are like starting to get used to the fact that they speak- they speak to a doctor on the phone beforehand, I think a lot of our patients like that face-to-face interaction, and sometimes it can be difficult, explaining to them that we can sort out this problem from the history and from what they're- or we can- there's different options available and that they haven't necessarily got to see me face-to-face for their problem and that- sometimes they sort of find that difficult I think? I think they... they like that physical and face to face contact, uh, with the doctor? Uh... so I think that that's interrupted that relationship a little bit um, but I think patients are used to it now, um, but uh, yeah I think that's more them getting used to the virtual platform, rather than seeing patients face-to-face.

Interviewer: Hmm. Do you feel like you've gotten used to it?

GP4: Um... I think I have? And, and I feel like I quite like it, in the sense that I'm sort of a bit more, because I'm triaging, I feel like I'm more in charge of my- more in charge of my day and I can manage my patients how I see best to, rather than having the patient in front of me, I can actually make sort of quicker decisions and sort things out, like either send a prescription direct to the pharmacy and just the whole process is faster and more convenient for the patient, and- but also, I can bring a patient in if I want them to come in and see them, and I feel like my time is more, like, more- it's more efficient, rather than waiting, you know 10 minutes for um, like an elderly person to slowly walk into the room and take their coat off, and then do their blood pressure and then put their coat back on and go… I can triage them in with a nurse or I can you know, get them, like, a home blood pressure or something like that, it just- it feels more efficient and I feel more in control of my day yeah.

Interviewer: I was going to say, it sounds like more control and efficiency! Great. Do you take the calls from home, or are you in the practice for-

GP4: Um, I'm in- I'm in the practice, I'm at home at the moment, um, but um, so yeah, but I was given a laptop actually, um, only a few weeks ago, so I've got the option to work from home, but that is, I mean really if I get- if I get Covid symptoms, so I'm doing... so we all do in our practice two week- twice a week we do a lateral flow test. If I tested positive on that, I've got the option to work from home. Obviously if I'm unwell, I wouldn't be expected to work, but if I test positive and am symptom free, then I can work from home.

Interviewer: That makes sense.

GP4: And yes, I've had had my vaccines so yeah I had my first one done in the hospital back in- in January.

Interviewer: Oh right, you were in the first roll out!

GP4: Yeah, due my second one towards the end of March, so it's- so that's good, that was really good, really happy. Yeah I jumped at- at the opportunity there!

Interviewer: Yeah I'm glad to hear that! Bit of a trickier question, but what is your opinion of the government responses to Covid-19 in terms of public health measures and policies? I'm sure as a GP you have to deal with your patients reacting to these and understanding these, so what is your opinion of that?

GP4: Hmm, I think it's tricky. I think in terms of the vaccination side of things, I- I feel like we're doing what- we're doing quite well in terms of how the government's, sort of, deals- in terms of getting vaccine supply and things. Compared to other countries, it seems like we're doing quite well with the vaccines and the vaccine delivery so far, so I think from that side of things it's good. In terms of managing the pandemic, and I appreciate it's like really difficult decisions areas has been quite tricky. Um, I think lockdowns and things previously were perhaps eased too soon, and I think, you know, it's tricky in terms of the schools going back as well, it just seems that after the summer, when the school- they decided to send children back to school and things, that- that's when the peak started to rise again, and so I kind of questioned that- that- that decision, a bit, but it's tricky because I suppose there's evidence saying that it's not necessarily schools that are causing the rise but... I find it frustrating in terms of also seeing the traffic on the roads, and I think, um, that I'm noticing the traffic on the road, because I've been driving into work, um, throughout the whole time and, it when- when, during the first wave, there was no traffic and then, it's funny that now this- this traffic jam starting to appear again and in terms of people going back to work, I mean...

Interviewer: Where are you going!?

GP4: I ask that question every morning!

Both laugh.

Interviewer: I remember in the first pandemic seeing cars, and thinking, where are you going right now? There's nowhere to go! Slightly more sensitive question, but how's the pandemic been for you personally, has it had any impact or how's your experience been?

GP4: Um, well I think personally I've been through, I just think a lot, this year, I've actually moved house, um... which so... I sort of managed to- managed to sell a flat and buy a house during the pandemic so that personally was- was quite a challenging experience and sort of going through, um, viewing houses and stuff in the pandemic was tricky.

Interviewer: Wow in the pandemic- that's quite an achievement, that.

GP4: Yeah so I'm quite happy with that, um I'm so relieved, and so, and also in terms of like my like, mood and mental health, like, and my, my sanity so I live on my own, you see, so being- I was in quite a small flat, so moving into a big, more open, brighter house is definitely sort of- I felt a bit of a lift, like and yeah. I think I'm quite a quite a strong and mentally resilient person, but I think being stuck in the flat would have been tricky, um.

Interviewer: Yeah you're human! You're super busy so living in a small space isn't ideal.

GP4: But I think as well... Yeah so I think a few of the big things, I felt going to- going to work, though, and seeing my work colleagues, so I'm very fortunate, I love the practice I work in, I get on really well with my work colleagues and consider them part-friend, part, you know, family almost and so I feel very lucky to have been able to go to a separate location and meet with a group of people, and talk to other people face-to-face like just in, like at coffee time or lunchtime, and things were all spaced out but, ug, it's really nice to have that- that chat and banter at lunch, and I think again that's something that's- that's helped me personally, through- through the pandemic, 'cause I went through a difficult time in terms of my training, because I was in the batch that had, so I don't know if you- in terms of GP training, the final exam- well, one of the final exams, is the CSA practical OSCE-style exam?

Interviewer: Oh yeah, were you switched to the RCA?

GP4: Yeah, so mine cancelled, so mine was on the 25th of March last year, my CSA got cancelled on the Friday before, which was devastating- I was devastated, because obviously like the peak of my revision, I was like bingeing all my revision! (*Laughs*).

Interviewer: Yeah you must've been so ready! You can't just be cut off at that moment, that's like when it's just one more push.

GP4: Yeah and then I had to rethink it all and prep for the RCA, which was, which was really challenging and I think was a big- a big thing to do in- just- there was- that- that was tricky as well, in terms of- you talk about sort of the government guidance, in terms of the Royal college guidance for that exam, obviously it was all new for them and I get that, but it was, yes, really not knowing what was happening and whether I was going to finish my training or not was really- that was- that was one of the hardest things for me really to deal with psychologically through the pandemic I think yeah um.

Interviewer: Yeah that sounds stressful, I understand that yeah.

GP4: And also, I suppose personally as well, another, another sort of aspect was just sort of them I restarted in a new relationship, um, so we kind of met in January of 2020 and then, just like trying to, dating and stuff through the pandemic was again really challenging, and then you know fairly early on, not being able to see each other, um... was quite- was quite tricky and yeah, yeah so that was another thing to deal with personally, but yeah.

Interviewer: Dating in the pandemic!

GP4: Well it will just be a great story to tell in the future! But we, we're going strong now, so yeah it's fine.

Both laugh.

Interviewer: I'm glad to hear it's going fine, and nice to have that company when you're working so hard I can imagine. So are there any changes which you think should be carried on into the future or equally anything that you'd like to see not carried on into the future?

GP4: Okay. Um, in terms of practice I quite- as I say I quite like the telephone tree system, I think that that- works quite well, I like that, we are using some new software as well called AccuRx, which is a messaging system, I love that. I love sending

messages- like when- when patients vitamin D comes back low, which all of them do, um, you you can just send them a message on there, or something, for example that's something I use quite often. Or sending... when I'm talking to them on the telephone triage I can send a message with a patient leaflet on, so- or or not so much a leaflet, but like a link, a web link or something with information, so I love that feature, um... and that's one of the main things that I really like I think. Yeah and in terms of things, was your question as well what not to...

Interviewer: You don't have to have an answer, but I'm just wondering if there's anything that comes to mind.

GP4: As I say I'm not a huge fan of- I think what needs to be done with the video consultation things, I quite like the idea of it, but practically it doesn't quite work, yet, or it's not quite slick enough yet. I was gonna say, a different point about, um, the hospitals, um, what I'm frustrated by I supposed is the waiting times for a lot of things at the moment, and our communication with doctors, um what I do like is having advice and guidance? Advice and guidance, so we can send messages to the specialities which is, I found quite helpful because we get a response back and I learn a lot from that? So that's good going forwards. But in terms of face-to-face appointments I don't know what we can do about it but, the communication, I suppose- the communication and referrals for our patients into hospitals is- can be difficult and something needs to be done about that, I have no idea how they're going to catch up on all the things that are still pending, especially what worries me is that the two-week-wait, uh, and how long, you know some of the breast two weeks now in **REDACTED city name** are like nine weeks or so? So...

Interviewer: Really? Nine weeks?

GP4: It's terrifying, yeah. It's scary, it is scary. And as I say, I think I think it's the same for some of the derm ones potentially? But don't quote me on that one. But definitely breast is taking some time at the moment.

Interviewer: I've heard from other GPs in **REDACTED city name** about derm being delayed, so...

GP4: But what I have found though, that with derm, is sending a photo, so that's another positive thing sorry, so taking photos and stuff on AccuRX and sending them on to the specialist has been really valuable and I found that really helpful for derm, um... and sort of sending them that way and messaging them. I get a fairly quick response back that way and, but um yeah. And I think that's hopefully prevented a lot of unnecessary referrals as well? In terms of face-to-face, I have a quick response, no that's not, uh, cancer, or it doesn't look like a melanoma or this, uh, you know something separate then- then- then you know it saves- saves them time and things as well.

Interviewer: Great, thank you for such a, yeah such a great answer, um, it's interesting I've found a lot of GPs are loving AccuRx, it's quite- quite popular. Um, and yeah I hear what you're saying as well about the triaging, and thank you. I look forward to putting these things together. Yeah definitely! Just as a sort of final question, what do you think we can learn from the pandemic? In terms of GP.

GP4: I think, um, yeah in terms of- if- if we have a future- if we have a future pandemic and this happens again then hopefully we'll be in a slightly better and stronger position than we were then in terms of, if- if there is, um, something like this again then hopefully we'll know more about sort of the- the PPE, the- the hygiene measures getting that across to all the patients and the population, and also the vaccination program and the rollout of that, um, and just how we adapt and I think- I think we were amazing, and I think it was amazing how we just adapted and changed, the way that we practice almost overnight, we suddenly went to this telephone triage. It almost- it almost was effectively overnight, the way we changed! And it was just- it was amazing to see and just, sort of, lots of these different and using these technologies to help, um, going forward, so I think hopefully- it's given us a bit of a boost really, 'cause I think we're a bit archaic in the NHS in general, hospitals and GPS, in terms of technology, and I think for the future, I think the technology side of things this has been- has been a real, sort of, bonus and lots- where we've learned a lot I think, hm, yeah.

Interviewer: Right, thank you. Yeah no I completely agree, I think, it's amazing that you guys did it over like pretty much one day, a switch to something completely new really. It's great that you had some experience from the **REDACTED organisation name** program, I've had a few trainees talk about that they say that that's been a bit of a godsend because that was their already exposure to it. But yeah just a huge change, so um is there anything that we haven't spoken about today, that is salient to you regarding the pandemic and your experience of it?

GP4: Um... I think I've touched on most of the points to be honest, I think it will be a time that I'll never, never forget, and I think will be, you know, trainees and then people in the future will be talking back about what it was like sort of in the pandemic and probably even, it will be interesting that medical students and- and- and school students will be studying the pandemic, probably in- in all subjects, and probably in like history, in medicine, in geography, in business, and you can just imagine it becoming part of like the normal school curriculum in the future. And it's funny that you'll have children, like asking us, like, what it was like in the pandemic, or almost like you know what you know, perhaps our grandparents talking about the Second World War which, which I think is- is interesting, and a fascinating time to be- to be practicing, to be honest, but yeah I just I still can't imagine in my mind what the world is going to look like in a year's time, in five years time I can't- I can't picture that yet.

Interviewer: Yeah.

GP4: It feels like we're a bit stuck at the moment, but we'll see.

Interviewer: I appreciate your time today.

Recording ends
