# Supplementary material for: Primary health care during the COVID-19 pandemic: A qualitative exploration of the challenges and changes in practice experienced by GPs and GP trainees
Source: PLoS One. 2023 Feb 9;18(2):e0280733. doi: 10.1371/journal.pone.0280733 (PMC9910752; doi:10.1371/journal.pone.0280733)
Supplement: S1 Data — (ZIP) [file pone.0280733.s005.zip › GP5 Transcript.pdf]

GP5 Transcript

Interviewer: So to start, could you tell me a little about general experience in general practice, and your practice itself?

GP5: Separate to Covid?

Interviewer: Yeah.

GP5: Sure, so, um, I did GP training as an academic GP trainee, so I did a four year training programme here in **REDACTED area name**, um, and my research at that time was mostly around, um, how can you improve end of life care for folk, um, in the out-of-hours period. At the end of my ACF I took a year out and became a fellow to **REDACTED person name** who at that point was the National Medical Director, and so I technically deferred CCT for a year. Um, after that, I have juggled general practice with a policy job, so for half my week I'm a senior policy fellow with the **REDACTED organisation name** where I run our portfolio of work on general practice, and I do four sessions a week, so two days a week, as a salaried GP. For the first year I worked in **REDACTED city name**, and then I came back to working in **REDACTED city name** because I love the practice that I trained in, and so I work in an area of really high urban deprivation in an otherwise pretty affluent city, so this practice is about 10 and a half thousand people, it's kind of homogenously a very deprived population, um, and I love it, it's a great practice to work in, it's got loads of challenges, um, but as a practice team I think everyone is- you know, in **REDACTED city name** you earn a lot less money and do a lot less work, if you want to work in other parts of the city, and my experience is that the people who work where I work really, really want to be there, um, and care about that population group? So, I find it a fulfilling place to work. It is incredibly busy, and yeah, pretty challenging, but that's kind of how my week works at the moment.

Interviewer: Uh, you sound very busy at the moment! So are the challenges due to Covid, or it more by virtue of the practice dynamic?

GP5: I think it's always been tricky, I mean like in my wider work a lot of the work I do is around equity in general practice, so we have the inverse care law across the country, so the more deprived the area a practice serves, the fewer GPs there are per head of population and the less funding a practice gets. So, it's not gonna be a unique experience that if you're working in a practice for a deprived area, you tend to have more- you're responsible for more patients and obviously people who experience deprivation accumulate multiple health problems at earlier ages, they often have- you know, in lots of ways, just very challenging lives, and… so as a GP you're dealing with that, and Covid has layered on top of that, and I'm not saying it hasn't been difficult for everyone, I'm sure that- that if I was a GP in a really affluent area I'm sure people would be having a hard time there too. I think our- our patients and our population have certainly had a really difficult time during Covid, and we've been

trying to support that and we- I'm sure we'll get into this, have massively shifted the way we work, but we're also trying to do that in a population where we know that, you know, fewer people are gonna have a smartphone than if you're work four miles down the road in a fancier postcode. You know, so, so a lot of the population, a lot of the problems we have are long standing, like most of where I work is social housing, if the government has a policy of public austerity then that impacts the hardest the communities that I work with, so the community I know has been stripped out over the last 10 years, the children's centre's gone, people's benefits are less generous than they were, you know, and all of that knocks into their health. So it's definitely not like just Covid has made this hard, this is like people who have a lot- people who have hard lives, and then Covid has made that harder, and we're trying to provide, you know, as good a healthcare as we can to them, in the shifting circumstances I think.

Interviewer: Yeah, okay, thank you. It does sound like it's complicated everything, a very complicated health demographic already. Um, well I would- you mentioned obviously a switch to using phone calls, could you tell me more about that?

GP5: Yeah, so basically, when lockdown happened last year- so it's probably easiest if I discuss maybe what we were doing beforehand, and then what we did after, just so you have a sense of how it worked. So… because one of the things with general practice is that obviously every practice is so different, so yeah, um, so the way we had it before was that I would see 12 people face-to-face in pre-booked appointments, and we had quite a good waiting time for those appointments, so I think we were over two weeks to get a routine appointment with a GP. And they were obviously face-to-face, you know normal general practice, then I would have three or four face-to-face urgent appointments, that would be booked in that morning by the duty doctor who would triage people who phoned in and said on the day, you know, I need to see a GP today, so you would have 12 routines, three or four face-to-face, and then I would have four pre-booked phone calls, um, for ones where we knew you didn't need to see them face-to-face, perhaps just following up a blood test result that was a bit abnormal or something like that. Um… and… that was repeated in the afternoon. Obviously when lockdown hit, we had to completely restructure the way that we worked to try to minimise contact, but we had quite- we're very lucky, we have a purpose-built health centre, it's big and there's lots of space but it's also very busy, so there was no way we were going to be able to safely have, you know, that number of people in a building, um… in lockdown, and normally people want to come and things. So… we… we tried a couple of different things. Over the period of the first two weeks, which was kind of chaos and carnage, we tried to move everything to a phone call if we could, but some people were still coming in face-to-face, because you've kind of got a lag time, because you have people who've booked appointments 2 or 3 weeks in the future.

Interviewer: Right, OK yeah.

GP5: Does that make sense? Because lockdown happened quite quickly and we hadn't yet changed the system, we had a couple of weeks where had appointments booked up, so there was that group of patients to kind of work through, and of those we swapped as many face-to-face as we could, and what we said was we said, OK, from, you know, from 2 weeks post lockdown, everything is telephone first. So the way it now works is that everything is on the day telephone first. So if you phone in and you say you want to speak to a GP, you will speak to a GP that day. So it's completely got rid of our waiting list. We've got other challenges, but one thing we've completely solved is we've not got a waiting list, if you ring us you can speak to a GP that day. You'll be booked into a phone call, so my session now looks like… four pre-booked phone calls, which GPs can book to follow up patients, and then 13 or 14 on-the-day phone calls. So that's- a patient rings up and says I want to speak to a GP, they won't be told you'll have to wait 2 weeks, they'll be told 'Yep, Dr **REDACTED participant name** will ring you back later this morning'. And… then if I speak to them, and I think they need to be seen face-to-face, and they can be safely seen face-to-face, ie. they don't have Covid symptoms in the home, then I'll bring them in to see me. So those are kind of extras on your surgery, and it is sort of pot-luck. Sometimes you might have to bring five or six people down to be seen face-to-face, sometimes it's none at all.

Interviewer: Do you bring them down the same day?

GP5: Yep.

Interviewer: Ok. And how's that change been, because that's a significant shift in how you deliver care?

GP5: It's been a massive shift in how we deliver care. Um… I think there are, it's probably easier- that's a massive question, it's probably easier to break down into frames, like who does it work well for-

Interviewer: Ok yeah, tell me about the accessibility of it.

GP5: Yeah… I think, for patients, the accessibility, if you can access us like this, is great. You know, if this works for you, it's incredible because you can ring up on the day, you'll speak to a GP, if you need to see us you'll see us, we also across the city, we have a federation of GP practices, so 14 practices across the city set up a federation and we have- we set up a Covid service that we run from a separate practice, so we have a system to see patients with Covid, um… at a separate site. We have a Covid home-visiting service… So if you're a patient who can access us, I think it's probably great. That leads to several challenges though, one of which is supply-induced demand. So… a lot… not a lot of… I have to be a bit careful of how I phrase this, but in the past, a weight was slightly therapeutic for some people. So they might ring us, book an appointment for 2 weeks later, but then actually not need the appointment and cancel it, because the problem had resolved. Or they might have a problem and

they might be right at the beginning, and by the time they speak to you, actually it's a bit clearer what the problem is, because now you have a situation where someone maybe has a problem at 2 o'clock, phones you at half past 2, and you're very, very early in the path of physiology of whatever that thing is, and it can be quite diagnostically difficult. Um…

Interviewer: No one's actually said that yet, that's a great point.

GP5: Yeah, it's interesting, and like- and obviously people are so different. Sorry I struggled to hear you for a sec! (*Wifi lag*)

Interviewer: No I just said just how immediate the care is, no of course. I hadn't considered this!

GP5: Yeah exactly, it's a funny thing, I think you really appreciate- I remember being completed flummoxed as a medical student when I first did an A&E placement, and being like, 'Oh right, people come to A&E for this!'. Because all you really know is like when you would go to A&E, or your family, or you know your friends might go to A&E, so it can be particularly challenging for example, talking to someone over the phone who rings you up and says, I've had stomach pain for an hour. And… trying to differentiate- now of course stomach pain for an hour could be that you've perforated a duodenal ulcer, it could be that you've suddenly got a horrendous- that you've got ovarian torsion, there could be lots of quite significant- ruptured ectopic! You've got lots of serious causes of stomach pain in the course of an hour. But you've also got- I ate too much at lunch, and, I'm constipated, and I just need to pass some wind (*laughs*) you know you've got this massive spectrum of things that can cause stomach pain for an hour, and they've called you very, very early on! If you've got an obstruction, at an hour you might not have vomited yet, but if you speak to that person in 3 hours time, and they're vomiting bile and they've not passed any wind and they've got a really bad stomach, then you're going to be like yeah, I'm worried that that's a- that that's obstruction. So I think in some ways, rapid access to diagnostic care… can- can come with its own diagnostic challenges, and it can certainly come with capacity problems, because… those people- that's an appointment. Um… and… and then the flipside of this is that I do worry, we all worry, about the people for whom accessing care in this way is more difficult. So I think, for some people this works incredibly well. They don't have to- I think the average time, this might be from the states, but you know, people on average apparently take 2 hours out of a working day for a GP appointment, by the time they've got to the surgery, you know left a buffer of time, been seen, come home. For someone that's able to take a call at work, to be able to just be rung, have- you know, it's 10 minutes, it's discrete, it's the time you, you know, or 15 minutes or whatever, but it's a small amount of time, a huge time saving. But… I worry that some employers are… that that's easier for some people than others, fundamentally, um… and also, there's the whole kind of digitial exclusion thing. Like lots of our patients don't have mobile phones, lots of people if they do have mobile phones are on a cheap contract where they don't get lots of data, or where, for example, it costs you to access a voice message, um… we have lots of people who just have landlines, or don't have reliable landlines, you know, so it's

the kind of- is it difficult for people to actually access care, and then there's a whole other layer of this, which is… people who aren't presenting because of the fear of Covid, um…

Interviewer: Yeah. In your experience, are there people presenting less where you'd expect them to, potentially because of accessibility problems?

GP5: Yes. I- I think so. And it's difficult my experience is of course when they have eventually presented. But I have definitely had memorable consultations during Covid where I've spoken to someone who pretty evidently has had a stroke, for example, you know, like- and… has delayed seeking care. Or… episodes where people have symptoms that would worry me as red flags for cancer, but I- I might say to them, have you been worrying about this?. Just, 'cause- that's an interesting- to me that's an interesting question, like no- they actually might be like, no, I've not been worried about this, but my wife or someone said I should ring you this morning so I did, but actually I've had consultations in this where I've said, have you been worrying about this, and they say, yeah I have been worrying about this I just didn't want to bother you guys I assumed you'd be really busy. Or, 'I was scared I didn't want to have to have tests at the moment'. So I think, yeah, and it's been brought out in the evidence now around, you know, delayed presentations of cancer and stuff like that, but I've definitely also experienced it. And I've also had some really challenging conversations with patients about admission to hospital, um…

Interviewer: In what context have they been admitted to hospital?

GP5: Patients who have, um… acute medical problems. Who, you know, need to- I really remember a conversation with a patient who I've known for a long time who has really quite severe COPD, and we have quite a high limit for getting him to hospital? Ie, we know him really well, and we know his sats really well, and he can tolerate quite a lot at home, and we can keep him at home for quite a while, but you know, at a certain point he really does need to go to hospital. And he'd hit that point of really needing to go to- you know, there was really nothing more that we could do, um, and his sats were still, you know, in the 80s, well below his norm. And he was terrified to go to hospital, and I kind of- they're really difficult consultations. And I think the other thing that you have to weigh up, I was thinking about this on Monday night, I had a patient last thing of the day, the lab phoned me to say an elderly chap had a sodium of 121, um… so that's a very low sodium. And he's- I don't know him well, but I looked through his notes and I could see he'd had some low sodiums before, but all the culprit medications had been stopped. So there wasn't an obvious reason for him to have this. And I rang him, and he felt completely well, he wasn't confused… you know like there was no obvious reason to send him to hospital. And I find that a very difficult decision, because in a normal time, when there isn't- you know there's always some risk of infection from a hospital, right, but it's low, certainly relatively low compared to now. You wouldn't think twice about recommending to this guy that he goes to hospital.

Interviewer: Sure, and investigate it

GP5: Exactly, but in this context you've got a really alarming blood test result with a patient who says they feel completely fine, and this risk that I send this guy to hospital, and actually he gets Covid! And he's 91, maybe he dies of it. You know, so it's- I think it's challenging for patients and it's challenging for clinicians, and I'd just add that- that challenge for clinicians, we've definitely noticed the face-to-face thing, bringing people for face-to-face appointments, it's much easier when Covid prevalence is lower. You feel much happier bringing people down for face-to-face appointments, particularly our older shielding patients, I- I feel much happier saying, yeah just pop down and I'll have a look at you, because we know the risk to them is lower- it feels much weightier making that decision to bring them down for their (*unintelligible)* for us I think, when you know, in January when Covid rates were really quite high.

Interviewer: It sounds like you're making, yeah, what would normally be small day-to-day decisions have become huge decisions with massive implications depending on when you're making them. Um, where we're at now, so hopefully this will decrease. I hate saying it, it feels too risky to say! But how's your care standing at the moment, do you have many in-person consultations?

GP5: So yes, interestingly I was looking at NHS- with my policy hat on- so yesterday we had more data- data out for January's appointments. NHS Digital Appointment data suggests that overall rates of face-to-face consultations have stayed more stable in this second lockdown, then they did during the first when they really dropped off. So I think that's interesting, particularly we'll know better in a month's time, when we get February's consultation data out, how- how true that stayed. I think anecdotally for my practice, we've definitely seen way more people face-to-face in this second lockdown than we did in the first lockdown, even though actually Covid rates and Covid risks I think have been higher. Um… and I don't know if… I suspect that's multifactorial, I suspect it's partly that there's this iceberg of unmet need from the first lockdown that's still presenting, and I think it's also people acknowledging that there's now a durational aspect to this, that this is like, this is going to go on for a while, so you can't just keep deferring things for people. There comes a point where that has to be factored into the risks and benefit trade off as well? The other thing that's helped is we have lateral flow testing now.

Interviewer: Oh right, ok, in the practice. How do you use that?

GP5: So we got lateral flow testing in the middle of January, we were each given a box of 50 tests, we are meant to do two a week. So we don't get told when to do that, but we logically- so for example I work on a Monday and a Tuesday, so I do a lateral flow test on a Sunday. Um, to make sure that I'm negative kind of at the point- the only reason that I don't do on Monday is that

I get up at 5am to get to work, so, I don't want, to get up at half four! (*Laughs*). But we're kind of using them, so for example we run vaccine clinics from our surgery, we're a vaccine site for anyone that comes in to vaccinate, and all the volunteers, everyone does their lateral flow test. Of course it's not 100%, and of course anyone who is symptomatic wouldn't come anyway, but it at least gives a bit more of… so far we've had one staff member test positive asymptomatically in the six weeks we've been doing it. So it gives us a little more security in knowing that we are not Covid positive.

Interviewer: Right yeah, well I'm glad your practice has that, it's just a little bit of security like you said. How, um, how supported have you felt in terms of PPE, so physical, and also emotional support during the pandemic?

GP5: Um… PPE-wise we've been fine. So we've always had enough and we've always used it for all patient contact, like I've had no personal challenges, kind of, sticking with guidelines on that. Obviously there's a significant debate about whether the PPE we have is adequate, but we- we haven't had a shortage of the PPE that we've been given, as it were. Um, and… emotional support… it's interesting, as a team we are way, way tighter, and way closer now, than we were pre-pandemic. I think… we've really… it's a classic, like people kind of feel like they've been through a lot together, and that brings people closer together as a team, I think there's a sense of people taking risk as a team as well, like, in some ways we're each other's bubble, we don't- it's funny, it's harder to meet, we don't meet, all of our meetings are on teams from separate rooms, like we are… we feel closer despite being more distant.

Interviewer: It's nice, and that's an experience I've had reflected in a few interviews as well is that people do turn inwards to their colleagues. Um, and it sounds like it strengthens relationships between colleagues. Has it changed your relationship with your patients?

GP5: Uh… that's a really good question. I miss them. I miss some of them. Um… I… I actually prefer, I think on balance, practicing I this way, I…. was… quite worried about it for a while, I was like well this is weird, this isn't what I trained for, like I didn't train to spend my day on the phone and I was really aware that telephone consulting isn't- it's a very different thing from face-to-face consulting, and I didn't want to assume that just- like whatever proficiency that you had in face-to-face consulting would just translate to being a good telephone consultor, and… so I kind of wanted to work and continue to want to work at, like, how you get better at telephone consulting, and how you… on all levels, like how do you become a better diagnostician, how do you become a better historian, but also how do you become better at building empathy and… you know I think the Americans call it website manner.

Both laugh

GP5: But uh… so those have definitely been things I've thought about. I…. and the plus side of consulting like this is that it does feel less stressful, it does feel like I can take more time with people. Um… and I think the major difference with that is just not having time slots? So, before I used to find it quite difficult to ignore the little counter in the top of your EMIS screen that tells you how many patients are waiting.

Interviewer: Of course, yeah, it's stressful to have that there when you're tyring to connect with a patient, thinking about the waiting room, and I guess you would have a physical waiting room, so-

GP5: Yeah, exactly! And you'd be like, there are three or four people waiting now, sitting there and, you know, um, whereas now I just- each call takes as long as it takes, and I do the next one when I'm ready, you know, and I have more control- I definitely am a lot more efficient, like my surgeries aren't taking less time, I find it a complete misnomer that if we all swap to telephone consultations it will be quicker. My experience is not at all. Like my telephone consults- by the time- my telephone consultations are often longer and I don't think that's surprising, I think it takes a lot of time to kind of make up, in some ways, for the information you're lacking.

Interviewer: Yeah that makes sense. Have you had any guidance for it, um you spoke a bit about triaging, so you're trying to sort of risk-stratify, have you had any guidance for how to do that over the phone?

GP5: Not really. So at the start, 'cause I also worked in a Covid, in the Covid hot hub, we called it, I worked in the Covid hot hub at the start.

Interviewer: Ok, I'd like to hear about that after!

GP5: Yeah so in that job where we were trying to triage Covid patients, um… there were loads of scores, you're probably aware of like the ROTH score, and then there was a phase of, should we use the NEWS score, and there were basically loads of ways that were kind of evidence-based for 5 minutes, of how to evaluate someone with breathlessness over the phone and… so we tried all of them, um, but actually most of them haven't stuck. That said, there were some really good articles, um, mostly from the BMJ, Trish Greenhalgh did a few of them, that helped us, or helped me, you know, kind of, how to triage patients with Covid, and I used those as, yeah kind of a safety net, I guess, to be like these are the questions. The other thing I do- and I've always done this, I never kind of, I've tried to fight feeling ashamed of looking things up, like there can be a lot of pressure to just know things, and I've always felt that that's completely ridiculous, because surely if what we want is to deliver the best, most

evidence-based care, to our patients, we should be looking at that evidence base, not just relying on our brains, which are never going to have as much- we've got the internet sitting in front of us, why would we not want to look things up? But I think there can be pressure from patients, and from ourselves, not to look things up, and I've always tried to resist that, and I've often used NICE CKS and all sorts of things while I'm consulting with patients. It is even easier to do that over the phone. Because I can be talking to them, and I can have NICE CKS up.

Interviewer: You're not the first person to say that!

GP5: Ah, that's nice.

Interviewer: And they say it as a sort of confessional, like um, exposing- but yeah, everyone does it, why wouldn't you? It's guidance that's been released for this purpose, so…

GP5: Exactly it's great! It just makes things easier, and before when thing were face-to-face I think I'd signpost to the patient what I was doing, now I kind of don't even need to, I've got 2 screens which is great, so I can just have their notes, NICE CKS or whatever the tool is I'm using, and I- I find that reassuring.

Interviewer: Great, OK, well thank you for that, that's a really good answer, thank you. Um, I would ask, I mean we've covered a few things by nature of conversation, but how has your role changed as a GP, would you say? Have you taken on any new responsibilities, I know we've touched on a few, or transferred from secondary care and so forth…

GP5: So I worked in a Covid clinic for uh… for the first wave, as well.

Interviewer: The… hot hub?

GP5: Yeah, sorry I'm using words interchangeably, that's not helpful.

Interviewer: No, no that's fine! I'll get used to then,

GP5: Um… so that was the first thing I did, so in that first wave my policy job very kindly sort of said go and- go and work for the NHS, that's absolutely fine, um, and so the way that I did that was I did my normal two days in general practice, and I did the rest of the week in the Covid hot hub, and the reason for that was basically, I mean A) I wanted to do it, B) all practices were

kind of asked to volunteer staff to do it, and I, um, I am young and fit. Kind of, as risky people go, I'm young and fit, and I live with my partner, I don't live with an elderly relative, or you know. I was happy to go and work there, and so… is it helpful to describe kind of what the clinic did?

Interviewer: Yeah I'm interested in your experience of the year, so yeah.

GP5: So basically it was set up to try and- different cities did it in different ways, different places did it in different ways, the way we did it here in **REDACTED city name** was to try and set up a service where patents who had Covid you needed to be assessed- I guess patient who had Covid fell into three broad categories, the first category was they could speak to their GP and their GP could manage them over the phone, the second category is that they spoke to their GP and they clearly needed to go straight to hospital, and then the third category was they spoke to their GP and it wasn't clear, um, and so the way, so we set up a special service for those patients. And in fact, what we used it for was not just patients with Covid symptoms, but anyone who had Covid symptoms in the home. SO let's say it was a kid with some abdo pain, but the Mum had a cough and a fever, they would come to the Covid clinic so the idea was that the other practices across the city stayed as clean as possible, and everyone who had possibly got Covid came to the Covid clinic. So it meant you could keep patients coming to the other surgeries safer. And so I worked in the Covid clinic. And it was hilarious at the time, we found it completely surreal, kind of the ways we were working, each of us in a separate room, you trot out to the- so people would arrive you re-triage them over the phone, get as much of the history over the phone, then examine them in the car park, that was reasonable, or examine them inside and- you know- it was just this completely different way of practicing, it worked really well, we still- we run it all through the- well it's been running all year. It's obviously been way busier through this winter, um, again, I only did it until June, um, and then I stepped back- or maybe May, and then I stepped back into my normal policy role. And now I'm doing lots of vaccinating stuff.

Interviewer: OK. Are you vaccinating through your GP?

GP5: Yeah so, we are… all kind of vaccinations are happening through primary care networks, it just means that within our primary care network, the practice I work in is the vaccination site.

Interviewer: And how successful has that been in your practice?

GP5: It's going really well, and I think another thing that is kind of, frankly I think it's been really kind of psychologically helpful for us, it's weird, it's been loads of work, but I actually think it's been a good thing? Like we've been really busy with other stuff, like, which I think is a good thing, we haven't seen this massive drop-off in consultations that happened in the first wave, we

stayed really busy through this winter, and it's been different, we're not seeing communicable disease in the same way because, you know, I've not seen a bronchi-baby all winter, that's insane, like never- I hope I'm never gonna have a winter again with no bronchi-babies and no viral-induced wheezes, because the reason that's not happening is they can't go out! (*Laughs*). But… at the same time… so we've been really busy, and I think it would have been just so depressing and horrible had it not been for the vaccine clinic, so even though they're loads more work, they're also kind of joyful, and they bring hope, and the practice team is working together, and the thing that's really nice about it that I've enjoyed is that, if I'm vaccinating, we have another person in the room who's the administrator, and that role is being done by practice admin teams mostly, and I feel like a lot of the time, they- they don't get seen by patients, they do this huge volume of work, but they don't get seen, and they kind of- GPs, and nurses, and the kind-of patient facing people get all the credit? Whereas in this, we've got- I'm there, and my colleague is there, and we both introduce ourselves, and we're there as a part of…

Interviewer: The practice is presenting itself, at the forefront.

GP5: Yeah, and I think it's been… I think it's been a nice… thing for us to do. It is busy though. Like, there's no (*laughs*)

Interviewer: Yeah I see what you're saying! Well it's great to hear, and I'm glad that people are taking up the vaccines as well.

GP5: Yeah, we are literally consulting from broom cupboards around the rest of the surgery to try and make space, but that's fine, you can do a consultation from a broom cupboard! (*Laughs*).

Interviewer: Have you done any from home? Any work from home?

GP5: Yeah, so in that first face, when I was in the Covid clinic, I did my two GP days from home. So I was the only GP working in the Covid clinics, the idea was basically that I was contaminated (*laughs*), so for me then to not go into the surgery. I mean it was like a technological nightmare getting set up, but… yeah, I- I did that for a couple of months. I found it quite strange, and it's not something I would personally be necessary keen to repeat, um, but it was kind of- it was necessary, and seemed sensible at the time, and I came back to practice when… they asked me to come back at the point where… face-to-face- the need for face-to-face appointments had picked up, to the extent where… they needed another person back in the building to see the face-to-face people, because obviously when I was working from home, I was only doing the telephone stuff for the surgery. The thing I found hard about that actually, was that if I was consulting from home, and somebody needed to be seen face-to-face, I'd have to book them in with a colleague. And I found that psychologically harder, because then what I was asking them to do was- then I was basically putting a Covid risk onto a colleague.

Interviewer: Sure that must be really difficult.

GP5: It felt like a different situation to just me consulting and being like, yeah I want to see you.

Interviewer: Mm, yeah, evaluating someone else's risk is very different to your own personal risk. Um, yeah it must've been quite difficult for you.

GP5: It was a funny one, you don't think about these things until they kind of creep up on you, because why would you, it was a very strange situation to find yourself in. Um, but I did find it challenging at the time, and I find it kind of much, much easier now where I'm just taking on risks for myself. And the other thing I think is… you know, we're nearly a year down the line, we understand so much more about Covid, and we've probably also become a bit more comfortable with risk, and now we're all half-vaccinated, like, it… it probably feels a lot safer for lots of reasons? Um, then it did back in the very early days, when we had so little information, and there was no community testing for patients with Covid, let alone for staff, and…

Interviewer: Yeah, yeah it's a very different place now. I'm aware you've got a meeting in 10 minutes, so let me know when you need to go. I wanted to ask what your opinion is, obviously it's a slightly loaded question, but what's your opinion of the government response to Covid 19? In terms of public health messages and policies. From your experience of a GP, having patients come in with the information that they're getting.

GP5: Sorry just- you said, what's my opinion on, and then it cut out for that bit of the sentence

Interviewer: Sorry- government response to Covid-19. In terms of their public health policies and how that's reflected on your patients coming into the practice.

GP5: So…(*very long pause*) I…. have very low expectations of this government, and those low expectations have been exceeded. Um, as in I think they've- I think it's been a disaster. Um… (*sighs*) I… it's hard to know where to begin to unpick that question Minka, it's a really big and important one. And I think that we will have to go back and look at- you know, there will have to be a huge enquiry, and it will also have to look at what was the NHS's role in this, because I think as a country, we always have British exceptionalism right, we think we're much better than we are, but we have catastrophically underperformed, for Covid. Not just in terms of the number of people who have died, but the amount of, I think suffering within communities, that potentially was preventable, um… not just from Covid itself, but from the economic impacts of Covid. And… yeah. I… I personally think that this

government has underperformed hideously, I think there are a lot- there are some kind of real points that I would pick out, the first being the first lockdown. Like- it just coming far too late. And the kind of avoidable mortality that happened because of that. And I think the stuff about care homes very early on, um… the kind of failure to appreciate that we were letting Covid run rife in very vulnerable institutionalised populations that could and should have been protected. I think the initial, um… I mean it's a great question, there are so many ways to…

Interviewer: Yeah it's why I tend to say public health policies, because it's just- there's just so much to unpack in it obviously. But I appreciate your answer, especially in terms of care homes, I've had a few people mention this. Um, is your GP practice associated with care homes?

GP5: Yeah, we look after two, and one in particular has just had loads and loads of death, um particularly in the second wave. Um, I think the care homes one is a massive one, I think you have to look at the overall lockdown strategy, and the balance of economic trade-off in public health policy, and public health also to me means how you support the least privileged communities, because you can't separate income and poverty from health in my mind. And so, failing to economically support deprived populations I think has been massive, one of the biggest public health failures, I think, , is the failure to support people to self-isolate, because that really, really impacts on the lowest-earning workers, fundamentally. You know, if you say to someone who is earning a fee for service on driving a van, or something, that you have to take two weeks off work or 10 days off work to isolate, and you don't offer any support, we can't- I don't think we can be surprised that people still do that, but we haven't. So there's obvious black holes, and then there's really predictable things that have really upset me, so… for example around the vaccine programme which I've… kind of been involved in… eventually ended up involved in the policy making because I got frustrated with it, um… was that if you prioritise chronologically by age, you discriminate against poor people, and minority ethnic people, because fundamentally getting to be over 80 is a privilege that is predominantly associated with wealth and whiteness in this country. So…

Interviewer: Yeah that's a brilliant point, it doesn't get raised much at all.

GP5: Yeah so like yesterday we're now down to vaccinating cohort sic in our group, in our practice, which is where national guidelines are. Um, and, all of a sudden we are getting many more black and minority ethnic people to vaccinate, which is precisely- we're now at a younger cohort so there are more people in that cohort who are not white, so in the older cohorts what it means is that you're prioritising vaccine supplies actually to affluent areas because you're- if you're over 80, what it means is that you're twice as likely to live in an affluent area than a deprived area. And of course if you're over 80 and you're my granny, then you can self-isolate and get your delivery in from Ocado, you're relatively safer than someone who lives in a big

intergenerational household where someone has to go out to work on a building site. So- so these things kind of- it upsets me because those are decisions that are taken well after we already know how Covid is playing out for deprived and for minoritized ethnic populations. So you could’ve predicted that that was what was gonna happen, back when the pandemic hit, based on everything we know about the inverse care law, and how access to care works. But then we had great evidence for it in the summer, and then it feels like at every opportunity public health wise, the same populations get shafted. Um… yeah. Sorry it’s a little rant.

Interviewer: No, it’s a great rant! I’m aware that your meeting is about to start, so I should probably let you go.

Recording ends.
