# Supplementary material for: Primary health care during the COVID-19 pandemic: A qualitative exploration of the challenges and changes in practice experienced by GPs and GP trainees
Source: PLoS One. 2023 Feb 9;18(2):e0280733. doi: 10.1371/journal.pone.0280733 (PMC9910752; doi:10.1371/journal.pone.0280733)
Supplement: S1 Data — (ZIP) [file pone.0280733.s005.zip › GP6 Transcript.pdf]

GP6 Transcript

Interviewer: Ok, so now to start, could you tell me a little about your general experience in general practice, and about your practice itself, pre-pandemic?

GP6: Ok. Um, so I'm… a salaried GP, I work seven sessions at my practice, I also have other portfolio roles, um, but mainly related to the GP stuff. Um… our practice is quite small, it's 4,000 patients, um, and it's, um a mixture of some deprived patients and quite a lot of elderly frail patients, which is predominantly my special interest. Um, we… we have a group of certain individuals who have some substance misuse problems, although we don't run a service of that respect. Um… but… um… we generally have more older people. We are part of a primary care network called the **REDACTED name**, um, which is a group of 11 practices. It's a group of 11 practices that sort of work together to provide extended hours work. So during the pandemic, we sort of joined forces as well. So um, when… pre-pandemic, we would do, um, face-to-face consultations mostly, but we would call people ad-hoc, not as proper slots on our records but as ad-hoc to tell them about blood results, or an X-ray or whatever. We didn't use any text messaging services, um, we are an EMIS web practice, um, so, with something like system 1 we have an in-built SMS service, but we didn't have that. Also, we had Skype pre-pandemic, but hardly ever used it. So it was part of a trial, the practice used it in pre-pandemic in 2018, but I never used it while I was thee. Um… post-pandemic, we switched literally overnight to remote consulting, and we call everybody first, and then based on what we think- if we need a face-to-face review, depending on what their, um, what their presenting complaint is, then um, then we'll bring them in. And what we do is we run a concurrent slot face-to-face, um, appointments, so we- so there's four of us doctors, and there's usually three of us in on a day, and we'll all have our separate lists with telephone calls, and if we want to see someone face-to-face, we block off one or two of the slots, depending on how- how much time that face-to-face might take, and then we book a concurrent slot in the face-to-face list and then we use one room in our practice to see them. And we just make sure we don't sort of cross paths with those patients. Um… so we think that works quite well for us, and we haven't- touch wood- had any problems with that yet. We also now have AccuRx, which is a text messaging and video messaging- video consultation service, which is- has literally changed my life and I think it's amazing. (*Laughs*). And the patients love it. So the patients love the flexibility and things as well there.

Interviewer: That's great to hear. So you're using that for the majority I guess, how many of your consultations are face-to-face now would you say?

GP6: So face-to-face, um let me think. So if I have about 30 contacts in a day, I'll have… maximum- well, maybe two or three face-to-face on an average day? So about 10%. And then… (*sighs*). Some days are a lot more heavy with face-to-face. So I've had six patients in on a day before. But they haven't all been generated from the same day, they may have been generated from a

previous day or a colleague… we have a male doctor who may have transferred some of the female patients who want a breast exam, or an intimate examination like a vaginal exam, so… will put that onto my list. So we'll see those ones as well.

Interviewer: Ok. You said there was the three on at a time, out of four? Is the fourth person at home?

GP6: Yes, so it's usually their off day, 'cause everyone's, um, part-time, so myself and the other salaried doctor are… we work almost full time, so we're seven sessions, and then the two partners are five sessions each. So we just cross- there's usually three people on in a day. Which is enough.

Interviewer: OK, great cool. So these changes to telemedicine quite positive, have you had any limitations to it?

GP6: Um… some patients haven't found- (*sighs*)- although they've- although they've found that it's good to be able to get an appointment quite quickly now, some people don't like talking on the phone, they want to see a doctor. And even though 80% of things we can probably deal with on the phone, because predominantly- you'll learn this in medical school obviously, the majority of your, um, management actually comes from the history? You'll get your diagnosis and everything from there. Patients actually like seeing the doctor, they're isolated and alone at home, they want to see someone face-to-face and tell them what the problem is, because that's they're used to? Um… some people who have impairments of some description, so hearing impairments particularly, people with arthritis who can't text and send you a photo, or… you know, they can't use a mobile or whatever, they struggle a little bit. Um… those with memory problems also struggle. Um… language barriers – although actually not that bad, because we've really gotten into using Language Line or BILCS, and um doing conference calls? That actually works really well as well.

Interviewer: Ok. How do you manage, um, so in terms of people who do struggle, how do you manage that situation?

GP6: I have quite a low threshold to bring patients in, face-to-face. Because for me, patient- patient safety is most important. It's not that I want to- you know, I still want to do the remote consulting, and predominantly we do do that, but if it's a frail older lady who cannot articulate what her problem is, or can't send me a photo, you know I tend to just bring them in. And when we bring them in we tend to do a bit of everything, so we'll do- we'll review all of their problems in one go, 'cause we can, 'cause we've blocked off slots and we can give them that time, um, but also if they're due any bloods for their annual review, or blood pressure or something, we'll just do all that at the same time.

Interviewer: Ok, that makes sense. Um in general, this is a slightly different question, but how informed did you feel about the risk of Covid 19 to yourself and to your patients?

GP6: Um, so early on… I think… I think I've been pretty informed, 'cause we, you know, we've got a lot of information, and every day there's something that changes, so at the start of the pandemic, I would, um, join in with a lot of the, um, the primary network calls, 'cause we used to have Zoom calls about what was going on, so that was good, and then we obviously had lots of emails filtering through, and just our general research as well. But it got to a point where actually I was just getting overloaded with information, so I would just not take part in the evenings, and I would just do my own research and read my email and things, and just stay informed in that way.

Interviewer: Where did you find that information?

GP6: Um, sort of various sources, so whether it was emails being disseminated through the primary care network, NHS England, from the Government, Public Health, just my own research, um, and things that people had collated together in various resources for GPs. So, things like, um, GP notebook, NV Medical Reviews, Medwell Reviews, things like that. And even things like Facebook! (*Laughs*). So reading on social media as well.

Interviewer: Yeah, fair enough, it's there for a reason I guess it connects people! Um, how protected did you feel, physically in terms of PPE, and also sort of emotionally with support.

GP6: Yeah, so, at the beginning I don't feel we were particularly protected. So it took a while for our PPE to come through, and then when we did get it, you know to be honest- to be frank, having, you know, a three-ply surgical mask, and apron and gloves, and a visor… didn't make me feel particularly protected? Um, but we've been using that for, you know, the past year now, and we've been. And, to be honest, 'cause I have a hospital role as well, and when I compare what people are using on the generic green wards, versus what they're having to use on the Covid wards, actually it's fine. And I work on an amber unit as well, and I use the same PPE in hospital as I do in general practice, and if anything, I feel more protected in general practice, because I'm in control of what I'm wearing.

Interviewer: Oh right, what's the amber unit?

GP6: The amber unit is… so, they have like red, green and amber units in different hospitals, and then Covid wards are known as blue? So ambers are where patients could possibly have Covid, or are pending a Covid test result. So if they're green, so not

Covid, they've had a swab and it's confirmed, we don't tend to bring those patients over, in case of cross-contamination with someone who may be Covid positive, um, so it's like an assessment unit really, and 'cause I have an interest in frailty, so we- it's like, um, an ambulatory assessment unit for elderly care patients who can try and get home that day.

Interviewer: Alright, great thank you. So, how did you feel making decisions during the pandemic, with the information that you had, which is obviously changing quite often, how did you feel in the role of the decision maker for your patients?

GP6: So, um… this was tough, because we'd gone from seeing people face-to-face, examining them, and having obs, to having to listen to them really carefully and having to make an assessment based on how they sound on the phone, and people aren't very good at telling you a history. If they're in front of you, you can at least take their non-verbal cues into you, and you can sort of making an assessment based on what you see in front of you. On the phone, they will say things like, oh I've had a cough for a while, and I really hate when people say that, because a cough could mean five years or one day. So you really need to stop people, and actually take quite a, you know, strong- you have to be quite close-questioned on the phone, when people are going off on a tangent. And then they'll start talking about lots of other problems, in the same thread. So it was tough, it was really hard. So decision making was initially difficult, but now having a year's experience of doing remote consulting, actually I'm- I've got into a pattern of how I want to do things? I will happily let them talk for 30 seconds, and then stop them, and ask my questions, and then I'll be able to make a decision going forward.

Interviewer: Ok, great. Um, did you get any guidance in terms of how to, sort of, risk stratify on these phone calls?

GP6: Um, not really! *(Laughs*). It was- it was very, much here you go! Just, you're gonna have to do it on the phone now! And that was tough. We did initially have, um, I think there was this risk score going around for some time, where you can judge how good someone's breathing is… I can't remember what it was called… oh! The ROTH score. I don't know if you've come across that yet?

Interviewer: Yeah.

GP6: So we used it probably for a week.

Interviewer: Yeah, that's what I've heard.

Both laugh.

GP6: Ok, and then we thought no.

Interviewer: Yeah, nobody's said yet that it continued to be used after the first week. Um… fair enough, so, in what ways has your role changed in the pandemic. Have you had to take on any new responsibilities from secondary care? Um… vaccinations, any sort of change.

GP6: So I feel like, we have to do so much more, in terms of risk management, that's the first thing. Patients don't know where to go, um, because a lot of things have been on hold with secondary care, they come to us a lot, so they're like, please can you change this appointment, or, please can you tell me what to do next, because my X specialist, someone, hasn't got back to me, I don't know when my next appointment's going to be, so we're having to manage a lot of things in primary care that we probably normally would've offloaded to secondary care by now? Um, thankfully we have Advice & Guidance, so we can either email, um, through Choose and Book, which is the Advice & Guidance, um, method that we use, or in **REDACTED area name** we have **REDACTED organisation name** so we can call someone, um, and just get some advice about what we should do in primary care whilst they're waiting for their appointment? And then, if anything becomes really urgently important to be seen by a specialist, we'll get them seen. We just kind of need to, uh, ring around a little bit first.

Interviewer: Ok. Have you, it sort of sounds like you have, but have you adapted ok to this?

GP6: Yeah fine, to be honest, I became a GP because I like being quite autonomous with my decision making. I like being in a team as well, but because of the type of individual I am, at home I might be quite relaxed but at work I'm quite neurotic, so I've always found it quite difficult to delegate tasks? I'm getting better at it. But for me, being the GP and being in control actually, it-it's a good thing. So for me it was fine, it was just a bit hard at first.

Interviewer: Ok. I understand that, I guess with telephone calls you definitely would be more in control in that situation, just because without the actual physical waiting room and that pressure, you can…

GP6: Yeah, that is really nice. Because we only tell people we're going to call them in the morning, or in the afternoon, we don't specify a time. Unless someone, say is in a meeting or whatever, then they'll say, oh can you call me after 3:30 or whatever, and that's fine, we'll accommodate that.

Interviewer: Ok, so, I have a couple of questions but you've answered a lot of them.

GP6: Sorry!

Interviewer: No it's great for me! Um, would you say that this pandemic has changed your relationship with your colleagues or with your patients?

GP6: Um… I think, it's actually improved our relationship with patients. So, because- we're a small practice, so patients used to have to wait three weeks to see one of us, and we used to have to add argents every day at the end of our morning lists, or afternoon lists. Now, people get appointments on the day, or have to wait a couple of days, max? Um, and urgent queries that they send via text, they send a photo of something, they send an email in, we'll probably deal with it there and then, um, if they send it in, we've always done prescriptions on the day… So patients are pretty happy. Um, and because a lot of patients who struggled to get in now can get appointments at home whilst they're working, or at work working, or whilst they're watching the children, and they can deal with several issues at once, actually they're pretty, you know, pretty pleased with that. Um, some patients aren't happy, because they still want to see someone in front of them, but you can't please everyone. But I would probably say 90% of people are pretty happy? Um, in terms of colleagues, it was quite isolating at first, because we were just sitting in a room, alone, and we didn't have any patients, and, so, myself and the other salaried doctor, we had a meeting with the partners and we said, this isn't nice, this isn't working, um, so we- we started having a coffee time thing, at eleven o'clock every day, and actually we really enjoy it now. The older members of the team were quite reluctant to start doing it, um, but, with fresh blood around we assort of pushed it through and everyone really enjoys it. I think (*sighs*) generally some people have found it quite tough working from home, so some of our staff had to shield, so they struggled, um, and then when that happened at the start of the pandemic, I started working in reception, so I would do all my calls in reception for three months, so I can support the reception staff. Which was quite fun, because you answer the phone as a receptionist, and then hear how they feel. It was really interesting. I'd recommend it if you ever do general practice.

Interviewer: I actually have worked in GP reception before, but obviously, not on the other side of general practice! With the coffee morning, would that be a virtual or in-person thing?

GP6: In person, we have a big reception space, so we'd just make our teas and coffees, and just spread out.

Interviewer: That sounds really nice, I've had other GPs express a, a sort of similar arrangement, and I feel it's so important to have people support each other.

GP6: Definitely.

Interviewer: Uh, this is a slightly contentious question, so feel free to answer how you want, but what is your opinion of the government response to COVID-19 in terms of the public health policies and messages going out?

GP6: Um… my- my views on them is very variable, I- there are things they've done not so great, and there are things they couldn't have done better, because actually this is new, we've not had to deal with anything like this before, so you can't fault them, and it's not like me expressing a particular political view or anything, I think if nay party was in power they probably would've dealt with it in the same way, um, I think my only grievance is that a lot of the lockdowns have been released too quickly, and because of that we've had to go into another lockdown, and then people have got angry again, and I think particularly the whole eat out to help out, and Christmas mixing, I think, although I was a fan of it, and I did go out to eat and all that stuff, um, I really was careful about where I'd go (*sighs*), although some people aren't. So when you give people too much choice (*laughs*), I know that sounds terrible to say! But when you give people too much freedom of choice, to do what they want, actually people won't listen to the rules, per se, and actually they'll go and do what they want to do, and now we had a bigger lockdown, and lots more cases. Um, but I think I have that view because I work in hospital as well, so I've seen how bad it's been in hospital and how bad it's been in general practice, I don't really- if I was, if I was the PM, I don't think I could've done a better job, because it is a tough thing, so I can't be the one that points fingers at other people. I think the public health response has been fine, probably could've been better, but this is where we have to learn and grow, and find new strategies going forward, to get out of the pandemic.

Interviewer: Yeah. Have you had any patients confused about guidance, have you found yourself explaining anything?

GP5: All the time, like all the time. Like the biggest bug-bearer for me is where someone rings me up and says, doctor I have a cough, but I know it's not Covid. (*Laughs*). And I'm like, but how do you know it's Covid, when I don't know it's not Covid? It drives me wild, um, then you sort of reason with them and you're like well look, if you have any sort of cough, you have to- even if you think it's your asthma, or you think it's your phlegmy… whatever you've got. Please just go get the swab done. And the number of people that have come back with positive results has just been unbelievable. My own mother and father, my dad had like a tickle at the back of his throat, that he didn't tell me about, until my mum started feeling a bit shivery, so I thought she might have a fever, so I said, go and get the test done and she was positive. Then my dad got tested and was positive, so yeah, so this is the thing that really gets to me. People don't follow, um, they don't follow the isolation guidance very well either, you really need to say no, why are you at work, if your or whatever is isolating at home you shouldn't be at work, go home.

Interviewer: It must be frustrating that it comes down to you to explain these things as well. As a first point of contact with health care services. Yeah.

GP6: We try and filter what we can with our reception staff, but then they want to talk to a doctor. And even then they ask for your opinion, and don't want to know. (*Laughs*).

Interviewer: Have you been doing triaging for your, um, phone consultations?

GP6: So… I wouldn't say- triaging only works if you're going to- in my view, only works if you're filtering things out and then booking a load of people in for face-to-face straight after? Because triage is short and sweet, couple of minutes and then you're done. What we're doing on telephone consultations, which take as long as a face-to-face, if not a bit longer… Some are shorter, and then you'll catch up in the middle. Um, so it's not quite triage, some people are straight forwards, so if they say, I've got stomach pain and it's getting worse and whatever, then I would quickly triage that call into a face-to-face, but if it was something like, I've got a rash and, this is how it feels, and this is the photo I sent- that I'd do that as a full telephone consultation on the phone.

Interviewer: That makes sense, yeah, thank you. So this is a slightly more sensitive question, so answer how you wish, but has Covid had any impact for your personally?

GP6: Um… I think it's had an impact for all of us. For me, I found it quite isolating, um, because I'm incredibly close to my family, and they're very dependent on me being around, emotionally, physically, um, so my mum has a lot of health conditions which I used to look after her for. So one example is, she's at high risk of diabetes, so every Thursday morning I would take her to Weight Watchers. And that was our thing, we would go together, I would translate for her 'cause there's a language barrier, so it was hard but it was really quite cute, because she was losing weight and things, and then the pandemic happened. The gym's closed, weight watchers went remote, I can't do a Zoom call, I live in a different house, so it's- it's so difficult, so that sort of thing was hard. And then mum had- had to be referred on a two-week wait, so I had to break things and take her, 'cause I was the only one who could translate for her. So emotionally, in that way, that was quite tough, and then I had friends, so… one of my best friends had a baby in lockdown and I've seen her twice- she's almost a year old now, um, like- they're so close to me, she's like a niece to me rather than my friend's baby, so that was tough. My husband and I haven't seen my in-laws in over a year… um… lots of things. But you know. But we adopted a cat in that time and all sorts, so there's lots of positives as well.

Interviewer: Sure. OK, thank you, that was a very candid answer. Um, are there any sort of protective measures you take for yourself, to look after your physical and mental health?

GP6: So, I, when the gyms are open I would go swimming regularly, I found that was my mindfulness, because I'd just focus on my breathing. Then the gyms got closed and I was really upset (*laughs*). Um, I like walking, but there's only so much walking you can do, um… I'm just getting a little ugh about it. And, um I did take up cross-stitching when the first lockdown started, but I got bored of it. I'm a Gemini so I will procrastinate a lot. (*Laughs*). So yeah, it's been hard. Um, I cook to relax as well, but I've been- I think the worst thing I've done is just work more, and do a lot of vaccine clinics, take on a lot of hospital work, I do a lot of locums, and I- as I told you in my email, I've worked 20 days in a row, because I've got nothing else to do. Which isn't good, but…

Interviewer: It's one of those things, you fill up rather than having to deal with empty time.

GP6: Yeah, exactly.

Interviewer: No I understand that. Are you involved in the vaccine clinics?

GP6: Yeah, so we share it across the PCN, um, I tend to cover, um, either weekends or Fridays, because Fridays tend to be my day off, tend to be I say (*laughs*), and so um, so yesterday I was lead GP for half the day, and it was fine until a woman got up, went outside and collapsed on the road, so cue people panicking, me running outside in scrubs and just had this stethoscope and this woman on the road, and we didn't know what had happened to her 'cause no one witnessed it, um, but she was fine, and we got the paramedics involved. So it's quite- it's quite, um, I feel like primary care has become like A&E, so I think it's interesting!

Both laugh.

Interviewer: I was going to say, it sounds quite exciting. Have patients been understanding of when they get to receive their vaccine?

GP6: They've been amazing, and they've been so grateful. Um, I had a chap and he was 80, and he danced in! That was so beautiful, it was just so happy. People dressing up in suits and ties, 'cause they haven't been outside in so long. This is why I love old people, like they are the best. And they were just so happy to see people, it was such an occasion for them, um, people coming in groups like in couples to have their jabs… it's been lovely, it's been really nice.

Interviewer: That's so lovely, I used to love being on GP placement and seeing elderly patients coming in with full-on, like, waist jacket and everything.

GP6: (*Laughing*) Yeah, it's sweet.

Interviewer: Yeah, do you miss that getting to see them in person, or is it, you know?

GP6: I do, but like I said I have quite a low threshold to bring older patients in, um… they… they need an eye, you need to be able to holistically examine patients, because often there isn't much you can do medication-wise, but physically supporting them with comorbidities, or reducing their falls risks, doing some risk assessments, supporting them with social care, or rapid response, that type of thing you need an eye. But also 'cause I do my frailty role, I still see patients face-to-face, so I'm quite comfortable doing that.

Interviewer: Right, OK yeah. And I guess you've got that, if you're speaking to patients at GPs, you've still got the human contact. Makes sense! So are there any changes that you think should be carried on into the future, and if so why?

GP6: Um… I have said to my practice that we shouldn't drop the telephone consultations. Even though they can be quite impersonal to begin with. It's helped us see our patients in a more timely basis, gives me flexibility at work, so I don't feel like there's five people in the waiting room, and they're all angry when they see me, because they've been waiting for so long. Um… it means we can triage out things quickly, that- like a sick note or a letter or whatever, and then consult for people who do need to talk to a doctor, but don't actually need too much time, and then filter out those people who need a bit more time, and then we bring them in and give them 20 minutes rather than 10 or 5.

Interviewer: Yeah, great. Are there any changes which you have not appreciated as much?

GP6: Um… I really, really hate the distance. I think it's- it's hard, not you know (*sighs*) being- we're quite a close-knit patient, and not being able to do a lot of the social things has been really tough? So we do miss things like that. Um… in terms of just medically, I don't think we're doing anything actually that's bad? I- I think the changes that we had to make overnight would- should have happened a while ago, actually, but my practice is sort of in the dark ages a little bit! Because they're- the senior practice manager is due to retire, and- and she's amazing, but they're used to working in a certain way that they're comfortable with, and suddenly we've been pushed out of our comfort zone and find that things work better now.

Interviewer: Ok, yeah great. In general it sounds- yea I understand that it can be tough, not- yeah it's just a big change isn't it, a very big change in quite a short space of time. Um, how do you think, um, well- in general, what do you think we can learn from the pandemic thus far.

GP6: Ooh- um… I think we can learn that we're good at responding to immediate changes, um… we're very good at working together, in primary care we are very, very good at getting patients on our aside and supporting them, um, especially with people who are very isolated, um… there are so many things… it's just the effort that we ca all put in, and- and- and, you know, towards a positive change, rather than being negative and being like, oh we shouldn't do this, we shouldn't do that- we've- we've been quite good and just responded, and I think that's what you need. You need reaction- rather than just reaction and being angry, you just need to respond and just get on with it, and we're very good at doing that.

Interviewer: Yeah, great. Is there anything else you would like to tell me about being a GP during the pandemic?

GP6: Hmm… No I think we've probably covered a lot of the stuff, I think. Being a GP it's been an absolute, uh, pleasure, because for some patients it's been a lifeline, because they haven't had any family nearby, I have- I have patients who I ring every two weeks, just to check in on them?

Interviewer: Oh, really.

GP6: Yeah there's… there's one particular lady who I know very well. Mental health problems, long time, there is nothing you can do for her, and it took me a while to understand that. But I just call her every two or three weeks and have a chat with her, and I know that sounds like a massive waste of NHS resources, btu actually I get a lot of satisfaction from doing it, and I think she does too.

Interviewer: That's great, I forgot to ask, has the presentations of patients changed in lockdown?

GP6: Um, in terms of what comes through the door you mean?

Interviewer: Yeah.

GP6: Um, there are, it's variable, so we have had some patients who kind of say, I'm really sorry doctor, I didn't want to bug you, but… So people tend to try and self-manage a little bit more? And then they come to us? But then you get the patients who really should be coming to the GP first, and they start self-medicating with their wife's tablets or something, and that's not so good. So it's variable, some people are every good, they manage, some people leave it last-minute and they're at crisis, and that's not good. Um… so yeah, and then you've got the grey areas in the middle.

Interviewer: Sure, how do you think that could be managed, the patients who aren't coming to the practice?

GP6: So we just try and educate everyone as much as we can. I think the problem is, there's a lot of bad press in the media that GP practices are closed and people really, you know feel that. They either- they think we're busy, or they think we don't have time for them, in a negative sense, and they think our doors are closed. Our doors haven't closed throughout the pandemic, we haven't stopped seeing patients throughout, um, since last March, so for us we just educate our patients, we tell them no, no, you can come inside, please come inside. Um, anyone I speak to on the phone, I tell them please call us back, and if we need to see you, just tell us and we will see you. And just throwing that out to people, it does help.

Interviewer: Ok, great, um thank you. That's a really great answer.

Recording ends.
