# Supplementary material for: Primary health care during the COVID-19 pandemic: A qualitative exploration of the challenges and changes in practice experienced by GPs and GP trainees
Source: PLoS One. 2023 Feb 9;18(2):e0280733. doi: 10.1371/journal.pone.0280733 (PMC9910752; doi:10.1371/journal.pone.0280733)
Supplement: S1 Data — (ZIP) [file pone.0280733.s005.zip › GP7 Transcript.pdf]

GP7 Transcript

Interviewer: Could you tell me a little about general experience as a GP?

GP7: Um… yeah, I think uh at the start of the first, uh, first lockdown, um, so when this was- this was all in, uh, China, and it hadn't quite reached us, I was in a state of flux in my career, I was actually- so I'm a GP, um, but I'm also a GP with a specialist interest in the musculoskeletal service.

Interviewer: Ok.

GP7: Um… and, um, so I had two jobs if you like. And in my GP career, I had, well two different jobs, so I had a regular job, a salaried job, and I had a um, like a locum sort of, I was a locum also. So the way my career was arranged was that you… I would, um… I would, sort of, get my clinical development progress through my regular salaried job, but my regular salaried job wasn't paid very well, so I would locum to make up the money. Um, at the start of the first lockdown, the… there was a few changes to the practice. We… we had to go online, obviously everything was shut for a good few months at the start, and certainly one of the things I think happened was I, um, there was this element of wanting to help also, um, but I think the way the service was structured at the time, they'd taken all the- all the GPs and dragged them out of retirement, and caused them to see patients, um… which… um… in my opinion led to a lot of people, a lot of these GPs- because they were higher risk and we didn't really know about their risks at the time, led a lot of them to be seriously ill. Whereas us younger guys with families and children, um, we got sort of pushed out the market as it were. And so locum opportunities became quite, quite fewer, um… I think there was a time, when, uh, I know some friends- it didn't happen to me, but I know some friends who actually had to apply for employment support. And these were full time GPs- as in full-time locum GPs, and they didn't have a regular earning outside of general practice. Um… and I... myself- and I was essentially left to a two-session salary. By that time I'd actually reduced to one session a week, because I was working in the MSK service.

Interviewer: Right, OK, and did the MSK service continue?

GP7: No, they furloughed us.

Interviewer: Ah, OK.

GP7: So um… it was quite interesting actually, because um, you know, you make the most of what you've got. So it was great staying at home and, you know, spending time with the wife and, you know, just sort of chit chatting away, because she was pregnant at the time.

Interviewer: I'm guessing you're a new father then?

GP7: Yes! My little one's seven months old.

Interviewer: Congratulations!

GP7: Thank you, yeah, he's just a- just a joy. But nonetheless, at the time she was pregnant, and, you know, as a father you have the usual worries about the financial needs or the financial burdens that a new child will bring on. Although, I must be honest, I was never overtly worried because I never worry about money in general, but um, needless to say (*laughs*), if you have three jobs, so three sources of income, and you are reduced to one, it would be fair to say you'd be a little worried about it! (*Laughing*).

Interviewer: Yeah, completely. So ended up on about one day a week? It sounds-

GP7: I actually dropped down to one session a week! (*Laughs*). Uh, yes, so um…

x

Interviewer: Were you doing that from home or in the practice?

GP7: Yeah so it was all remotely done, but I was doing it- I was doing it in the practice. That's the only way I could keep my job, because everybody else was remote, and they needed somebody on the floor- on the shop floor as it were.

Interviewer: I must say that seems really tough, although you obviously seem to have a good approach to dealing with these things, but yeah it sounds tough, definitely.

GP7: Yeah, I can't deny the fact that it was tough. I mean one of the reasons why I wanted to leave my salaried job was because uh, I made a fair amount of progress, in my job, but there was some sort of… um, politics, as it were, as all work places have, uh… and I'm sure you've- you'll have probably figured out by now um, in terms of, um, in terms of um… because of your research, that the salaried GPs get dumped upon a lot, and uh, I- well- it was quite funny because I was a partner before, and I

never dumped upon my salaried when I was a partner, um, but when I became a salaried, uh, you know, there was some issues. So, nonetheless the point is I was actually on the way out, from my salaried job, um, and that's what happened, I think about two months into lockdown, or a month and a half- somewhere after a month into lockdown, I'd actually resigned from before the lockdown, so I left my salaried job, so there was a period of uh, a month or so, I think it was a month- one or two months, where I had, um, I basically had no job, just sitting at home doing nothing.

Interviewer: Was that difficult?

GP7: It was, yeah, it was, but to be honest, um… I had a great time, um, the wife was home, we'd sort of cook budget meals, and uh, you know, who knew that um… supermarket pizza was actually not so bad! (*Laughs*).

Interviewer: It's alright! As a student I can say it's alright!

GP7: I mean I'm quite, you know, my friends say I'm very resilient, but that doesn't change the fact that it was sort of quite tricky. Sorry, what was the focus for your research, was it our personal lives or was it more to do with clinical experience?

Interviewer: Both, so I'm going to ask you more about the changes you had in practice, I have questions for that, but I do ask later about how it affected you personally, so I feel like you've told me a little about that, so thank you.

GP7: I mean going forward it would be good if you, um, if you sort of tell me what sort of line of work you'd be more interested in? Because obviously the MSK service is different… I mean it is still general practice, it's just a slightly more specialist form of general practice if you like.

Interviewer: Um, yeah it's definitely your general practice experience, most of my questions are focussed on how, um, your clinical practice changed, and how your like working lifestyle changed. Um, I've had a few people tell me- because I've had a few trainees, I've had a few people tell me about their hospital rotations as well, um, that's mostly useful for the comparison to GP, rather than stand alone. Um, so-

GP7: Yeah well I can give you some information on hospital rotation as well because I have some- last year in August I actually got appointed to be a sports and exercise medicine registrar.

Interviewer: Oh great.

GP7: Yeah so I've been posted in A&E since.

Interviewer: Ok, so what are you doing now then?

GP7: Um, so now, as in the last six months I think it is? Three months? Since August last year? So it's actually coming up to a year now, eight- seven, eight months. I've been a, um, I've been a GP, um… I've been a sports and exercise registrar three days a week, and- and a locum for the remaining two.

Interviewer: Ok. The GP that you were salaried at for the majority of the pandemic, can you tell me about the demographic of the population you were serving?

GP7: Yeah sure so I think there were ten thousand or 11 thousand patients, it was quite a novel practice, very technologically advanced practice, we had started to change our service model to accommodate physicians associates, and they- I was quite instrumental in that change, and we made it- we made it such that the workload for the GPs was spread out across the team, and it was quite a nice model actually to work from. And we were already, in a sense, as a practice we were already prepared to deal with the changes that Covid-19 brought, and that was because we were already quite telephone-heavy and internet-heavy from the start. So we had for instance, just to give you an example, we had uh, a sort of- so the demographic you asked for, it's a very ethnically diverse mix, I think there's a fair bit of deprivation. So this is a **REDACTED area name** practice, that we're talking about, and there's some areas in **REDACTED area name** which basically don't feel like **REDACTED city name**, they don't feel like you're in the UK, they feel like you're in some foreign country, um, and this is one of those places. Lovely people, lovely practice, overall it was a good place to work in, uh, in terms of the government, in terms of the area. There a fair bit of mental health, um… and actually, the practice was poorly performing in its indicators, so I took on the role as the mental health lead, and I took on the role of developing um… as service that would sort of help, sort of, support these patients with mental health, and we actually discovered quite a few problems, um, as we went along, so- so yeah that's the general sort of demographic of the practice.

Interviewer: Could you tell me, um- so you said you felt fairly prepared for the pandemic as a GP in terms of technology, um, did you feel prepared in terms of support, so um emotional support from your colleagues, and also physical support, did you have any PPE available?

GP7: No. So in terms of emotional and physical support, um, I think speaking as a practice, um, we- we didn't have PPE at the start, in fact we were shut at the start. We were shut and we were running an entirely, sort of telephone and virtual service, sort of photographs and emails and stuff like that. For quite some time. Eventually we got some PPE coming through, and some of us started using them, but I was sort of put, um, to man the phones, um, so I was the triaging GP, so I spent a lot of my time, uh, in the first lockdown, when we actually opened the doors, I spent most of my time remote consulting, um, there would've been a period of, I think, two months where I didn't see a single patient.

Interviewer: And how did you find that.

GP7: (*Sighs*) It was strange, I think. I'm quite used to working in out-of-hours and I've been a locum up and down the country, so I didn't find it too strange to adapt to the telephone model, and I wasn't- I mean I don't have the normal anxieties that some of my colleagues do, in terms of not being able to bring the patients down, um, I'm quite sort of decisive if you like- in terms of making an action plan, but, um… it was a bit strange, um… in terms of, um…. not being able to… I mean, I think I was worried about de-skilling, more than anything else, I was worried about losing my clinical skills, I was worried about losing my examination- and deep down I knew we could sustain this for let's say, a couple of months, but you couldn't sustain this, sort of, ad infinitum. So I was worried about what might happen.

Interviewer: And have you found it to be alright in the end?

GP7: I think so, I think um as I changed… as the practice opened up now, before the pandemic we had worked a system where the number of staff that actually had to see patients was very, very small anyway. So we were running, to give you an example, we were running three sessions, uh, three GP sessions a day, with a practice size of ten thousand plus, which is quite low if you think about it, you know, just to have in GP, and then one in triage, um, so with one in triage and three actually seeing- three sessions actually seeing patients, it's actually not a lot. And often we would have appointment, uh, slots un-booked. And this was before the pandemic. So we were having… we were in the situation where we were coping quite well to begin with, and then changing from pre-pandemic to post-pandemic essentially was a case of wearing PPE and being aware of what the service was like.

Interviewer: And at that point did you have sufficient PPE to do that?

GP7: Yes at that point I think there was sufficient PPE there was just no clarity on how to use it, so I yeah, I basically just bought a mask off Amazon, I got an FFP3 mask for myself because, as everybody I think I did panic a little bit- actually I wouldn't say I

panicked it was my wife who was more worried to be fair, um, she was worried about the unborn baby and everything. Which is a reasonable concern I think.

Interviewer: Yeah, especially with the limited information at the time.

GP7: Exactly, you know, I think guys being guys, I think I was just a bit blasé about it all which is probably not a good idea! But nonetheless I did, I did wear the masks that I had. They were providing us with PPE but it was a bit patchy. I actually remember giving out my masks that I had bought, because there was- I don't know if you remember but there was a period where there was a big mask scare? So there were no masks everywhere, everyone was just buying FFP3 masks, and I managed to lay my hands on something. And I remember my nurse she just looked at me with virtually teary eyes and, you know, and she just, she looked like a puppy basically, so I just ended up giving my mask to her, thinking, you know what, just have it. Uh, and then I got my mask- I actually ordered a couple different types, so I gave her sort of my spare mask, um, so…

Interviewer: That's very lovely.

GP7: (*Laughs*). Well yes it is, but I think that was a problem that there was patchy provision of PPE and there was no clear guidance and there was certainly no conviction about the use of the PPE at the time. I mean what the government was providing, if you think about it, was a pinny and gloves.

Interviewer: Was it.

GP7: Yes, that's what it was, it was a pinny with gloves on. Now, I don't know if you know about pinnies and gloves, but we use pinnies and gloves in hospitals for MRSA patients, right, when you enter MRSA rooms, and over there we know they don't anything other than for contact, so if you happen to, you know, if their leg happens to touch or something like that, then it would reduce- supposedly would reduce the transmission. It's not proven, you know? So what you really needed was protective headgear.

Interviewer: Sure.

GP7: Um, and sort of relatively less for the rest of the body, um but what we got was a pair of gloves, mask, um…

Interviewer: It's interesting from interviews, because I've been interviewing GPs from all over the country, and some have had a similar experience- no PPE or the bare minimum, some seem to have had proper PPE for months. So it's just interesting to hear about who got what really.

GP7: Most of the NHS is a postcode lottery unfortunately.

Interviewer: Yeah. Well this is showing me that definitely. I wanted to ask about- you spoke about guidance you had regarding PPE. Did you have any guidance regarding the rest of general practice and the changes you were going through?

GP7: The rest of general practice changes? Nothing at all. I think we were just making it up as we went along.

Interviewer: How did you feel making decisions then, with the limited guidance you had, in terms of sort of advising your patients?

GP7: Um, I was quite OK with it personally, but I know my colleagues struggled. I mean, you've got to understand, I trained in India, and where I trained- my medical training was in India, so when I came- by the time I was a fifth year medical student I was intubating patients in A&E, we were managing sort of traumatic amputations, MIs, poisoning, organophosphorus poisoning… all sorts of weird and wonderful stuff. My first patient, in my first year, was a 22 year old with 99% burns. That was the first patient that I clerked in- quote unquote clerked in. I didn't clerk her in obviously, I assisted the surgeon doing the sub venous cutdown. And then I watched her basically rot to her death for the next three days. Um, so when you've sort of done that, you tend to develop a lot of confidence. I don't know about competence, but you certainly develop a lot of confidence! (*Laughing*). Uh, so I was quite OK dealing with difficult situations, I mean I- I actually went to Africa last year to do some charity medical work, and I could see my colleagues- there were some Canadian doctors there- they were panicking quite a lot, um, they were worried about you know, the standards, the quality, and I Just got on with it- I was quite relaxed, I was able to adjust and was quite comfortable with it.

Interviewer: You sound quite resilient and adaptable which is probably great qualities to have during a pandemic.

GP7: Yeah so I didn't actually feel an awful lot of strength, uh, personally, um but I- as I went along- I think it was good because a lot of my patients, I spent a lot of time, uh, whether this was in general practice, as in our practice, or out-of-hours because I did manage to do some work with the 111 service at the time.

Interviewer: Did you.

GP7: Yes, eventually I think some work did come along, um, though there was few and far in between, and they ended up putting me in home visits which was great.

Interviewer: How was that, doing home visits?

GP7: Um, it was a bit scary, at the start, but ultimately I think, um, you know, I knew that I was- because I was relatively young, my risks were perhaps lower than some that were older than me, although I am technically in the at-risk BAME category, but I knew that the main issue was perhaps not exposure, but the viral load, the size of the inoculum. So in agricultural medicine they have something called D50, or something like that, and it's the dose at which 50% of your livestock is gonna die. So the dose of the virus at which- you know, it may be a virus or a bacteria, but essentially the dose of- the number of viral articles you need to kill 50% of your livestock, so there is this concept that already exists in veterinary medicine, so I- I knew at some level that it's not just the Covid, it's the strength of-the number of viral particles you basically get, so I knew if I protect myself even if I get Covid, I'll probably be OK. And actually demonstrated quite well when I did get Covid actually, working in a Covid ward.

Interviewer: Did you!

GP7: Yeah I got Covid last year… Yeah I got Covid in December 2020, um, it was just after Christmas, I'd offered to work in the- what's it called, one of the Covid rehabilitation centres in the community hospital? You know about community hospitals right?

Interviewer: Yeah.

GP7: So these community hospitals they had a Covid ward. And actually they didn't tell me that they had a Covid ward, uh… if they had I probably would've declined the shift, because I wasn't mentally prepared, and, you know, for the wife. And actually the wife still doesn't know that the ward I've been to was a Covid ward! (*Laughs*).

Interviewer: I'll keep this anonymous!

GP7: Thank you, you have the secret to destroy my marriage now (*laughs*), I'm just joking, but the point is I wasn't aware that there was a Covid ward, and I-

Wifi error.

Interviewer: So you had Covid, how was your recovery from that?

GP7: Oh yes, so I was asked- I was asked to go to a- a community hospital that had a Covid recovery ward and wasn't informed about it, and this was in the second wave, so the second lockdown, um, I obviously, you know, isolated and made sure that the Wifi had the lowest possible viral load was hard because isolating is, you know, hard in the best of times, and it's harder still if it's just you and your wife with very little family support, or other people to help you out, and even, you know, and it's hard for her as well because she's only been in the UK a year and a bit? And she's not really gone out that much. So she feels a little overwhelmed sometimes. I mean she's great, but she just needs that little bit of support, it takes you a few years to get used to anew country doesn't it.

Interviewer: Yeah, of course I understand that.

GP7: Yeah so she was, sort of, in the other room with a little baby trying to, sort of, manage everything, and uh, I was very strict on the isolation and I was very happy I did that because for ten days, um, I… um… so when I- so when my symptoms improved and I came out of isolation, um she didn't actually have any symptoms. It was only when I went back to work, I think two or three days after my isolation was over, uh, that she got some Covid symptoms, but interestingly enough her Covid symptoms were a lot less than my symptoms, and I think some of it is down to the fact that we reduced the viral load. So I think in terms of official guidance, I don't think there was much at the start, or even to this day there's a fair bit of confusion. I mean it's starting to come out now, which is great, but it wasn't there at the start, and essentially every clinician was left to fend for themselves, um, and you were looking for advice. And people went to the weirdest- we were one step away from selling snake oil. It's a figurative speech- not real snake oil- there were some people who were suggesting – I spoke to one person who said oh, you can get so and so vitamin combination, um…

Interviewer: I guess it is just healthcare professionals just trying to cover a lack of knowledge.

GP7: You know, I mean I think the problem is, uh people are – and I definitely experienced that in my colleagues, people are very uncomfortable with informed inaction, um you know, because we know we must do something, that's not necessarily the case, um, you know, and we just learn to learn that sometimes the right things to do is to wait. It's like, you know, waiting in a lobby for your flight to come, there's no more real action we can take, the flight will come when it wants to come.

Interviewer: That's a nice metaphor for the situation. Would you say that it changed your relationship with your colleagues or with your patients?

GP7: My patients were definitely a lot more anxious, and I also became something of an emotional firefighter, and it wasn't a pleasant… well it made the task harder, um, because when the, um, when you're reducing the physical appointments, the burden or remote appointments increases. And you requirement for each staff to work is more with each patient requiring more time- that becomes a harder job to achieve, and that actually burdened us as a practice more than we had expected to, you know because people were taking longer all the time, and it did cause us some- some challenges in the workplace, it did cause, you know, some concerns in the workplace, and people were quite stressed at one point, you know, I heard some people- I was called in on once by a senior partner. So it did get a bit rough, and a bit unseemly.

Interviewer: Yeah. How did you- did you experience delayed waiting times and referrals for your patients?

GP7: Yes, um, I think two week wait referrals were doing OK, um… but the routine referral system was completely shut, um, I think I waited for, um, a couple of my referrals I basically- before I made the referral I knew what was going to happen, so I just warned my patient it was going to take absolutely ages, and then we talked about how we could manage it in the context of home. It kind of became a little bit like relief medicine, like you're working in Africa or something um, you know, so I'm glad I had those skills as I was able to transfer them here, therefore making realistic action plans and managing my patients' expectations, without necessarily trying to overburden the service. In fact a couple of my patients, I mentioned my concerns back to them, and said look I could refer you now, but would you rather be in the system and wait, or what would you like to do, because it's going to take time, and here's what we can do in the meantime! And they were actually happy not to be referred. I mean I sent the referral anyway, because I knew that the queue in the system would be much longer by the time they came back needing help, so uh, so yeah, I mean we were sort of burdened with issues that we wouldn't normally have to deal with.

Interviewer: Yeah it sounds like you were taking on new roles, as a GP.

GP7: Yeah, to be fair though, that is ultimately the grand plan for general practice anyway, um, if you look at GPs in America for instance, not that I'm a big fan of the American system, but if you look at their GPs, their family physicians, they definitely do a lot more than we do in terms of the interventions and the number of treatments they treat, so I don't see that to a bad thing. I'm actually quite excited to be taking on more responsibilities from the hospitals, because half the things we do in general practice, we don't have to do to be honest. And that's my personal opinion, I know there's quite a few colleagues who don't feel like this and are therefore nervous about managing greater complexity-more challenging situations.

Interviewer: You're describing things that I've had described in other interviews. I've had people speak about secondary care changing, as a result of this sort of shift in responsibilities. Have you- sort of- have you been using advice from them, or has it been the other routes you've been talking about?

GP7: No actually, when Covid hit, and when I started seeing patients, I actually reduced my communication with secondary care myself, and I started managing a greater amount of risk because that's what I felt was necessary. yes, a higher amount of risk means there's a higher chance of, um, litigation, higher chance of complication, uh, so on and so forth, but then ultimately desperate times call for desperate measures, and I genuinely thought that this was the right thing to do. Uh, I haven't actually mentioned this to anyone, because I felt that if I did my colleagues would take me to be, um, an inappropriate risk taker, and you know these things do matter, and ultimately you don't want to be the black sheep of the community you come from, but in terms of my communication with secondary care, I actually didn't have- I'm just trying to think, across my jobs, um…. I did not, I don't think I ever rung anyone, until my A&E job, uh, I didn't actually ring anyone for advice. I knew if I had to send somebody in I'd send them in, and that would be it, obviously I'd give them my clinical advice… but I was making quite, um, clear decision plans. Trying to make life easy for everyone involved.

Interviewer: I mean, it's a shame that you're in a position that you couldn't say you were doing something which was probably altruistic to help the practice along, um but yeah I've heard a similar thing from GPs which is that they're talking on more roles, just not openly, which is difficult. Um, hopefully that change will become more official, and it will be easier to- like you said, like the American system.

GP7. Yeah. The changes will become more open, the problem is we will never get the support that we need to go with that. That's the big problem. Um… it's happening, and you can see it happening. What we're not getting is the financial support, but also you need the infrastructure to go with it? So on the one side you expect general practitioners to see more patients, I'll give you an example. Just yesterday I was in out-of-hours and I saw this man, I think he was 65? And he'd come in with what seemed to be a UTI, but with his UTI he had a little bit of dullness and pain in his right upper quadrant. Now this is not typical for loin to groin (*unintelligible*) colic, but something within me told me that this man is likely going to need stronger antibiotics, and this could just be the first sign, you know that before the pain localises it refers to the dermatome? So my concern was that this was actually early ascending pyelonephritis, it's just not there yet. So this I would normally treat with a broad-spectrum antibiotic, like co-amoxiclav, but I know, for instance, that I am, because I am a GP I have limitations on what kind of antibiotics I can prescribe. And if I prescribe co-amoxiclav, somebody's going to be knocking on my door asking me about the reasons for my decision-making. And quite honestly, it's just not a good use of my time. It's a bit annoying- it's one of the reasons I actually

went into specialty care because they ask fewer questions there, um, and you know, I thought you had more freedom in a GP, but clearly you don't. So I ended up prescribing him something and (*unintelligible*) really, really hard, and (*unintelligible)* when I knew I could prescribe him something better. It's quite frustrating, when you think about it.

Interviewer: Yeah, that sounds very frustrating. Um, but a really good anecdote to demonstrate what you're talking about thank you. But yeah very frustrating. Um, it's a slightly contentious question so answer it how you want, but what is your opinion of the government response to Covid-19 in terms of public health policies, and management of the pandemic?

GP7: That's a good question, I think, um, I mean a lot of my colleagues have described the government actions as shambolic, or you know inappropriate or, you know, not good enough. I actually take- I'm not a big fan of Boris Johnson, I do think he is, um… I'm not a big fan of the Conservative government personally, I have… I have, you know I think they are- I think they'll be the first people to marginalise ethnic minorities- communities, and I'm certainly not- you know, my wife wears, you know, a face veil, so technically a letterbox as Boris Johnson so believes, so I have my problems with the Conservative government. But I think considering the circumstances and the situation I have to be fair. They tried, is the best way I can put it. The problem is not how the government handled the pandemic, the problem was what the government actually did before the pandemic came about, because there'd been a warning about a global pandemic for I think ten years, or longer? There's been warning of that for quite some time. I remember I did a presentation about a good few years ago, I saw it was there, you know, amongst the top ten global threats, um, and I think that government has not taken the advice seriously enough, so it wasn't well prepared to handle the circumstances, and the results are what we're seeing- the results of the institutional structure of the government, so if they were not good enough, id the response is shambolic, it's not because they were bad people- considering the pandemic response, a lot, you know, you saw the good nature in adversity of people, and Boris Johnson and his government is no exception, I think the government tried really hard, it was just the nature of the circumstances that meant they couldn't respond. They had to make decision calls, and for some reason we look up to Sweden like it's the epitome of civilisation, um, so we tried to follow herd immunity which clearly wasn't a good idea, and we know this from the figure in Sweden, which basically- they tried the herd immunity, um, and as my then-landlord put it, he put it best- herd immunity is whoever survives after not being dead from the infection. Um, you know, and it's a shame that we looked up to these ideas. But again you can understand, I actually spoke to a GP at the time and she had- or her husband had rather, a private business of some sort, and she had a private practice also which was affected severely, and she was arguing against lockdowns. This was in the first lockdown. And this is a GP, and I was calling her- you know it was hilarious, I was surprised by the response and the way that she was looking at things. And I think that, you know, considering they are a conservative government, you know, they would've been anti-lockdown and so on and so forth, they've still done a fair bit- they've responded to the public opinion over their own and they

haven't' imposed their opinion. They've done things which are good, and you know, they're firefighting ultimately. Um, so I wouldn't expect- the response was shambolic but I wouldn't expect anything more.

Interviewer: OK, thank you for your answer. In general, are there any changes that you've seen which you think should be carried on into the future? And equally, are there any changes which you have found less effective in general practice?

GP7: I need to think about that one. Um… so I think- I think remote working and telemedicine, quote unquote, um, it's here to stay. Um- I can't say it's the best way of treating, but it's certainly- it needed to be- it needed to become part of clinical medicine, especially general practice, for a long, long time, and it's happened now so that's great. I do believe also- so that's one thing that I'd like to see going on. I do believe also that there is an element of, uh, education that can be provided virtually, and- and that should- and I'm sure that you hate all your virtual lectures- um, and that is absolutely correct, but there are some things, and some circumstances, where it is actually better to do things virtually. And- and I think this experimentation with technology, which Covid-19 brought along, um, ultimately is a good thing, because we have to move with the times, you know, there are efficient ways of doing things which are convenient, but it has to be an integrated systems approach, which I don't think you've done as well with, I mean, you know, communication channels… I mean some great partnerships came about, um, you know, some academic researchers started to turn to us, the population, almost as if- almost like using citizen's scientists, you know, same approach, and they, you know they started to- so some unusual collaborations came about which was good, the problem is we are unable to contextualise that, and actually make it a consistent thing, and I believe that the lack of a systems approach, the lack of a mutually incorporative approach, is probably the one thing that should be developed further.

Interviewer: Right, OK thank you. By collaborations, who are you speaking between?

GP7: So, um, well in my idea- this is probably utopian- but in my ideal mind, um, what you want is, um, what you want is um, a clear passage, a clear pathway, between academia and clinical medicine. So academia for that you need to learn how to communicate their results, or their findings, uh they're terrible at that, and that's the reason why there's a communication rift. Um, and then, it's the- it's a clear pathway between clinicians and academics with the general population. One of the reasons why we've had so many um… um…. uh… myths and weird and wonderful ideas about Covid, you know, about this being a government conspiracy, and (*unintelligible*) networks, and, if you just slice a lemon it will be fine, so on and so forth, and that the vaccine is worse than the virus, is because we haven't been able to communicate the message, uh you know we still haven't communicated- both academics and clinicians. I mean it's starting to come around now, which is good. That connected with the government, to provide the overarching infrastructure to support the finances, the relevant finances, you know- they were- they were able to get the vaccines by running several arms in parallel, the question I ask is why can't we do that for anything, and

why did we not think of that before? And it's not like we didn't think of these things before, we knew these systems could be done, but were too complacent perhaps. Um, you know, I mean back in the day I remember when I was an orthopaedic SHO, I tried to float a research study- something to do with blood on the masks in theatre, we found that actually there was blood behind the masks? Uh, in orthopaedic theatre? And even the radiographers, the furthest person away from the patient, had blood on their masks. So I tried to get this study approved by ethics back in the day, and it took me absolutely bloody months until I gave up on it. And now if I have a Covid project, everybody is rushing to give me approval, um, but if it's a non-Covid project, we're back to where we were. So… I just find that, you know, we've sort of learnt about integration. There's something about disaster response that brings the best in us, and then we forget about it. So I think that if we were able to translate those findings in the systems-wide approach, where all organisations were working for the mutual benefit, I suppose we'd have to define what mutual benefit is, but for the benefit of the user- of the end-user, then that is what I'd like to see going forwards.

Interviewer: Great thank you for such a- just such a great answer. Thank you very much. I'm aware that we're reaching the end of our time, is there anything that we haven't covered that you think is important to you, in your experience of general practice?

GP7: There was something and then I forgot! (*Laughs*). I just can't remember it now. But uh… let me see if I can remember it. Um I was going to say something earlier on, it's completely slipped my mind. Uh but yeah, I think, one of the things that you, um… which we covered the conspiracy theories as well, um… yes and I- and I spoke about the rift between- I mean there's doctors out there nowadays who are essentially going against the government rhetoric, you know, um, and it's actually quite scary, um, and- are you studying the effects of Covid-19- the changes on GPs?

Interviewer: Yeah.

GP7: So in terms of clinical practice, um… we mentioned… one of the things I forgot to mention was I don't know if I have mentioned this, it was about the- the effect of the, um, of the service on allied healthcare staff? So I don't know if you know but when the- when the first dose of Pfizer vaccines came along, in Oxford, they… they sent out an email request, um- I missed it- they sent out an email and it was basically a link that you could click and book. And there was no system of verification. So that link got overburdened in two days, or something like that, well not two days- and that's because staff had started to write to family and friends to book in to get the vaccine.

Interviewer: Really.

GP7: Yeah… yeah. And you know, as much as you can argue about the ethics of this thing, and you know I don't think staff did the right thing, because then I didn't get the vaccine and I got Covid, um… but, sort of, besides that point, what I learnt was that there was a significant amount of anxiety amongst the staff, um, and there was a lot of, sort of… fake reassurance I say? You know, superficial reassurance, when no one knew what was happening? But very little, um… um… very little- very little substantial… and I'm not- this is not a complaint, this is more sort of an observation about what happened, and I think this panic, um… certainly contributed to some of the panics we've had, uh, amongst sort of the patient and the local community, because you know, it's well-known that doctors- that's why we double blind patients- studies- isn't it, because if the doctors have confidence in their treatments, then the patients have more confidence also, and if doctors are worried… And it extends to nurses and health care assistants and so on, so forth, uh the problem is we as doctors- and you will learn this, you know, we get the humanity beaten out of you, and you learn to sort of put on a brave face, and I know I'm exaggerating but you get my point- you learn to put on a brave face as soon as possible really, but I don't think the extended healthcare professions have that quality just yet, and I do think that that- that definitely has an impact on, um, on your personal health, because everyone's panicking and one person's setting off the other, and also the… uh… effect that it has on the patients and the patients they consult on the phone, you know, um so we had a couple patients where they'd spoken to a nurse and actually they'd started to become more panicky and then come to the GP and then we then had to reassure them. So patients were being escalated and you could say it was all a mess, but I think ultimately the mess was just anxiety. You know, fear of the unknown. Also the- I do believe the BAME community staff were marginalised, despite some of the evidence that was presented. I've had ITU nurses come up to me, um, you know, Asian or otherwise- Arab- African, who were told quite categorically that they had to be front-line staff, doing high-risk procedures which, um, you know they felt- they were worried about really, and quite rightly so. And that's not to say that they should be put in bubble wrap and protected, it's simply having a realistic set of safeguards, and making sure that, you know, the safety of the staff… You know it's- somebody put it really nicely on twitter, somebody said it really nicely, that, you know, if you are a, uh, builder, then you know, then you have a lifetime risk of falling off a ladder, but you know you shouldn't expect to fall of a ladder at some point in your life because there's relative safety procedures and checks. But for some reason NHS staff just expected to get Covid.

Interviewer: That's a brilliant analogy. Yeah.

GP7: I mean I got Covid- I said, you know, it's gonna happen sooner or later, it was just going to happen. And when I stop and think about it- I didn't think about it until that message- but when I stop and think about it, I realised I am… it's fine that, you know, there's altruism, and we're in this 'til our graves, but it shouldn't be misused by the powers that be. You know, every general practice, uh… every time you examine someone's throat, it's actually an aerosol-generating procedure, you know, and yet um… ITU patients and secondary care patients, got level three PPE, and general practice didn't, and worse than that they

didn't even get an apology. You know, if a CCG was to say simply, look guys we can't afford this sorry, you know, and did something else…

Interviewer: Some recognition?

GP7: Exactly, some recognition. And simply an awareness of their limitations as an organisation. You know, it's not- it's simply an awareness, it's what you would do if your- I don't know if you have a boyfriend or whatever- but it's what you would do with your wife, or your brother, or your family, you know- I'm sorry I can't do this, I recognise it, this is- sort of, almost beyond my control, I cannot afford to buy you this toy, or whatever it is you want.

Interviewer: That's entirely reasonable, I'm sure people were asking for it.

GP7: Exactly, and I think that would've created a significant effect, uh, because what happened is I've spoken to staff, and you know, you might be surprised but I've spoken to allied healthcare staff, who are manning the 111 lines, and who are nurses and health care professionals, even a couple of GPs, who believe in conspiracy theories? Genuinely believe in that stuff. It's one thing to flirt with it- and I'm sure everybody's flirting with some idea, you know, some people don't believe the moon landing happened, and I'm not one to- and flat-earthers- and I'm not one to judge people's perspective, I'm only interested in the effect it will have on their practice. And I think that if staff were given clear logical explanations, including the limitations of our knowledge, uh, then perhaps some of this conspiracy business- which by the way was significantly- was responsible for reduced vaccine uptake, and was certainly responsible for the increase of Covid-19, uh in some populations, could've been avoided.

Interviewer: Conspiracies do arise to fill gaps in knowledge, things that we can't explain, and this case it was about where the explanations were, um, yeah it's a great point that even healthcare professionals themselves were vulnerable to conspiracies. A worrying point, yeah.

GP7: It is, and it just tells us about- about what- what- what's in hold for the future. This is completely by-the-by but you might find it interesting, then I'll probably end on this. Do you know this woman called the human Barbie?

Interviewer: Yeah, uh, the woman who's had all the plastic surgery?

GP7: Yeah, I read an article on her, and this was a sort of auto-biographical… like an interview really, she spoke about herself and it was all, sort of, written up, and this person actually believes that she is an alien from out of space, who's been sent down.

And I remember there's a few human barbies, she's produced some spin offs- yeah but there is one, there's an original one and I've forgotten her name, she's Russian in origin though, and uh, she… she believes that she's an external alien that's come from an extra-terrestrial planet somewhere, with the sole purpose of, uh, elevating mankind to a higher level of existence? Whatever that means. And she is currently an alternative practitioner who does some weird spiritual practice, I think it's called, uh… soul migration? Or trans migration? Something like that. So she does that, she actually has like classes and lectures for this. And the thing that I find interesting is that she went to a psychiatrist to get assessed, which I thought was pretty self-aware, and the psychiatrist confirmed that she was an extra-terrestrial sent to spread the issue. Now this is obviously an extreme case… I think this is an extreme case, I'm not sort of saying by any means that psychiatrists or indeed doctors are like that, or nurses, the point is that it just makes a really good point that the amount of incoherent- it's assumed that because we're educated in traditional Western medicine that we are coherent and a coordinated bunch, uh, that's how the public see us anyway, um, it's not necessarily true and I believe that something needs to be done to address our own personal understanding and expressions… and I do think it's played a part in how we've dealt with the conspiracy, uh, as clinicians and as allied healthcare staff. Scary isn't it?

Interviewer: A little bit scary, yeah. I know I've gone over our time so thank you very much for today.

Recording ends
