# Supplementary material for: Primary health care during the COVID-19 pandemic: A qualitative exploration of the challenges and changes in practice experienced by GPs and GP trainees
Source: PLoS One. 2023 Feb 9;18(2):e0280733. doi: 10.1371/journal.pone.0280733 (PMC9910752; doi:10.1371/journal.pone.0280733)
Supplement: S1 Data — (ZIP) [file pone.0280733.s005.zip › GP8 Transcript.pdf]

GP8 Transcript

Interviewer: So to start could you tell me a little bit about your general experience in general practice?

GP8: Um, yeah I can start- well I can start from the start when it first obviously hit, when Covid-

Interviewer: Yeah, um, and pre-pandemic, sort of what you've done in general practice?

GP8: Okay yeah sure, so I can- I can talk about my practice actually some of my old practice, so we were a two-partner practice, uh, 8000 patients across two practices, 98% face to face, 1%, um... actually 2% telephone, 98% face to face, um, very much a typical GP pre-pandemic general practice, which was a lot of patients walking in, people making, we'd- our patients make appointments online, we did no online, with no- we had no video conferencing facilities, we had no, uh- we barely did telephone calls, it felt like nearly everything was in a face to face basis and that went for everybody, GPS, our nurses, our clinical pharmacists, basically everything was done on a face to face basis. We had no members of staff working from home ever, we didn't even allow people to work from home at any stage, it was, no one ever asked the question- I think if we asked the question we may have been sympathetic to it, but no one ever asked beforehand, to um, to do that, so um, obviously when Covid hit, the um... I remember text message coming because I was preparing for this interview to remind myself, and um, we had a- a text come from **REDACTED name and role** for NHS England, uh put a Whatsapp message out saying 'close your doors tomorrow morning' and... I'm obviously a younger GP, my previous partner was a- quite an old-school, 30 years in the practice, 60 year old GP, and, um, at that initial point because obviously we have financial year end, and things like QOF and stuff, the- the, when we got to hit our targets by the end of March.

Interviewer: Right.

GP8: And so there was a conflict between me and **REDACTED colleague role** straight away. Because the fact that he wanted to keep the doors open. He wanted to have nurses to do all those chronic disease checks, those checks with blood pressures and the blood tests for all the diabetics etc, and the COPD checks and the asthma checks, and there's me on the other hand, who saying 'I've got this Whatsapp, I've got this message from a really- really high-up person basically saying shut your doors, we're gonna let you shut your doors, don't open your doors' and I clearly- I distinctly remember on the very first day I was at our branch site which has 1000 patients, and I told- I called our receptionist the night before, I said, do not open the doors at all, lock them. Um, I got to work and I got to a branch site, and I got called by **REDACTED colleague role**, saying **REDACTED name of GP8**, **REDACTED name of colleague** is trying to open the door, he wants the door open and I kind of said, no, lock them and he said 'he won't listen to me, can you do something'. Great. I said I don't want to call him because he never picks up his phone,

so I said take your phone and put it on loudspeaker, and I literally just remember saying **REDACTED name of colleague** keep the doors locked do not open these doors, don't do it, you're risking our patients and our staff, don't do it' and at that point he relented because I think he realised how- Am I allowed to swear in this interview, by the way (*laughs*). I was so pissed off. You know what I mean, I was so pissed off, and I'm not a very angry man, but that was closest I got to getting really pissed off with 'cause at that stage, because he was so insistent on that- on keeping the doors open, luckily I managed to convince him to keep the doors shut, and we kept the doors locked from then until what, they're still shut really, you know that- we let a select few patients in, but those have been essentially locked since that start point, so in terms of how the pandemic changed things, it was at that point where we went all to telephone calls. Um, we were really lucky, and I'm sure you've probably heard about AccuRx, which has become like the gold center every GP in the country, uh, who obviously offer their services for free, but the majority of practices didn't even have, um, webcams, so our computers didn't have webcams so we ended up having to- I ended up having to scurry around on a- on Amazon on a weekend, looking around for any webcams, which are not ridiculously priced 'cause we're independent people we had to pay for them ourselves, and you know buying webcams and getting enough webcams for our practice

Interviewer: So were you personally paying for that equipment?

GP8: Yeah I did it first, there was a relief thing that was eventually put out later on, but to be honest I never claimed for it, I just thought this is the right thing to do so, we just got web-cams in. I remember getting the webcams in and then unfortunately not being able to install them because of IT issues, and then having to call someone from IT to come out, so they can, um, because the access, we have to load on extra programs is zilch as GPS, as anyone, so they had to come- so we had to get someone to come out to actually put an administrator in so we could then unlock, um that, and then the stupidest thing was like, if I remember correctly, Microsoft edge didn't allow AccuRx at all, it wouldn't let it in because it said it wasn't at the same level, so then we had to get them back out to get Google Chrome, because Google chrome was only one initially that would let AccuRx in, so it was a real pain the ass, to be honest, and-

Interviewer: As the manager, were you sort of the one, sort of coordinating this whole change?

GP8: Well it was partly me, and partly my practice manager, my other partner, we were coordinating change together and there's- there's lots of things that we did at that point, we- e obviously were completely remote and we used AccuRx's functions to its maximum so we made our messages, I remember on the Friday, we have a protocol- I played- which is where we send messages out to patients, and we text every single patient- really, in retrospect, it probably should've been in block capitals because I wanted people to realize this is bloody important but it literally was just saying, if you have an appointment at the

surgery it has been cancelled. Do not come to the surgery under any circumstances whatsoever, unless we call you, please call the practice, if you want to get online access this is how to get online access, but please do not attend the surgery expect your appointment to be honoured.

Interviewer: Yeah.

GP8: I probably did put block capitals to make it sound serious, but in the end, it just sounded like I was shouting, which is probably not what I wanted to be honest, as the um, the outcome (*laughs*). But um, uh, then after that, we did lots of other stuff so we put lots of other text messages together, so like, uh... rashes and stuff I mean this- it's ridiculous how many patients we used to get in to come and see us for a rash. Nowadays we just do photograph, send us a picture back and things like that and it's been very, very useful in that way, so in terms of how it's changed, we are now, well we were- nearly every practice I go to now as a locum as well, are um, it's um, GP first and if, in the event, a GP needs to see a patient we bring them in so you know, I yesterday did 35 consultations telephone-wise, I brought one person in, and the other 35 were all remotely. There's other days, where I've done 50 consultations but not bought one person into surgery. That has had its own risks. Um, where yeah, there was a risk at- very early on the pandemic. A woman called me with a lump in her axilla, you know in her armpit, for about a week, painless, we did a video call she examined it for me and it sounded all quite, um, benign and at that stage you didn't eat meet breast cancer criteria and- and I truly believe that even if I'd seen her face to face, I still wouldn't have done a cancel referral, because the- guidelines, um unfortunately later on in the pandemic she got exactly- you know, the lump persisted ended and ended up with breast cancer and I reflect back thinking, crap, if this had been face to face and I'd seen her face to face, would I have referred her, even if went against the guideline, and there's that, kind of, um, internal question that goes in my head.

Interviewer: Yeah that sounds really tough, how do you manage the responsibilities of risk stratifying over the phone, have you had any guidance for that?

GP8: Um, so there's not been any guidance for telephone calls, I was lucky that we had done some, there's a- back on- there's a previous contract, we had a couple years ago about access, and we were really as GPs trying to do it via telephone, so we had an some telephone triage training, so I've gone through that, and so I learned my risk stratification from that but for me in terms of the telephone or seeing them face to face, in terms of my risk stratification. I don't know if I really changed much, I think, I was still trying to treat them as if they were in front of me, the only problem was with this lump, I couldn't touch it, but I think, even if I touched it I would've done the risk stratification, saying, this lump has been here for two weeks, when we look at the guidelines for breast cancer in a 32 year old woman we shouldn't be referring them for a lump for at least six weeks, so it should

have settled by them, and so I would have probably done the same thing, is what makes me sad, but the biggest guidance- the biggest difficulty it gave me is that I'm very- I try to gauge what my patient response is on a face to face basis, and when you can't see them face to face and you're going off a phone call you've lost the sense there, you know, there's a sense, you get about someone's responses over a face to face consultation-

Interviewer: Yeah.

GP8: -that you're losing in the- in the digital era and stuff.

Interviewer: How have, um, how have your patients being with that change, and also how have you been with that change to telemedicine?

GP8: So I can do- for myself it's been- it's been tougher than I thought it would be, I thought I'd loved it, I thought, you know initially I thought about doing this, this is amazing, this is great, but you miss that face to face contact and I think there's, especially now that I'm working as a locum, it's very difficult to make a sustainable face- your GP relationship with a person you don't meet face-to-face. And I think there's loads- I'm um, I shouldn't admit this, but I'm a big hug- I have lots of- I love- not in a weird way, but you know I'm- I'm a hugger and so there's a few patients who used to come in and give me a hug and stuff and so stupid, but these especially-

Interviewer: No it doesn't sounds stupid, especially with the elderly patients, I can imagine.

GP8: Yeah and not even that, like all the little kids, like, I remember we used to do a cough and cold clinic, I remember there was this one child, I used to see like every other week 'cause his mum was really worried about him all the time, which is fine, and um, he came in- I came in one day- cause he- our nurses used to run it, and they used to do all the histories and I used to walk in a do the last bit, and I remember one day I walked into this room, and this little kid was next to his mom, saw me, run across the room and hugged my legs, and- and that kind of stuff you miss that kind of thing, and you miss- there's so many children that I used to see growing up and there's so many kids that I- I was there for five years, and you see kids born when you- when you started there and who you know- who I feel I know them, and I haven't seen them in over a year, and it sounds weird to say out loud, but you know I miss those bits, I really missed those, watching those kids grow up, or watching that patient with anxiety, depression, because unfortunately with the anxiety and depression patients, you don't do it by video call, you do it by phone call, and you don't get to see that um... you haven't done psych because you're doing third to forth year, but you know when you're in fourth year you do psych, and the one thing we talk about is how the patients looks, you know what are they wearing,

how do they stand like, and stuff and they're all really soft things that you're not going to get over a telephone call, at all, and those are things, I would say I miss, from- from a patient point of view, I think it's been very difficult depending on which kind of patients, you talk to, uh, one of my other roles, I work at the CCG in terms of this thing called primary care committee in common, which is basically get patient representatives in, and one of the big gripes from them was is okay great we've now got all these 'silver surfers', so these elderly patients who are becoming good with it, good with the Internet and stuff, but there's a lot who aren't, do you know what I mean, how do we A) train our patients to start being becoming silver surfers too, but at the same time, if they don't want to become silver surfers, or what if they've got dementia, or other problems, it's a real challenge to- to work in that kind of fashion really. Um, I forgot to bring up the nursing home actually, because we have about four nursing homes and the way- 'cause I used to do, one of the (*unintelligible*) I used to do was the weekly ward round, so you know, go there for an hour a week and... and go round all the patients and review anyone who was sick. When we obviously went into the Covid and the horrors of kind of April, May, um I didn't go there at all. So I went to a digital way, so we literally did um, I- I would facetime them, or I would Whatsapp call them, because they didn't have laptops so they didn't have (*laughs*) any- so we were literally having to use our mobile phones and do, uh, facetime video call, so literally you just go right let's do a whole ward round, for the whole patients, let's just do it via facetime, and me one of the nurses would just go around the nursing home on facetime, yeah.

Interviewer: Yeah. Sorry, what were you going to say?

GP8: I think these- you come back to the- the whole idea of the, how patients are taking it, I truly believe a practice is part of the Community and there's bits where patients would come in for particular reasons to ask particular questions which weren't even related to medicine, and that has stopped now and, some people say it's a good thing, but I think it's pulling us back as GPs, has not been pillars to the community and I think it's a really sad thing that we're no longer... If we carry on with this way even post-pandemic, we're losing a very valuable part we play as part of the Community, and I think that has massive risks.

Interviewer: Thank you that's a very candid answer, and you know not a- not one that I haven't heard before, and yeah it's tricky because, obviously, I guess, there's a convenience factor but obviously you lose so much from it. Are you working from home when you take these calls, or in the practice?

GP8: Um, well we would- because one of the changes we made was because obviously, the more people you came in contact with, the more, the higher the risk you had of getting Covid, so we went to a- we went just a wardless system, where basically whoever's on call did phone calls and only phone calls and whichever the GP wasn't on call would do face-to-face appointments. So we would do uh, an hour every day in the afternoon, which was just phone calls, I mean face-to-face calls, so when I was on-

call I would actually not even go to the surgery, because my argument was, is that, and this had an impact on relationships with staff because, that's another thing that always... that's really hard, I used to love hanging out with our staff, I used to spend Tuesday mornings, when I was on my ST3 days, I'd just go and sit in there for an hour with them and just have a cup of tea or coffee and a biscuit and talk to our practice manager and talk to staff and just have a joke and a laugh, and when we went into a pandemic, the Covid hit, the argument became I need to only speak to them when I have to speak to them, not because I don't care about them, but because I do care about them. I don't want them to get sick, what if I have Covid and I give it to them, or conversely, what if they've got Covid, and they give it to me? So losing that section of relationships was really, really tough.

Interviewer: How did that change your relationship with your colleagues?

GP8: Um, well it had a massive impact on my relationship with my **REDACTED colleague**, he and me sort of stopped seeing eye to eye, around- there's other reasons that were not Covid-related, but at that point, mine and his relationship fell apart completely and within... uh, by the end of March- by the end of April, we would just arguing- we were two guys who had gotten on really well, joke and laugh with each other, there's about a 20 year gap between us, we had a really great relationship. Due to other reasons, not down to Covid, um, because we weren't seeing each other face to face, because we weren't talking to each other anymore, we were just arguing, literally... full on shouting at each other, arguing with each other, and fortunately both of us left the practice at the end of November, as a result of our... because- our relationship- a GP practice works on strong leadership and having people who are working in the same direction. So, at that point we started working in complete- if anything I would say he would disagree just for the sake of disagreeing with it. And he was making decisions which were quite, for me, which felt quite selfish, but with no explanation of, uh... why he was doing certain things and, um, as a result, our relationship- and it impacted on staff and then it impacts on patient care because staff don't know... you need a strong, um, culture of what 'this is the process that we're going to follow'. But when we started diverging from that and I think two different processes, it became very difficult for staff to keep up and then staff became stuck in the middle, which withdrew me even more because I felt like, I can't speak about an issue I have with him, because if I tell them about the issue I have with him, it's unprofessional of me, so I was keeping my mouth shut, and- and withdrawing even more so, I would literally go into... so um, pre-pandemic I would be at the surgery Monday to Friday, 8 ’til 6:30 every day, so about 50, 60 hours a week, post-pandemic I'd only go into the surgery between nine and three on a Friday?

Interviewer: That must have been really tough.

GP8: It was! It was yeah, it was really tough in terms of... it's stupid, it sounds ridiculous, I can't believe I'm saying it- the pandemic wasn't affecting me as much as the relationship problem with him, because that was affecting my livelihood, that-

that potentially meant, if things had gone wrong- things are going wrong, and if they continued going wrong, you're going to get involved, the GMC would be getting involved, and it could have become a lot more horrific a situation than it already was and so there's a lot of stress from- from that and trying to... salvage or solve that problem, so I could stay at my job, stay at my partnership will really, really drive us... so it was really tough and the price of my- There was not many people I spoke to at my work, and I literally became very, very withdrawn with- with work, I think it impacts on my- even though (*unintelligible*), I would have expected, not to sound bitter or anything, but you know when I left I thought there'd be a lot more of a 'we're really gonna miss you' literally no one got in touch with me, I think they just got used to me not being there? But you know that person that was literally one of the guys that'd always be hanging around with them, no, no longer hung around with them at all so... We have about 25 staff, and I only talk to three of them intermittently now? Whilst pre pandemic, I would literally be spending all day and all night with them, and I'd know about their families, I'd know about their kids, I'd know about their relationships, I'd know everything about them and now it got to this stage where now I don't speak to- I barely speak to any of them, so it's been tough in that way.

Interviewer: OK, so what impact has Covid had for you personally then? Going from that- from partner to locum, how do you manage that?

GP8: Um, with difficulty is the honest truth (*laughs*) and the personal impact for me- Yes, Covid was there, I think the problem that I had would have happened irrespective of Covid to be honest. There was, um, financial irregularities, **REDACTED colleague name** was stealing money for the practice and stuff but yeah so that- that's not nothing to do with Covid, that's just him being an asshole. You know what I mean? (*Laughs*). So those fights would have still happened and I still think I would have ended up in the same situation I was- I think the difference would have been if Covid wasn't there is that I probably would have had a face-to-face chat- chat with him and meet with him? Because we weren't doing that, and really by that point I'd lost trust with him, so I was emailing- we were talking by email by that point, because there was no point trying to have a face-to-face conversation with you because you're such a, um, uh, difficult character to deal with, to be honest, so, um, we- we went away from- from doing that, but from a personal impact- point of view it's been tough, to be honest, that nursing home I talked about- we lost- the 45 bedded unit, we lost 10 of the patients of Covid and we- we normally would have, pre-Covid, we would have something like two, three deaths a month? We were getting about... so over the whole period of Covid we expected about- So the first lockdown, from about March to June, we were- normally expected about... 12 deaths? We had about 50, so we had a five-fold increase in deaths. So sort of patients had a really good relationship with, and not being able to see them properly before they passed away, had an impact, um.

Interviewer: Very painful, yeah.

GP8: It had an impact on my personal relationship as I say, in terms of, um... my colleagues, people I cared about, your reception staff, nurses, pharmacists, our practice manager, a guy that I classed as one of my mentors. Losing all those people in one swift go was tough, I actually didn't work November and December, I took those two. So once I'd got out of the practice and just left the practice, I actually took that time as just time from clinical roles? I didn't do any clinical work in November, December. If it wasn't for the fact that I was worried about losing my clinical skills, I probably would have taken more time off? Um... because I have other roles outside that which has to do with workforce or help with GP retention and I'm also now the **REDACTED role** for the CCG, but-

Interviewer: Are you?

GP8: Yeah, yes, that's another thing I picked up, say again?

Interviewer: I'd like to ask about that, because I wanted to ask about your changing roles as a GP, can you tell me about that, the vaccination programme?

GP8: Um, so yeah, I- basically I left (*unintelligible*) and I had a lot of spare time on my hands are I was taking it more for recuperation and getting better from, all the crap- because with all the stuff I'd been through I'd lost weight, I'd lost my hair, I'd lost everything, it was really crappy (*laughs*) I was actually a lot worse than- than I am now, and the CCG just said look, we need someone to **REDACTED** for our CCG to help support our GPs to implement the Covid program, would you be interested to do that? And, um, I wasn't doing anything else, so I thought it'd be good to keep my mind active and so I've been involved with that so it's about really helping practicing **REDACTED CCG name**, uh, and then what was originally the 85% target, but now has become the 95% target, vaccinating 95% of your cohort one to 10, um, and so it's been an interesting role, it's been a lot of, um, negotiating with other practices, and speaking to other GPs and stuff about how they're going to do this and how they're going to work together, because obviously with the Pfizer vaccines they're too difficult a thing to give, you can't give it in a practice, it's not like the flu vaccine campaign, where you know, you just get you know, a couple of thousand in your fridge and you usually just tell people, this has gotta be, you know you've got to get nearly 1000 done in three days, three and a half days, sometimes, and so working at a scale has to happen. And trying to convince certain practices to work with other practices that they don't like, they don't get on with, has been challenging, but then also making sure that they have the right processes in place. A part of my role has been in quality assurance, so I've been at their sites, I've checked over their sites in terms of when- before they kicked off and they started up, I would go to all the sites, or go to a few of the sites, to check the site- Have they got social distancing there, are they going to store the vaccine properly, are they going to vaccinate people properly, do they know how to

give the vaccine, how are they going to check everyone's got the training, you know, all that kind of stuff, so it's been an interesting role in terms of that, um, so that's changed a bit.

Interviewer: Um, how prepared did you feel? You've sort of told me this already, but how prepared how did you feel for the pandemic as a GP professionally?

GP8: None whatsoever (*laughs*). It was- it was nothing there was none whatsoever, and literally it was making up decisions, initially on the hoof, and just making up what we thought- yeah this sounds right, that sounds safe, and then obviously, we got the, uh, NHS England standard operating procedure come out, which is updated on a regular basis, but changing those kind of things was- was- was huge, to be honest and it made a massive- yeah so in terms of were we prepared at all for this, none whatsoever, we didn't even have enough laptops. I remember when we started working remotely we didn't have enough laptops, so we had asked for about five or six laptops from the CCG, they also gave us thing called 'away from my desk logins' which we had- none of us had those things, none of us who were really prepared to work from home, and literally in the space of about three days getting ourselves changed from working in the practice to working from home...

Interviewer: Yes, a huge- a huge quite stressful shift definitely and- and when you were in the practice, did you have anything in the way of PPE or, um support, sort of social support?

GP8: Um PPE, we- we didn't have any initially and obviously we- we- we stocked up massively and the most sad bit of that was where, literally, that nursing home I looked- we looked after, were calling us up saying 'can we borrow your PPE, because we haven't got any' and in the end because cause we- one of the smart things we did was one of the practices we worked with, the head of the practice at a scale of about 60 to 70,000 to go to one of these providers called medical supermarket, and so we were given a preferential treatment because we- we were classing ourselves as a big organization, 1000 patients, so we were a priority person for them? Well, unfortunately, they wouldn't- they wouldn't- they wouldn't supply the nursing home because they weren't NHS based? So the nursing home would call us up and say- hey can you order gloves, aprons, masks for us, because we can't get hold of them- um... which is pretty scary to be honest, and we were ordering them on their behalf and having a bit of a relationship in that way but initially we had nothing we had the box come from- from NHS England, which was a joke. It literally came and we were looking at it going... we're buggered, this is all they're going to give us, and literally at that point we started buying our own stuff, I remember, I... I went against the NHS England guidance, which is a surgical mask, I have one of those big deal- it's kind of used when you're doing spray painting and stuff (*laughs*).

Interviewer: (*Laughs*) Yeah.

GP8: I've got one of those. And I where that everywhere- and everyone looks at me like- are you crazy! Like I don't care what I look like I actually, yeah, actually just want to protect myself, so I've got one of those big BAME-type masks, to be honest, and I wear them when I'm seeing patients face-to-face.

Interviewer: Fair enough! Um, could you tell me about your opinion of the government response to Covid-19, in terms of public health policies, I'm sure you've been mitigating for patients who call you asking you about guidance?

GP8: So... very disappointing you know, you see- hear what's in the press, you hear that our Prime Minister is not going to Cobra meetings on this thing five weeks before it starts, you look at, uh... When we locked down, and we should probably have locked down a couple weeks before that, you look at uh, Cheltenham festival, you look at a physical Liverpool vs Madrid Athletico, uh- I'm a big football fan, so the one that still never make sense in my head, it was, uh, Ateletico Madrid fans couldn't go watch their own team in their own stadium, because of Covid, however, they're allowed to get on a plane flight to Liverpool and watch Liverpool play Athletico Madrid with 45,000 British people in, uh... and that's just, you know, that's the bit where you just think what the hell, and I think the public messaging from them was really poor at the start when you've got Boris Johnson going on about, you've probably heard all this stuff before so I'm sorry I'm going on about it-

Interviewer: No, it's your experience, so-

GP8: Yeah, um, you know, Boris Johnson talking about how he went to a hospital, he shook hands with people that have got Covid, he's not going to wear a mask at first not being helped- also international policy and international comments by people just Donald Trump where he's saying he's not gonna wear a mask and he's not advocating to wear masks, I think, unfortunately, the weak government response we've had is what's probably fuelling... so a lot of people are anti-lock down and anti- all these things have been... they've been facilitated by the government, to be honest and because of the mixed messaging. We needed a strong message and we instead got a very weak message at first. You obviously look at, um, the guidance in terms of social distancing, the opening up, you know, the eat out to help out, which just seemed like a bonkers idea- for myself, I didn't- personally I'd tell patients when that came out, don't do it, and I was advocating for patients don't go to restaurants- just because restaurants are open, doesn't mean you have to go there, you can stay home and do those kind of things, so I think, from a government point of view, I think, the response is really poor. You've obviously now got some Brazilian mutation that's got into the country, and you look at that and you think right why the hell are our airlines- why the hell are we still open, why aren't we locking the border, you know what I mean? And you look at, if you look at these places like New Zealand, and how well they're doing with it and Vietnam and Taiwan and all these kind of places and you think- Okay, New Zealand is, but Vietnam

and places like that, they're not, you know, we are a more successful economic country, then them, and if they can implement these changes, why the hell can't we? And, you know, we've had- we had China's response, we've had South Korea's response, we had all these other responses about how we should manage this and we ballsed it up completely. As a as a government, the government on our behalf, you look at track and trace and you look at how rubbish it is and you just think, well, this is not good guys! The vaccination program you've got right, and I admit I didn't particularly agree with the three weeks to 12-week change in the vaccine Program. Um, I thought I was a massive risk but it looks like that's paid off, but frankly I don't think we should be betting on people's lives and stuff, do you know what I mean? But the vaccine role has been very, very quick and again, if you look at, in terms of that one you, look at the mass vaccination sites which I think are, um, a waste of money, I don't see what they're doing, you're getting lots of regular things where they're not getting their slots and people who are not in the right call are getting appointments, and you get people who are working, you know working in GP-land like I am where we're trying to vaccinate enough, get everyone in the right cohort and we're telling off PCNs when we find out that they're not doing that, and you've got the NBS sites sending out text messages to patients, or people going home going, um, 'there's no queue, go down there now get your vaccination' at the mass vaccine sites, which is really frustrating I don't know why we needed to make those mass vaccine sites. Um, I think it's a bit of a mistake (*laughs*). Oh, god!

Interviewer: (*Laughs*). No that's good, a vent is a good thing! Yeah and I mean nothing you're saying I haven't heard before as well, um thank you for your answer. Um are there any changes which you think should be carried on into the future?

GP8: Um, to an extent, yes, I mean, I think I was talking to someone else about this a couple weeks ago, and one of the big changes I think is, we need to be, I think, we're now going to be a lot more open to digital solution problems, and I think, um, video consultation's staying for good, and I think that's a good thing, because a lot of things that we get people to come down to- to waiting room and, you know, sit there in waiting rooms when they didn't need to, and we can be a lot more adaptable, work around patients' lives, and you know they can do they, do they- see us at work, you know what I mean if they go into a private room they can go and see us at work, from that point of you, or that patient who's got a rash, who's home visits-wise has a massive impact because we now don't really need to do as many home visits- there'll still be a few we need to do, but overall there's a lot of things we could do remotely now, um, I think we need to find a hybrid model, I still- I still think when we get back to whatever normal is going to be, I still think 50 to 60% consultations are going to be face-to-face, but I think 15, 20% would be phone call and the other 30% would be digital now, and that might be video calls, I know lots of places are using things like e-consult and stuff like that, and these are ways to try and help streamline us and make us more efficient. Um, I also think, in- in a good way, this will give the opportunity for patients to take a bit more responsibility for themselves, you know they can check their weight at home, they can check their blood pressure at home, they don't need to come and see the nurse to get their blood pressure checked anymore. And so, there's lots we can do that way.

Interviewer: I wanted to ask has patient- patient presentation changed much for you and how have your patients been with the change?

GP8: Um, so the, complaints have goes through the roof I think everyone complains now that they want to see the GP, you know, unfortunately media-bashing of GPs say that we're always closed and stuff, and patients calling up saying 'you're then shut, you know, you can't be shut'- we're not shut we're bloody open we're working our asses off. Demand-wise it's gone through the roof, and I think people think that demand wise has gone massively up in terms of, um, the presentation I think people are presenting more soon now, I mean I've had, an example I'll give you yesterday, the locum yesterday, where a patient called up because she knows you don't have to come into surgery she called up because she'd had a headache for two hours we've got an appointment and it's like, wow.

Interviewer: A very quick turnaround.

GP8: Yeah exactly we're doing too much- the turnaround's now much quicker than it's been ever before, in terms of- quick, we've got to get them seen, we've got to get them seen, and I don't think that- The speed at which general practice is working right now isn't sustainable and we're going to need to reset post-lockdown, post- post- post-the new normal, we're gonna have to reset patients to their expectations, I think in some ways it'll be quicker, it may even come to a bit where we'll start saying we'll give shorter video-telephone consultations, or even with rashes, I mean, I think a great idea is a photo clinic where a patient texts in, says this is my rash, this is how long I've had it, answer all these questions that you'd want them to answer at the GP.

Interviewer: Okay.

GP8: And you respond via text message. You don't need to see that patient and by doing that you've saved yourself loads of time. Um, my brother is a GP as well, he's made a program called 'my pill check' which basically-

Interviewer: Good for him, he's made a program in lockdown!

GP8: Yeah! He made it pre-lockdown he's got that started, and then it-it basically, it was meant to be just for the pill, because he's been (*unintelligible*), but he's actually now pushed it for learning difficulty- learning disability assessments and asthma checks, and normally asthma checks would take nurses 15 minutes. If a patient fills in their asthma control questionnaire, pre-

appointment, sends in their peak-flow readings, your nurse could actually get through- in an hour normally a nurse could do four, an hour? She could potentially do 20 an hour now if she was because, and so it speeds people up, and so what you could do is a bit like I think what we all want to get to is break away from the 10 minute appointment so get the simple things done quickly, and then you've got 20 minutes for that really complex patient, you know it's going to come in as a face to face. And so, in that way it can work well.

Interviewer: Great, thank you, that's a great answer. And another thing that I haven't asked about sorry I've moved towards the end but I realised- have you spoken about referrals? Have you found delayed referrals- well, delayed waiting times for the patients?

GP8: Yes, massively there's a number of, um, in terms of delayed waiting times for a lot of things it's just gone through the roof. Two-week-waits, still stuck to it, but in terms of outside that any routine referrals. Um, an example I can give, um a dermo- again- a dermatology referral done for a two-year-old normally that would take... using a community dermatology model would normally take about six weeks, but- but the child was seen in nine months and so yeah, they were referred pre-first pandemic, and only got seen a couple of weeks ago and you see...

Interviewer: Nearly all of that child's life.

GP8: Yeah exactly, because we were waiting for a dermatology referral. A lot of things where it's getting better now, we're in, they say we're in the recovery phase, but still even now, we're being encouraged as GPs, rather than doing straight referrals to do advice and guidance referrals now... a lot more, and the idea being is that the consultants can then vet those referrals, work out what needs to be seen, and then reduce them that way as well.

Interviewer: What were they called, sorry, guidance referrals?

GP8: Advice and guidance.

Interviewer: Ok, so is that sort of passing the role back to you in managing those patients?

GP8: Exactly, so the bit where we'd say oh, we need to get this patient, you know we've got a patient with X, Y, Z condition, we want, you to see them. Now they might say- Oh well, I've looked at your referral and based on that I think they need test Y, go request test Y for me, and see how it goes.

Interviewer: Right

GP8: And so that's great in some ways, but in other ways, it makes you a bit like the House officer, the junior, which is not the right way the relationship should work, um, it's easy in some ways, but there's bits- there are some really complex tests which we have no clue about, and I'm like no actually I think you need to see this patient, why don't you organise the test, you can test them, and then you can look at the result, rather than getting me to look at it, and then also, it's delaying for the patient itself, because what if that- we do that test it comes back abnormal then we say yeah they need to be seen by you, and then they go 'alright, let me book them into my clinic, oh my next appointment is six months away'. Do you know what I mean? I- in terms of that way, it's not- it's not perfect, so in terms of referrals, uh, and also surgery, any formal surgery, it's just been crazy now, so I saw a patient in Kings Norton las week who'd been referred for a total knee replacement in February last year, got seen after the first lockdown, and was told 'yep, we'll book you in' but since then there's been such a long wait list, they are still waiting- it's been 18 months they've been waiting for a knee operation now, uh, a total knee replacement, so it's had a massive impact or waitlists, to be honest.

Interviewer: Thank you. Um, what do you think we can learn from the pandemic thus far to inform future GP care?

GP8: God that's a really hard question to answer! Um, I think it's um... so what we've learned from this pandemic is A) we need to accept and embrace digital changes and remote changes to-to patient care, and how we can work remotely manage patients, we have- but we've also got to learn how we merge that with the old way of working and how we, we find it- a hybrid that works for us in terms of it- yeah that's all I can say, that was a good question.

Interviewer: Thank you, that was a good answer, thank you! Um, is there anything that we haven't spoken about today that you'd like to talk about, anything that I haven't said that was salient to you in the pandemic?

GP8: I think I know I touched on it, and I think you probably- but the- the media...

Interviewer: Yes, tell me about that.

GP8: Yeah, the media side of it, I think has been really awful this- with some particularly journalists have been pretty- pretty ridiculous in their comments about general practice and that's not helped our- our bit and even now, when you look at, uh, the Covid vaccinations, I don't think GPS are getting the credit they deserve, for all the work they did on the Covid vaccination?

We're doing 75% of them. And it took me- on TV we've done what 21 million that we've done now? 75% of them have been done by general practice. But, we're not really getting the win for that that we deserve to be honest, so I think it's a bit disappointing.

Interviewer: Thank you for saying that, um, I hope, I'm only a student but I really do hope that this research might highlight that, um, because other people are saying the same thing, it's just- it's just awful to work so hard and have the media work against you know.

GP8: Yeah, it's a bit rubbish! And it's not good, it's not good for two reasons, one it's not good for- for our morale, but also Minka we want you to come into general practice, you know, every medical student, we want you to go into general practice, and I say this as the retention lead for- for our area, we've got to make you know general practice more attractive and at the moment, unfortunately crap like this happening, with people saying rubbish like this, isn't going to help us attract people like you in years to come, because they're going to think that GPs are lazy, and GPs are this, and they want to look at the more, um, sexy, amazing A&E kind of jobs, which are going to impact on us. We're this wonderful gatekeeper for- for- for the NHS in itself.

Interviewer: I can say that, um, because I'm at Birmingham I'm quite privileged in the sense that we have um, we have a GP placement every other week from first year, all the way through to fifth year, and most places do more clinical places at first, so I'm grateful that I've gotten an exposure to general practice outside of what you hear? But, um, yeah, this has definitely not deterred me from general practice as well…

GP8: I was a Year 1 and a Year 3 tutor, while I was practice lead- I was a Year 1 and a Year 3 tutor, and so I gave that up as well, unfortunately, when all this hit, we were arguing this much we were like we can't teach, which is really a shame, but that was rubbish, yeah, yeah.

Interviewer: I'm going to stop the recording here.

Recording ends.
