# Supplementary material for: Primary health care during the COVID-19 pandemic: A qualitative exploration of the challenges and changes in practice experienced by GPs and GP trainees
Source: PLoS One. 2023 Feb 9;18(2):e0280733. doi: 10.1371/journal.pone.0280733 (PMC9910752; doi:10.1371/journal.pone.0280733)
Supplement: S1 Data — (ZIP) [file pone.0280733.s005.zip › GP9 Transcript.pdf]

GP9 Transcript

Interviewer: Okay, thank you very much, um, so now, can you tell me a little bit about your general experience in general practice, sort of pre-pandemic.

GP9: Um... I work three days a week. And I do a day of training with med school, I do CBM on one day.

Interviewer: Oh yeah.

GP9: Um, so actually I don't have a huge amount of clinical exposure, I do two- two fully days of clinical exposure a week. Um, which is a nice balance of things, I have two, um, I now have two very young children and at the start of the pandemic I only had one very young children.

Interviewer: Congratulations on the new one then.

GP9: Thank you. (*Laughs*). Thank you, that's why I look permanently tired. And... I had- I still do have a really great job, I really enjoy general practice. I really enjoy where I work, I think it's a great practice, it's well organised, it's a bit different? In that we don't really- We didn't really have appointments. We have a drop-in surgery so between the hours of half eight and 10 o'clock in the morning and four o'clock six o'clock in the evening you could turn up, and sit in a waiting room full of people, you can imagine why this went over the course of the year. Um... and you would get seen, and we would see between... um... 30 on a good day and maybe 55 on a bad day people- between the four of us in two and a half hours. Um, and it meant there was quite a satisfied population that had easy access to the practitioner that... And we thought we were offering a good service. And I enjoyed that, and it was whatever came in, came in, and you saw it. And then, some of it was acute and some of it was... chronic stuff and it was all mixed in, and it was, um, yeah, I really enjoyed it!

Interviewer: Could you tell me about your practice demographic and population?

GP9: Um... so our part of **REDACTED city** is predominantly white population- white British population. When you compare it to the rest of **REDACTED city**, um, most of our patients speak English as a first language it's a- yeah a- a mixed group in that I would say most patients are poor and there's a large amount of unemployment, um, but dotted within that, because there's some really big houses within **REDACTED city**, over the last few years we've had people move out from **REDACTED area** and **REDACTED area** and so on, that you might associate more with people that live in those areas- doctors and university workers and stuff that live in... in that community as well, so it's quite- it's quite mixed but they're only a handful.

Interviewer: Okay, great, thank you that's a good instruction to your practice, and you're still working there now I assume?

GP9: Uh-huh.

Interviewer: Ok cool, yeah, could you tell me- and it's a broad question, I've got more specifics if it's too broad- um, can you tell me about your experience of the pandemic professionally?

GP9: Um... just as an open question like that for me to go with as I choose rather than...?

Interviewer: Yeah well um, I suppose, for a start how prepared were you in terms of physical support? Like PPE or guidance? So, emotional and clinical support?

GP9: It all came, sort of suddenly, didn't it?

Participant had to take phone call, paused during this.

GP9: I'm sorry, um?

Interviewer: Do you feel in your practice to move, for example, to telemedicine?

GP9: Not. Not. Yeah it just came out of the blue didn't it, and it went from being just something we'd watched on the news to being a growing prospect, and then we went to people on the front desk.

Interviewer: Yeah.

GP9: Have you been to Korea in the last three weeks, well no, none of our patients will ever go to Korea. And then it became uh... suddenly changed and we just suddenly changed overnight, we went from running surgery where everybody's sat all together, to that couldn't happen, we have to stop that, but we did that, before lockdown happened.

Interviewer: Did you?

GP9: Yeah because it was- it was clear that having lots of people in the same room all at the same time was a bad idea, and that it was becoming difficult to differentiate... All the coughs.

Interviewer: Yeah

GP9: Yeah not- not forgetting was again in general practice that in 2020, 2019 into 2020 we had already had a reasonably bad flu, so there was a lot of cough around, and they became really difficult to manage, um... (*laughs*) or at least that's how I remember it. And then we just stopped. We went from hardly ever use- as a practice we hardly ever used the phone, we did do a lot of telephone calls and we didn't have an option to do telephone... consultation as a practice prior to Covid, or maybe we would have like two calls a day or something like that, and then a... a disorganized- we call it the 'message book' where you put anyone that wants to speak to a doctor and they might accept that they get run back at any point during the day, so that's how we did the phone, but everything we did was face to face. So that would changed very significantly and all of a sudden, no, I don't think we were prepared for that, but I think, the practice as a whole is very flexible and team driven and we coped with it quite well.

Interviewer: Great.

GP9: And I think it helped that at the time, there was a sudden seismic crash in demand. There was- as- as it appears that this, this would happen, and you know everybody did that panic buying of toilet roll and so on, they also panic-stocked they're- mostly inhalers and stuff so there was a big drive on demand for prescriptions and then, once that was over, we- because we switched on to calls we sent everybody in the community a text to say, and a letter, I think, we're, this is what we're doing from now on- we will let you know as we adapt- adapt the system, and so there was a huge rush on getting stuff sorted before it was gonna hit in and after that there was nothing. And the thing that allowed us to cope with the change because, at the beginning... when was it, 23rd of March?

Interviewer: Yeah... I can't remember the specific day?

GP9: As it drove into April and hospitals went crazy, we at general practice were talking about taking 50 phone calls between… four people in the course of a day.

Interviewer: Okay. And was that manageable then?

GP9: So, um was that manageable than. That was really manageable, to the point of feeling a bit guilty that you weren't contributing to the...

Interviewer: Yeah, so did you have video consultations, or texting, or was it all telephone based?

GP9: Um, it was all telephone based, and then, um, the video consultation... I started to adopt using video consultations and then over the course of year I've got rid of it again. It- AccuRx came through a few weeks into it, that we could do this and use their services, texting and sending pictures and doing video consults and stuff so we, uh, played with that. Other doctors do things differently, uh, I don't find video consultations, particularly helpful.

Interviewer: How have you found the process of consulting patients by telephone, in terms of the responsibility of risk stratification and not be able to see your patients?

GP9: Um... on the whole I hate it. Um, it definitely served a purpose, we can definitely take something from this and learn things, and change our technique and offer a variety of services that suit people differently, um, but in terms of thinking about it purely from how I consult, how I get information from patients, how I feel about safety, in terms of risk and stuff- I really, really dislike it.

Minka: Why's that?

GP9: Um, it puts a barrier in front of you- in between you and the patient, that is difficult to surmount. Um, some people are very capable of using the phone and making themselves heard over the phone and some patients cannot.

Interviewer: Right. Would you say it's accessible generally, or no, not so much?

GP9: It's certainly very, very accessible, I'm not saying that they can't physically use the phone, but in terms of their intellectual capability to be able to describe, a new, difficult symptom like abdominal pain over the phone without the benefit of you poking and prodding them at the same time, and them pointing to where you're talking about. Trying to use it as a tool to specific- to come away and think do I specifically- am I confident that I know exactly what they're trying to tell me? And a lot of the time the answer is however hard I've tried to do that, it's no. Especially, throw in an extra barrier like a translator- translated phone call going three ways and it's a bit...

Interviewer: Yeah, so difficult.

GP9: And then it's all extra bits that I don't enjoy- you've lost the person touch bit that makes being a doctor nice, the bit where you get to speak to people and be people with other people. You also lost the all of the unspoken communication, I was- I recognise this year I rely on very heavily to look at people, to try and read their tone, try and read their face, read their body language as they are talking so that's all gone and you're relying upon what they say literally and- and I find the confidence in the answer that I'm getting, like am I sure, in what they're telling me, is lost versus when they're in the room.

Interviewer: Yeah.

GP9: It is definitely risky. Riskier, that is, um...

Interviewer: How does that change your management of your patients and your relationship with your patients?

GP9: Um... some of the patients really liked it and they found it benefits them in their level of accessibility and what they can deal with. It's an okay tool to deal with certain problems, but it's a rubbish tool to deal with a lot of problems and it's knowing, knowing that and working out- and- knowing your patients and working out which of them will be able to help you- which- which you will be able to help over the phone and which you won't. And I think for some of them they're satisfied that they've had a consultation with the doctor, and some of them are under the impression that, unless you see me face to face that it's not a real consultation and that we're inaccessible, and that they're not getting an actual service, despite the fact that a lot of things, that they've phoned up about- and would be dissatisfied with the services offered, are absolutely manageable over the phone.

Interviewer: How did you feel making decisions with the guidance that you'd been given, um, in terms of just the information you had about Covid and the risks of it?

GP9: Um... I don't know if any of the guidance has ever been particularly helpful or up to date with what was going on.

Interviewer: Okay.

GP9: Yeah, Yeah I don't know.

Interviewer: OK, that's fine.

GP9: I don't feel that we've ever had any decent guidance that told us exactly what we needed to do and- and how we could risk stratify and we were kind of left to our own devices to do that, which is fair, in a way, because, the building and the layout of the building is as integral to managing risk of bringing people in and out of the building, as- as the rules are in the spread of the virus, and so it's individual to the individual practices.

Interviewer: OK. Sorry I was smiling at the little one, it's quite sweet (*re baby in background).*

GP9: (*Laughs)*

Interviewer: It's not noisy or anything, um, so, in your experience how have GPs being utilized or how has your role changed? Have you had to take on roles that would normally go to secondary care, administering vaccinations, anything that's different to your care prior to the pandemic?

GP9: Uh, yeah we're... probably doing some of the stuff secondary care is doing. I don't know, not a lot from what I can tell. Definitely taking more risks and avoiding referral when we can do it.

Interviewer: Right.

GP9: We've done vaccinations, I've started vaccinations- I've been vaccinated myself as well. I suppose the difference in the- The general public is getting told or was certainly getting told that general practice is still open.

Interviewer: Yeah.

GP9: And from my point of view, sitting in my chair at general practice, that we were definitely open, and we would definitely accessible, and we were definitely seeing people. To which they were- seemed to be surprised that we were there, the patients quite a lot of the time. But next bit was that they would present you with something that usually, you would refer- I, part of my work is to refer to secondary care, and then that aspect of it was completely shut.

Minka: Right, yeah.

GP9: Some of the hospitals just cancelled, all of the scans that had been booked between March and June and didn't tell us they were, and stuff like that, um... some general surgical, and you know, I totally understand why they did it, but it, the message to the public, we are open, but at the same time, they can't do anything about any of the problems you're going to present to them. Yeah. Was incredibly- has been and still is very, very difficult to manage. People with severe knee pain, people need operations and stuff and they come in, and you assess them, and then you say 'well here's some painkillers I can't do anything else about this at the moment', even if you wanted to see a physiotherapist face to face you're talking about the waiting list being six to seven months right now.

Interviewer: Right.

GP9: And so, the feeling of being completely useless and everything being futile is- is definitely there, and then you question why did I bring you into even look at this problem, because I knew on the phone you had a bad knee and I wouldn't have be able to do anything about it... but it's balancing this concept of being seen, being available, the tool of- as the doctor as part of medicine, rather than just to say like, I got seen, and therefore I, the problem was treated seriously. I kind of- the psychological mindset that goes with...

Interviewer: Yeah it's interesting what patients perceive as like a complete consultation.

GP9: Yeah and but- at the end of it we're telling them I can't do anything, I'm really sorry, I can write a letter it will either get rejected or you'll be added to a list, and right now it's highly likely with a lot of specialties you will not be seen before 2022, and selling that, um... it's been really, that has been really hard. And people are extremely, and rightly so, frustrated by the whole thing.

Interviewer: Sure.

GP9: Because they're getting told that we're just open for the business as usual, everything's running, it's just not-

Interviewer: Yeah the messages- the communication does sound off from the way you're describing- um, between the official communicators and what's actually going on in GPs. Um, that sort of leads me onto my next question, which is what is your opinion of the government response to Covid in terms of public health measures and policies? Yeah.

GP9: Declaring my conflict on interest before I start, I'm... rabidly detest the Tory party and everything they stand for.

Both laugh.

Interviewer: Noted.

GP9: And so... it has shaped, probably, some of my opinion and it's not because I'm a... hardcore Labor supporter or anything, it's because I can't stand what they stand for.

Interviewer: In terms of their policies, or communication?

GP9: The communication's been terrible, the, yeah- Especially towards general practice, we were finding that things out from the news and we still continue to find out things from the news.

Interviewer: You as a GP, you say?

GP: Yes.

Interviewer: Right, that must be very frustrating.

GP9: Yeah so you'll have seen- I'll get back to the beginning, but as a tangential point, you'll have seen that there's a South African variant and it's been out in Birmingham, and it is in Northfield.

Interviewer: OK.

GP9: We didn't find any of that out from public health or government, we found it out from the BBC, and we've still not had an official point on it, and everything we've received has been gossip. Knowing that we were going to lock down has always been gossip, there's never been, you know- Here is some information for you, as the health care provider, this is what you're going to do- it's just 'follow the news make it up as you go along', yes, so, on the receiving end of government bodies it's been poor, um, their handling of the early crisis was atrocious and has contributed to the death of 10s of thousands of people and there will never be a, um... inquiry that is actually meaningful, but they could see it clearly in France and Italy and Spain what was happening, and they still didn't lock-down for another two weeks, and they allowed things like entry... and whatever to go ahead. The- the football and Cheltenham goal cup, all went ahead, even though they knew- and it seeded it throughout the

country didn't it, and- and so that they had the warning, they knew- I know we're not an island, like New Zealand, but we're an island like Japan, um, in terms of us being a regional hub, very densely populated. With a certain amount of control and reasonable messages and global closures, we would have controlled- controlled it better. That said, their vaccination response has been excellent, um... and I want to fault them, because of everything that they've done with Dominic Cummings and so, and that was also another... um, that- That taught that nobody cared about the rules and there wouldn't be- there wouldn't be any consequences to the rules, and, it- lots of people tried to downplay how serious that was as a trigger point throughout the year in terms of adherence and obedience within the general public, but it was important because they showed in a very public way that they- the- the body that are in charge of this aren't following the rules, and so what we see in general practice is actually that no one is following rules, you ask them to lock down and they're not and I think part- it's not all him- but some of it is. Sorry I'm getting wound up again, the, uh, um the vaccinations they started off-

Interviewer: It's normally-

GP9: What's this?

Interviewer: It's normally when I ask this question I have to go, I'm sorry, it's a bit contentious, and they'll go it's fine and start and then, once you do start thinking about it, there's a quite a lot of facets to unpack! (*laughs*). Um, but that's fair enough, but yeah the vaccinations definitely sound like the silver lining.

GP9: Um... they got it right I'm not sure they meant to get it right, and again communication on it's been poor. They were telling the public at the beginning that vaccinations were out there, that everybody was getting blasted with vaccinations and- and we weren't seeing any vaccinations on the ground.

Interviewer: OK, right.

GP9: We got offered 13, 1-3, Oxford vaccinations one week. For your- For our practice. Yeah, we got- and then- and then they said- the deliver- in the delivery-the delivery of all of the vaccination stuff was rubbish to start off with. We've got it now, and it's coming- it comes through regularly it's still short notice, but, we are doing it and it's clearly- clearly going really well.

Interviewer: Yeah. But your practice experience, is perhaps, um...

GP9: The reality within the first few weeks of the vaccines kicking off was it was really disorganized and no-one knew what they were doing and they hadn't thought- they were they were relying upon general practice to pick up the pieces for them.

Interviewer: Yeah?

GP9: Here's vaccinations and we don't know what we're going to do with it, can you sort something out. And we did, the issue of money for general practice and vaccinations has been interesting because they've...

Interviewer: What's that, sorry?

GP9: They've splashed a lot of money around, um, with Covid and with their- some of their, um, and some at university or whatever have received huge banks for PPE or whatever. General practice will struggle to break even on delivering Covid vaccinations, or it did- it definitely did, to start with, we were being asked to do them on Sundays, um... so... and- and the beginning of the Pfizer vaccination was, it was only a doctor allowed to mix the Pfizer vaccination, it was...

Interviewer: They weren't allowing nurses to do it?

GP9: Not at the beginning or not, on the local patient group directives, CCG directive, so you had to have senior people there to deliver, and that costs money.

Interviewer: Yeah.

GP9: I work two Sundays, eight hours or whatever, and I, because I like my practice and because I want this to happen, I work for my normal hourly rate on a Sunday.

Interviewer: Normally, that would be considerably more?

GP9: (*unintelligible*) double time on a Sunday, which is only reasonable to ask people to give up their time with their family.

Interviewer: Sure.

GP9: But the practice as a whole, because they were getting paid not very much money per patient, was running at a loss. It just seems that their whole mentality towards spending money has been very loose and then suddenly it comes down to this, which is the real thing, that- the key, you forget ITU, forget everything else, this was the bit that was gonna solve the problem. Rather than rescue people, this is going to prevent it continuing and they couldn't pay for it properly, and they let everybody...

Interviewer: That's disappointing.

GP9: Let everybody do it out of goodwill, and they ask for a volunteer force rather than paying for it, um, which is fine, because it's getting done and on the whole, is really good, but it just...

Interviewer: I'm glad you raised that, because my hope is if this research goes anywhere it will highlight the, you know, the gaps that GPs have had to fill quiet- quietly. And- and yeah, um, from speaking to most GPs, it just seems that vaccinations been like a huge work effort, um, with not much credit given... That probably sounds a bit opinionated for me to say as an interviewer. That's the experience I seem to be hearing about.

GP9: No, I think you're right.

Interviewer: But yeah if I could ask, this is a more sensitive question so you don't need to answer- you can answer to whatever extent you want, but has Covid had any impact you personally, I understand you've got a new child during the pandemic?

GP9: Um, it's been a challenging year. Um, yeah. My dad died between- just at the beginning of the Covid pandemic, not of Covid, he had bowel cancer, and had been unwell for a while.

Interviewer: I'm really sorry to hear that.

GP9: Oh no, thank you, and we... he had his funeral on the fifth of March, and then lockdown happened a couple of weeks after, and from that moment it's just been... it's been... I haven't really stopped, it's been a psychological slog. I must admit that, at the beginning, I almost enjoyed it? And I know that's weird.

Interviewer: It's not the first time I heard that.

GP9: Um, I, um... which I didn't declare at the beginning, I'm um an infectious disease person by training, before I was a GP.

Interviewer: OK.

GP9: I previously worked for the HIV department at the **REDACTED hospital name**, and I've got - plus I did tropical medicine stuff, and I viruses and disease and epidemiology, so-

Interviewer: It's lot of my course at the moment- global health is like a lot of communicable diseases, yeah.

GP9: So it's been fascinating from an academic point of view and the strange teenage boy side of me that always dreamed about living in some post-apocalyptic world found the whole thing interesting.

Both laugh

GP9: And we had special privileges, where we were still allowed outside our house and still allowed to drive around and go to work and chat with my friends, so life actually has not been that- aside from going to the pub with my mates- life hasn't been that different. Um, and the beginning of the pandemic when it was warm and sunny outside, and you could spend lots of time outside with your immediate friends and family, was all right! And then... I said back in April, isn't it remarkable how wonderful the weather is, and how this is going to keep people going, and then number three in the cold winter has truly been- it's been horrible and I hate it and I'm totally done with it now. And having two small children in this environment is very, very, very hard work, there is nothing for them to do at all, they are, um **REDACTED name of child**, the youngest, has met like five people in her whole life, um... she got, well when she was born she saw a few people, but now she can actually recognize faces and stuff, she hasn't seen anybody in the flesh, other than outside in the freezing cold, um.

Interviewer: That's such a weird thing to think about, yeah.

GP9: Yeah um so, this bit's a drag, as lockdown has gone on, work has become more and more challenging, um, bringing GP back into it, but also how it affects you personally, it's- it was- people being polite and grateful to start off with, and as it's gone on, people's tolerance for this has become less, and less, and less, and less, and they are becoming more and more and more impatient, some of them are becoming more and more unpleasant, and that is a lot. And the, um, the frequency of mental health, which already, given the demographic of lots of unemployed poor people, was quite high, is now spectacularly high. Um, on the callback list every day, there will be at least one person threatening directly to kill themselves.

Interviewer: Really.

GP9: Yeah there was two on Friday, and I took both of them! (*laughs*)

Interviewer: That must be so tough for you.

GP9: It's very wearing to be unloaded on like that.

Interviewer: Yeah! As a frontline- taking all these- yeah.

GP9: I know I'm not- I'm not holding anybody's hand while they- well we're still doing palliative care. I'm not directly staring into the faces of people with Covid in our Community, but we're looking into the consequences of the rest of lockdown, on these places. It's very, very wearing, I only work for three days in a row, but I am exhausted by the time I get back on Friday, because it's- it's non-stop and it's very adversarial, very confrontational work environment at the minute. Taking on lots of mental stuff that is really challenging and unpleasant and a lot of the time we can't do anything about it.

Interviewer: Thank you for such a candid answer, um, it yeah- sounds really difficult. Especially fielding mental health calls and on the telephone I imagine just completely (*unintelligible*). Um, are there any changes which you think should be carried on into the future? And equally changes which you would like to see not continued?

GP9: Um, yes. We're definitely, definitely more accessible in my opinion and that's for the better, um, and as much as I liked the open clinic style thing that we did-

Interviewer: Yeah?

GP9: It's not feasible to sustain it. Even for the next two years, with Covid burning on a slow- on a lower level- we can't bring- we'll never really be able to bring 50 people all together with coughs. That's out of the window. Um... and calling people, we've been we've been able to deal with some problems better, and more efficiently and more directly, and provide a faster service for them. Um... there's a photo thing? You can text photos.

Interviewer: Is that AccuRx?

GP9: AccuRx. I love that. I use that all the time.

Interviewer: Okay, great.

GP9: I don't use the video chat thing very often at all, I use it less than once probably once a week, I find it unhelpful.

Interviewer: That's fair enough.

GP9: In my mind if I need to see somebody on a video chat to determine whether they're unwell or not it's not a good enough tool for that? And the risk is unacceptable, so they either come in to see me or they've said enough on the phone that they should be somewhere else.

Interviewer: Right that's a very good point.

GP9: And that's how I use it, I know other people use it differently, if it's a physical thing I want to use- see, I'll take a photo of it or I'll bring it in. Um, so. AccuRx has been helpful. The secondary care system has changed, and- and if I was being cynical, I would say it's been adapted, their referral system, to avoid taking referrals. But what has come of that is this advice and guidance system where I can write a letter and say- this is what I think, what do you think? And they'll write back and say why don't you do this, this, this and this. And, whereas a year ago, they would have added that patient to a list and they wouldn't have been seen for three or four months. Now I get a letter back within two weeks, regardless of what's going on, and so I've got some kind of thing to work on with the patient, while they wait, and I would like that to carry on, although I would like patients to be seen faster as well, yeah, and I'm sure there's a way of using that in tandem.

Interviewer: Yeah it's a balance between- um, I've- I've had a similar recommendation from other GPs, that telemedicine can be positive and the advice from specialists can be positive, but in a balance, sounds like...

GP9: Yeah, definitely.

Interviewer: Um, I'm aware that I've eaten into your time and I really appreciate all the time you've given today is there anything that I haven't covered that you think would be important to speak about in terms of general practice? Or, do you have any questions for me?

GP9: No, no I don't think so.

Interviewer: I'll stop recording.

Recording ends.
