# Supplementary material for: Primary health care during the COVID-19 pandemic: A qualitative exploration of the challenges and changes in practice experienced by GPs and GP trainees
Source: PLoS One. 2023 Feb 9;18(2):e0280733. doi: 10.1371/journal.pone.0280733 (PMC9910752; doi:10.1371/journal.pone.0280733)
Supplement: S1 Data — (ZIP) [file pone.0280733.s005.zip › GP10 Transcript.pdf]

GP10 Transcript

Interviewer: So to start, can you tell me a little about your experience in general practice before the pandemic? So about your time as a GP.

GP10: Right, well, I joined my practice in 1993. I became a senior partner in... Well, a few years later. And then I retired. Um... but then I was made an offer, I couldn't refuse to be a retainer GP in **REDACTED area name** and that's where I am now.

Interviewer: Ok, when did you re-join general practice?

GP10: Well, I didn't actually stop all together, 'cause after I retired and we had a big party um, I was called back to do a year's maternity leave cover on a salary, and then I did a couple of locums and then I was made an offer I couldn't refuse, so.

Interviewer: Okay, so when did you join this practice in **REDACTED area name*?*

GP10: Ooh, two years ago.

Interviewer: Right. Can you tell me a little bit about your practice in terms of the demographic, so the population you served?

GP10: Well it's a rural practice with about 4400 patients.

Interviewer: Okay.

GP10: Yeah, it's semi-dispensing, um...

Interviewer: Ok. So, could you tell me- big question- but could you tell me a bit about your experience of Covid-19 professionally?

GP10: Uh... well, um, it's changed the nature of the practice. Pretty much overnight. So we went from 100% face-to-face to 99% telephone and video consultations, um... fortunately in my old practice we did a lot of telephone triage, so I was quite used to that.

Interviewer: Right ok, so you had some experience in that. Okay, how have you found that change?

GP10: It's uh, yeah I mean it looks- like I say it's not much, wasn't much change for me, um... yeah... it's been fine.

Interviewer: Do you take the calls from home or in the practice?

GP10: Um, in the practice now. Initially, well firstly- (*laughs*), my son got a fever and a cough so I had to isolate for two weeks, right at the beginning of the outbreak so I was doing everything from home and then for the next few weeks we were doing everything mainly from home, and then we started just going in, doing the- doing phone calls from work, and then bringing people up as necessary to examine them wearing all the Covid clobber.

Interviewer: Okay, um how prepared did you feel for the pandemic?

GP10: Oh, not- not very. We were sent (*laughs*), we were sent some out-of-date masks which had had the use-by dates overstuck with the new use-by date. I'm sure it was fine, but it was... I was in practice when we had the MURS and the SARS, so I was a bit complacent because they fizzled out, and we were all given face masks and- Gloves and aprons and stuff and they all sat in a cupboard doing nothing because it fizzled out, and I have to say I thought the same thing would happen this time. But then it didn't. So I thought, huh, well that's a surprise!

Interviewer: Yeah, an unpleasant surprise.

GP10: Yeah, yeah.

Interviewer: Um, so not much PPE, you said you had 99% of consultations at home, did you have a, sort of, procedure for bringing patients in?

GP10: Yes, locally there's been a hot hub which I've fortunately not been rota'd to do. So you know we direct people to a sort of local hub, where people who might have- who either might have Covid or who have other conditions but might have Covid as well would go and they'd be seen you know, in the appropriate facilities, you know with, um, even better PPE. We took a long time to get proper visors, but fortunately, I had a face protector, which I used for, um, when I use my chainsaw so I used- I took that in. I got almost all of the sawdust off it and then took that in to wear and I'm still using that actually, it's quite good. In between when I'm chain-sawing, obviously.

Interviewer: What are you chain-sawing can I ask, out of interest?

GP10: Fallen trees around the estate (*unintelligible*).

Interviewer: Oh right, ok, well great I'm glad that you had that at your disposal! Did you have any support offered to you in terms of sort of um (*wifi freeze*) did it affect your relationship with your colleagues?

GP10: No. We still just sort of passed in the corridor, 2m, I mean actually social distancing around the practice is almost impossible because it's just not big enough to you know, to socially distance properly. And our practice meetings, instead of being held over a cup of coffee are now done via zoom... So I suppose that's a change. Actually at the start there was a huge problem with, uh, visors so a colleague asked me to print some on my 3D printer which I was able to do.

Interviewer: You have a 3D printer?

GP10: Oh well, I did, and I'd just started to do all these... and it broke! And I just couldn't get it going so I had to order a new one, and... off Amazon, and so I put that together and it didn't work, and so I had to order a new which took a few weeks. I think there was a huge run on 3D printers, you know, because everyone was printing their PPE so I had to wait while to get a new one. It's jolly good though. You're the first person I've spoken to who printed 3D visors!

GP10: Really!

Interviewer: Yeah, and it's quite exciting! Not many people have a 3D printer so maybe that's the reason for that (*laughs*).

GP10: You know I got one as a as a leading present from one- when I was a senior partner, it was great. But when- when I started using it to produce volumes it just packed up and I got new bits for it- ordered new bits for it, they didn't work, so I've got this new one, which can print in two colours!

Interviewer: It's the little things! (*Both laugh*). I'm glad to hear that, that's super resourceful that's interesting to hear, um, how well informed did you feel your patients were about the pandemic. Did they understand that they were having to switch to telemedicine?

GP10: Mostly yeah they were- mostly there were fine. There was a few, um, firstly, there was an impression given that general practices were closed.

Interviewer: Right.

GP10: Which, um, was pretty unhelpful. In fact I heard on the radio our pharmacist saying, well we're having to step into the breach because all the general practices are closed, and as usual I was yelling at the radio saying no, we're not closed, we're just over the phone you tw*t! Or words to that effect. But I do- I do spend quite a lot of time yelling at the radio...

Interviewer: You've got to yell somewhere.

GP10: (*Laughs*). Yeah.

Interviewer: Is that the media then, that you felt was putting out that message?

GP10: Um, there was a there was a bit of a media issue with um, suggesting that we were closed when we weren't.

Interviewer: Okay, how did you handle that? Were patients still coming to you?

GP10: Well, they were still phoning up, being told they were getting a phone call. There were one or two that were a bit iffy about being dealt with over the phone. (*Laughs*). One bloke kept saying, yeah well why can't I just pop down. I said, well we're in a pandemic but we can talk on the phone. Yeah but you're closed. No, we're not closed, this is me not being closed we're talking to you on the phone. Yeah but can't I just pop in? No you can't there's a pandemic on. So. Anyway. But most of them, most of them were fine, and I did do some video consults which I didn't find very satisfactory. Most of the um, well because most of the video quality just wasn't high enough, because most of the patients were using their mobile phones, and you know, you can't really diagnose a rash on a mobile phone, or a skin lesion. So we're tending now to get people to take photographs and send them in which are higher quality.

Interviewer: Is that AccuRx?

GP10: Um, well the AccuRx was the video consults, but we're either getting the, um, photos, you know, sent in by SMS via AccuRx or just emailing through and copying them into the- into the- into the record. Um, all the photos that we get and have to attach to the medical record bring up, um, a warning saying this file is over one megabyte in size and will slow down use of the

system. Which is more of a commentary on the, uh, robustness of the clinical system rather than the size… (*Sees something on screen*). Sorry I just had a warning.

Interviewer: A warning?

GP10: Because we use, um, locally, we use this thing called citrix to... for remote working, and, it keeps wanting to update itself and the last- the IT folks, um, they say, oh you don't need to update it it's fine you just click don't warn me again. But there isn't a bloody box for don't warn me again! So every few minutes I get this thing. Well, I love technology, except when it's crap.

Interviewer: Oh that's good. I guess you do have a 3D printer, so I should've known.

GP10: Yes and I design software.

Interviewer: Do you?

GP10: Yeah.

Interviewer: Oh OK. Wow, okay. Quite an all-rounder as well as GP it sounds like. Which is already the most all-rounder you could be.

GP10: Well that's right I'm a professional all-rounder.

Interviewer: How did you feel making decisions with the guidance that you had, for your patients, with the information you were being given?

GP10: What guidance! (*Laughs*)

Interviewer: Right. (*Laughs*). So how did you feel with the lack of guidance then, I'll rephrase.

GP10: I felt as usual, most of the guidance we get is rubbish, so I'm used to making my own decisions.

Interviewer: And was that- Was it- was it similar to before in terms of navigating... stratifying risk, for example.

GP10: Yeah.

Interviewer: Okay. So you told me about how you switched to telemedicine, and has there been any other changes in your... in GP care for you?

GP10: Well, the, it's um... doing the examinations wearing all the kit is more challenging. My stethoscope normally gets caught up in my mask, and visor and... (*laughs*) I'm quite often nutting the patients with me visor because- when I'm trying to get close-up with me otoscope and I think, oh, and I try and get all the verbal stuff done over the phone beforehand. So I'll just call the patients in, examine them, and make a decision, and have a bit of a chat later so, um, so it's not quite so, sort of, personal.

Interviewer: Um, have you had to take on any new roles as a GP? Be that in terms of vaccination, responsibilities shifted from secondary care, um, anything new really?

GP10: No, I mean I did all the vaccinator training, and I've volunteered, but I'm yet to be called upon. But otherwise you know... most of the stuff I used to send to secondary care still has to go to secondary care, because that's where... if they're having a heart attack I'm not taking on domiciliary tasks like stenting or anything.

Interviewer: I meant maybe more like, monitoring patients during long referral times?

GP10: Yeah I suppose there is more of that, mind you, um, because of the cuts, uh, waiting times were pretty long beforehand, so you know, back in the halcyon days of new Labor, the waiting times were quite, you know, short, you know you'd refer somebody and they probably wouldn't need another consultation before they were seen and dealt with, but um, following austerity, waiting times grew much longer, and you know they're actually- although they probably are much longer now than before- they don't feel much longer.

Interviewer: Okay, and so I assume that's a reflection of your patients as well, are they not finding it to be much longer?

GP10: I dunno! I don't ask!

Interviewer: Okay, fair enough, that's an answer. Has it changed your relationship with your patients, the pandemic?

GP10: No I don't think so.

Interviewer: Ok, how about their presentations?

GP10: Um, no. It's much the same.

Interviewer: Ok. I ask because I've had other GPs who have talked about mental health changing or chronic management changing. It did sound like you were already used to phone calls, so.

GP10: No I haven't really noticed a huge change. I suppose, there must've been, I suppose there's been a bit more low mood and tenseness, but it's- it's- it's a rural population, so they can get out into the countryside and, they're not um, they're not you know confined to boxes. I don't- I'm fortunate I don't have to visit local care homes they're all dealt with by the partners, I have a very advantageous, um, contract, so I don't have to do any visits, I don't have to do any care homes, and I don't have to do any medical bloody reports.

Interviewer: Is that because of the offer you couldn't refuse, the retainer?

GP10: That's right yeah.

Interviewer: Can I ask about that? You're the first person I've spoken to with a retainer offer.

GP10: Right well there's a load of conditions attached, I've got- I've got to have a mentor who is one of the partners, um, I'm not allowed to do more than four sessions or four hours each week. Um... I can't do any other work- any other clinical work, any locums or anything like that, which suits me fine. It's a great excuse, because when people say, oh we're desperate, can you come and do a locum, I say, I'd love to, but you know, contractually I'm obliged to decline! Alas!

Interviewer: Alas, if only!

(Both laugh)

Interviewer: That sounds convenient! Slightly more contentious question, but what is your opinion of the government response to Covid-19, in terms of public health messages and policies, um I'm sure you're a good person to ask, as a person who's mitigating for patient responses.

GP10: Um well, it's been diabolical of course. Has any of your GPs not said it's been diabolical?

Interviewer: Varying levels. I've had one or two people who say, oh it's all right, um, it's interesting to ask because some people have um, the guidance that, like the clarity of guidance is reflected in how the patients come to the GPs and so that- that varies a lot so patient response.

GP10: Okay overall extremely poor. You know lockdown came too late. Um... track and trace was a disgrace. Um, they opened up too early and too soon. You know the only mitigation is the vaccine rollout which has been very good. In fairness to Matt Hancock, I mean it goes against the grain for me to say this but ordering lots of vaccines was the right call and we are, we are indeed blessed that he saw the film Contagion early on in the pandemic.

Interviewer: Did he say that he saw Contagion?

GP10: He did! Did you not hear? Oh yeah, no, he said- he said he watched the film Contagion and that gave him the idea of ordering up lots of vaccines, have you seen the film Contagion?

Interviewer: Oh yeah I've seen it a few times, and I made a mistake of watching it in the peak of the first lockdown. (*Laughs*) Bit unnerving, it didn't really end in a super satisfactory way, so yeah probably not the best time to watch it! It's interesting to hear that Matt Hancock's used that as his medical source though.

GP10: Yep, absolutely. Well, you know. So the vaccine has been alright, but everything else they've done wrong. You know, and this is reflected in the very high levels of, um- the high death rates.

Interviewer: Um, so a more sensitive question but has Covid had any impact for you personally?

GP10: No. Except for when we had to self-isolate for two weeks, right at the beginning, because like I say my son had a cough and a temperature, but I think he had Covid, I think he just had a cough and a temperature. So, none of us have caught it we've all been very careful, uh, we've not (*laughs*)… We go- I go shopping once a week at seven o'clock in the morning at our local

Tesco, when there's hardly anyone around and you know that's about it, I go to work, I come home, wash all my clothes at 60 degrees with disinfectant and- and you know that's about it. I suppose, yeah, fortunately, we have quite a lot of land, so we can wander around without coming into contact with any- any infectious people.

Interviewer: That must be lovely.

GP10: Yeah it is. I mean I'm not much of an outdoor person myself, but it's quite nice to look out of the window at it, um, and we've got a decent-sized house, so we don't have to (*laughs*)- I don't have to meet any family members if I don't want to. Um, actually I said there's not that much of an impact, there was a huge impact because we got four weeks booked in Australia last summer. We were gonna drive around the whole of Australia. We were gonna do- we were going to fly nonstop to Perth, fly to Adelaide, up to Melbourne, up to Uluru, the red rock, Sydney, we were going not to Cannes, but somewhere on the great barrier reef and then a few days in Singapore, ooh and Sydney yeah. And that was going to be great, and it was all planned out, it was all booked, and all cancelled of course.

Interviewer: I'm very sorry that you missed that.

GP10: I appreciate your- your- your condolences.

Interviewer: I hope it's something to look forward to.

GP10: Well yeah I mean, it's not gonna happen- I don't suppose it could happen this year, maybe next.

Interviewer: Next- next year seems not to... It seems almost realistic next year. This year- I know people are hoping to get away this year, I'm not so sure about that.

GP10: I think it'd be- it wouldn't be prudent. Although I have had both my vaccines!

Interviewer: Congratulations!

GP10: Yeah, because I'm A) old and B) a frontline worker (*laughs*).

Interviewer: Good for you! I'm glad you got those. So how are you doing then. You've had your jobs, done a year of pandemic, how's it going? Are there any changes that you have appreciated?

GP10: Since when?

Interviewer: Since the pandemic began, I guess.

GP10: Well apart from all the ones I've mentioned mention the changes in consultations , I'm not going out, the- I'm not going to the theatre because we're not far from *REDACTED area name*, and so there's the RSC fairly regularly, and quite often used to go to *REDACTED city name* to shows there in fact on the ninth of March, we um, we went to the Tutankhamun exhibition at the Saatchi gallery and so we went down by train with all these- all these people, and the tube with all these people, and then we went to a restaurant with a load of people and in the evening we went to see a play with some upstart crew with David Mitchell…

Interviewer: With a load of people!

GP10: With a load of people, you know, right close up all laughing and clapping and expectorating and coughing and, uh, we got away with it so! (*Laughs*). But yeah, you know, so we do that sort of thing fairly regularly and, of course, well that's all gone out the window.

Interviewer: I would ask, what do you think we can learn from the pandemic?

GP10: Well um we've got to have more, um, we've got to have more! (*Laughs*). More of everything, I mean the- the limitations to the service were made absolutely explicit by the by the pandemic, because, you know the lack of intensive care beds, the lack of, um, lack of everything in the NHS. Um, was made manifest and I'm sure that's one of the reasons we've had such a high death rate is that, um, you know all the- all the, best (*unintelligible*) in the world, there just weren't enough facilities and I'm sure know it could have been worse, it could have been over your keyboard. Or the computer. Um, yeah. I think there was some somebody from the intensive care society or something explaining that you know, we had about nine intensive care beds per 100,000 for the pandemic, the Germans had 33, and the Americans and 34 per 100,000 population, um, and over the past few years, as you do, I've been keeping an eye on the OECD websites which has comparison tables of, um, you know, leading countries and you know we've got fewer doctors, fewer GPs, fewer consultants, fewer scanners, fewer nurses, fewer everything than comparable nations and spend much less on our healthcare, um, so uh, it is- it is clear that, in order to- apart from proper

planning having a proper testing- you know, track and trace system in place, um, which we did have you know, because people, public health doctors would go out and- and find index cases and trace all their, um, all their contacts and isolate them and test them, and you know it was done properly, not done by some bloody call centre run by a bunch of hedge fund managers and people. Um, yeah so you know there's got to be a lot more flexibility and resources within the health service to- to cope with anything like this in future.

Interviewer: Absolutely, I agree, thank you for that response. Yeah, no, completely and- and you know something that other GPs are saying as well, so it's nice to hear...

GP10: A consensus view.

Interviewer: There is- seems to be a consensus view for a few of these things yeah. Um, is there anything that we haven't spoken about today, that is salient to you?

GP10: I think we've covered it all.

Interviewer: Right I'll just stop recording there then.

Recording ends.
