# Supplementary material for: Primary health care during the COVID-19 pandemic: A qualitative exploration of the challenges and changes in practice experienced by GPs and GP trainees
Source: PLoS One. 2023 Feb 9;18(2):e0280733. doi: 10.1371/journal.pone.0280733 (PMC9910752; doi:10.1371/journal.pone.0280733)
Supplement: S1 Data — (ZIP) [file pone.0280733.s005.zip › GP11 Transcript.pdf]

GP11 Transcript

Interviewer: Let's get started, then, to start, could you tell me a little bit about your experience in general practice before the pandemic?

GP11: Oh it's quite lively, (*unintelligible*) my team, uh, we chat, uh, pretty much in the morning, lunchtime, and we have formal informal meetings. Um, yes, we do see patients that- just in our room, uh, most of the time is spent behind the closed doors with the patient, uh, but in between that there was lots of other activities, uh teas in the kitchen... it's all stopped.

Interviewer: And you said, um, you're locum now, is that right?

GP11: Yes.

Interviewer: OK, so do you work in multiple practices?

GP11: No, just for one practice.

Interviewer: Could you tell me about your practice demographic?

GP11: Sorry?

Interviewer: Could you tell me about your practice demographic?

GP11: Uh, I used to work there as a partner, so I know the team, very well there, and you know (*unintelligible*) a family, I sort of decided to step down and start working as a locum.

Interviewer: OK. Um, could you tell me about your demographic, in terms of what your patients- the patient backgrounds?

GP11: Um, it's a mixed one, it's a, uh, semi, uh, rural practice. Just a few miles from the town centre- the **REDACTED city name** town centre so we have mixture of patients, uh, the most of them are working at the hospital- our practice is just behind the hospital, so there's uh, the consultants there, and the doctors, nurses, porters, uh, lots of staff as our patients, and the **REDACTED city name** state is very commutable so there's lots of city workers as well.

Interviewer: Okay.

GP11: At the same time there is deprivation as well, so people on benefits, drugs, alcohol, low mental health problems, so it's a mixed bag, I would say, yeah.

Interviewer: Right, um OK yeah, definitely mixed. Um, could you tell me about your experience of the pandemic professionally?

GP11: Uh... it's sort of the first of all, we don't know what we're doing, uh, instead of- every day, the things are changing we start with something in the morning and all of a sudden, we will receive an email, read through the email, we'll start changing what we're doing, all of a sudden, in the middle of the day, yeah it's quite scary, yeah.

Interviewer: Scary?

GP11: Scary, yeah, and at the same time, we used to have a face-to-face day- uh face-to-face consultation, now we are just doing the telephone now. I initially felt like, uh, I'm just doing a half-job or sort the, uh, three quarter of the work, not really serving the patients fully?

Interviewer: Right.

GP11: But now, uh, we, yeah, I sort of get used to the idea, of uh, yes, telephone consultation if I'm not really feeling comfortable, yes, call them in, and I finish the rest of the consultation.

Interviewer: So how often are you bringing patients in, and are you doing these calls from home or in the practice?

GP11: Practice.

Interviewer: OK. What's the process for bring patients in, do have, um, adequate PPE?

GP11: Uh... yeah according to the government guidelines, yes, we're using the, uh, thin plastic apron, the surgical masks, the gloves, I never felt they sent enough or appropriate PPE, yeah, if the patients are absolutely fine then it's going to be like meeting anybody in the supermarket or outside, I don't have any problem with that, yeah but if the people are coming with the Covid symptoms... I feel that's not really adequate.

Interviewer: Sure.

GP11: Especially I feel sorry for the hospital staff, the nurses, doctors, they work in the Covid area they need to have a quite close contact, and not all the hospitals have enough ventilation, so I feel that, uh, the government failed the healthcare professionals, yeah, and the PPE supply, yeah.

Interviewer: Yeah that's very disappointing.

GP11: Yeah.

Interviewer: Have you- did you feel prepared for the pandemic? It sounds like you weren't in terms of PPE, but otherwise were you prepared?

GP11: Not really but it's quite difficult to prepare somebody for a pandemic because, isn't it, uh, it sort of all, uh, just come across, but I think that all in all, all the, uh, doctors, all the medical community, yeah, adapted really quickly, yeah, and not only the medical team, all the supermarket, all the frontline staff, adapted really well, so yeah, they did an amazing job I'm really proud to be part of that amazing team.

Interviewer: That's great, yeah. No completely, it's been such a quick turnaround, I can't even believe it.

GP11: Yes, exactly, yeah.

Interviewer: Yeah, um, did you have any support in terms of peer support? I know you said that you used to see your colleagues quite frequently, the team staff, how did that change?

GP11: Uh... used to have face-to-face we hardly used, uh Teams, or all the other software for the meetings, other than sending, uh, little emails or letters, or the system message notifications, but now, even if we are in in the practice, we have all the meetings through the Zoom or Microsoft teams. So we hardly, yeah, see each other.

Interviewer: How is that, losing that?

GP11: We do have all the meetings through the, uh, Zoom, or the Microsoft team, uh so the clinical meeting, practice meeting, everything happens, everybody in their own room, all join in the team, so Zoom, have a meeting, yeah.

Interviewer: Yeah.

GP11: And, uh, we used to meet up in the kitchen, uh, the common area, now we have very strict guidance of only one person can be in the kitchen.

Interviewer: That must be tough, going from a very, um, sort of, interactive environment to not interacting at all.

GP11: Yes.

Interviewer: Um, how well informed did you feel about the pandemic and making decisions during it um, in a position of responsibility as a GP on the end of the phone, how did you feel with the guidance you had?

GP11: I think it's quite poor I feel like sometimes patients know the information before we get the email or confirmation.

Interviewer: Really.

GP11: And, uh, we usually get the update from the briefing that, like any general public.

Interviewer: Oh, as in the actual 10 Downing Street?

GP11: Yes, the Downing Street briefing, so that's very scary!

Interviewer: Yeah!

GP11: We get a little bit of detailed information, the following day, yeah, but the main information we are getting from the Downing Street meeting, so as a GP practice we quickly formed a Whatsapp group, and one of the persons makes sure that that they sit down and watches the full Downing Street meeting, and they put a little summary in the Whatsapp group, just to update the people who are missing the Downing Street meeting because they may be working, or maybe, yeah, busy with something else.

Interviewer: Wow, OK, um, that must be a little bit stressful. How do you feel when you're making decisions for your patients then, when you're trying to help them with confusion and so forth?

GP11: I usually try to be honest with them. I know... pretty much same as, or little bit more than you do, so, I might say the completely wrong thing, uh, today, and it might get corrected back to um, just advising based on the information I have, right now.

Interviewer: Right, your understanding of what's....

GP11: Yes, but if anybody challenged me, yeah, I usually take it back and I check.

Both laugh.

GP11: Yes, exactly, I sort of never feel comfortable about reassuring anybody, yeah!

Interviewer: Yeah, um, that's difficult, not being able to reassure somebody when normally this is your expertise, I guess.

GP11: Exactly and uh, I'm not really reassuring over the phone, and, uh, they lost the face-to-face, the body language reassurance, so it would be really hard for the patient, I feel sorry for them.

Interviewer: How have you found the change to telemedicine, to using- is it phone calls or video calls...?

GP11: A mixture of both.

Interviewer: How have you found that change?

GP11: Initially, quite shocking, we did use the telephone and- but that's mainly for triaging or just follow up, but not for the first consultation as a practice, we decided, yes, we use a mixture, especially on our on-call day if the demand is- is so high yeah, you tend to get- triage them, to see whether we need to triage them today, or wait until tomorrow or the day after tomorrow, so we do a little bit of triaging, yeah. But everything on the phone is really a bit of a shock to the system, I- I really struggled to cope with that.

Interviewer: Yeah, yeah I can imagine it's...

(Participant's camera view changes)

Minka: Oh, you've got a proper headset!

GP11: Yes! (*laughs*)

Interviewer: Very professional!

GP11: Yes, it's easy, yeah, I can do all the typing and everything...

Interviewer: Hands free yeah, no, I would love one of those. So, could you tell me about any other changes to GP care, other than telemedicine? Has there been any other changes in your role?

GP11: My role, uh, I was working as locum, yeah, and um, so all of a sudden, the locum work dried up because of the pandemic it all moved to the telephone, the patient was scared to come, yeah, especially with the first peak, so the demand has gone down, so I used to work four sessions, that's cut down to two, pretty much within two, three weeks of the pandemic, and uh, I was doing the cardiology work, the cardiology GP, the cardiology work, that also started drying up because we decided we need to do more with the pandemic rather than doing the cardiology work? So we were just doing the core work unless they have an acute problem. We decided, we're not going to provide any of the service to the patients, uh, no routine service, so that's also cut down, so hours like, pretty much in other words, uh, unemployment or having, uh only one or two sessions a week. At that point, I decided to join the bigger, uh, the Covid-19 111, um, service.

Interviewer: Could you tell me about that?

GP11: So it's, um, a- it's telephone service, so initially it's a bit of a shock, but that's going to be everywhere, even with the community cardiology or GP surgeries I sort of get used to it, um... They have quite good supportive teams, but so they usually support and a lot of forum, lot of guidance, a lot of teaching training, so that's helped me to gain more comfort-confident- or I feel comfortable doing the telephone calls and telephone management.

Interviewer: Great, uh, the transferable skills between the roles, I guess.

GP11: Yeah!

Interviewer: Has it changed your interaction with secondary care? Have you, um, had any shifts in responsibilities between those two sectors?

GP11: Not really, as me personally, I didn't get involved with any of the, uh, secondary care, but my community cardiology nurses stepped down to go to, uh, hospital to help out with the Covid patients.

Interviewer: Right okay.

GP11: So, uh, they have, uh, have a lot of shift around, but as doctors we didn't go for me, the, uh...

Interviewer: Ok. How has it changed your relationship with your patients?

GP11: I would say that most of the patients were really, uh, sort of accommodating they understand that we are in a difficult position, but there are a few people... they didn't accept that, they were thinking that we need to know the in and out of everything.

Interviewer: Yeah, um how do you manage that, when patients are-

GP11: It's quite difficult, we need to explain, so it's, uh, good communication, try to explain that the information we have is just a little bit more than that, and we are changing every day based on the evidence, so instead of keep repeating the same thing... But a few yeah unhappy patients.

Interviewer: Have you had to deal with delayed referral times?

GP11: The referrals has changed, you mean, uh, the hospital referrals, uh, that have quite changed due to the pandemic, uh, because, um, they again cut down the non-medicine referrals and they also increase lots of telephone advice service. So rather than referring the patient, we tend to call the consultant and get advice, then, rather than referring the patient and then they end up waiting for months and months before somebody seems them.

Interviewer: Yeah, yeah that makes sense, actually yeah. Um, slightly more contentious question, what is your opinion of the government response to Covid-19 in terms of public health messages and policies?

GP11: Uh, it's a bit difficult for them as well, I understand.

Interviewer: Yeah

GP11: But I think they should, uh, be a little bit more honest with the public? Because I do remember that a few of the Downing Street meetings they were saying that no, uh, everything is running as normal, the care shouldn't be- get affected, but we are getting, uh, emails, messages from the, uh, RCGP, the hosp- the NHS England, saying that you need to rationalize your referrals rationalize your management, but they were telling something completely opposite to the public? So that's going to get- irritate the patients, uh, saying that the GPs, so all the doctors, are not really looking after them, because of their age, or because of their co-morbidities, but the government is saying no, you should be getting all the care, like, that you usually get.

Interviewer: Yeah, that must be difficult for you as a GP having to explain that to your patients, um-

GP11: Especially as the government is reassuring the public saying that, uh, it's not the case, we're saying that no, that is the case, so it's like we're in the front line getting the fires.

Interviewer: Yeah, sure. Um, how have you, how have you sort mitigated this, have you had patients calling you about guidelines?

GP11: Yeah there a few things like shielding, the government sent out loads of letter, uh, with the, you know- there are a few patients, they are clear-cut, they actually are not shielding, but there are not a lot of people in medicine, like, uh, all parts of medicine, uh, there's a lot of grey areas, there is hardly any black or white, most of the things are grey, we need to make the clinical judgment, so the shielding guidance is not very clear, some people felt, uh, they should be shielding but they didn't receive the letter, but some people will receive the letter, but even going through the nodes and the history, we aren't able to figure out why they will- received the shielding letter, yeah. The government sent out shielding letters to the house address without, putting that- somebody's name. So they get confused, is that the shielding letter for me, or my mom or my partner so that's again uh, that is confusing.

Interviewer: Yeah, no definitely and yeah I've heard a similar thing from other GPs, that shielding is variable.

GP11: Yeah.

Interviewer: This is a more sensitive question but has Covid had any impact for you personally, how are you doing from the last year?

GP11: Uh... like everybody else I'm missing my family, friends, uh, even colleagues, even though I go there every week, I hardly see them, so few other colleagues, uh, I haven't met them, for- I mean face-to-face and sort of having that sort of meetings and chat, uh, I haven't met them for months and months, especially my cardiology colleagues, now we spread out the days, yeah. We used to bump into each other, the one person finishing in the morning, the next person will go in the afternoon, so we'll have a little chat before the other person finishes and then I start, but I haven't seen them, since they, uh... it's nearly a year now.

Interviewer: How is that for you, not having uh, not having people?

GP11: People are social animals, so we're missing that, so I can't wait, uh, to get out, uh, get out of the lockdown but I'm a bit worried about, yes, exactly I don't want to have the third wave, or another peak, and get into trouble again.

Interviewer: Hopefully it happens when it's- when it's right for it to happen, yeah definitely. It's a funny one isn't it, you want to come out of lockdown but you also don't want to yet.

Both laugh.

GP11: Yes exactly, another lockdown, no.

Interviewer: Um, are there any changes which do you think should be carried on into the future post-pandemic and equally are any changes that you haven't liked in the pandemic?

GP11: The complete lockdown, uh, I'm sort of dead against it, because, uh... but, without the complete lockdown, when the, uh, numbers are going uh, rocketing up, and regional lockdowns and the regional restrictions are not working, it is inevitable, so I can understand the rationale behind it. Uh, the few things O sort of like is that, continuing, that- the flexibility of the telephone

video consultation that because that some people really benefit from it, especially for the people who work far away, they don't need to take time off work and they would say 'oh hang around I'll just get- get into a private room' or some area, and they could be able to get the care, but it used to be, they need to take time off, and they need to come and see us, that's going to be a hard day for the, especially if they are working in a distant area, that may be a whole day of leave for them, that they need to take. So that's the good things we need to remain, but it needs to be a happy balance.

Interviewer: A happy balance, yeah no. No definitely that sounds about right yeah! What do you think we can learn from the pandemic?

GP11: Huh! There's lots of things to learn. Yes, so there are a few minority that cause problem, but others are really amazing, the majority of the people really stick to the rules, they obeyed, they were doing everything that they were told to do, and it's really amazing I didn't expect that, especially with the first lockdown, even when the government first announced the lockdown in April were really sensible, they were trying to do what other countries were doing so that- I was really amazed to see. We were trying to see, can we do something, but the patients were saying 'no, there is something going on, uh, we don't want to come and spread the virus to you, or get the virus' so they were really, uh, yes, thinking, the majority did really well.

Interviewer: Thank you that's a great answer. We've talked about quite a bit, um, I feel like we've gone through so many questions, but is there anything we haven't spoken about today that you'd like to talk about anything, that's important to GP care?

GP11: Uh... not really I think we covered pretty much everything.

Interviewer: OK I'll stop the recording there.

Recording ends.
