# Supplementary material for: Primary health care during the COVID-19 pandemic: A qualitative exploration of the challenges and changes in practice experienced by GPs and GP trainees
Source: PLoS One. 2023 Feb 9;18(2):e0280733. doi: 10.1371/journal.pone.0280733 (PMC9910752; doi:10.1371/journal.pone.0280733)
Supplement: S1 Data — (ZIP) [file pone.0280733.s005.zip › GPTr1 Transcript.pdf]

GPTr1 Transcript

Interviewer: Alright, so I'll start the interview then. Um, let me know if you've got any questions. So, could you please tell me a little bit about your experience in GP care and in your practice?

GPTr1: In general, or during the pandemic?

Interviewer: In general, in terms of...uh, you know, your time as a GP and also what your practice demographic is like?

GPTr1: sure. So I guess I work for... a fairly big practice which is actually a kind of group of practices - you've got quite a diverse group of patients so, we have patients in **REDACTED area name**, which is where I predominantly work, um, which is fairly diverse and then we also have a practice in **REDACTED area name** which is, um, got a very high ethnic minority population there... um, so quite varied and quite supportive practice, a training practice, so we've got a lot of trainees. Um... so we're pretty busy, but um... lots of hands-on-deck which I think was very helpful, um... during- to make us quite flexible in the pandemic.

Interviewer: Do you have an estimate of your population size?

GPTr1: Um... I think, so the practice I work at I think of 9000, but I think in total it's about 23,000.

Interviewer: Wow okay!

(*both laugh*)

Interviewer: And so, can you tell me about your experience of the pandemic, um, professionally?

GPTr1: Um, yeah, a time of very rapid change, basically, so I think, um, March last year was when lockdown came and we obviously know this was- we knew stuff was going on around February um... and there were sort of talk about sort of whether we should start to change practice, then I think, um… about whether we should be doing more telephone consultations, but then I think, uh- and we started to sort of limit the number of people that were coming into the surgery.

Interviewer: Yeah.

GPTr1: Um, but I think once the national lockdown came, which was March 23rd, um… I think that was our sort of signal that we really should, well, we interpreted that as we really shouldn't be having any patients face to face, um... so we stopped doing things like blood tests... um... completely, at that point. we stopped doing, you know, sort of routine things like coils and implants and the patient stopped coming into the building, pretty much entirely, for probably... certainly at least a couple of weeks, then a month. We were doing everything by telephone, we were very much deferring things so saying, um, sorry we can't deal with that now... there's a pandemic - I'll ring back in four, six weeks when it's all over.

(both laugh)

Interviewer: A year later.

GPTr1: A year later, exactly, yeah, so we started, we changed things very dramatically, quite suddenly. Um… again- and routes of teleconsultation... fairly quickly we started getting to grips with video consulting, um… so that was quite a quick change and we were able to get pictures of their skin conditions and things like that , um...

Interviewer: How did you find that change?

GPTr1: Um, yeah, I find it quite helpful- I- you know, I tried out video consulting a couple of times, but I found that a lot of patients struggle with technology, with kind of logging into the video system. Um.... some people can do pretty good photos and you know things like moles and rashes, and other patients find that harder and we just don't get good quality pictures. But in general- so most of the time- what I'm doing is, sort of, audio-only calls which people seem to be used to. Most things can be sorted out that way.

Interviewer: Is that through a program or um...

GPTr1: Yeah. Basically I just call people from the- from the phone, using the phoneline.

Interviewer: Cool, OK. You said that, you were, sort of, trying to work out what to before the official pandemic, before the official lockdown began. Um, was there government guidance for that, or did you feel like you were more working it out yourself?

GPTr1: I don't... I don't remember exactly, probably coloured by what's happened, you know, since then… You know, since then I think...

Interviewer: Yeah it was a year ago in fairness.

GPTr1: Yeah, there were, sort of, CCG level guidance coming out. I think, one of the major changes was the development of the, kind of, red-centres or the hot sites?

Interviewer: Yeah?

GPTr1: So there was one the one in **REDACTED city name**. It was run by, um **REDACTED name**, which is an out-of-hours service, so they set that up at the-the main centre in **REDACTED city name**. and that was like uh... basically anybody we thought needed to be examined or, um... yeah so anybody you couldn't deal with over the phone so, even if it wasn't- and if- if they had any covid symptoms, if they had a fever or cough, um... They were referred into that centre... for examinations. So that- that kind of came on and off, it was quite underused, and we were worried that our patients wouldn't want to travel that far?

Interviewer: And in your experience, did they utilize it? Was it, effective or...

GPTr1: That's a good question- uh, I've done some research on that centre as part my academic work, so...

Interviewer: So you're probably quite informed about it, as a...

GPTr1: Yeah... I know that, wasn't, they didn't have very much for them at all, but from our research we in the practice really thought the patients who wouldn't want to travel that far, they'd have to travel past a lot of A&Es to… to use it, but actually if you kind of map where patients, uh... actually came from, and travelled from, to that centre, they- they went miles to see a GP face-to-face, yeah.

Interviewer: It says a lot about them wanting to... about their relationships with GPs I guess, I think. Um, how informed did you feel about the risk of COVID-19 in terms of the personal risk to you, um, as well as your patients.

GPTr1: Um, I think, yeah... I guess I knew as much as anybody, did, you know, at the time? I think my perception throughout is that being young and being white, might- the risk to me was pretty low. Um... and I guess the information about that hasn't really changed much. But I guess now we've got things like long Covid, which, you know, do affect people of my age so...

Interviewer: Yeah, it gets closer to home, every time that a new, um, development happens. Um, could you tell me about the PPE that was available to your practice, if there was any available to you?

GPTr1: I think it's been fairly good, we were... we had a lot of, uh, surgical masks and that- that that was never a supply issue. I remember that at the beginning, being worried that you know, maybe I should ration the use of masks, you know get one and keep it for the whole day, or, you know, never change it between patients. But actually, I was reassured by my practice that we had lots and that we should be using them.

Interviewer: That's good, that's good news as that's been quite a varied experience, I guess between practices and, um... the last question about- in terms of preparation- were you offered any support, or did you feel... supported emotionally, as well as sort of, in terms of your risk.

GPTr1: Yeah, yeah. As a trainee I think I've got a good relationship with my supervisor, so I always felt comfortable.

Interviewer: Okay, that's great. And then, on this topic, how did you feel making decisions with the guidance you had during the pandemic? I can see a lot of responsibility comes down to using your own, sort of- I'm not sure how to phrase it- you end up making a lot of decisions without really knowing the full picture, because obviously we're still working out the full picture of COVID.

GPTr1: Yeah, absolutely, it definitely felt uncomfortable early on and making the transition to telephone calls, was... kind of felt really uncomfortable... not knowing, you know, not feeling like you know the whole story. I think we've gotten more used to that, gotten more comfortable with it over time. And I think my experience as a trainee, um... it's actually been quite- it's been- I mean there's lots of reasons for how I think it's bad for training; we're not really learning, you know, what real- real general practice and I'm glad I've had some, you know, at least a year of experience, before the pandemic. But what was actually different is that this was a completely new disease that nobody knew anything about so... and I knew that the senior partners who'd been training for 30 years didn't know anything about it, and neither did I, um, so it was a bit more levelling for me? Because I could say, look I read this paper I, but- you know, teach- teach you that much it's quite interesting.

Interviewer: Would you say it helped your relationship with- with other staff members in your GP practice?

GPTr1: Yeah definitely, I think it's bought us together more.

Interviewer: Um, in what ways did common practice change for you? So I understand you were in the same role for a year prior. Um, that could be changes in terms of clinical guidelines, or, you know, in your hours that you work.

GPTr1: Um, I guess it hasn't really changed the hours I work or anything like that. I think if I- if I really wanted to and I needed to, I could work from home, but I prefer to go in and have some face to face contact the practice and, obviously, if I need to see patients, I can do that, yeah.

Interviewer: Is that why you've continued to work in the practice?

GPTr1: Yes, yeah.

Interviewer: Okay, all right. Okay, have you had any new guidelines? I know that obviously as distancing is the obvious one, but in terms of how you look after your patients?

GPTr1: Um, yeah so there's the things like you said PPE and distancing and- and how you assess people over the phone, so I know this kind of guidance has come out throughout about how to tele- to assess people on the telephone and on video for Covid. Um so it has, you know…

Interviewer: Has that been helpful?

GPTr1: Yeah! I have found it helpful. And I guess new guidance is coming out about how to manage long COVID, um... so that's always been welcome, this, you know, I- you know, I think it's been some, you know, I know I know how long it normally takes to get a paper out- new research, so actually I think guidance has come pretty quickly. In the grand scheme of things.

Interviewer: You've told me about your interactions with other GP staff. Has it changed any, like, relationships between GP and hospital staff? Or changed- or shifted any responsibilities between the two?

GPTr1: Yeah definitely... it's been interesting. I feel more conscious that if I'm sending a patient into hospital, that might just be through a, you know, a phone consultation. And I feel that... I'm, you know, putting the, you know, I haven't put myself at any risk in assessing that patient on the phone, um... Even if I think there's nothing I can add by seeing the patient face to face, when I send them to A&E, they're obviously- the A&E staff there will be, you know, in contact with a, with potential infectious patient, so I appreciate that they're taking risks that I'm not. At the same time we've done... I feel like we're doing some more secondary care work? Waiting lists are getting longer…

Interviewer: What sort of secondary care work do you mean?

GPTr1: So I essentially mean- kind of managing long term conditions, so that's kind of a longer time between the, um... the outpatient visits. But also, phone, uh- blood tests were off- You know, they now, write to us, to- can you do the blood tests for this patient.

Interviewer: Would you say this has changed your relationship with the patients? If you're seeing- if you have more responsibility- well- new responsibilities, in that case.

GPTr1: Some- I think it's kind of it's been a slow change? It's hard to see, um... So… I think possibly? Um... we are gonna be looking after more things in primary care, that- Well, But- you know, that might not just be COVID related, might be gradual change anyway.

Interviewer: Yeah. You've spoken about telemedicine which sounds like a huge change, um, and also hot hubs. Have you had any experience with NHS 111 as I know, this is another-

GPTr1: Um, no I mean I obviously get the letters through and the referrals, but not much.

Interviewer: Okay, and vaccinations? Have you been involved?

GPTr1: I haven't personally been involved in it, but I'm keen to get involved in it- once I've done my exams, yeah

Interviewer: Okay, is that something you can do once you've progressed through training?

GPTr1: Yeah, yeah, so I mean- I- you know, there's no clinical barrier to me doing it, it's just um, if I do that, then I'm, it's kind of using up my time, that I could be using for clinical training.

Interviewer: Fair enough, one thing at a time when there's a pandemic going on.

(Both laugh)

Interviewer: Um... also have you had any interaction with track and trace so far?

GPTr1: I mean, I have the NHS app, but I've not had any...no- not had any notifications from it.

Interviewer: No referrals from that or anything?

GPTr1: No.

Interviewer: Okay. Um, what is your opinion of the government response to COVID-19?

GPTr1: Um... generally fairly negative. I think they've been slow to make decisions and, uh... you know, slow to uh... You know, lockdown and uh... I know there's been, you know, obviously, problems with PPE contracts and uh... You know, I think there's been a lot of cronyism and um, kind of, you know, government contracts, making money from other people's illness. So yeah, a bit negative I'd say! *(Laughs)*

Interviewer: Fair enough. Do you think they've been effective in controlling the pandemic, I feel like you might have answered my question already...

GPTr1: Um... I mean, I certainly agree with that you know that it's very impressive about the vaccination program, they should be, you know, commended for that. Um… and I think that's... hopefully a big part of it, and they have- they have, you know, bought in regulations about lockdown, albeit just late I think.

Interviewer: Ok, thank you very much. Can I ask you what your experiences of the pandemic have been personally?

GPTr1: Um… yeah so, it's obviously been hugely changed, you know, sort of social life it's pretty much just staying at home, and… you know Zoom conversation with family and friends. Um… yeah it's been a big change and it feels like, um... you know, I think kind of on the boundary of personal and professional, and kind of working academically that you might find this in intercalating, but you, because you're working from home there's no distinction between personal and um… sort of work and it feels like well there's nothing else I can do, you know, that, you know, I can't go to a concert or theatre, I might as well just, you know, just do some more work now.

Interviewer: It's quite hard to, um, yeah like you said, differentiate between the two and when you share one environment for two very different things, um it's a bit disorientating I found personally, um, yeah I've just been at home. But you said you were very going into the practice mostly is that right?

GPTr1: Yeah I'm glad for that actually, it's, it's kind of a privilege that some people don't have in- working from home, change of scenery and actually seeing some other people

Interviewer: Yeah is that something that most people have done at your practice? Do you know what the general experience has been?

GPTr1: Yeah there's one, at least one, member of staff who's shielding, so he works from home, um... A couple of the staff, especially if they've got children at home or, um, parental responsibilities, they have worked from home sometimes, but found that difficult. Um… but generally most people are at the practice.

Interviewer: OK. Would you say that um, GP care is still accessible for patients, because obviously it can improve and also negate accessibility, depending on the type of patient.

GPTr1: Hmm. I think we're still available, we certainly get, um, I certainly hear people complaining about how difficult we are to get hold of. Um… um… and certainly in a couple of patients who've thought oh I didn't realize we could phone you, we thought your doors were closed, but I think-

Interviewer: Not- not coming to care because of…?

GPTr1: Certainly sort of more to the beginning, I mean we did close our doors, essentially for a couple of weeks. But I think most patients got the hang of it, the new system by now yeah.

Interviewer: That's good to hear that it's come around, um... I... Have you taken any protective measures for yourself during the pandemic? That- I mean, that could be looking after yourself mentally or physically, have you found any coping managements, or…?

GPTr1: Uh… not especially no *(laughs).* Just keeping going really, basically.

Interviewer: Yeah. Are there any other changes which you think should be carried on into the future and why and how could they be?

GPTr1: Yeah, um, I think the sort of telemedicine, teleconsulting is probably here to stay. I definitely think there's drawbacks… um… and we don't know about some of the drawback, um.. yet, but I- I know a lot of people, especially younger people who, you know, who generally are at work, you know, they want to you know check a pill, you know contraception. They can just you know, have a phone call in the middle of the day. They don't have to take time off work, come physically face to face to see me, so I think that's a real positive, I think that's here to stay.

Interviewer: Okay. Okay, I guess, that's a form of improved accessibility, then for those patients.

GPTr1: Yeah, yeah.

Interviewer: And, sorry- are there any changes that you think should not be carried forward?

GPTr1: It's tricky, I think we're just we're just adapting all the time.

Interviewer: Yeah of course. No worries. You don't need to answer, I've asked so many questions, you don't have to answer them all! Um… more specifically to your experiences as a trainee, you've spoken a bit about this already, but how do you think the pandemic has influenced your training?

GPTr1: Yeah I think it's been… Uh, I can sympathize with people who worry that we're not getting proper training that we're only seeing limited things. My brother's a GP trainee and he found, he was in a practice that... they essentially put him because he was the sort of youngest, uh, (*laughs)* and the least at risk theoretically, they put him doing all the face to face consultations that had already been triaged by another GP, um, but I think that's quite limited because he's not doing the initial consultation, you

know, he's going to be getting a specific sector, but I think my experience been quite good, I think. I think what we're doing now is kind of how things will be in the future, I think I am training in, you know, in the current system and…

Interviewer: That's a very good point that yeah, you're adapting to something that is probably not just a short-term adaptation at all.

GPTr1: Yeah, and in a way that's kind of nice to be a trainee in that situation, and you know when things have been challenging.

Interviewer: Yeah, and you're learning as everyone else is learning and, as you said, level... levelled in that sense with older GPs, I guess. Thank you so much that's a great answer. Has it informed your specialty choice, are you still interested in general practice?

GPTr1: (*Laughs)* It's a bit late now, but yeah.

Interviewer: No backing out now! And it's a- it's a vague question, but what have you learned from this time, from this year in the pandemic?

GPTr1: I think I mean I mean I learned a lot about Covid-19 itself, so that's, you know, just been constantly looking up the new research that's coming out about it. Um, kind of learned that I and the team that I work with can adapt so that's been very positive. And, you know, a kind of appreciation of you know, people who have worked from home for years, you know, what that's been like for them. And it's, you know, patients are- it's kind of a bit of an all in it together situation, so you know, patients are obviously quite distressed at the moment, but you can kind of, you know, engage with them that, you know, it's wish rubbish everybody right now, so.

Interviewer: Yeah no that's true. Um, is there anything else you'd like to tell me about your time working as a GP during the pandemic that I haven't touched on or?

GPTr1: Probably good.

Interviewer: Um, just a quick thing, you mentioned triaging which I hadn't asked you about. Has there been a shift to triaging in your practice, and could you describe that to me?

GPTr1: Uh… I don't think, no, I don't think, well... I thought- it has changed because you- patients used to ring up and say, I would like an appointment with a GP, whereas now I think they ring up and they talk to the receptionist and say at least vaguely what it's about because, um, before I call the patient, I've got a little like you know, it says pain in legs, or you know, has a cough so I have a bit of an idea of what it's going to be, which is good as a trainee because I can then make sure I've looked up what's…

(Both laugh)

Interviewer: Yeah okay great, well, thank you so much. We've covered quite a lot, but it seems, um, now we're a year in and things are settling into new adaptations but obviously we're still learning as we go. Do you think we've covered everything today, are there any salient issues you would like to raise?

GPTr1: No!

Interviewer: Okay well great, thank you so much for your time then. I'm going to stop the recording.

Recording ends.
