# Supplementary material for: Primary health care during the COVID-19 pandemic: A qualitative exploration of the challenges and changes in practice experienced by GPs and GP trainees
Source: PLoS One. 2023 Feb 9;18(2):e0280733. doi: 10.1371/journal.pone.0280733 (PMC9910752; doi:10.1371/journal.pone.0280733)
Supplement: S1 Data — (ZIP) [file pone.0280733.s005.zip › GPTr2 Transcript.pdf]

GPTr2 Transcript

Interviewer: So, to start, could you tell me a little about your experience in GP care, and the practice that you work in, in general?

GPTr2: Um… so, I've, um, so the practice that I'm in currently, I've been there, um, for… just over a couple of months now.

Interviewer: OK.

GPTr2: And it's, it's a little bit different from where I did my, um… GP rotation in ST1. Because that was, uh… at the beginning of the Covid… you know, lockdown, and this is now, you know, when Covid lockdown has been in place for a while, so a lot of systems that were new initially, have been put in place- have been, you know…. uh, working, tested and, you know, tried and test, and then- and now there's a system that's working, so it's nice to see that.

Interviewer: Great. Could you tell me about your practice a little bit, in terms of the, um, demographics of the population, and how big the population is?

GPTr2: Um… so… we are based, um, right in the centre of *REDACTED city name*. And, um… we have sort of a mixed, um… population, uh, some-some of the population is a little bit deprived, but some are well off as well, it's a mixed, um, demographic. Uh, and, this area in general is, uh, was, a mining, um, area, uh, cold mining was very popular, and uh, a lot of the population used to work in the coal mines, so there's a predominance of, uh, lung problems from that time.

Interviewer: Oh OK.

GPTr2: And uh, the other- the other thing that I would mention is we have, um, a good uh, Eastern European population, because we have, uh, a big Sports Direct hub nearby, and Amazon hub, uh… and there's a lot of Eastern European, um, sort of, um, you know people from East European background working in these places, and they live in this area as well.

Interviewer: OK, alright, that's good to know, thank you. It's great to hear from somebody who has a varied population in their GP because, obviously the experiences are… well, yeah, more likely to be affected by Covid. Um, can you tell me about your experience of the pandemic professionally, um, so within GP care?

GPTr2: Um, so… I was actually doing my GP rotation at the start of the lockdown last year.

Interviewer: Oh right, OK.

GPTr2: That's when, you know, all the different changes were put in place, like for example, that's when we started doing- moved from face-to-face consultations to more, um, telephone triage and doing things over the phone. So initially it was, um, quite a challenge for us, because this is not something we were trained for? You know, going through medical school, we've been taught a certain structure for gathering data from the patient, which is, you know, you take a history and then you do a physical examination, um, whereas, now, all of a sudden, we've had to change- change that and start doing more telephone triage, so we're not seeing the patient, but just hearing them over the phone, so you don't examine them, but you just have to

figure out what's wrong based on, you know, what they're telling you. So we miss out on one important aspect of data gathering, which is your physical examination.

Interviewer: Sure, how- how's that been, that transition?

GPTr2: So, it was quite challenging initially, but I think over time the more we got better at- at doing it, and um, found ways of uh, you know- found ways of making it work, and eventually we did start seeing patients face to face, so that helped, so then we would triage them on the phone, and try and sort out those patients that need physical examination, we still had an opportunity to bring them in and see them.

Interviewer: Great, has triaging been useful?

GPTr2: Uh, definitely, 'cause sometimes, um, if it's something straightforward like renewing a sick note for example, or doing a medication review, then we don't physically need to see them, which we would have done in the past, but now we can just do it over the phone, and it saves us time, it saves the patients time. They don't have to take time off work, um… you know, for something small like that, they can just step out of their workplace, answer the phone, and then go back to doing what they were doing.

Interviewer: Yeah, sounds efficient. Would you say it's still accessible for patients?

GPTr2: Definitely.

Interviewer: OK. Um, do you mind me asking how you're doing them, I know some GPs have different programs that they use it. Is it- is it generally phone call, or do you use, like, messaging services?

GPTr2: So we are mostly doing phone calls, but we do have, um, uh, so for example if I have a morning session of telephone triage, then in the afternoon I will do a face-to-face clinic, kind of, splitting between the two.

Interviewer: OK, great thank you. How prepared did you feel for the pandemic as a GP would you say?

GPTr2: So like I said, this was a, you know obviously came as a surprise to everyone and we obviously had to adapt to a new way of working.

Interviewer: Sure, whilst you were training.

GPTr2: Yes, whilst we were training. So, um, I don't think we were prepared for it, but we kind of just had to get on with it, and- and adapt, you know, with the situation. So while we were, starting to do this, some- you know, reading material, or learning material, was coming up on how to do, um, better telephone consultations, or video consultations, uh, so, that helped because- you know, sort of doing these little e-learning tutorials and things?

Interviewer: Oh right, where- where did that guidance come from?

GPTr2: Um, so different places, so, um, the RCGP was, uh, was doing some work… in that direction, you know, and they were coming out with, um, e-learning modules that we could do, to help. So obviously that with- doing that, and also, the actual experience of doing it, um… first hand, uh, so that proved useful.

Interviewer: Did you find that, uh- did you feel you were covered in terms of um… physical support as in PPE, and also emotional support, during the pandemic?

GPTr2: Um… so initially we did have a problem with uh, PPE, but I think it was more of a problem in hospitals than with GPs, because you weren't seeing as many patients face-to-face anyway, so kind of used our PPE sparingly.

Interviewer: Right.

GPTr2: Um, emotionally… the practice where I was at, the um, you know the staff everybody was really, you know, came together as a team, you know supporting each other, so I think that was- that was really good.

Interviewer: Ok great, that's nice to hear. So would you say- do you think it strengthened your team going forwards?

GPTr2: Yes definitely. The only thing, uh… the only thing is we rotate every four months, so you kind of, uh… you know, go through an experience with one team, you work well together, you get to know each other, and then, and then four months later you have to rotate somewhere else and start working with a completely different team.

Interviewer: Have you been rotating between practices, then, for your training?

GPTr2: Yeah so in my ST1 I was at a different practice, and then I went- I went back to hospital, um… and now I'm back at GP, yeah.

Interviewer: OK great. How well informed did you feel your patients were about the pandemic, um, I'm sure you would have to had to inform them of new safety procedures and so forth, did you feel that they knew what was going on?

GPTr2: So… I think, um, I think so, because you know everybody was quite aware of what was happening in the news, so I think all along people were quite informed of what was going on, from what I could see.

Interviewer: OK. Well that's what I wanna know anyway, is what you observed, because that's what's useful! When all the voices come together hopefully we'll get a picture of how it was experienced. Um, how did you feel making decisions in the pandemic, considering, I'm sure, it was an evolving situation with lots of new information coming in. How did you manage that, with sort of, differing advice coming in?

GPTr2: So… um… from a trainee perspective, um, we have a lot of support from our, you know, trainers and supervisors.

Interviewer: Oh, OK.

GPTr2: So I was always kind of made use of that opportunity, if there was anything I wasn't sure of, I would speak to somebody a bit more senior, and get advice from them, see if they had any advice on doing things differently, from what I was doing, and take that on board.

Interviewer: Cool, OK, thank you that's a good answer. Um, in what ways did you find that common practice did change for you, um, in your year. I can understand that it was quite a unique experience for you in that you were training, as you were learning about a very new thing that everyone else was learning about at the same time. But, um, how was, you know, clinical guidance and so forth changing as you did that?

GPTr2: So clinical guidance. I would say, more emphasis on… taking the history, looking for… what we call red flags. So more emphasis on that, rather than, you know, seeing the patient and picking up on, sort of, signs.

Interviewer: Hmm OK.

GPTr2: So that's definitely changed.

Interviewer: Thank you, I haven't actually heard much about that before, so that's good to know! Thank you. That makes sense, I guess, when you've got to field people by the phone. Um, did it change your working lifestyle, as in your hours or the staff that you interacted with?

GPTr2: Um… so not, so my hours were still the same, still went to work at the same time, came back home at the same time, um… but in terms of interaction with staff, um… I don't think that changed very much, because we would still… you know, you would still meet people in the corridor, you know, in the kitchen when you're making yourself a hot drink, or in reception, um… so I don't think that changed very much, at least not in, not in the GP- the GP setting, but in hospital it was, uh, a bit more different, because people were, uh, you know trying to distance themselves, um… less, uh, you know, meetings and things like that, in hospital.

Interviewer: Did the, um, interaction between GP and hospital staff change in your opinion? Or um, did any responsibilities transfer between the sectors?

GPTr2: Uh… we definitely had, you know, a little bit more responsibility, because you know, a lot of the specialties were postponed all their appointments, or cancelled their appointments, especially in the beginning. So all, uh surgical fields, they had cancelled all their work, so all these patients were left in the community, and they were coming to us, saying, you know, my appointments been cancelled, I'm still having this problem, what can I do in the meantime? So we were trying to, uh, cope with that, trying to get advice from specialists, you know writing letters to them, trying to see what else we can do in the meantime while we wait for the operation, or whatever they were waiting for.

Interviewer: Were you able to get advice, regarding-

GPTr2: Yes. Because I think the specialists were, uh- they were not as busy in certain specialties, they were not as busy at the beginning so they were quite accessible.

Interviewer: And is that due to the referral number decreasing?

GPTr2: Yeah

Interviewer: OK. Um… that's good to know, thank you. I had a question to ask about that- in terms of your patients, did it change your relationship with your patients, because I guess you're taking on more responsibility in terms of their holistic care, um, did you notice any change?

GPTr2: Um… so I think it does a mixed sort of, uh, response 8from the patients, some often were obviously appreciating the work we were putting in, and they were always, uh, you know, thanking us, the NHS in general, uh… whereas others were still kind of demanding, and you know saying… they were upset because you know their procedure had been cancelled so… they…. the next person they can talk to is their GP so sometimes I found that they were taking it out on us.

Interviewer: That must've been quite difficult.

GPTr2: It was yeah, especially when our hands are tied, because we can't…

Interviewer: Sure, it was out of your power.

GPTr2: Exactly, we can't push specialties to, um, to bring forward their appointments or do a procedure that they're not doing.

Interviewer: No, of course, how did you manage that when patients were coming to you with their frustrations?

GPTr2: Um… just uh, just doing our best to explain we can't do anything at the moment, we just have to wait and see what happens, you know, when- when the services resume.

Interviewer: And is it starting to go back to normal now? In terms of… um….

GPTr2: It did, yeah, so between the first and second lockdown, a lot of um services had resumed, so I think a lot of backlog was starting to clear up, and then we went into the second lockdown, and suddenly services shut down, but I think by this time people were, you know, kind of coming to terms with it, and were not, um, you know, as upset that, you know, something was cancelled.

Interviewer: OK, that's a good change, I guess. OK great, thank you, the questions I had to ask about practice are… you've touched on most of the things I was interested in, in terms of telemedicine, um, triaging… have you had any interactions with NHS 111, I know some responsibilities have been shifted because of them, has this had any effects on GP care?

GPTr2: Um… so initially, I think, uh, NHS111 was overwhelmed, because they were the ones dealing with all the Covid patients, and anyone that came to us, we were directing them to 111. But then over time, that responsibility has kind of been shared between us, so, now, it's come to the stage where we can comfortably give advice on Covid symptoms, and we know what to do.

Interviewer: OK, great. What about track and trace, I mean, would you… what were your views on track and trace?

GPTr2: So track and trace, my personal view was that it is been… run by… people who are not, not- fully trained to do their jobs, and they kind of just have to follow, you know, mechanical, like questionnaire in the system, and if they have to think outside the box, or deal with a situation that's a bit unusual, they- they can't cope with that, they get stuck, they have to involve you know, their managers, and we just go round and round in circles. So I'll give you my personal example, I, uh, tested positive for Covid, around Christmas, new year's time, and I did my isolation, and went back to work, and at work this was when we'd started doing weekly covid tests, for all the staff, so, a few weeks later my test came aback positive again, now this was a remnant of my previous infection, because it was only then that we discovered once you'd had covid, you shouldn't do another test for at least, you know, 8-9 weeks, because it will still be positive, but uh track and trace were constantly calling me ringing me, telling me I need to self-isolate.

Interviewer: Really

GPTr2: Yeah. But then when I called them and explained the situation they said, we'll look into it, but you need to self-isolate, and then we were having discussions with you know NHS England, with the, uh, infectious disease teams, and they were saying no, you can carry on working, and then the person on the phone, on track and trace, because they're not, you know, they're not aware of what to- to do in this situation, they'd just say 'no you need to isolate, because your test was positive).

Interviewer: Right.

GPTr2: It took a few days to, sort of, sort that out, but eventually we did get, uh, the correct guidance and I was able to go back to work.

Interviewer: Did you find the testing experience OK?

GPTr2: Yeah, so I think the testing in my area was pretty straight forward. Um, you ring, or you book the appointment, online, and, uh, you- you go the centre, you don't have to wait very long, and you get the test done straight away.

Interviewer: Great! Not great that they tried to make you isolate for two months.

(*Both laugh).*

Interviewer: So you had your isolation period, I'm assuming you worked from home during that period?

GPTr2: No, so, um… I didn't have to work from home for the days that I was self-isolating.

Interviewer: Oh, lovely! Did you work-

GPTr2: Because I think for us it's a little more complicated, because for staff that are permanently based at the GP practice, they… they can get, you know, a computer from work, laptop from work, that they can go home with and link to the system and have access to patient records and things.

Interviewer: Yeah.

GPTr2: I think for… uh, GP trainees who are only there for a few months, it's a bit difficult for them to give- give you know, me a computer and access from home.

Interviewer: OK, that's good to know thank you, because obviously it's been very mixed, you know, some people have worked from home entirely and some haven't, but that explains some of that difference. Ok, so then I assume you've worked in the practice that whole time then. OK, um, in more… away from GP care, but also involving GP care, um, what is your opinion of the government response to Covid-19? This is a big question (*laughs*) so only say what you're comfortable saying, but…

GPTr2: OK. So I think the government did, um, I mean people have mixed views but this is my view, the government has done their best, I think, given that- that this is a you know totally new… new situation, one that we've never faced before, and nobody really knows the right way of managing it, so, I think given the circumstances, the government, the government did the best that they could. I think in sight we can always criticise, we can always say, I think the government should have done this instead of the other, but, when you're in that situation for the first time it's very difficult to, um, you know decide what's- what the right way of doing things.

Interviewer: Sure, it was a very new situation, and no one knew what was going on completely. Yeah no, that's a reasonable answer.

GPTr2: And there's a lot of factors coming into play that we still don't' understand, because, uh… you know certain countries have not had strict measures like ours, but have still, um, managed to not have the effects we've had, you know, from Covid.

Interviewer: Hmm. Would you say that, um, because obviously I know patients would have been coming to you if they were confused, would you say your patients were, uh, adequately informed by the government about what was going on, or did you receive direct guidance?

GPTr2: Most of the time they were quite aware, because everybody was, you know, watching the news, and, the government's campaigns were quite clear, and very simple to understand the rules, whether people followed them or not, that was a different issue, but everybody knew what they were supposed to be doing.

Interviewer: They knew if they were following them or not? Um, what would you say was your personal experience of the pandemic. And feel free to not answer that, or answer as much as you want, but… did you feel like you needed safeguarding for yourself, are you an at-risk person, did it affect your life, or…

GPTr2: So I'm the kind of person that just kind of gets on with things, you know, with uh, we're faced with difficult situations and you just kind of adapt and move on. You know, being a medical student, you go through difficult exams and different pressures and sort of, um, you know because I'm from Kenya, I've been bought up in a different way, to really appreciate you know, what we have, so I kind of, um, just got on with things even though the situation was difficult, but I think, because it's gone on for a long time, it kind of has taken a toll on me, if I'm honest.

Interviewer: That's fair, yeah.

GPTr2: And during Covid, we've just had our baby.

Interviewer: Congratulations! That's so exciting!

GPTr2: Yeah, thank you, he's 9 months now, but he was born during lockdown.

Interviewer: A proper lockdown baby, yeah.

GPTr2: Yeah! Lockdown baby. So that was something that we, uh, had to deal with.

Interviewer: Was that manageable?

GPTr2: Yes we did manage, we've had our ups and downs I think, things are… things are getting better now.

Interviewer: OK, I'm glad to hear that, and congratulations again on your baby!

GPTr2: Thank you.

(Both laugh)

Interviewer: Um, did you have to take any protective measures for yourself, um, physically, or- or just looking after your mental health in terms of getting through?

GPTr2: Um, I didn't necessarily take any extra measures… I just followed what the government was saying. In terms of PPE, I was wearing what uh, what the guidance said.

Interviewer: Yeah I understand.

GPTr2: And um, and um yeah?

Interviewer: OK! In terms of the future of general practice, are there any changes which you think can be carried forwards, or should be carried forwards, and equally are there any that you think should not be carried forwards?

GPTr2: So, uh… so the main, sort of shift, I would say, is um towards more telephone triage, and more telephone consultations, and… and I think this is the way forwards, doing remote consultations is the way forwards, and I think that is something we should carry on doing, even after the Covid pandemic, because it's a very efficient way of working, I find, um…. but we also have the opportunity to bring, and see patients face to face, if we want to?

Interviewer: Sure, so you don't lose patient contact completely.

GPTr2: Exactly

Interviewer: Because I imagine that wouldn't be easy for you or the patients to not see people when you're working.

GPTr2: Yeah.

Interviewer: At the moment, how much of your consultations would you say are telephone-based, if you could estimate?

GPTr2: So my… on my personal rota, I would say… about 60% telephone? And about 40% face to face.

Interviewer: OK, that's more face to face that I expected, but I guess it is a year in.

GPTr2: Yeah, and it depends, some practices are doing it, uh… you know 50-50, and some-some trainees, for example at my practice, the more junior trainees, have more face-to-face contact. So ST1s are doing more face-to-face than I am, whereas I have certain days where I'll do entirely telephone based.

Interviewer: Why is that, that the ST1s are doing more face-to-face, is that because they're younger?

GPTr2: I think because of the experience, um, so I'm not sure what the reasoning was, from the management of the practice, but my feeling is because uh… you know, they, as an ST2 you're expected to have a little better experience, so you can work things out over the phone.

Interviewer: Right, the red flags and everything that you spoke about. So as a trainee, a few trainee questions and then- these are my final questions really, but, um, how do you think the pandemic has influenced your training?

GPTr2: Um… so… luckily, you know, last year, during the uh, you know during the height of the pandemic, exams were cancelled, but, for me, I was in ST1 and we don't really have to do any exams in ST1?

Interviewer: Oh, OK, right.

GPTr2: So I wasn't, from a trainee point of view, I didn't, I haven't missed any exams or anything, as such. We did have to cancel all our, um… so we have a teaching session once a week, every Tuesday afternoon. Initially that was cancelled completely, just… and we continued to see patients during that time, during our educational time, um… then… the… you know, the college decided that education shouldn't be stopped, and that it needs to be carried on, so then we restarted, you know our weekly training, but everything was done by like, you know, a Zoom, or a Microsoft Teams meeting, uh… and that's how, that's how we're doing our teaching at the moment. So initially we did miss out on the teaching, but now we're back to doing it.

Interviewer: OK. Do you think that will have had an effect, or do you think it's been recovered?

GPTr2: Uh… Initially it was difficult, which I imagine a lot of people have faced, problems with, you know, IT, things like 'can you see the slides?' 'can you hear me, can everybody hear me?'

Interviewer: As a student, I can relate to that, yeah.

GPTr2: Exactly, but, uh, I think now, people have learnt how to use Microsoft Teams, and you know, Zoom and how to do their presentations online, and speak to an audience, you know… remotely, and it's starting to work out now.

Interviewer: OK, great! Has it informed your speciality choice, or are you, nothing changed…

GPTr2: So initially, I did, uh, doubt my choice doing GP training, especially at the beginning, because I was really missing out on that, uh… you know human interaction, because I would go to work in the morning, say hello to whoever I saw on the way, and get into my you know consultation room, or the room that I was allocated, and I'd sit there at the end of the telephone, talking to patients, trying to sort out their problems, um, and I'd come out at lunch time, I might, you know, meet one or two people, go back, spend the whole afternoon there on my computer, and at the end of the day go back home, so I was missing out on that, you know, that human interaction.

Interviewer: Yeah, I can imagine it felt a bit lonely.

GPTr2: Yeah, I felt a bit lonely, as opposed to… you know, before that I was in a hospital, I've only done hospital jobs, where you're constantly working with a team.

Interviewer: Yeah, you're used to a very different kind of interaction level, to a pandemic job.

GPTr2: Yeah you were working with staff that were more senior to you, junior to you, you know your friends, so I, I missed that, and I think… was I better off working in hospital? So initially I did have those sort of questions. I think now, I've uh… sort of come to terms with it, and I'm not thinking- I'm not having mixed thoughts anymore.

Interviewer: OK, are you happy with your choice, do you mind me asking?

GPTr2: I think so, yeah.

Interviewer: Think so (*both laugh*). OK, great, um, well I'd just like to ask, is there anything you've learnt from your time in the pandemic? I'm sure there's a lot you've learnt, but anything you could say in particualr?

GPTr2: Um, so I think I've touched on most issues… but I think, um… the main thing that I would tell- I've learnt as a skill is, you know, remote consultation, yeah I can't think of anything else that would've changed if we didn't have the pandemic.

Interviewer: Yeah, I guess it's just caused a natural change to happen very, very quickly. Um, do you think that there's anything we've missed out, during this interview? Or are there any other issues or topics you would like to raise?

GPTr2: No I can't think of anything else.

Interviewer: Ok alright.
Recording ends.
