# Supplementary material for: Primary health care during the COVID-19 pandemic: A qualitative exploration of the challenges and changes in practice experienced by GPs and GP trainees
Source: PLoS One. 2023 Feb 9;18(2):e0280733. doi: 10.1371/journal.pone.0280733 (PMC9910752; doi:10.1371/journal.pone.0280733)
Supplement: S1 Data — (ZIP) [file pone.0280733.s005.zip › GPTr3 Transcript.pdf]

GPTr3 Transcript

Interviewer: To start, could you tell me a little bit about experience in GP care in general, and about your practice?

GPTr3: So the practice is a small… kind of… um… village-based practice, so in the countryside, uh, demographics-wise, um... tends to be middle, upper-middle class, so that kind of influences what kind of conditions you see, and also, um… a lot of people wanting private care, presenting quite early, a lot of health anxieties, um, and… that sort of thing, tends to be people who, um… kind of wealthier people you might live by themselves, have children, rather than big families living together? Um… so that's the area, um, what was the other question, sorry?

Interviewer: Can you tell me about your experience in GP care?

GPTr3: So… prior to this I was in a different area, I was in **REDACTED city name*, definitely demographics slightly different, but it was kind of just standard in terms of you just saw everyone face to face, there was very few, I don't remember doing a telephone appointments as a trainee at all.

Interviewer: Ok

GPTr3: And… obviously you have the time pressures of people being in reception, waiting for you, but you got to know people a lot quicker? Uh, that's quite important in general practice, because, my trainer does have the insight of knowing what the patients like, what the family's like, in terms of you know, what do they do for a job, um, what kind of social support, and also what they tend to be like in terms of their consulting or their narrative when they come. So some… are very anxious, but I don't know that, because you have to go through their records and see actually they keep presenting with very vague symptoms continually, seeing a lot specialists, um… that's- that's where I'm out now, tends to be like, and because we're doing everything by telephone, I can't get those insights of what, you know, people tend to be like… I can't remember faces if they consult again… you're kind of like, 'ooh what did I see them about?' – you just see them as that one, kind of, symptoms that you dealt with before (*unintelligible*), that you've dealt with before, which is a bit of a shame.

Interviewer: I was going to ask, how has that impacted, or has that changed your care, and your relationship with your patients?

GPTr3: Um… it affects your risk management in some ways, because you don't know whether this person is… it's not to say that people who keep presenting aren't genuine, but it-it affects how you, um, not pacify them, but in terms of 'how do you say to them, you know- you know, let's look at this a bit more deeply… do you think that- the- anxiety or depression is coming into play

with this, or, do you feel that-‘ they might mention work, I don’t go into it, but my trainer knows that, you know, they recently lost their business or something like that. I don’t have any of those insights so I can’t bring that into the consultation, or don’t think to ask about it, because you don’t know if it’s going to be important. So a lot of them, you end up just doing what you’d do for any patient! It might be a referral, or treatment, which you don’t know really, is it actually necessary, should we delve into this some other way before we do that… So that’s kind of, for me, it’s risk management that’s difficult. And especially not seeing people in person, um, you’re, you know, you’re trying to diagnose on the phone, um… especially elderly patients, they really struggle with the videos… um, we don’t do many video consultations because people don’t seem to pick up the link, or answer on time, you know, calling them multiple times especially for elderly people…. I don’t think I’ve seen anyone on video, so you’re not getting that kind of visual input as well- do they look unwell, what’s their breathing like, you have to guess over the phone, you know, what do they sound like, what do I picture them to be. Um… yeah…

Interviewer: Right, OK. Have you received much in the way of guidance about the change to telemedicine?

GPTr3: It came quite… after the pandemic, like there’s webinars coming out now or things, it’s like, well, we’ve been doing this for 6 months, it’s a bit late now.

Interviewer: Nealy a year now!

GPTr3: Yeah exactly. The only thing I have, and it wasn’t- it was before the pandemic, we have to do out of hours anyway, and they’d done, um, a session on telephone triage before we’d started doing it, um, so that was useful, you didn’t know you were gonna have a pandemic later and you were gonna be using it all the time, um, but that was quite useful. We don’t do triage, myself, the on-call doctor will do that, but it’s certainly useful for your consultations, your structure, how to document things as well.

Interviewer: OK, thank you. Um, did you feel informed about the risk about Covid-19, um, going into it, and also about how to reduce your own risk?

GPTr3: This is so contentious, what I’m going to say!

Both laugh

GPTr3: Obviously now, (*unintelligible*) have recommendations, then the hospitals, kind of, or the government had their recommendations, the problem hasn't been is that I feel, I think some of the doctors feel, the evidence has been so conflicting, so when I was originally in hospitals for ST2, I was on the acute medicine ward, and we had a covid ward and a non-covid ward, because it started out- initially we had sort of the covid ward for definite, and then everyone else belonged in the other ward. So no, I didn't feel protected. Initially- (*unintelligible*)

Wifi break

Interviewer: Sorry I lost you, can you hear me now?

GPTr3: Yeah I can hear you. Um, is that alright? Yeah, so on the non-Covid ward we were having patients who were testing- would develop symptoms, would have a swab, and then if positive move onto the positive ward. We weren't allowed to use the facemasks, because they were non-Covid officially, um, we felt at risk. Then they changed their minds- well we started using stuff anyway, because we thought (*unintelligible*), then they changed their minds and said you wear it everywhere, and that became throughout the hospital, much later, like, a month or two months into the epidemic, but it just felt like we were playing- the government was playing catch-up quite behind what we were seeing on the ground? The other things was, I'm pretty sure, that the facemasks… the normal F2 ones… they don't protect you, they protect everyone else around you, so we have patients who don't wear the facemasks on the ward, but essentially, you know you're wearing it but you're not infected- So when I was in the hospital, I had symptoms at least twice, separate occasions, maybe a week, two weeks apart, um… and at that time, it was when, I mean this is when things have changed, we're finally playing catch up – because I didn't have any official symptoms, you know a cough or temperature, I wasn't supposed to isolate, so I ended up having to go back to work, like, 2 days later, then later on this subset of symptoms came along, you know, up to like, a few months down the line? So, in general practice I feel way more protected, because the partners make the decisions for themselves, it's not coming from much higher up. So they- they have decided we're not seeing anyone with a sore throat, like- we're not looking in anyone's throats, you can come if you have a sore throat but we're not gonna look in the throat. They seem to think that if you've got a covid-positive patient, which we don't see, we're also- I clarified this yesterday, we're not seeing them full stop, um, if they did, they were supposed to have the FFP3 masks, which isn't actually technically cases, well it is in hospitals, just type 2s unless you- you know, unless in certain circumstances you use the FFP3, so I feel like in general practice there is more scope to protect yourself better than what you would do in the rest of the NHS, um, I don't know about availability in terms of – because I think initially the practice had to, um, source their own masks, aprons, everything, to… until the stores caught up, but the other thing that really kind of us made us feel at risk was the fact that um, when the masks were coming through, they'd come in the boxes, first of all they were all made in China which is a bit dubious but that's not the point, the point was the expiry dates were all stamped over multiple times? So

they should have expired 2 years ago, there'd be a sticky label where somebody had re-dated to say it had another year, then another stick label on top, so you'd just be peeling off these sticky labels thinking you know this has been in some warehouse for years, and now they're just saying because of the pandemic, you can use them. And the nurses were upset, because normally if you have out of date stuff it has to go straight in the bin, and if it was found on the ward you'd get in trouble, but because it's a pandemic and the government has no other resources, we're having to- we're now just made to use it, and it's kind of like 'is this stuff safe?'. I personally didn't see any of this, but some of the staff were seeing some of the stuff had gone mouldy, and it had still been distributed, so it was like uh- it didn't seem to have any consequences, and my whole view of the NHS has changed completely.

Interviewer: Yeah. Was this in hospital wards, or was that in GP practice?

GPTr3: That was in the hospital. Because in the GP practices we had to source our own stuff initially, everything we were getting was newer, donations from, um, the patients who like, owned stuff anyways, as part of what they did, uh, or money to fund stuff. I bought my own FFP3 mask, and this was another thing- I bought my own, because, um, the- the fit testing, I kept failing the fit-testing for one at the hospital for one, and um, I was told I couldn't use it, because it was a reusable one, which is what they in intensive care were using, but they didn't provide me with any support to- to show me how to clean it, I knew how to clean it because the instructions said so, but it was just, 'you have to use our type-2 masks'. I could see where they were coming from, in terms of just, you know, that's their guidance or whatever, but it's a higher level of protection, and they always say it being reusable isn't, so why is ITU using this, 'cause they used to have the hoods, and things like that, so yeah, it was just a bit all over the place, and it just wasn't very pleasant. I find GPs much better.

Interviewer: OK.

Wifi cuts

GPTr3: Where if you don't see a patient (*Wifi cuts - unintelligible)* they go into the room, it's booked for your certain time, um and then you clean down the room afterwards, and sort of it's a one-way system, it's all very organised otherwise.

Interviewer: Great OK, so how well informed did you feel your patients were about the pandemic, in terms of wearing masks and why they couldn't get their, um-

Wifi cuts – switch to telephone call

Interviewer: OK, um, I can’t remember where were, but I have-

GPTr3: You said something about patients and masks.

Interviewer: Oh yeah, did the patients understand what was going on when they couldn’t get consultations, and had to follow new rules?

GPTr3: In the main they’ve been really… good. Some have, the problem has come since we had a patient... did she die? I think she died, this lady. And the- prior to that, the patient hadn’t actually asked us for face to faces, but they seem to think we’ll just jump and come out- they seem to think we’ll come on our home visits and (*unintelligible)* but because it’s not their usual doctor, and it’s not the same, and this that and the other, so they called, um, to say that she was unwell, and the GP had assessed the situation from what the reception had told them, and said she needed to call an ambulance, which she did, went into hospital, and later she died, but they died in a kind of- the husband was kind of like ‘oh, you know Covid has been an excuse for you not see us, and not to… access and all sort of other stuff’, and it’s like, it’s not, there’s a pandemic on, and an ambulance was called which is what’s appropriate for that situation, she needed to be in hospital, so I think some people when there’s adverse outcomes, they are kind of thinking, you know, how has Covid affected things, and could it have been, and you know, it’s not optimal. The other thing we’ve had is patients putting off consulting because there’s a pandemic, they keep thinking that we’re too busy, and it’s like we’re busy, but we’re still here for you?

Interviewer: Right.

GPTr3: So I had a lady yesterday, she hasn’t had a smear for 2 years, because it was last in 2019 or something, she was supposed to have it last year, um… was it a smear? She was having something… oh no, it was her thyroid monitoring!

Interviewer: OK.

GPTr3: She- She’d completely missed the date, and had no intention of booking in, so I was speaking to her about something else, and I said to her you know, you need to come for this, obviously, you know, and she said ‘oh no, I thought it’s a pandemic, I’ll just leave it!’. And also, people are a bit fearful of coming anywhere near anyone else, you know, even if it’s the practice, they don’t understand that we’ve got measure in place, you know their hand gels everywhere, everyone’s in masks and their aprons, they just think ‘Oh I’ll just wait until it all blows over’, which could be forever.

Interviewer: Hmm.

GPTr3: Um, a lot of them have been very well-receiving of the vaccination programme? Um, really, super keen, uh… to get back to you know (*unintelligible*) as soon as possible, which is good. But in the main, people have been really understanding and accepting of things.

Interviewer: That's great to hear. Um, how have you felt, um, sort of making decisions for patients with what sounds like, kind of, quite limited guidance yourself?

GPTr3: So… I wouldn't say the guidance is limited, what has been difficult is accessing, uh, appointments at the hospital and things like that, so… If I give an example, it's just the waiting times that's ridiculously long, if you're not doing a 2 week wait, then you can forget being seen anywhere near the next three to four months.

Interviewer: Oh dear.

GPTr3: And in specialities, you know dermatology, neurology… I think dermatology is one of the ones we need input with, in terms of you know, if we're struggling- we're struggling, but the wait is just ridiculous. If it's a two week wait, they're getting seen in three weeks, usually, bit longer than it should be, but at least they're being seen. If it's anything else, it's like should I even bother referring, because they might not even get seen, until like later this year? I had one patient, um, who, it's very sad actually because of these things that they've got are really impacting their work, so I had one patient, a builder I think it is? Who runs his own company, was having intense back pain, he…had, um, was struggling with all the pain medications we'd given him. So I contacted the pain clinic, to see, um you know, if he they could do a spinal for him, because he'd seen a private surgeon, just to discuss what- you know, what was an option, and they'd told him this could be an option, but the price was just way too much for him, and the wait for it was months, and that was, you know, for pre-existing people, cancelled or whatever, he was gonna come, you know, pretty much at the back of the queue, and you know his business was at risk because he couldn't go to things, and it's just very sad to see people in those kind of positions, because I can't really upgrade the referral any further, because part of the problem is, in the hospital bed space, you know, if thing are converted to covid wards, you can't put a patient in there, there just isn't enough space, so it is sad.

Interviewer: How do you, um, how do you sort of manage those situations, when you have… it sounds quite difficult.

GPTr3: With difficulty. So, you do the referral, and… you tell them it will take time, and they're usually accepting about that. Then they'll come back and say it's taking too long doctor, blah blah, so if things have changed, and things have got worse, I'll write and say, you know, can things be expediated. I don't know in what percentage of cases that actually happens, but otherwise you have to just be honest with them, and say, you know, it's gonna take time, if you want to go private, go private, if you can't- you're just gonna have to wait realistically, unfortunately.

Interviewer: It must be really tough.

GPTr3: Yeah. And if it's desperate, then just go to A&E, 'cause we can't do anything else.

Interviewer: Ok. Um, could you tell me about any, uh… sort of changes in your role as a GP that you've noticed? Um, if you've had to take on any new roles, or sort of, changes in responsibilities between you and secondary care…

GPTr3: Um… obviously the vaccination programme

Interviewer: OK, have you been involved in that?

GPTr3: Um, I wasn't… at the moment. I was sort of asked, but not put on the rota, um, so , in terms of- we've had the nurses, the doctors, the pharmacists, all helping at the vaccination centre, but… we've been um… I've been involved in the kind of planning for that, and also the flu vaccine? Because obviously we had the flu run, and then a few weeks later we had to do all the Covid vaccinations, so it was kind of coordinating when are you going to do this, um… and it is- I did (*unintelligible*).

Interviewer: Oh right.

GPTr3: We changed the system for booking, so now we have a triage system we didn't have before. I think it's an improvement, because, um, a doctor can assess, you know- well, to an extent actually, I feel like most of the people who, um, get booked in, didn't need phone appointments, and we've had a meeting yesterday, and they've realised that, um, I think, to some extent, the partners are kind of pushing them to be seen on the day, so we've just seen them and be done with it.

Interviewer: Right.

GPTr3: There are a few people you get a routine appointment following that, um, but, you know you can have a little pimple on your face and get a same-day appointment! It's supposed to be an emergency appointment! The other thing is, um, in terms of, um, I think the added burden of multiple consultations, because a lot of the time stuff that should've been face to face, for example, a rash with no photo, even though we've asked for a photo, they haven't sent it, so you don't know what you're working with! So you're wasting time on the phone, like can anyone get another photo, they're like no, so then you're booking another appointment just to look at the rash, so there's been a burden of that of multiple appointments for things, or things that you couldn't- you dealt with on the phone initially but didn't resolve, so a lot of people originally if they had like earache, would just get amoxicillin, treat it as an infection, and if it doesn't work we'll look a year later, which works in some cases, but not in some, those dubious cases where someone has an earache, no discharge, and like it could just be wax, but we've given them antibiotics. Um, so those sort of things have been a bit- adding- adding to the workload. I think other than that, I can't think of anything else that has been an extra, um… for the practice.

Interviewer: OK, thank you that's a great answer. It raises a few new things for me that I haven't really thought of so it's great. Um… so you've spoken a bit about telemedicine, have you had any experience of hot hubs? Or NHS 111? Um…

GPTr3: Um, what did you say? NHS 111 and what, sorry?

Interviewer: NHS 111, um, and hot hubs, have either of these been something you've encountered in your practice?

GPTr3: What's hot hubs, is that the, uh, walk-in centres?

Interviewer: Yeah, those are the places where the Covid-presenting patients have been sent to a few times. Your GP practice might now have had one.

GPTr3: No, we don't have one. We don't- we don't do 111 either. We've got a- we're not a hot hub- we do have portacabins, for seeing suspected Covid, but if you're positive, then forget it you're not coming in! (*laughs*)

Interviewer: Fair. What's the suspected cabin for, is that patients coming in for testing, or?

GPTr3: So they, they- I've never seen a person in there, and it was because, I think it was because her, some relative living in the house had got Covid, or was isolating- no, they were a contact! So they had to isolate, but technically she didn't have to isolate, so we just put her in the cabin anyway, um, just in case, but if you've got any of the four symptoms or a temperature, and it

sounds like you could have Covid, you're not coming. If you've got, maybe a cough, which is like your asthma flaring or something, maybe you'd come in, but generally that could be dealt with on the phone anyway, uh so it's very rare we have anyone going in there.

Interviewer: OK, um, great OK, so on a different note although actually I do feel like you've kind of touched on it, um, what's your opinion of the government response to Covid-19, um, in terms of public health messages, and policies and… pandemic control. I know it can be a bit of a contentious, question, but!

GPTr3: Yeah (*laughs*). I really don't like criticising, because the thing is they've got access all the data that we don't but I feel like they knew what was coming on the horizon in February, if not before that, and then we had the virus was spreading, we just, the borders were open for a very long time, then they said it wasn't imported diseases that was the issue, and that might have been true at the time, but they said it because they've let so many people back home or in, um, but, prior to that, certainly imported disease was how it gets here from somewhere, and then, I felt like, um, in some respects, there was too much lag between them saying something needs to be done, and then doing it, so for example now the, um… quarantine, and the hotels for these people with strains. I think they gave you a good few days headstart, and it's like if it needs- you're going to have all these people rushing back, and importing this new virus strain, you know as far as they can get back

Interviewer: Yeah

GPTr3: And there was a few other things, like, the beginning just felt was very, very slow. I liked New Zealand's response which was just that's that, no one's coming back, and if you are coming, you're going to be in a hotel quarantining, um… I felt the, um, furlough scheme was good, because obviously you know people being financially oppressed in some ways is a determinant of health? So, we've already seen anxiety and depression and things, you know, on the rise, and that's been abated somewhat by people having, you know, some income, so some people have been wealthy- you know, I don't know how wealthy he was, but his income was, I think, halved on the furlough? So for him it was a major stress, because he's still got a big mortgage to pay, his partner's I think a teaching or teaching assistant so she's in contact with kind who, you know, may or may not be wearing a facemask and things like that, um… but having some income I think has abated the absolute devastation of mental illness that I think it would've had on some people, and obviously, it would have, not so much in my area? But you know, keeping children fed and things like that, helped with those things. Um, generally, I just think very slow. We've had another withdrawal of some *unintelligilble* mask I think? And it's just, it's so disheartening, because I don't know if I've ever worn that mask, I don't think I did looking at those pictures, but we did have when I was at, um, the hospital the face… you know the glasses? There was glasses that looked crap from the start, they looked absolutely ridiculous, um, they got withdrawn, because later on they found they

were crap, but it was weeks later, you think, we've al been wearing this, because that's all you gave us, and now you're telling us it's rubbish and doesn't reach the standards, we could've told you that from the start! (*laughs*)

Interviewer: Yeah, it must be so frustrating.

GPTr3: Yeah, it was a little plastic band, that you- it ill-formed around your head, and then a little plastic sheet you fed through and it just covered, it just about touched your nose. and obviously everything was going round the sides, top and bottom, and it was just really frustrating, and it felt- I didn't feel valued as an NHS staff, at all, um, and it's completely affected my career decisions, in terms of I don't want to be employed, I want to be working on my own terms, in some respects, so like as um, a locum, because I just feel that you've got that impendence to make decisions, and decide what's going to work for you. When you're under an employer, you have to do what they're saying, regardless of whether it seems right or wrong, and I- I felt that the government just didn't have our back, because the equipment was shoddy, facemask and things had been pulled because they're not reaching standards, redated masks, it was just, demoralising I felt. And also getting swabs, when I had Covid symptoms, I couldn't get one! I'm trying to think why. It was, first of all, my hospital had a system problem in that- and I've raised it with the **REDACTED PCN**- because they had a problem whereby all GP trainees were not- 'cause our smart card was tied to a different trust, because we're all centrally employed by **REDACTED trust name**, so um, a trust in the North, it's a central contract, because we were all employed by them, our smart card wasn't tied, wasn't technically tied to the hospital, so they technically didn't know we were employees! So when things were going out, they sent a letter to all BAME employees about Vitamin D tablets, to say come and get them because you're at risk or whatever, none of us got them, there was another issue where, um, I was trying to get Fit tested, and it kept saying employee number not recognised, not recognised, so it was like I just dropped off the radar, with all these other GP employees, and it was just do you even know we exist? Like if there's something important we need to know about, and I think it was, um… I think the vaccine came out afterwards, it was the antibody testing and the antigen testing as well, so the swabs, I wasn't getting anything, I had to chase people to find out how to get it, um it was, it was horrible, and yeah, the access to things was just really poor.

Interviewer: Yeah, it sounds like quite a chaotic time. You spoke about raising things with the, was it the **REDACTED name**, was there- was there anyone you could speak to, was there any support with things?

GPTr3: Yeah so I'm on, I'm on the committee anyway for all trainees, so I raised it to say that has been an issue before, because I told the trust, they said they were gonna try and do a computer switch, because they realised… (*laughs*) they emailed me back to say you've managed to uncover a really big computer error, we'll try and sort it, and I, I finished my ST3 year so I, I left, just couldn't care less, there comes a point where you've told enough people and can't keep chasing them, and obviously I don't

work there anymore so, I did kind of try and keep in touch with- because I know the GPs there, to see if there's, um, any changes been had…. but I don't know.

Interviewer: Ok, how what are you doing now, you've finished your ST2, um…

GPTr3: Yeah finished ST2, ST3, working at the GP practice, yeah.

Interviewer: And you said you'd probably want to be locum in future, is that general practice? Has it sort of informed your speciality?

GPTr3: So I'll stay with general practice, but it's also informed how many days a week I can- there's no way… I've always been a five-day-a-week-er, so working 40 hours, or 48 hours, you know, half of the grind I enjoy, whereas now I'm just like no, as far as that's concerned, if I can make my living just two days a week, three days a week, that's all I'm gonna do, because I felt, work-life balance… as you go through the training years, you feel like you've committed a lot of your time to the NHS and the training programme, especially more so than other specialities, so you're working 12-hour, 12 and a half hour shifts, breaks are rubbish and all over the place, so even if you're on a break, nurses have protected breaks, that's just not a thing for doctor – you're bleeped constantly through your break, and I just feel like all the time that I've given back. And then we have this pandemic, and we get clapped, great, whoop-di-doo, we don't even get PPE that's in break, we have our stuff withdrawn, that's all we've had back for what we've done.

Interviewer: That's very disappointing for you.

GPTr3: Yeah very disappointed, and also with this death-in-service benefit, I mean, great that they've introduced it, because originally locums couldn't – well they can't – get death-in-service, if they die of Covid then they can get it, but it's 60 grand which is the same across the NHS, whether you're a cleaner, doctor, or whatever, and how what, you know, how often you've actually worked for them, they give no recognition for the fact that the 60 grand for a consultant is going to be, what 6 months of their salary, for a cleaner is going to be maybe gonna be, hmm, 4 years, there is- there is a difference. I'm not seeing that cleaners do any less a job, because we absolutely need them, but to just say, you know, we'll just give everyone the same amount and that'll fob them all off and it's gonna be great, it's just like, it doesn't working like that, because families can't live if they were on the consultant salary, you know who was the breadwinner, off 60 grand, it just doesn't work like that. If you don't have a life-insurance policy, 'cause the standard death-in-service payment is like, I think it's like 4 times your salary? Which is

proportionate, because I think this is a top-up payment, but it just- it just seems like the government just thought of something nice that they could just hand out to people.

Interviewer: Right I hadn't actually heard about this policy in the NHS, so thank you for raising that. I see what you're saying.

GPTr3: Yeah the other thing as well, was I worked in Burton hospital and, you know the ENT consultant that was-that was the first doctor- I think he was the first doctor- to die in the UK, it was, I think it was our hospital, and it was, I was so devastated. I was on ENT, the rotation before he died, and so I still have like his Whatsapp messages on my phone, because if I was on-call and he was my consultant that night, I'd text him with whatever, and it just bought it so close to home.

Interviewer: Oh of course, and to start like that.

GPTr3: Someone I worked so close to just died, and it was horrendous, it really makes- 'cause he was relatively young as far as I was concerned, sons in school and things like that, so it just made it so much more personal, that Covid can really tear apart, you know, anyone from routine, and it was slightly ? as well, because at that time, when we knew that Covid was hitting us ?.I was doing all sorts of things in case I died! (*laughs*). I was writing my will, I was telling my husband things he needs to do, um…

Interviewer: That must've been such a distressing time.

GPTr3: Yeah it was just all things that you don't want to think about? I mean I don't mind thinking about them, it's being forced to think about them in that context, because you know, that the protection the government was giving you either way… was a bit crap, and something might happen to you!

Interviewer: Yeah, no completely, it's an awful thing to have to think about for being part of the NHS at this time.

GPTr3: Exactly.

Interviewer: Protecting yourself against death, while… well I'm glad that you're ok and that we're on this interview! So, this is more of a sensitive question, but has Covid had any impact for you personally?

GPTr3: Personally… um… I, I don't… other than him dying, uh… I'm ver,y very resilient I think, so when you are in a hospital, you don't see this in a GP thankfully, but when the patients were dying of Covid and you could tell it was Covid, they would be

gasping, they would look like somebody who's dying, but very actively dying in terms of their breathing faster, sat upright, very alert, it was a different way of dying? Usually people fade away, very gradually, and it's very peaceful, but they were fighting for their last breath and then just dropped and that was it? So, for some people, that's had a massive effect. For me, not so much, because I try not to get personally involved. I think I can be quite emotionally detached, I can be attached when I need to be, but I think to protect yourself definitely you have to make a pact to just kind of detach yourself from what's going on around yourself clinically, protect yourself, be nice, and supportive, but that's it, don't get emotionally invested at all? And it's definitely taught me that. You can't rely on employers eventually, you have to rely on yourself, which is an odd lesson, I'd have never thought that I would learn that, I've always been very trusting of the government, they know what they're doing, the NHS, you know… it's all… it's a structured, um, thing, and you know you don't need to worry about it falling apart, but you know, it can fall apart, uh, quite rapidly around you unfortunately. And I think as well, it's made me realise – 'cause I got married, June 2019.

Interviewer: Ah congratulations!

GPTr3: Yeah, and then we had March and it was rubbish!

Interviewer: Yeah, your honeymoon year!

GPTr3: Exactly, so for me, all I wanted to do was just spend time with my husband, instead of constantly, I find in the practice I'm in, you're expected to give above in some ways, and beyond, even it's a kind of unspoken rule, so if the day runs late, you're just expected to stay late? And no one cares, no one says thank you, my practice before that, in ST1, it was different, if you stayed late, you know, they'd be relatively grateful about it, you know, you don't have to – it's not an expectation – and even, like, so we had a meeting yesterday and I was saying – when we go back to face-to-face, or start re-introducing it, um we were discussing if you do have a ?, what is the length of time gonna be? And everyone was like 'ten minutes'. And I was like, how can it be ten minutes!? It was ten minutes before the pandemic, you got two, you've got to go and get your patient, because we're not allowed to just call them in, you've got to go and get them, hand-gel them, before that we've got to PPE and everything, and one of the partners was saying, you know 'we don't need to do much PPE, just a facemask' – well, no, we can't, because at the moment if you're not in a facemask, apron and gloves, for contact-tracing purposes, if you've been in a positive contact, that's it, regardless of ? at work, so you'd have to go home, and that's 10 days, and we'd have to do the whole shebang, so that changes the rules. And everyone was like 'oh, yeah, yeah, that's so true'. So you've got to do that, if you examine them, you've got to clean everything they've touched, the pulse machine, oximeter, thermometer, whatever, chair, door handle, then you've got to see them out, hand-gel, it's like, you expect me to do all of that, plus the normal consultation in the same 10 minutes you were originally allocated? And it's like, what he said, was, and this is part of the reason I've had it with the NHS, is he said, um 'well,

we’re still gonna have to, we’re just gonna have to keep up’, and it’s just like, if I don’t have the time to clean the whole, you know, whatever’s been used, I’m not gonna do it, I’m just gonna move onto the next patient, and hope that I can, just, um… clean later!

Interviewer: I mean, I yeah, I can’t imagine. It just sounds so frustrating knowing these things and not having the option to stay safe.

GPTr3: Exactly, exactly. And the problem is, because- it’s not a huge problem, but because practices are partner-run, and it’s dependent on how the partners perceive things and what decisions they want to make, it’s- it’s not necessarily the best for everyone, it’s just what they- if his- if his priority is making sure everyone’s seen on that day, so it’s however callers have to cut, that’s it, he just assumes we’re all gonna stay late, and do whatever- that’s not sustainable! We’ve been doing this for a year, and it just like, you expect me to do this for another few months until July- or is it June, when they lift everything, it’s just not sustainable, you have to work sustainably. Another thing I don’t think has been kind of championed, you can’t champion it if you’re a GP unfortunately.

Interviewer: Yeah, you need to pace yourself.

GPTr3: Exactly, exactly.

Interviewer: Have you been working from home at all, or has it all been, um…

GPTr3: So we’ve all been in the practice, there’s only been one occasion where I had to work from home, I will have to do that again, it was horrible! (*laughs*)

Both laugh

Interviewer: Oh, really?

GPTr3: So I was a contact for, um, someone who tested positive, pre-operatively, so it wasn’t-they weren’t symptomatic, I found out, I think the next day, so I got a computer and worked from home. It was awful the internet kept cutting out, the uh, the system I was on, so we um, we use EMIS, it would log out, so we would have to reconnect, I was having to use my own mobile phone for it, ‘cause we were having to use- you know you can’t use your own phone, so I had to call everyone on a private

number and things like that. Usually, uh all the consultations are recorded in the practice? So I didn't have that on my phone, so you know when you think, oh medically, legally, what if there's a problem later on? So I was using my mobile phone. I know that not every practice does record anyway, I think that's a *unintelligible* thing, it's just ours do.

Interviewer: Well you were worried about your safegaurding, so of course.

GPTr3: Exactly, exactly.

Interviewer: You were aware of it.

GPTr3: Yeah and then you get your debriefs from home, which isn't too bad, but you couldn't you know… ugh… I was trying to manage my time by like booking an appointment if I knew my computer had gone down and can actually block it out, so I block it, it makes sense, you're running late because your computers gone down, your internet's switched off, and you're messaging the practice manager 'help me', no one's replying, and I got told off for blocking! You shouldn't be blocking without permission! And it's like, you guys aren't responding, I can't wait an hour for you to reply to me, at the end of the day I'm an independent person, I've got, you know, I'm a professional as well, I know the workload I can imagine, with what I'm working with, which is crappy internet and a computer that won't stay logged in! It was horrible.

Interviewer: I can't imagine, but I understand the frustrations of working from home. So you ended up in the practice doing telephone consultations. Did you have any video calls or was it mostly telephone?

GPTr3: In the practice or at home?

Interviewer: In the practice.

GPTr3: In the practice, pretty much everything is telephone. I can do anyone face-to-face if it feels appropriate, originally, I had to ask the partner with each person, um, but now he trusts our judgement. It was just that he was trying to reduce our contact. Because initially, I was shocked about telephone consultations, like how can you possibly manage most of these things on the phone!? But it's actually really easy, um, it does contrast a lot with how, um, we used to do it, so I would say, about, 5 to 10 percent of my consultations end up being face to face?

Interviewer: Is that now.

GPTr3: Yeah, yeah in a week. Although it can go up to, what like, it can be 4, 5 people in a week, it just depends what I have, it can vary.

Interviewer: Yeah, thank you. So I'm aware I've taken an hour of your time so I just have a final question for you. Um, what do you think we can learn from the pandemic, thus far, to inform future GP care? Are there any changes that you think should be carried on into the future, or that you think should be dropped?

GPTr3: OK… I think… triage is a good thing, in general, um it allows you to assess, you know, who is the urgent patients, you know. I think it could be used better, so you know, who is the urgent patients, who is… I think it could be used a bit better so you know, there are people who could wait. I think people need to be able to book ahead. I think that was a big frustration, of not being able to book your routine chronic health management, for three or four weeks the doctors need it, um, a lot of things have to be call on the day, and hope you can get an appointment on that day.

Interviewer: Yeah

GPTr3: Uh… what else, I think, it's so difficult, because- because a lot of the fears are about consulting, as in patients coming in- has come from their perceptions of what's available- um… like I heard on the radio yesterday, an advert saying, if you're worried about your baby, you're pregnant, um, the midwifery team is still here, NHS midwifery team is still here. That was the first time I've heard something from the government saying, um, that-that the NHS is still open essentially. So I think GP groups – I think they probably already have done, but they need to push for more health campaigns to say we are still open, don't worry about us being busy, we're always busy, that's not gonna change, if you need something just call us, don't sit on it? Um, what else.

Interviewer: That's some great advice, yeah.

GPTr3: I can't think of anything else, well… well! OK, I think a lot of locums have been out of work, um, over the pandemic period because there's been kind of less requirement for them, um, I think this responsibility lies less with GPs, but essentially we've bought this pandemic all retired people back, which is all fair and good, but there were a lot of locums who weren't doing very much, who weren't tapped into resources? You know, using locums who have chosen that as their pathway, rather than bringing people back who- I'm a ?? and I've just been sat at home for the last five years and I feel like helping, I think a lot of the time as well, those kind of the people, you can't necessarily shuttle them to the front line, especially if they've got chronic health conditions, um so you're limited in you know, what you can get people doing, whether it's admin, and that's not making use of

their skill set, whatever their skill set was. So I think definitely using locums where it was needed, and I think also… making sure people take their holidays. So we've seen people in this pandemic, all though it would be a couple months and we'll be happy as Larry! It's not, it's been a year, a year and a half now, people are delaying their holidays -uh, I don't mean going away, I mean taking annual leave, um, and also, not being able to take holidays, I don't know- it was certainly like that in the hospitals, our GP was pretty good, they encouraged you to still try and take it, but it depended on your specialty. I don't know about, you know, what it was like in other general practices, but in general we've been able to take- well I've had some annual leave declined, but I think for other reasons other than the pandemic, but yea I just think making sure your staff are looked after, and taking the time that they need to reoperate from things?

Interviewer: Ok, thank you that was a great answer. That was a really good answer, thank you so much! Um, is there anything today that I haven't touched on that you think would be important to talk about, or you know, any experiences you haven't raised that you would like to?

GPTr3: Um……. I can't think of anything. I think I've gotten everything off my chest!

Interviewer: That's good! It's been great for me, thank you so much. You've raised so many new points, and also solidified what I've been hearing so far, in terms of the switch to telemedicine it sounds both beneficial and negative in different ways, especially the risk stratification sounds quite complicated, um, and also, talking about how it's affected your training has been really useful thank you for that. Um-

GPTr3: Oh I'd forgotten about that!

Interviewer: Yeah?

GPTr3: So with my training, um, I was supposed- how it's supposed to be is every year you have four months in GP, I completely lost that four months in ST2, I think I was forced- in fact yeah, we were all forced! We had to stay within the placement we were in, so I ended up having to stay for 8 months in acute medicine, staying on an acute Covid ward, and it was horrible, 'cause I didn't want to be there, for one, you kind of look forward to the end of nights, and all this rubbish you just don't want to deal with anymore, it wasn't your chosen career path, and then I was told about 2 weeks before I was supposed to leave, oh actually, everyone's staying now! Subsequently, um it became optional, only quite recently- oh no, redeployment was optional later on, but that time, the letter just went out and everyone was staying and that's it. Which was very disappointing, very disheartening, you already felt like you weren't looked after, and then you're forced to stay somewhere you don't want to be anymore. And

also, because my exams are this year, so I'm now having to delay til May, because I don't feel like I'm at the standard if I'd have done that four months – my four months would have run into my trial as a GP, so I would've had a lovely 16 month of GP, and you know, it was an apprenticeship essentially, and I was being apprenticed in the complete wrong location, because acute medicine is totally different style of um, consulting, it's very focussed, very symptom-driven, there's no kind of community-orientational care because the nurses will arrange the social care, so it was very limited in terms of that. And then you were seeing the same darn thing all the time, which was just Covid, and so the experience, it just, kind of, nose-dived from that point, which was an unfortunate thing.

Interviewer: Yeah completely, I think I got slightly confused about the structure of ST1 to 3 programme, but, do you choose to specialise in GP and then have to go to acute care despite that, or was that all

GPTr3: So that was, you always have two rotations that aren't GP, this is in the midlands, GP in ST1, there are two rotations in ST3 that aren't GP, and then um, you go to GP. So I got my… ENT I think it was? Then acute medicine, then was supposed to go to GP, then got told you're not – you're now staying in acute medicine here, uh it was horrible because the practise I was supposed to go to is the one I was attending to work at, so I was planning to, um, do, maybe 2 days salaried or two days locum at this practice, and just do… I would've run my own business and my- the rest of the time, or just do some sporadic locums – I'm just gonna make it what I want it to be, as opposed ot being told a certain way. And that's where- you know I was saying about- you know, when you're in hospital you're told, you know, this is your shift, this is what you're doing, and again we got it dictated to us – you were supposed to do this, you're not, we don't know what affect it's going to have on your training, it's tough but this is what you're doing, it was just disheartening, really, it was a lot.

Interviewer: I understand. Thank you so much for talking about all this today.

Recording ends.
