# Supplementary material for: Primary health care during the COVID-19 pandemic: A qualitative exploration of the challenges and changes in practice experienced by GPs and GP trainees
Source: PLoS One. 2023 Feb 9;18(2):e0280733. doi: 10.1371/journal.pone.0280733 (PMC9910752; doi:10.1371/journal.pone.0280733)
Supplement: S1 Data — (ZIP) [file pone.0280733.s005.zip › GPTr4 Transcript.pdf]

**GPTr4 Transcript**

Interviewer: Can you tell me about your experience in general practice, and about your practice itself?

GPTr4: Currently or before?

Interviewer: Uh, before.

GPTr4: Ok, so I am a GP registrar trainee, so, um, when I was in GP for my first placement, that was in my ST1, and that was before coronavirus hit. So I was in a practice in **REDACTED city name** and it was a small practice, um, and I was working as an ST1 there. So, um, my experience was really positive? Um, in the sense that I got exposed to a lot of different kinds of patients, so from different socioeconomic groups and different ethnicities, so I was exposed to a wide range of disease. Um… yeah, I- I did a combination of telephone calls, but not very many, mostly were face-to-face. Um, the telephone calls I did were mostly telephone triage calls, so triaging to see if people needed to come in, if they needed to go to A&E, or if the issue could be resolved over the phone. But the majority of consultations were face-to-face consultations. And at the time, in my ST1 year, I was having about 20 minutes per patient? Which was… a bit stretched back then, because I was um, an ST1, but um, as I got used to it, 20 minutes was actually a really comfortable, nice amount of time to see a patient. Um, yeah, generally a really positive experience. Is there anything specific you would like to know about the experience?

Interviewer: Yeah, well how prepared did you feel for the pandemic, as a GP?

GPTr4: Well… when the pandemic hit, I was actually on hospital placements, so things in GP I guess had, um… been hugely disrupted and I think whilst I was on my hospital placement, the GP that I would end up in now, was probably doing a lot of preparation to handle Covid anxiety, to um… sort of implement protocols about patients coming in with symptoms, um, and obviously ordering and making sure there was enough PPE. So actually, when I joined the current surgery that I'm in now, which was in… uh, December- beginning of December, I have to say they were actually quite prepared. And I was fully briefed on what to expect, what the protocols were, um, what- you know, what I would be telling patients should they call up and say they had symptoms? Also we had moved to telephone-based- purely telephone-based consultations, and only bringing patients face-to-face if we absolutely needed to. So I think they were, by the time I got there, they were quite well-equipped, well-prepared, so then I felt quite prepared? If you would have asked about hospital medicine, completely different story, because when it hit everything was kind of crazy, but um, yeah.

Interviewer: Yeah, how did you find it? So you felt prepared when you got to the GP, how did you find telemedicine, in terms of accessibility for yourself and for your patients?

GPTr4: Did you say accessibility?

Interviewer: Accessibility, yeah.

GPTr4: Um, in what sense?

Interviewer: Did you find that your patients were able to use telemedicine, or did it have any limitations?

GPTr4: Sure. Um for me, um, I think… I think it definitely was a change. I think the benefits were that actually, you could deal with something quite quickly over the phone, which… which meant a patient didn't have to come all the way in. Um, also telephone appointments, they are just a bit quicker, because you're not dealing with, you know, the patient having to come from the waiting room, having to sit down, you know, um, and I think when somebody is face-to-face as well, because you build such a strong rapport, it can be difficult with timing? I feel like face-to-face, people are more inclined to be… you know… Oh! And I've got this problem! Oh, and I've got another problem! Which… is difficult then for timing. So I think actually telephone consultations are a bit more time efficient? However, for me… the limiting factors are, um… the limiting factors were- although we could convert to video consult by the way! There was an app, um which we could use to convert to video call. But even with video calls, it's really difficult to assess somebody that has, um, joint stiffness or joint pain, because you can't do the manipulations yourself, it's really difficult, um, sometimes to see what exactly a skin rash looks like, sort of assessing the dimensions of it, assessing warmth… um, yeah, and I think just generally sometimes, um, older patients obviously, the people who are most at risk of developing coronavirus and having severe, um… morbidity from it, it was really difficult in terms of, you know, using telephone consultation with them, because you can't see them, a lot of them didn't use, um, or didn't have camera phones that could allow video calls! (*Laughs*). So I think for me those were the main limitations, um… for patients, I think actually, there was probably a bit more access? The reason I say that is that my GP practice, we have two systems in which patients can request for a consultation from GPs, and that was only implemented, you know during- after coronavirus had… the pandemic had started. So, one of them is, um… AccuRx which is a software, so they submit a form on that, and then another one, I can't remember what it is, but there's another format, I think it's an online form format, they can submit an e-request for a consultation there. And beforehand, it was mainly telephone calls. Call the doctor, 9am in the morning or… or you call the practice around, between one and two, and you ask for a call back. Now the waiting times for calling into the GP are crazy, so um… so I think actually in terms of getting appointments, possibly a bit more easy for patients? But I guess those patients that

weren't desperately wanting to be seen, um, you know, I think probably, not so much for them, because if we felt they didn't need to be seen, we wouldn't bring them in.

Interviewer: Right, fair enough. You spoke about… sorry there was a few things I wanted to speak about! In terms of risk, uh, sort of like, trying to judge how patients are doing over the phone, do you have guidance for doing that?

GPTr4: There wasn't clinical guidance to be honest, but um, but my supervisor- I work closely with my supervisor, so I go through all my patients with her, but I think the general expectation is, if you're unsure, and if they don't have any symptoms of coronavirus, you can bring them in for a face-to-face and assess the risk face to face? Um… Or, if I was unsure, I'd be able to just talk it over with my supervisor, and she'd be able to direct me, um, as to what to do. But I don't think there was uh… a strict guidance as to risk assessment, in terms of levels of uncertainty, bringing them in, when to… when it's okay to just keep the patient home and do telephone consult?

Interviewer: Was that difficult or manageable…

GPTr4: I think in the beginning quite difficult, but I think as I got sued to it, I think it became a lot easier.

Interviewer: Ok, fair enough. You spoke about getting guidance from your supervisor, has the pandemic changed your relationships with your colleagues?

GPTr4: Yeah… that's a really good question, um, and actually something I spoke about with my supervisor before. So before the pandemic, um, I was definitely the kind of GP trainee or doctor that would be mingling a lot with, you know, the pharmacist, or the receptionist, or practice manager, and um socialising with other GPs as well, so going out with them and having dinners and what-not, so… and um… that actually played a huge role in getting my colleague feedback as a GP? As a GP trainee? And… I think that now, I had to send out my, um, a questionnaire to my colleagues for feedback this week- just this week, and it was really difficult to pick, I think 10 people to give me feedback, because I just hadn't really met people or I- I hadn't been around them, so it's really… um… affected relationships just in terms of not being able to get to know people or form bonds. Um… but again, still- still being expected to submit, you know, questionnaires with colleague feedback. So yeah!

Interviewer: Ok. Um, have you felt that you've had any sort of support emotionally during this time, if, um, you've been a bit separated from your colleagues?

GPTr4: Yeah… um… that's also an interesting question. I think support has been there, definitely, so there has been, um, I think in my- in my GP, um, cohort, there has been, um, a couple of links of information about psychological support put out in our group? But to be honest, I never actually- you know, investigated or went down that route of having, um, a look into it because I- I don't feel like I personally needed it? But I- I know vaguely that something was there, and was in place, but to be honest I never really got full details on it, because I never felt that I needed it, personally.

Interviewer: Ok, well that's good to hear! Um, did you feel that you had protection in terms of PPE, I know we spoke a bit about guidance already.

GPTr4: Yeah, yeah definitely. So our practice manager, um, he's amazing, so he would always make sure- he would come down, well he still comes down, about every two weeks just to check on every GP to make sure they've got a full stock of masks, so he gives us, um, a full stock of masks to each practitioner in the practice, to make sure that, um, you have everything you need. Um… visors also were supplied, but um, but I think that was much later on, so I think masks were the primary, um, and then visors came much later on. But yeah, I think they've been- they've been pretty good in terms of keeping up with PPE requirements.

Interviewer: I'm glad to hear, it that's good news. It's been quite a varied one between practices, it often depends what network you're in, I think.

GPTr4: Oh really?

Interviewer: Yeah, so I'm glad to hear you had access to that. How did you feel making decisions with the guidance you had, sort of making decisions for your patients when you maybe didn't really know the full picture of Covid?

GPTr4: Yeah… I think anxious, nervous, because um… yeah, I think the fear is that you bring somebody in and they have- they have a symptom that perhaps you haven't asked about? So for example, because, um, I guess the…. the official symptoms of corona were- that list was increasing- you know, changing all the time! There was always an anxiety or fear that I hadn't asked about, I don't know, a sore throat! Didn't use to be a symptom of corona, but now it is! So um… I think there was always a bit of anxiety and fear that, yeah, that um, that I'd bring somebody in with corona. Um, yeah.

Interviewer: Did you, um, did you take any of these telephone consultations from home, or were you often in the practice when you took these calls?

GPTr4: I was in the practice, I've only worked from home, like, twice- literally twice.

Interviewer: Ok, so… could you tell me about any changes in your role as a GP, so, um, for example taking on responsibilities from secondary care, or vaccination programmes, etc, anything different really?

GPTr4: Hmm, okay, yeah, so I have been asked to, um… do vaccination training. So that will be a new role I take on, um, in the near-ish future. Um… any other new roles? Hmm, I don't think so.

Interviewer: Ok.

GPTr4: I don't think so, what was your first example?

Interviewer: In secondary care, has it changed your referral system, or…

GPTr4: Oh OK, OK, I think yes, in terms of, um, we've been told that there are quite a few services that will not be taking referrals, um, for a while? So that has definitely changed and caused us to try and mange things in primary practice, um, the best we could? Before being able to refer them, Um… and I think that we now do a lot more Advice and Guidance calls? Because I know that referral waiting times are going to be a lot longer, what I do more frequently now s just call up the respiratory registrar in the hospital to ask for some advice, um, because the patient might not necessarily need to be referred? Or I call, um, I don't know… the paediatrics registrar, instead of sending the child into A&E, for example, just to get some advice. Yeah, so a lot more telephone calls for guidance and advice with secondary care, um, specialists, just so I can prevent, you know, a referral that perhaps doesn't need to be a referral and would be a delayed wait any, or admission to A&E, when there's a lot of Covid anxiety as well.

Interviewer: Sure, and has that been effective?

GPTr4: I think so! I think so, because I've got a lot of- I've had a lot of um… really useful, um, feedback from specialists that… has been really useful and has enabled me to just manage them in general practice?

Interviewer: Great, OK that's good to know. So, quite a broad question, but what is your opinion of the government response to Covid-19, in terms of public health messages and policies? I'm sure as a GP you see the result it has on patients who are trying to find information, so what have your experiences been?

GPTr4: Yeah, I mean I think that, um, as a government, um, I guess there's no perfect, there's no perfect way to handle such an unexpected crisis, um, and I guess in terms of relaying information to the public, I think that that has been… I think it could have been, um… It's really difficult to answer, because things are changing so quickly, that you can't expect- I guess you can't expect even, you know, the people that, you know, are researchers that are studying the virus, and people that are informing the government of, um, you know, boundaries to set, rules to set, um, and new… new guidance- it's really difficult to say. I mean, obviously I think we should have acted sooner, a lot of patients have been really confused about what to do, not what to do, and looking to us GPs for.,. for you know, what they're allowed to do, what they're not allowed to do. Often I've been confused with, you know, specific updates and having to read guidance over and over again, just so I'm giving out the right information, and actually, um, I… I'm on social media and I, um, I do sort of posts about health and wellness, but I actually said to myself I'm not going to post about the coronavirus- guidance on coronavirus, because I'm so afraid of getting the information wrong, or things changing so quickly, and then that being on me…

Interviewer: Yeah it must be so stressful for you to-

GPTr4: Yeah! So honestly, I just completely refrain from mentioning coronavirus guidance, government guidance, on my social media platform. So yeah, I think it's been quite confusing for people, but at the same time, I realise that- it is that, because things have been constantly changing…

Interviewer: Yeah completely, there's no set rules.

GPTr4: Yeah, so in a way like, a part of me does believe that, you know, from a public health perspective we probably did the best that we could? Um… but yeah. I think that, fi this happened again, god forbid, we'd be a lot more prepared and, I think that, um, yeah, lockdowns would probably be better structured for more appropriate durations, and not being, you know, lockdown, not lockdown, lockdown, not lockdown, lockdown, not lockdown. I think, yeah, it\s political as well isn't it, because, trying to save the economy, but trying to save lives, it's…. yeah.

Interviewer: Yeah I mean, I wouldn't know where to start in that position!

GPTr4: Exactly.

*Both laugh.*

Interviewer: I wanted to ask you, do you think that GPs were utilised in the right way in the pandemic?

GPTr4: Hmm. I think probably…. difficult question I guess, because I wasn't there in the height of it?

Interviewer: Sure, yeah.

GPTr4: Um… but Minka do you have a specific sort of question there?

Interviewer: Um… no not really, um, you spoke a bit about different roles. I've had people who appreciated being called in for the vaccination programme because they felt that GPs were the right ones to do it, for example, um, but no I don't really have a specific question so if you don't have an answer that's fine!

GPTr4: Um yeah, I think, um… I think, you brought up the point about vaccinations, I think what could be useful is actually if um the GPs did have mandatory teaching about the vaccines, the pros and cons, um, and also a bit of education about vaccine hesitancy? And how to educate and help people overcome that? So I think actually that could, you know, like in ethnic minorities are less likely to take the vaccine and I think that that, you know there's a lot- there's a lot going around in the media at the moment, just saying, you know, if you have hesitancy, speak to your GP. But I know a lot of GPs that wouldn't know what to say. So I think actually, on that, just more education about the vaccine and vaccine hesitancy could be a way to better-utilise GPs.

Interviewer: That's a great point, thank you, no one's actually raised that yet, but yeah absolutely makes sense, especially when you're the one that has to- that patient's come to you for advice about all these things, completely. Um, this is a more sensitive question, so only answer as much as you wish to, but has Covid had any impact for you personally?

GPTr4: Um, thank god not on a very personal level. But I mean, I think that the biggest kind of effect on me, and that's completely trivial relatively, to other people's situations, was just that my- our wedding got cancelled! Due to corona. And it was supposed to be in South Africa, so that was yeah.

Interviewer: Oh, I'm sorry that's rubbish.

GPTr4: Oh no worries, thank you. So yeah, and I think just not being able to, um, my… um… yeah my sister had some unfortunate news, with losing a baby, so I guess not really being able to be there, present, and support her, and you know just be there physically, I guess, has been a difficulty with um, with having to quarantine as well.

Interviewer: Have you been quarantining?

GPTr4: Oh sorry not quarantining, social distancing.

Interviewer: Yeah completely. Um, how have you managed these things going on in your personal life with the stress of the patients?

GPTr4: Hmm, I think just, hmm… just speaking to friends and family really, just being open with friends and family. And my husband- my husband now- we ended up having a small- small wedding when we had a window of opportunity.

Interviewer: Well congratulations on that.

GPTr4: Yeah! Oh and I will say, um, you know that there's been apps that have been free for NHS, so things like, um, not Calm, what's the other one.

Interviewer: Headspace?

GPTr4: Headspace! I can't remember it but I use it all the time! Headspace has been amazing, because I did have, I think, this past year, more than any time before, I've had, um, quite noticeable anxiety and insomnia? Without really being able to pinpoint all the time what it was, so headspace has been really useful with that. So yeah.

Interviewer: Great, OK. I've used it myself for the same sort of thing. I think it's great

GPTr4: Yeah, it's really, really good.

Interviewer: Thank you for that candid answer, thank you. So I'd ask on a different note, are thee any changes which you think should be carried on into the future, and if so how?

GPTr4: Hmm… Yeah I definitely think um… telephone calls and video consults are really the way forward… and I think definitely- I think it's just really efficient, and again I think that, like I said, I think that there should be implemented guidance into, um, kind of like risk stratification? On sort of- sort of the major areas, so that we know what to bring in, what can be handled over the phone, um, you know, from that perspective. Another thing that I think could be a little bit more accessible is Advice and Guidance pathways as well? So, you know, perhaps having, like, a specialist on-call just for GPs – so we have this, um, specialist that's on-call just for GPs in geriatrics. They just answer GP calls, that is purely their role. So I think more of that in more specialties would prevent a lot of referral and a lot of sending patients to A&E? And I like I said, Advice and Guidance has been probably more used in my practice, and I think that- that is definitely something that should, um, should stay and probably be further developed and rolled out, um, onto other specialties. Um… what else… hmm… I think that's- I think that's it? I think that's all I can think of at the moment.

Interviewer: It's quite hard to think of things like this on the spot.

GPTr4: (*Laughs*) Yeah

Interviewer: So specifically to your, um, experience as a trainee, how do you think the pandemic has influenced your training?

GPTr4: Hmm… First of all, I think that it's been, um, it's really, I used to be very sociable, so we used to have teaching every… every week, um, and see the other GP trainees, which was really nice to actually, um, to see people, to socialise, to interact, um I literally haven't seen- I've seen one person from my- or actually, probably more than one person because I've worked with them, but um it's been really difficult in terms of not being able to socialise with people, and I think that's part of our- that is part of our GP training, just to have a community of support I guess, when you've got questions, or, um, yeah when you just need a bit of support or advice, um, so that has, that has affected training, and I think again, the… has not affected me yet, but um our final exam has been changed to, um, video recordings now, and I know that that's been quite stressful for a lot of GPs, but that hasn't affected me yet, um… I think another way it's changed training I guess, is um… I spoke about teaching, and teaching used to be every week. Um, one day a full day, and one day- sorry one week a full day and the other week a half day? Now because of the pandemic we only now do half days, so it's just changed the amount of teaching, I guess, um, the hours of teaching we get in general? Um… and like I said before, I guess that having to… having to um seek feedback from colleagues has been really difficult because these requirements are still expected on our portfolio, so that has been- has been a bit tricky. And I

think also, you know, asking people to sign you off on this assessment or that assessment has been actually quite difficult, because you know that people- I mean in the height of the pandemic anyway, you know people are not thinking about trying to sign you off on these things! (*Laughs*).

Interviewer: But you still need to get through your training!

GPTr4: Exactly, exactly, and I guess um, finally, a lot of people have been, um… their placement has been prolonged on a specific speciality. So for me it was obstetrics and gynaecology, instead of doing four months I did eight months? For me that wasn't a bad thing because I really enjoyed it, and I, um, I'm really grateful for what I- what I learnt there. But I think- and what I missed out on was elderly care, which I've already done in my foundation years anyway? But I think a lot of other people would've struggled with, you know, having eight months, for example, of elderly care, or eight months of A&E which is a really stressful job, that really just disrupts your sleep cycle, your social life, so yeah.

Interviewer: Ok. Yeah you're right, I've had other trainees who were stuck for eight months on different things and yeah, different experiences depending where you end up.

GPTr4: Exactly.

Interviewer: Sure, yeah. Um… has it informed your specialty choice, are you still interested in being a GP? Is that still what you want to do?

GPTr4: Yeah I think… I think- potentially yes, and in a roundabout way. I think the pandemic and having more time to do- having more time to myself, to do self-reflection, and um put more time into self-development, I think that actually has opened a few more doors for me, in terms of what I want my career to be in the future? I think I do still want to be a GP, but I've been thinking more about portfolio things that I can purse, and I think having this time just to kind of like look inside, and do my own thing without many distractions, has actually enable me to, to actually have a broader, kind of, perspective of what I want my career to look like.

Interviewer: Ok. Out of interest, what are you interested in doing now?

GPTr4: Yeah, so I'm really interested in doing some sort of media, um, media presenting type of thing. I've enrolled in a health coaching course, um, which I probably wouldn't have done before, so I'm really looking to incorporate health coaching into GP further down the line as well, so yeah.

Interviewer: Great, that's exciting! My final question for you is, because I know you need to leave around now-

GPTr4: Thank you.

Interviewer: What can we learn from the pandemic thus far?

GPTr4: Yeah… um… do you mean in a GP-medicine capacity, or in a human capacity? (*Laughs*)

Interviewer: Both really! Yeah, both.

GPTr4: Um, generally speaking, um, human capacity, I think that we can learn that we cannot take life, health, social interaction for granted, because, um, yeah you never know, you just never know. Um, and I think that it has taught us what the important things are in life? Um… yeah, one of those things, just connection with the people that you love, spending time with people, with loved ones, family friends, um… and I guess from… from a health perspective- sorry from a GP perspective, I think that it has taught us, um… I think we can learnt that technology is really, really useful, and I think that we can- there's so much more to do in terms of making our services accessible, efficient and effective, um, that can be aided by technology, and I think that the pandemic has really sped things up in terms of that? So I think that we can just do a lot more development in terms of learning how technology can help us, in terms of our consultations and the way we practice.

Interviewer: Ok, that's such a good answer!

GPTr4: Thank you!

Interviewer: Thank you.

*Recording ends.*
