# Supplementary material for: Primary health care during the COVID-19 pandemic: A qualitative exploration of the challenges and changes in practice experienced by GPs and GP trainees
Source: PLoS One. 2023 Feb 9;18(2):e0280733. doi: 10.1371/journal.pone.0280733 (PMC9910752; doi:10.1371/journal.pone.0280733)
Supplement: S1 Data — (ZIP) [file pone.0280733.s005.zip › GPTr5 Transcript.pdf]

GPTr5 Transcript

Interviewer: So to start I just like to ask, could you tell me a little bit about your experience in GP care in general, before the pandemic?

GPTr5: So before the pandemic, it was mainly face to face with one or two telephone calls a week, um, which... yeah- so yeah almost all face to face.

Interviewer: OK, what's um, what are the practices like that you've been at?

GPTr5: So the first practice that I was in ST- well in F2- was in a mainly white working class, um, mining town, um, quite, deprived, quite a lot of challenges that bring with that and in ST1 I was in a working class, white- predominantly white working class area, but with quite a lot of multicultural pockets in, uh, which led to a lot of interpreter consultations, um... which… probably was useful, the fact that we were doing a lot of face to face then, um, and we had interpreters available at the surgery um... and then ST3 now has all been post-pandemic.

Interviewer: OK, are you in one practice at the moment? Or...

GPTr5: Yeah, just one practice, yeah.

Interviewer: What's your practice demographic like?

GPTr5: So, at the moment again it's white- probably working to middle class slightly more affluent than the places I've worked previously, um, extends out into the peak district so there's some really affluent areas that we cover as well.

Interviewer: How do you think that influences the presentation of your patients?

GPTr5: So, um, it's probably quite a divide and the affluent patients present- present far earlier um... and the less affluent patients, um, probably present- some of them present later and some of them just present much more frequently, um, with- and therefore it sort of becomes a little 'boy who cried wolf' in a way. We've got a good number of patients that we sort of struggle to work out if there's anything new going on, if there's anything serious going on, um, because we speak to them two or three times a week, um...

Interviewer: Okay, um, is it a big practice?

GPTr5: Um, it's about 10,000 patients.

Interviewer: Alright ok, so fairly, fairly big! Alright so, could you tell me a bit about your experience of the pandemic professionally.

GPTr5: So professionally I was- when it first hit I was working at the hospital the **REDACTED hospital name** hospital in **REDACTED city name**, and so we had quite a lot of Covid patients brought over and seen in the infectious diseases unit, um... from the cruise ship, where it all started first in February, so it was fairly intense early on, and then I was working mainly on the stroke wards and we had a couple of early Ward outbreaks, erm, where the majority of our patients got Covid on the Ward, um... Do you need to know about it, mainly from GP, I moved to GP in August, or is that, is this still useful?

Interviewer: Um, I'm interested in GP yeah, but it's good- it's good to know what's you've been doing beforehand, just because it gives a clearer picture-

GPTr5: Yeah so I had quite a lot of experience in managing inpatient Covids during the first wave, Um... and then it probably died down over the summer and I left the hospital, when the hospital was relatively well controlled, over the summer, and moved into GP where again there wasn't an awful lot of community spread of Covid at the time, in August and September, um, so... although the workload was predominantly over the telephone, um... very- we had a very low threshold for bringing people down to be seen face to face, um... because the Community spread was so... or just yeah, there wasn't much in the Community at the time, um... And then that sort of changed gradually throughout the year and we've got slightly stricter about who's coming down face to face, um...

Interviewer: How do you do that, how do you determine who comes in?

GPTr5: So, nobody is supposed to be booked into a face-to-face appointment before prior triage with a doctor. Um, so everyone has a telephone appointment beforehand, um... Where we take the majority of the history, um, and then, if we need to see them, we can arrange to see them after we've asked them- after we've made sure they've not got any Covid-type symptoms, um… but we do the majority of the history still over the telephone, so that the time spent in the surgery is as... well the least amount of time as possible.

Interviewer: Right. How's the process of triaging gone, have you had any guidance on how to do it?

GPTr5: Um, so... it's not- it's difficult to call it a- a triage I guess because we still... it's almost like it's a full consultation over telephone? So it's not just a quick- a quick do we- work out whether we need to see you down at the surgery or not, it's a 'how are you how, can we help today' and take the fall as much as we possibly can over the telephone, um, so we're just sort of treating the telephone as if we had the patient in front of us and we're doing a full a full proper face-to-face history.

Interviewer: How have you found um, how have you found doing that, rather than having in-person consultations?

GPTr5: Um... well it's got pros and cons, I think I quite like the flexibility it brings, um... and I found that as a trainee it's helped me keep to time better, um... and it's for patients that have short yeah you know- things that would previously be really quick face to face appointments can be done just as efficiently over the phone. Um... so I think it's been a really efficient system that my practice have got at the moment I think it's been really good um… and I think, on the whole patients quite like it as well, and...

Interviewer: Yeah.

GPTr5: We've got quite a young demographic, where- where we are, so I think it's much better for them and they don't necessarily have to plan their lives quite as much around a doctor's appointment as perhaps sometimes they had to previously, um, which is good. Um... but... Interestingly, our DNA rates have plummeted, 'cause obviously people just have to answer the phone now, um... Previously people forget and don't come to the face-to-face appointments etc, etc, but now their phone's ringing from an unknown number, so they just answer it, um... and they're like 'oh yeah, I forgot that I called, I forgot about the appointment, but now you're there...' and- and they, so our DNAs plummeted so we're probably actually consulting with more people than we were before, um… from- from speaking to the partners that are there now um... So I think, on the whole, most people like it, we have had a couple of occasions where sort of more elderly people have come down and said that they can't hear anything over the phone, um… that it's not fair on them, you know, can we just book them in to see someone', and so we do try and be flexible, um… And if reception are happy- you know if we ask reception to make sure they're not got any Covid symptoms, which you would hope not, because they're down face to face at the surgery trying to book their appointment, um, a lot of the time, and then we are- we do try and squeeze them in face to face, so I think- I think it's good that a lot of it is done over the telephone, but you need to have a really variable threshold for different people, to allow them to access face to face appointments as well.

Interviewer: That's a great answer, thank you, it- yeah it's a great answer because it sort of answers the question I have about what you would advise, um, for, uh, policy in GP. How- how informed have you felt about the risk of Covid-19 and have you been protected with things like PPE or emotional support during this time?

GPTr5: Yeah so I'm employed by **REDACTED city name** teaching hospitals, and they... we frequently get invites to, um, meetings with the chief executive and the chair of the Junior Doctor committee, and I think they call them **REDACTED name** meetings, um, which I think happens every fortnight, um, where we can raise concerns, ask questions, learn about the like the Cov- the stats in the Community and in the hospitals, at the moment, um...and as part of that they've offered, um, really regular, frequent um... emotional support, um, telephone counselling, stuff like that, um... I haven't felt the need to access any of it, but it's been fairly well signposted so it's there if we need it. And PPE-wise... fine. It is what it is, they- I have accessed everything that I'm officially supposed to have been able to access, you know we've got, um, aprons, surgical masks, um, visors, gloves have never been- never been- had any shortages of anything like that, um, when I was in hospital they felt a bit frustrating and confusing that we were starting wearing the gowns and then the gowns got taken away from us, um… and we're getting...

Interviewer: It must be very distressing, for people to be able to say you need this and then run out and say actually you don't.

GPTr5: Yeah exactly, I think it was it was just the fact that we were told it at one point that was taken away from us when, really, the guidance changed just because they couldn't get hold of it, um, and, and that felt a bit disconcerting. But... yeah and then there was also the weird guidance at the time when I was in hospital, when I was carrying the crash bleep, about the anaesthetist had to wear an FFP3, because they were near the airway, but everyone else was OK in a surgical mask, um… which just felt very, you know- if someone's doing compressions and the same distance away from the airway as everyone else! And so that felt very unsatisfactory, um...

Interviewer: Yeah, I've heard similar things from other trainees I've spoken to, that yeah, the allocation is a bit flawed sometimes.

GPTr5: Yeah, and you'd be there sometimes, you've been to a couple of arrests, where we'd be there in surgical masks and then aesthetics would come down in the full hoods with the full space suits… it was just such a disparity- stood in the same place, a meter apart and it just felt like, why are some people getting all that, and yeah.

Interviewer: There must be frustrating, did you feel what you had in the practice was adequate for the exposure or did you have, um, some people spoke spoken about like red zones or hot hubs? Did you have anywhere where Covid-likely patients were seen?

GPTr5: Yeah, so it was, um, all done in the primary care networks, so we had a couple of GP surgeries across the city that if we were speaking to someone on the phone and we felt like they needed to be, um, assessed face to face, and they had Covid-type symptoms, we could book them into there, um, or if they were isolating for exposure reasons or family reasons, um, we could get them booked into there.

Interviewer: Okay, great. Um, how did you feel making decisions with the guidance you had for the patients. Um, I can imagine you were being asked for- being asked about things that maybe you didn't even necessarily know about depending on where we are at in the Covid timeline. How did you handle that?

GPTr5: Yeah so we were asked about even- even now, this week, you know when the new shielding letters have gone out, we're getting a lot of queries about people that have had previous gestational diabetes that have received, um, shielding letters which we don't have any control over, um, and we don't necessarily know exactly why they've been sent it, we can make best guesses looking at their notes, but we don't know for certain that that's what the risk algorithm has picked up on, um, so that's quite frustrating, um-

Interviewer: Sorry.

GPTr5: No, so we're sort of saying at the moment we don't know- if you've been told to shield by the algorithm then assume that that's correct, um, but we've had... Because often it's- it's grey areas, it's like people that have had previous gestational diabetes, but then also they also smoke and they've got a really high BMI so we don't know if it's like a- a holistic thing, where it's pulled out all those three separate codes and churned those out to say yes, they are high risk, or whether it's just the past- past- past gestational diabetes, which has been coded and then that's what's causing the problem, so we're just saying we don't really know, if you feel like you really don't want to shield, then we can't force you to, um, it's for your protection, not for anyone else's, so if you want to go against that then that's up to you, but we can't advise that, um... and we've had that quite a lot throughout, especially when- it's not even shielding, is it, it's just clinically extremely vulnerable, sort of way it changed from 'shielding' in the spring to 'clinically extremely vulnerable' later in the year, and people asking us whether that- what that meant to them, and we a lot of the time, we had as little knowledge of that as they did.

Interviewer: That must be quite frustrating for you, having to explain- yeah, explain things that you don't really understand yourself. Did you feel that you were being given any information about- or do you think, do you think it was considered that you were going to be the one having to explain these things to patients?

GPTr5: No, I suspect, it wasn't, and often what happens is that we have the problem for two or three weeks and then it's clarified, so, and that's- that's what's happening at the moment with the gestational diabetes, I think it's dawned on people that we're now being asked those questions, um, trying to work out why and we'll get the answers in a couple of weeks, so- and that was the same with a couple of other incidences yeah, um... where we weren't really sure, weren't really knowing what to do, um, then eventually the information filtered down to us, and we were able to get it out a bit more comfortably.

Interviewer: Okay, thank you, that was a great answer. Um, and it's something that seems to keep coming up, I've had so many so many people say the same thing that is- yeah nobody really considers the fact that GPs are the ones that know patients and have to deliver these things, like the information. Um... in what ways is common practice changed for you as a GP? Obviously you've spoken a bit about telemedicine, I assume that's through... have you been using AccuRx, or?

GPTr5: Yeah, yeah, So I think AccuRx is brilliant, um, I use it in most telephone consultations, um I just send some of those after, I send them a quick text with a couple of websites, perhaps, or just a quick, um, 'nice chatting to you, this is what we'll do, I'll speak to you... whenever', um, just to sort of close the loop, make sure that we're all on the same page. So I use AccuRx all the time, um... as you say, I use telephone a lot. Um, other than what I've already said I don't- I can't really think it's changed an awful lot of what- what I do.

Interviewer: Has it changed the roles that you have- Have you had to do anything that you're not- that secondary care would normally be doing or, uh, vaccinations anything sort of different to standard GP care?

GPTr5: Yeah I've been doing some of the vaccinations and the home visit vaccinations for Covid, and the flu vaccines.

Interviewer: How have you found that?

GPTr5: Quite interesting, something different. Um... It was- it was made very clear to me that it was optional and I didn't have to do it if- as a trainee if I- if I wanted to concentrate on seeing my own clinical patients, then I could do that but if I wanted to help out in the vaccines, then that would be- that would be helpful, so I chose to help out. Which is nice that they gave me that

option, um... I don't- I can't think of an awful lot of occasions where, um, I've been asked to do stuff that felt like it should be done by secondary care and that I couldn't access secondary care.

Interviewer: I've had, um, I asked because I've had a few examples of people saying they've spoken to... they've been trying to- because obviously referral times are so long, um, they've had to change how they do referrals or what they do for patients, but if you haven't that's fine, it's just why I asked. Have you- have you experienced delays in... patient referrals or patient-

GPTr5: -yeah a couple of times, there's been- you know, um, I guess now you say that I hadn't really thought about it but there's a couple of people whose knees and joints I've injected where previously- whilst they're waiting for an orthopaedic opinion, because they've been told that orthopaedic opinion is going to be ages, um… and then there was an ENT patient with hearing loss who normally we would refer to ENT and ENT would organize the MRI scan, um, but they've been told their ENT appointment wasn't going to be for five, six months, and so I organized the MRI scan in the community, um… so that we could get a bit more information in the meantime, um, which, yeah, I guess I wouldn't have done, had that appointment been sooner, I would've left it up to ENT.

Interviewer: That yeah- that's a great example.

GPTr5: I hadn't really thought of that yeah.

Interviewer: No that's fine, 'cause it yeah, it's not exactly- when I say, well, role it obviously sounds like a whole job, it's just like a slight shift I guess in, what standard procedures are. Thank you that's interesting. Um, has it changed the relationship between yourself and other colleagues in the practice? Are you still going into the practice or working from home office also, sorry I didn't ask!

GPTr5: Yeah now so we're still getting into practice, my practice has... um, yeah it's- we probably don't social distance, as well as we perhaps could or should, um, the... everyone used to have lunch together, um, all the admin staff, the nurses and the doctors used to go out for lunch at the same time. That's now been split into three separate times to limit the amount of people up at the same time, um, but people are still able to sort of socialise with colleagues at lunchtime, just- just fewer of them. But yeah, I know colleagues who work in the same city, who were… speaking to their GP colleagues in the room next door, via Zoom, rather than popping in and saying hello and asking them a question which I think is insane, but I can understand why you'd want to do that.

Interviewer: Yeah, it's a very- it's a very different time but yeah I've heard some GPS who have massive meetings together, some who speak by video call it is interesting. Do you still feel like you have enough contact or, I guess, sort of like um social support, from your colleagues?

GPTr5: Yeah, yeah they- at my surgery yeah definitely, they've been great, um, it's been fine yeah.

Interviewer: Ok. Um, how has it changed your relationship with your patients, if at all?

GPTr5: Um... I think, there's a... some patients are extremely, they- they talk a lot about 'I really, really didn't want to call about this it's been bothering me for a while, but I know how busy you are', and are really sympathetic, and then there's the flip side, where people are angry about the delays in secondary care, um… and then we seem to be the point of contact for them venting saying why is it taking so long, why is it taking so long, why is it taking so long, um… so I think it's a bit of a split really, some people seem really frustrated about how things are at the moment, and some people are really overly understanding, I would say.

Interviewer: How do you, um, how do you handle the patients who are getting frustrated?

GPTr5: It's- it's just about trying to like, like in the situation I described about the ENT appointment and the orthopaedic appointment, we're doing what we can in the Community, um... to keep them ticking over in the meantime, and trying to speed things up for them when they get to the next step. Um… and there have been a couple of occasions, I think not myself, but my colleagues have had to re-refer with more information if you see what I mean, um, so, in the interim period whilst they've been waiting for the appointment, they've had to send another letter saying 'they're still waiting for an appointment, and this has not happened, I would appreciate if you could see them sooner' and- and that’s happened, they have been seen sooner, um... so I think it's- and then part of it is just trying to manage expectation and I think everyone that kicks up a first doesn't necessarily mean that we can open the doors for them and get them seen quicker, um, but I think it's just having in the back of the mind that sometimes things do change, and they might need to be seen quicker.

Interviewer: Great, thank you. Another great answer yeah, it just- it just covers a lot yeah. It must be difficult, being the person that these- person that patient sees as their first point of contact, in these situations, but I'm glad to hear that also some patients are patient with the process.

GPTr5: Yeah, really patient, and I- I would say, perhaps sometimes too patient, um...

Interviewer: Yeah, do they ever present late from...

GPTr5: Potentially and it's not necessarily stuff that's- that's, you know, serious that gets put- that's presented late, and that we miss, or you know, that could have been dealt with much sooner, but it's just stuff that, like, there's really been bothering them, um… and it's not life changing but, you know, we've been open all of the time, um… and could have dealt with that at any point during the pandemic and I think it's hard for patients because they really do want to do the right thing and stay away as much as they possibly can. But I do sort of feel sorry for some people that have put up with symptoms, for months, that really could- could have been dealt with at the time.

Interviewer: Perhaps not realizing that it is still accessible.

GPTr5: Yeah and I think they probably could have been, I think the message to sort of 'stay away' and not bother your doctor unless it was an emergency, was perhaps over-the-top in a way.

Interviewer: Yeah well on- on that note, I wanted to ask if you have any opinions on, um, like, the government public health policies and control of the pandemic during the last year.

GPTr5: Yeah um I think it's difficult to know because I only have my experience in my practice but we had availability- we've had availability throughout, um, and so, when patients have run up and said 'Oh, I Really don't know whether to bother you with this', we've always been saying we're open if you need anything, call us like you normally would. Um.. and- but I think a lot of people have been put off by some of the public health messages, um, saying, 'if you can manage things yourself then do, and- and stay away, etc, etc', um, but I know practices that have been much harder hit than we have, and they've had staff off, and they've had to close the practice, and things have been much trickier, um… so, from my point of view it felt like it was a bit over the top, but I can understand for other practices, if they were in a different position to us it was necessary.

Interviewer: Ok great. Well I'm glad to have your point of view, because yeah, I have, yeah, a mixed- mixture of different demographics, but to get the overall picture, yeah definitely yeah. Thank you. Um, so, a more sensitive question, you don't need to answer it, but has the pandemic impacted you personally?

GPTr5: Um, in the same way that it's affected everyone, personally, I guess, um, I've been fortunate in that I don't know anyone close to me that was passed away, um... or you know, has been affected, in that way, um... it's been- it's been crap in the sense

of that we haven't been able to do anything that we want to do or enjoy, but I'm conscious that I've been- both me and my partner are very fortunate, we've been able to work... throughout, and haven't had any sort of money issues or significant boredom issues, because we've been working five day weeks, etc.

Interviewer: Yeah, yeah I'm glad to hear. Obviously as a trainee, I know, it could be a stressful time to be- well, stressful in any situation, but that's why I ask. Ok, have you taken any measures to- any protective measures or, like, sort of, physical or mental health sort of, you know management strategies.

GPTr5: No not really, other than just trying to, you keep busy exercise and keep active.

Interviewer: We've all started running.

Both laugh.

Interviewer: And in terms of future general practice, are there any changes that you think should be carried on into the future, um any changes that have been positive and, if so, how would that happen?

GPTr5: I can't imagine that- a world of ever going back to 15 face to face patients in a session, um... I think that would- I think that would be ridiculous, as long as you've got that flexibility to do stuff in other ways, like video calls or face or face to face if you need to, um, I will push if I'm ever in a practice that wants to revert to face-to-face, to say no, um, I would always want to have that split that we've got now I think.

Interviewer: OK great, um, are there any changes that you think should not be carried forward, can you think of anything that's been- any negatives from this time?

GPTr5: Not- no not- not- specifically and, no, not from- not from a GP point of view, no.

Interviewer: Great. Have you had um, the same amount of, uh, I don't know what they're called but like, commissioning groups…

GPTr5: Like CCGs?

Interviewer: Yeah or CCGs, and so forth during their um, like, annual checks, have you had any difference in that or?

GPTr5: So there's been changes to the- when we go into lockdown, like, official lockdowns, um, we get stopped from doing minor surgery in the- in the GP surgery, um, other than that, we don't change anything, I know that some of the QOF criteria has been changed throughout the pandemic, to say that you don't necessarily need to this, this will be marked as null and void, etc, etc during QOF- uh, during the pandemic, but there are things that will presumably revert to normal yeah.

Interviewer: Okay, great thank you, um, so as a trainee, how do you think the pandemic has influenced your training?

GPTr5: Um, I missed out on a rotation in OBS & GYNAE?

Interviewer: Did you do the eight months instead of four months?

GPTr5: Yeah, so I did I did eight months on stroke, rather than four months on stroke and four months on OBS & GYNAE,

GPTr5: which is obviously... when you've only got four hospital jobs in your training, that's a relatively big chunk, um... my exam changed as a trainee, um, instead of doing the CSA OSCE-style exam face to face with actors, it changed to the RCA recorded online consultations, um... which initially I thought would be great, because you don't have to revise or worry about an actual exam, you can just record your normal consultations, but I think there was, or, there are problems with the exam. Kind of trying to shoe-horn real life, patients into what you would expect an actor to do or say, um, doesn't quite work, the patients don't quite get it and, but- so as a trainee it changed my exams and it changed my jobs um... but I think I've been fairly fortunate that is not changed an awful lot else, I noticed some- some trainees have got- have just- just worked on Covid for the last 12 months, uh, some colleagues I know, and that would be awful yeah.

Interviewer: Yes, very intense uh... has it influenced your view on general practice, are you still happy to be going into this field?

GPTr5: Yeah, definitely.

Interviewer: Okay that's good to hear. Um, a vague one, but what- what have you learned from the pandemic?

GPTr5: Um... I think it's just- it's just shown that change can happen quicker than it necessarily was happening before, um, there's no, I think a lot of people, especially my generation, wanted things to be like how they are now, and but there's a lot of

pushback from more, what older- older GPs I think, um... who wanted things to be how they had always had them, um, but the pandemic forced the change, um, which now, the vast majority of people seem to think it's beneficial and- and it's just shown how quickly we can do stuff if we- if it's forced upon us rather than, we just have to let rumble on yeah.

Interviewer: Thank you you've answered all the questions I had and more! Do you have any questions to ask me or is there anything that I haven't raised that you think is key to your experience?

GPTr5: That’s okay.

Interviewer: All right, well, thank you very much your time.

Recording ends.
