# Supplementary material for: Primary health care during the COVID-19 pandemic: A qualitative exploration of the challenges and changes in practice experienced by GPs and GP trainees
Source: PLoS One. 2023 Feb 9;18(2):e0280733. doi: 10.1371/journal.pone.0280733 (PMC9910752; doi:10.1371/journal.pone.0280733)
Supplement: S1 Data — (ZIP) [file pone.0280733.s005.zip › GPTr6 Transcript.pdf]

GPTr6 Transcript

M: So to start, could you tell me about your… general experience in general practice pre-pandemic? So about the practices you work for?

GPTr6: Yeah, so um, the majority of my GP, um, experience before that was really based in an F2 GP placement that I did? So that was when I was training up in **REDACTED city name**, um, so that was kind of the- kind of standard face-to-face consultations plus home visits, and things like that. I joined my GP practice for GP training about three… ish weeks before, um, kind of all of the stipulations came in and we had to quickly transition from standard face-to-face and home visits to an incrementally guarded approach to seeing patients, so it was quite an interesting time actually, to- to go in.

M: Have you been based in one practice, or rotated through?

GPTr6: Yeah so I've just been based in the single practice during the pandemic yeah.

M: Could you tell me about your practice demographic?

GPTr6: It's quite interesting really because, um it's in an area, I suppose one of those areas you see quite a lot nowadays where there's a fairly well-off pocket and there's an area, where um… patients are just about doing OK, and then there's another, kind of, district where there's a lot of social deprivation, a lot of kind of people who are unemployed, a lot of domestic violence, and that kind of thing. Um- and my practice initially served a population which was more the middle ground? Um, but then a really large, kind of, multi-practice conglomeration closed last summer, so we took on 1.5 thousand extra patients from that area.

M: Right, that must've shifted the presentations you were seeing, I can imagine?

GPTr6: Yeah, quite a lot, yeah.

M: Um, what sort of- what are you seeing now?

GPTr6: So, um, it's a real mix now, it's been really interesting in terms of the kind of things we're seeing from the kind-of patients who were with the practice beforehand, were kind of- quite standard between all the age groups, but then the patients from socially deprived areas, we're seeing a lot of people, kind of, coming with mental health issues due to their housing, um, some people being around, kind of, areas with high drug use, also people suffering as a result of going through domestic abuse,

or actually going through court proceedings at the moment. Unfortunately seeing some children being affected by that as well? Um, and then seeing a lot of patients who I suppose… You would hope their health would be better if their conditions in life were better, kind of coming to us with dental abscesses, um, due to poor dental health. Also an increase in the amount of, um, people asking for controlled drugs, and things like that. But I think that's probably been like that for patients across the entire demographics, there's been a lot of people kind of, saying- I would normally try and cope with this but it's just one thing too much with the pandemic, I need zopiclone- or something like that. Also seeing a lot, from the, kind of, patients- not from the deprived areas but from particularly from the parents coming with an awful lot of stress having to work from home and having their children at home, um… and kind of that…

M: Yeah that must be tough

GPTr6: Yeah, and also noticing a lot more with their children, so coming to us and saying- our children are around us much more than usually, we're noticing this about them, we're not quite sure if their gait is normal…

M: That's interesting – yeah, the consequence of more observation of your children. Yeah that's really interesting actually, nobody's mentioned that yet. Could you tell me about, uh, the changes that you've experienced in general practice?

GPTr6: Well I supposed, kind of- it was quite a swift change, so if it helps to put it in a little bit of context, my uh, patient was really unusual in terms of most of the practices around my area in **REDACTED area name** when kind of the pandemic really started to hit in the UK, closed for a few weeks? Almost completely. My practice didn't, kept seeing patients face-to-face, and tried to gradually transition bringing, um, public health measures and things like that. So it was really interesting, because day-to-day, everything was changing so you'd come into work and there'd be another kind of change to the environment over the way that we were seeing patients, um… so in terms of, kind of, what happened it was- it was, kind of, keeping appraised of what the practice needed us to do, but also sometimes actually the patient distressed in the way that they're – the way they could access us was different. And I think it's a really tight-knit community, where actually some of the GPs were born and raised in that community, which is really, really nice, but I think a lot of the patients really, really struggled with that kind of disconnect, so I think what we did was gradually over a few weeks, the face-to-face appointments were cut down, and what started to happen was we bought in the telephone triaging and then bought patients in. I think there was one or two weeks which were really, really tense, where we hadn't actually cut down on the face to face appointments, but the patients were getting really, really anxious in the waiting rooms, um, obviously the kind of- the standard is if you have a patient who takes a long time, then someone else's appointment- they're waiting for 30 minutes. Usually- well I'll- I was- I was anxious too, but they were also anxious staying in the waiting room, and then we gradually moved- I say gradually, but maybe over a couple of weeks (*laughs*)

moved to telephone triage, um, and what my practice has done is they… haven't used the e-consult software or anything like that? So in terms of, quite a lot of practices around this area, I don't know about **REDACTED country name** and other areas, but um, a lot of the patients have to fill in, kind of, this quite long-winded e-consult questionnaire before they get- that gets then sent to the GP, who reads through it and decides do they need a telephone, or invite face to face to do blood tests or something like that. So what's actually happened in my practice is that patients can still phone up and book in an appointment, and they actually get a time for their appointment? So we've had quite a lot of really good feedback from our patients, saying it's really good you're offering discrete telephone times for the appointments, because our lives are still going on- you know, with long business meetings or things like that. And then, we talk to them, and then it's a triage in terms of face to face… or recommended blood tests, and then coming back for review. Um, there was a fair amount of challenges, particularly when we took on the extra 1.5K in the summer, initially, um, people not being able to get access to appointments, um, quickly, and that's something the surgery's always prided themselves on? So I think that was an issue for a few weeks, but, kind of, bottomed out once, I think- I think a lot of people had had to store up a lot of medical issues when the practice they were in before had, kind of, um, failed, for want of a better word. Um, so in terms of the way that we- we worked, and how it changed. So um… we stopped having the weekly formal practice meetings, kind of, in the- in the room together as a multi-disciplinary team, and then, kind of, lunch times used to be used for a, kind of, informal, um, experiential learning really? And also to discuss cases, support teaching, things like that. And that really went, um, obviously because we had to, kind of, social distance. So a lot went to, kind of, email. My practice didn't really use Teams or anything like that, which I know a lot of practices did to kind of start practice meetings.

M: How did that effect your relationship with your colleagues, having less physical interaction?

GPTr6: Yeah so I think it was difficult, and possibly more difficult for me and for them, with respect to me, because they didn't know me in the way they knew the partners who had been working there for 20, 30 years.

M: That sounds tricky.

GPTr6: Yeah… And I was very conscious also, for quite a lot of the pandemic, that obviously without knowing their personal details, risk stratifying them- they were in much higher risk for, kind of, serious complications of Covid-19. So I was, kind of, very conscious in my responsibility to not have too much close-contact with them, and things like that? So I think it cut down on the informal kind of support and kind of networking and talking about cases, and that kind of, you cultivate almost a community of learning don't you, um, kind of, really lost that, and I think that's one of the core things about GP that's great? That you have that, kind of, community feel. Um, it did- it's got better, and certainly it's got better I would say, probably since the Autumn.

Because I think people kind of just- it's kind of the new normal now, and everyone's… to some extent learnt to live with the risk. Possibly I think people have started the feeling of just- their anxiety levels have had an edge taken off with their first vaccinations, and things like that, and the fact that our practice hasn't- well we did have one trainee with Covid-19, but it didn't spread through the practice, so I think that that's been good, because we know that we're doing all the right things. Um, but it has been, I think it has been really difficult, because you've kind of lot that sense of teamworking to a certain extent. I think a lot of our communication again has become kind of remote, so on E-mis we'll use the messaging function quite a lot to each other, where we would usually kind of pop into each other's rooms. Um… and again, email and things like that.

M: On a similar note, how has it affected your relationship with your patients? Obviously I know you're still getting to know them as you're new to the practice, but um, having to manage all these anxieties, how have you personally managed that?

GPTr6: Yeah… I think the pandemic has been really- I find it really, really interesting in terms of the sheer amount of responsibility I feel for my patients? In terms of- it's really affected my behaviour in my personal life, because I think I try to- you know, for obvious reasons I try not to expose myself to risk, I try really hard not to do that, because I know I am seeing, you know, really unwell patients on chemotherapy, or in their 90s, or things like that. I think the patients have found it really difficult. They found it especially for the first few months, probably into the Autumn, in terms of missing that human connection, and, um, the kind of- I think because it's a very much- a practice embedded in a community, it's almost part of their social contact, so kind of, a lot of the patients we usually do routine home visits on, we haven't done, um, probably- we started doing those a couple of months ago when the levels were low. And that's partly because- that's one of the main ways that they socialise sometimes.

M: I'm sure it's the main part of their calendar!

GPTr6: Yeah, there's quite a lot of anxiety, um, and I think people find it more difficult to be open on the telephone, particularly when they're at home? I think sometimes it helps being completely detached from their home life, when talking about some things, and I think we've been aware because there's been a case- at least one case in our region of where, um, someone only disclosed domestic violence when they were brought into the surgery after some time- the implications for that. But- and it's- it has just been really lovely when patients have come in for their to face appointments, and sometimes I'll see someone after speaking to them three, four times on the phone, and they'll say that it's really nice seeing- kind of, putting a face to the name. And you just miss so much. I think it's quite interesting coming from anaesthetics, where you're quite clinically-minded, you're working with numbers and protocols, and kind-of scientific theory, but GP- so much of it is about kind of the nuances that individualise perception of how someone's kind of moving their head, or responding to something, and it's all about

communication. And you miss so much of that when you can't see someone. And the trust- you can't build up the trust, especially as a new doctor in that surgery, you can't build it up as much?

M: Yeah that must be difficult. In terms of guidance for the switch to telemedicine, how supported did you feel, did you, um, because I can imagine it's difficult to do things like risk stratification whilst you're learning how to use telemedicine. So how's that been?

GPTr6: Yeah, so, in terms of the guidance I think, particularly the risk stratification, there was a really, really, uh… a real stress point I think? Um, and I think for quite a lot of- particularly GP trainees, especially because we're also at the same time learning to deal with more uncertainty than we have in hospital-based medicine, um, so it was quite difficult in terms of trying to learn about general practice, but at the same time trying to keep abreast about the current guidance on, you know, dealing with risk via remote consultations, especially with the Covid-19 patients we were supposed to assess initially over the telephone. And then sometimes they were video. And I think that the trouble was- I think that we tried to trust the guidance, so for example there was a particular way that we tried to risk-stratify whether we need to send someone into hospital with Covid-related difficulties, and we kind of trusted that and used it for a few months, and then an alert came out saying- please don't use this, there have been multiple incidents where people have died because they've been risk-stratified by this, and it was- it's been, um, really unsettling.

M: Thank you, that was a very candid answer. That must- I can't imagine, that's very frustrating, I can assume. Um, how did you feel- and you've sort of answered this, but how did you feel making decisions with the guidance you had, um, sort of advising patients on things that you were still learning about?

GPTr6: Um, I think that for kind of a few months I learnt to live in my practice with a degree of um… a question mark, all the time (*laughing*), and I did quite a lot of checking up on patients to see how they were- how they were doing. Um, I think I did a lot of kind of, I spent a lot of time phoning people back when I wanted to check how they were doing, or booking them in for follow-up appointments quite- quite soon after, and things like that. And I was certainly more risk-aware, or, my practice changed a lot more I think, than my GP partners, 'cause they did remark on it, and they tried to talk to me about that. Um, so I did find that really difficult because I think I felt a lot of responsibility and I'd gone from kind of, settings where you'd- you know you'd at least be able to take someone's blood pressure, or put a SATs probe on someone's finger, or even just looking at someone, so that's something that I really learnt from doing the telephone consultations and bringing patients in. The things that people would say on the telephone- I've got quite a vivid imagination and sometime I'd be thinking- Oh my goodness! This sounds horrendous! And I'd book them in for a really early emergency appointment that morning, and they'd come in and

there'd be nothing remarkable at all. And then someone would just say something very benign, and I remember one particular person came in, just because she came in with like a shopping list with six different things, and I thought- she's elderly, so I'll bring her in. I put a hand on her abdomen and it was rock hard, and they subsequently found a really advanced malignancy, and I think, possibly… I found it harder as I went on after a while, because I learnt that the information I was getting on the phone didn't necessarily match the reality.

M: That must be so… yeah. Yeah, I really sympathise, um, having to stratify going by what people say, completely what you've said has been reflected by over people, patients will under or over compensate on the phone. Um, how prepared did you feel, in terms of physical, um… protection- things like PPE, or I don't know if your practice had things like red zones? Did you gave anything like this to look after yourself?

GPTr6: Yeah so, um, so the practices in the area, the health board asked them all to come together and to make, kind of, a Covid centre but they actually- a lot of the GP practices in my area rebelled because they wanted to look after their own patients, so we created a red zone, which was in a separate part of the GP practice. That was- that was good. In terms of PPE, it was quite challenging to start off with, and it was quite uncertain, and I think we all had big question marks. I think what really helped was the communication from the partners, and that they were trying to do their best, and also saying to us that they were uncertain about the guidance too, but I don't think that slight lack of confidence ever went away, or didn't go away for multiple months in terms of, there was just so much out in the media about whether this PPE was appropriate, and the infection levels and things. And I think my personal anxiety levels only started to dissipate when I started seeing Covid patients, or patients that I didn't think had Covid at the time as they were asymptomatic, but then got tested afterwards during- and I saw them within the infective period, and I didn't contract Covid with kind of measures that I was taking. Um, because I think- I think I would, have been- if I was just responsible for my own health, I would've felt less concerned, but I felt like I was a vector for all of these…

M: Yeah well I understand that! You've got the elderly patients coming in and you've got the young ones you are asymptomatic, and yeah. Did you take any- you said the measures you took for yourself- what were the measures you took for yourself?

GPTr6: So it's quite interesting actually, in my practice um people kind of almost jested about how I because I followed everything to the letter. So for quite a long time people would pop into each other's rooms to talk to each other, or wouldn't necessarily always wear their mask around because- you know **REDACTED country name** was a little bit later than **REDACTED country name**? The mask, um, eejit? So I would wear my mask all the time and I'd make sure I kind of cleaned my hands, um… and I'd try and distance form them and kind of step back, so that was the kind of thing I did when I was at work. And for quite a long time I remember people thought that was funny. I remember one time I had to do a tutorial, they wanted me to do it in

person, not on Teams, and I was a little bit uncertain about that. When I got up at the end of it, I offered to, um, clean down the chair I'd been sitting on, and he just burst out laughing! (*Laughs*). Which was quite funny. But I'm glad I did it, and I think yeah, it was a time to follow the rules wasn't it.

M: Yeah, you haven't lost anything by taking these extra precautions. And I remember, probably a lot earlier than **REDACTED country name** but people were wearing masks on the bus and it looked funny because it looked so over the top, being in a different country, but obviously it wasn't it just takes a while to become a cultural norm. Um, but yeah that's interesting. This is a slightly contentious question, so answer how you wish, but what is your opinion of the government response to Covid-19, in terms of public health messages and policies? I'm sure that you're mitigating for the guidance your patients receive, I understand that GPs tend to be the people that patients go to when they don't know what's going on. That's a convoluted question!

GPTr6: No, no, it makes a lot of sense. So I think, I mean gauging from what patients have said, I think the messages haven't been clear and they haven't been consistent, so I think the government could've done much better in terms of giving a clear vision for what they were trying to do, and even if they felt they could- they wanted to experiment by pushing to improve the economy, you know, for a certain amount of time, and then changing things again, I think it would've been better to just be consistent in terms of their approach. I think so many changes, in terms of local, um, kind of restrictions coming in and out, and so much about- people saying with great certainty- we're never going to go into a lockdown again, and then things switching back. I think that's really, really damaged, um, how the public can trust in the government advice? And I think also the fact that the government position has often been very different to the position of the doctor-leader messages? So I think that's really created quite a visible gap between us, and I think at some points it's been unfortunate that the medical profession has almost been blamed for somethings? In terms of the nosocomial spread, and things like that. I think also just from a primary care perspective, it's been really, really difficult, I think, particularly in the last six months, seeing the number of delayed diagnoses, um… yes definitely, the kind of the cancers and that, and also seeing people's treatments for cancer being altered, so not having chemotherapy, you know, potentially living with a life-limiting cancer rather than a potentially curative cancer, also I had patients who… who have Chron's, or I've got one patient who has a really- really high marker for inflammatory bowel disease, who's been waiting for six months, has lost their job, is now kind of mentally unwell, is almost suicidal, and the reason they haven't had an appointment, haven't had a scope yet, is obviously the Covid-19 burden, things like that, um, and I just think-looking- retroscope is a great thing isn't it, but I think we kind of did see it, I think it would've been nice if the government did take note of what we were saying, and yeah, and it just feels, I think, with this- the talk of opening up again now, I think we're all a little uncertain about that, because we just know how much illness, non-Covid illness, and also long Covid, and how on earth are we going to catch up with all of this?

M: How have you managed patients who I assume are worried by delayed referral times? In terms of communicating with them and also comminating with secondary care? I understand that some GPs have been taking on new roles to try and cover for waiting times, have you had any experience with this?

GPTr6: So, um, we have had an awful lot of patients coming in with a mix of either, you know worry and anxiety, um, feeling sort of under pressure by their employers, also quite a lot of anger as well about waiting times, so I've increasingly spent a lot of time, proportionally, a greater time, trying to contact secondary care, um, both in terms of sending follow-up letters, you know sometimes just three or four follow-up letters in the system, trying to get, you know, either update them on their condition, whether they need to be triaged higher, or just to let them know about things. I've had letters back from secondary care saying- you should not tell patients who have been referred on a 2-Week-Wait or a Rapid Access Cancer Pathway, you should not tell them they'll be seen soon, because they won't be. Which is really hard, to speak to cancer patients. And also I think, we spent a lot of time just trying to deal with the emotional aspect, people being incredibly frustrated, even them going to like their surgeons, secretaries, saying- no, none of the surgeons are operating at the moment, we can't tell you when you're going to be seen, and then also dealing in primary care with a lot more things secondary care would be dealing with? Which is like, an extra bit on my learning curve as a GP. So I suppose the additional roles I've taken on, I suppose it's been more admin? It's been more, kind of, um, follow-up letters but also hospital referrals or admissions, because we find that patients really do just deteriorate over the months? And also just taking on, um, perhaps more of a counselling-coaching role for patients, trying to cope with these waiting lists and the uncertainty.

M: Yeah, thank you for that answer, covered a lot. It must be very difficult having to be the person passing on these tough messages of waiting times and so forth. Thank you for explaining that well. It's a more sensitive question- has Covid had any impact for you personally?

GPTr6: So it's had quite a big impact in terms of, um, so my sister and her family live in America, so I haven't seen them for 16 months now, and I actually have a niece who's 18 months old and I've only seen her once. So I've just seen her grow up over Facetime really. And my sister's definitely my best friend, so that's been really hard, and also kind of not being able to help her out with my little niece, who's quite a handful. And then also kind of anxiety, so I've had to completely distance from my parents, and I've also made a decision to distance myself from a lot of my friends, particularly my non-Medic friends? Um, particularly when the information started coming through that, you know, people in their 40s were dying of Covid or getting really unwell, so I haven't touched my parents in a year now, um, I haven't been kind of inside a building with them, probably since- I think since the numbers started to increase again, so probably October time. And I haven't seen a lot of my non-medic

friends for almost a year, um, one of my best friends had a really difficult time, so she miscarried last spring whilst she had Covid-19, and then got really, really depressed, um, and I couldn't see her with the various lockdowns and things like that. And now happily she's 32 weeks pregnant, and she's been anxious, and she's been working in A&E whilst- and with the lockdowns, I still haven't been able to see her, and I now also feel that I'm more of a risk to her?

M: So how are you doing, protecting all these people, how are you doing yourself?

GPTr6: I think it's difficult, you have to kind of find different ways of coping, and possibly for one of the better- coping with a kind of interim level of wellbeing? Not necessarily, like a low-level of wellbeing, but just accepting that you can't… I think there's been a lot of- it's been good, but I think there's been a lot of sort of promotional things over the last year about, you know, make sure that you look after your wellbeing, maximise your wellbeing, does this do that. And I think it's difficult to aspire to optimal wellbeing in these, kind of, conditions, particularly when you know that none of the things you derive your wellbeing from, you need to, kind of, put to the side for the moment. So it has been difficult, I think it's been more… everything's been more effort, so I'm doing a diploma this year, um, and… I'm involved in, like a trainee committee and things like that, and it's been quite interesting in just that it's more effort to apply yourself, it's more difficult to apply yourself to the degree that you need to sometimes, and I've gradually accepted that I can't quite do as much as I would usually? I'm usually just one of those people that wants to do something and I get twitchy if I'm not doing anything, but I kind of expected that, you know, there's only so much that I can do at the moment, you know, yeah. I spend a lot of time talking to my cat, I'm very lucky that I adopted a cat in February last year, so I spent a lot of time talking to him! (*Laughs*).

M: I miss my cat so much. I um, you know you're one of a few GPs who've told me they've adopted a cat in the last year! It seems like the right time to have that extra bit of company I think. But, yeah. Um, are there any changes you think should be carried on into the future, have there been any positive changes? And equally, have there been any negative changes, anything you think we could learn from this time?

GPTr6: Hmm. I think in terms of positives- I think the- the kind of the way that things have evolved over time has been interesting, because I think actually the way my practice uses now AccuRx, so I think it works really well for kind of skin conditions and things like that.

M: The photo texts?

GPTr6: Yeah. Having photos and then having them on the system. Then… I think there could be a great potential-capability for using those photos to use an e-consult with secondary care dermatology, I think that could work quite well. And also, having the facility to, um, txt patients with information guides and things like that, going along with the concept that patients are- anyone can only take in a finite amount in a consultation, but then giving them things to over time- drip-feeding things, I think they found that really helpful. Um, I think an element of telephone consultations, I know this is contentious, I think it's good, and I think some patients prefer it because they're very busy, they don't necessarily feel they need to see a doctor, if it's something really simple, the risk is much lower than other things, and it's more convenient for them, they don't have to take a whole day off work. Which I know is really great, because a lot patients have said- in the past I would be disciplined for taking so much sick leave in a year, simply for coming to GP appointments.

M: Really.

GPTr6: Um, so that's worked really well.

M: Ok, I'm glad to hear it's successful for some people then.

GPTr6: Um, I think in terms of negative things… I think… I think- well my practice has been better than most practices in the area, but I think the loss of some of the kind of essential services, which have been put to one side? So all of the access to different contraception methods and things like that I think, is something that's been quite negative for patients, um, definitely I think the level of telephone consultations isn't a great idea, um… and… I think… I think those are the main things, because I think a lot of the other things are again positive. So the health ward have set up a consultant-GP telephone service? So if you want to get advice, instead of kind of waiting on the phone for 20 minutes for the poor med-reg to speak to you, you can kind of, um, phone a dedicated consultant in that speciality and get some advice, and kind of keep someone in the community? Which is the whole, kind of, aim I think of general practice, especially in my area, is trying to keep people where they want to be. Um, so that works really well. Um, yeah I'm trying to think of the other things. I- I think perhaps the other thing which hasn't been good in the pandemic is at some points when it's got really tense, I think the clinical leadership, um… hasn't been great in terms of compassion? So lots of people have found the emails sent from the clinical director quite affronting at times? Particularly when colleagues were concerned about PPE and things like that, um, they- there were a few quite- there was one where the email was quite sarcastic? So I think, kind of, greater equipping clinical with more actual leadership skills and a, kind of, leadership ethos and things like that, um, would be much better.

M: I'm really sorry to hear that, you would hope that you'd be supported by the people leading. Do you get most communication from your CCGs, or do you have a Primary Care Network?

GPTr6: Yeah we have um, so essentially we have kind of the health board of clinical directors, um, and then, essentially they kind of, the practices get their commissioning from the health board, so it kind of comes from that way. So they're the people I would say are most senior, but also most separate from primary care? Because they're not essentially working for primary care in the region.

M: That's interesting. Thank you. Is there anything that I haven't covered that you'd like to talk about, anything that's been important to you in the last year?

GPTr6: I think in terms of, I don't know whether it's helpful for your study, but in terms of being a GP trainee, um…

M: Yeah I wanted to ask how it had affected your training?

GPTr6: So I think, because so much of GP training is based on human connection and building human relationship between your trainers, I think that's been quite affected, um, so they haven't gotten to know me as well, I haven't gotten to know them as well, the kind of opportunistic learning hasn't been as good. Um, and I haven't, for instance, got as many workplace assessments and things like that. Also the kind of experience has been different. Obviously it's been very much, kind of, telephone-based in the majority, um… I haven't had as many home visits, and the kind of things I'm seeing are very different in terms of there's less of the kind of chronic long-term care management at the moment. In terms of the learning per-se, we've been really lucky in our region that are our vocational training scheme- our training programme directors are amazing- they're completely – it's like a completely flattened hierarchy, their response- they're really open, so we have kind of weekly VTS schemes, uh, teaching sessions, and they'll kind of open it asking how we are, and they'll say, kind of, things they've had difficulties with this week? And it's not stilted at all, it's really, kind of, human which is really good. And they're really approachable. Um, it took a long time to get teaching back up and running, so we had a few months of no, kind of, formal teaching or anything like that, so I did a lot of trying to find my own, kind of, virtual learning and things like that, um, and then they bought a virtual learning package for us which was good, and then they started to do things online. Which I think- possibly I'm a little bit different from other GP trainees, because I quite like doing this online, because there's less distraction? There's not, like, 20 other people in the room, eating their sandwiches *(laughs*).

M: A lot of trainees say the same thing, that they quite liked the shift online! But also a lot of people say they expect it to be contentious, to say telephones are convenient, um, but I haven't heard a single person not say it yet! So it will be interesting to see, overall, I guess it also depends on the age, being younger maybe it makes it a bit easier to use the online packages? Um, has it influenced your views on general practice or your decision to go into general practice?

GPTr6: I think… I think, um, I've been quite surprised this year, so essentially when I moved from general anaesthetics quite a few people said- what on earth are you doing, (*laughing)* you won't last, GP is awful, it's so stressful, you won't like it at all. And I think I've just been- because I'm someone who, I think, hopefully is quite realistic, I was expecting to have some doubts? And I've been really surprised with the complete lack of doubts I have.

M: Oh, lovely!

GPTr6: I think it's really reinforced how much I want to serve a community, and how much you can do that as a GP, and how much potential influence you have on making a difference with every single contact? And it doesn't need to be a prescription, it can just be listening to someone, or doing a bit of motivational coaching, and also GP is just so diverse, and I think we've really seen that there's diversity in the clinical and social presentations, but also in the ways that we're working. Um, and it's been great- I think there's been a great comradery, even though it's been virtual, in terms of the MDT working, and just- I think I'm lucky because where I work the patients are generally really nice, they've still got that kind of traditional, old-fashioned, appreciating-the-doctor kind of thing? Which is completely different to the practice where I worked in **REDACTED city name** which was in **REDACTED area name**, um…

Both laugh.

GPTr6: Yeah, so it's completely different. And I think, yeah, just it's made me- I think Covid-19 has also made me realise just how responsible I am as a Doctor, um, in terms of- sorry, how much responsibility I have? In caring for the community and in how I live my life, has an impact on patients, which probably sounds a bit dramatic, but I think it (*unintelligible)* more than in a hospital, you get more human connections.

M: That sounds completely reasonable to say! Thank you so much for that answer.

Recording ends
