# Supplementary material for: Primary health care during the COVID-19 pandemic: A qualitative exploration of the challenges and changes in practice experienced by GPs and GP trainees
Source: PLoS One. 2023 Feb 9;18(2):e0280733. doi: 10.1371/journal.pone.0280733 (PMC9910752; doi:10.1371/journal.pone.0280733)
Supplement: S1 Data — (ZIP) [file pone.0280733.s005.zip › GPTr8 Transcript.pdf]

GPTr8 Transcript

Interviewer: So to start, please could you tell me a little about your experience in general practice pre-pandemic, so the practices that you work in etc?

GPTr8: So pre-pandemic, uh… and I would say post-training, so after medical school, I got a 4-month post in F2 which I had done in **REDACTED location name*,* which was a fairly… you know, white middle-class area, um, predominantly, and I had a great time there, it was quite well-sheltered and I didn't have that much independence. And then probably more recently in ST1 I was working – I had four- six months in a practice in **REDACTED area name**, again like, well, bit of a mixture, so mixture of middle class and then pockets of quite deprived areas? So we looked after a couple of fun travelling communities and trailer parks and that kind of thing, so a bit of a mixture, and… yeah, I- I've always really loved GP, um, I've always really been drawn to it as a specialty, because I like looking after people in their own environments, so in communities rather than in hospital where I felt like it was a fairly artificial experience. Yeah, particularly in ST1 I was kind of starting to get into the swing of things, I got my appointments down to 15 minutes and it was kind of like a back-to-back surgery in the way tat you would expect a GP surgery to run.

Interviewer: Great, so are you in one practice now?

GPTr8: Yes, so I work full time, so that's… seven… eight? Nine sessions in a GP surgery and then we have a half day of for self study. Which is why I've got time to talk to you! (*Laughs*)

Interviewer: I appreciate you using your time for this!

GPTr8: No, no, that's fine.

Interviewer: So could you tell me about your experience of Covid-19 professionally?

GPTr8: So, um, it's been a big shift towards, um, remote consulting, so I really didn't have much experience at all of telephone triage because it wasn't something that was a big feature of either of the practices I'd worked in before? Um, so, at the moment the way our surgery runs is every single person is telephone triaged by a GP or a doctor, and it's then still decided whether or not that person needs to come down to be seen, or whether it's something that can be dealt with over the phone, um, and that's the way I believe this surgery has been run since March of last year, since everything started, and it's certainly the way we're still running at the moment. So that was a big shift.

Interviewer: Mm. How prepared did you feel for the pandemic?

GPTr8: I think my surgery has done really well in making sure that I adapted well to telephone triage, um, you know I had a good, like, introductory period where my supervisor was sat in with me and was listening to some of my consults before I was, sort of, let loose on my own, you know they made sure that I had time to debrief after every surgery and go through all of my plans, and say vaguely, you know, what was going on, what wasn't going on, what my plan was going forwards and they kind of really supported me to make sure that I felt safe? Um, and I think it also helped that when I started in August, they had already been doing this in the surgery for- since March? So they had gotten a little bit more used to telephone triage and things and they could ease me into it easier, and I think they probably felt quite overwhelmed when it all just kind of hit in March last year.

Interviewer: Ok, did you have any protection in terms of PPE, and also sort of social protection, any guidance?

GPTr8: Yeah, so I mean our surgery is quite a social one, you know, we really value our coffee time and being able to sit down in the surgery after the morning, um, finishes, and I think we worked really hard to make sure that everyone was sat 2 metres apart, everyone was wearing masks, but also to really try to preserve that social interaction, that was something was really important for us. And I know that there are other trainees in other practices that, you know, it's easy to feel isolated in GP as a job anyway, if your surgery is not the kind of surgery that does that and I felt that my surgery was really important to maintain. I'm also lucky in that my husband is a doctor as well, he's an A&E trainee so I could sort of come home and decompress about stuff, and he had the option to do that with me as well. Um, I think if I was living by myself, that would have been really, really difficult, in terms of not being able to see people.

Interviewer: Completely, yeah.

GPTr8: In terms of PPE, um, we had access to a couple of FFP3s if we really, really had to, um, we had face masks, we had aprons, and gloves, um, we never really felt like we were running low at any point, but I think there was a certain worry that, you- you know, that wasn't necessarily adequate, if you were seeing someone that actually had had Covid, but that being said, that's the same across the board whether you're working in a hospital job or not, I know that that was the level of PPE you were given, you know, unless you were actually doing an aerosol-generating procedure, so as far as what we had compared with everyone else, it was fine.

Interviewer: Ok, well I'm glad that you had some protection then. How informed did you feel your patients were then, about the pandemic, in terms of, um, understanding the new policies and why they were having phone calls?

GPTr8: So I think, you know, in many ways a lot of our patient demographic have benefitted from being able to do remote consulting, you know a lot of our families are young working families that have children to look after, and, you know, for them actually physically making it to an appointment can be really tricky sometimes? And I think some people really benefitted from a bit of a shift to remote consulting, um particularly if it was something that could be easily dealt with over the phone, like, you know, contraception checks don't necessarily have to be in the surgery or something like that, um, so from that point of view I think some people did benefit. Um, the demographic that really didn't I think is probably the elderly, the frail, comorbid population, I think there's been a lot of times when I have essentially had to convert to a face-to-face because I just felt like that person was not able to communicate well over the phone, um, just- and I think their- their perception of the extra barrier to care has really impacted them as well. You know, really felt like they haven't been looked after properly? Like- how can you give me a course of antibiotics if you haven't even seen me, doctor. You know that- that shift for them I think has been really difficult.

Interviewer: How do you manage those situations where the patients are frustrated?

GPTr8: I think you just have to reinforce that at the end of the day you're not keeping patients away because- just for fun, it's not because we're trying to shirk work, or it's not because we don't want to see people, um, you know, particularly for those frail elderly people, you know before the vaccine roll-out particularly, and when we were in particularly bad spikes, it was dangerous for them to be coming down to the surgery, and it was an additional risk. And obviously there's the risk then, you know, of not seeing the person and missing something, because you haven't actually seen them face-to-face, but there's a completely calculable risk in bringing them down to the surgery, particularly with people who are meant to be shielding and don't leave the house for any other reason? Um, so I think, particularly when people were starting to get angry, I'd try to bring it back to- listen, I'm not doing this for fun, I'm not being lazy, like, I'm doing this to protect you at the end of the day, and I've, kind of, got your best interests at heart. Most of the time people do kind of deescalate in response to that kind of comment.

Interviewer: I'm glad to hear you've sort of developed your response in response to those patients. How did you feel making decisions for your patients when you had probably limited information about- I'm assuming you had limited information, it sounds like the guidance wasn't really there?

GPTr8: Yeah, and I think… (*sighs*) I think at the end of the day it- it- the- the decision that we make have- have fundamentally altered- you know I can think of several people that I didn't send in to hospital or didn't- didn't phone the hospital for a review because I felt that if that person went to hospital and got Covid they had a very high risk of dying, and you know you really had to- I know- I know that hospital acquired infection has always been a factor in decision making, but for it to be a real risk- particularly for the people who were shielding, who weren't having any other type of exposure, it became a big factor in whether the risk in sending them to hospital was worth, you know, whatever you- you thought you were balancing against? You know, I had a couple of people who were potentially surgical abdomens, or you know they were clinically well and it could have gone either way, and I think at that point it's just about having a really frank discussion with your patient and saying, you know I think- I think we can watch and wait. You just have to have a really low threshold for changing your mind out of hours, phoning us back, we'll keep an eye on you obviously over the next few days, but I'd never forgive myself if I sent somebody up for a query appendicitis which didn't turn out to be appendicitis, they then got Covid and died. You know, it- it completely fundamentally changed your risk analysis.

Interviewer: That's so much responsibility, to consider that as a consequence every time… that sounds really difficult.

GPTr8: Yeah. Yeah. I mean I'm really lucky, I've got- I've got a lovely supportive practice, I've got a great supervisor, you know, it- it's quite a culture in our surgery to grab somebody else and run it past them, even amongst the other GPs? You know, they've all kind of got special interests and things like that, the patients are all really used to their doctor running into another room like, can you just come and have a look at this. So particularly for trainees, that's a really nice environment to be in, because you really feel like you can field queries and share the responsibility a little bit, and- and get an outside perspective as well.

Interviewer: Great, thank you. Thank you for that answer, very honest, and um, yeah it- it makes me think definitely. I would ask- so you talked about in terms of changes to care, you've had telemedicine, have you had any new roles as a GP? Be that in responsibilities from secondary, vaccinations… as some examples, but any changes you can think of?

GPTr8: From secondary care… I think nothing that has been handed over directly, like I personally haven't had any letters back saying- we would normally do this but we're asking you to do it instead, in light of the pandemic. Probably more so what we've had is like indirect… indirect consequence of people not being seen? So I think we've been managing things for a lot longer, because that person is waiting for their routine appointment which has been putting off and off and off, um, there's a lot of like dermatological stuff like severe eczema, severe psoriasis, that you would kind of hope would be seen sooner, but, obviously you know a lot- the cancers are still being prioritised, that kind of thing is still being seen, but all of the stuff that is kind of routine, that probably can wait, but still has a huge impact on that- that patient waiting months and months to be seen. Severe eczema is

a really nasty thing to be sat with if you're absolutely covered in it, and that kind of thing I think we have been managing- we have received a lot of burden from people. So I think dermatology is a big one, I mean, mental health- mental health is huge, um, you know I think we're- we're seeing a larger volume of both mental health and functional symptoms at the moment, just like, a factor of… you know, people's lives are a lot more stressful, they've not got anything to distract themselves, they've not got the social interaction that they usually do, so I think that volume of stuff has gone up anyway? But we've also had unfortunately our local mental health services have closed down a lot of what they're able to do, they've not- from what we can see anyway they've not replaced their clinics with telephone appointments, so a lot of people have been shifted to us, and we- we feel like we're managing a lot more than we usually would. So yeah, to answer your question, probably nothing has been handed back, per se, but I think we're managing a lot more, just because the hospitals have had to reduce their- their service.

Interviewer: Ok yeah, makes sense. Difficult but makes sense. Has it changed your relationship with your patients, fielding their enquiries by call, and…

GPTr8: Yeah… I mean I think it's- it's hard to say really, I think, for… I'd like to think that if I'm not really not winning with someone over the phone, I just bring them down, um, and, you know that's obviously a risk-benefit weigh-up every time, but… if I feel like something is really stilted over the phone- you know, I've had a few examples, particularly people that are phoning up with something that sounds quite functional, and then you bring them down and they burst into tears in front of you, and actually like it's a mental health, or it's something going on in the home that they can't talk freely about when they're actually in their house around the situation. So I'd like to think that if I feel like I'm not establishing good rapport and I'm not getting a sense of what's happening over the phone, I tend to bring them down, that being said, the worry is the sort of unknown-unknowns, the cues that you sort of haven't picked up on, and I definitely think there must be a lot more of that than we realise.

Interviewer: Yeah, um, yeah that is… we'll see I guess, but um, do you feel comfortable when you bring patients in, are you alright with that?

GPTr8: Yeah, we've got a select minority of people that refuse to wear masks, which is not ideal, we've got people that either don't believe in Covid or say they can't wear masks for… I would say tenuous reasons. You know people that- I think, you know, you always have to make allowances for people that have got, for example, people that have got PTSD or people that have got trauma related reasons or, you know, they've been threatened or asphyxiated in the past, I think you obviously have to make allowances for that, but we've got a fair few people that are probably like the lanyard-wearing people, who could probably wear a mask but just don't want to, or are Covid-deniers or something like that…

Interviewer: That must be very frustrating.

GPTr8: … really difficult, and I think at the end of the day it's something that any patient- because we don't (*laughing*) unfortunately you don't always like your patients, that's just the way it is, um, you just have to try and manage them all the same, because they deserve care as much as everyone else does!

Interviewer: I appreciate your honesty all the same! *(Laughs)* Yeah, it must be very frustrating to have somebody who is a Covid-denier, when you're working with Covid, I can't even imagine!

(Both laugh).

Interviewer: Has it changed you relationship with your colleagues? That could be a positive change.

GPTr8: Yeah, I think um, within my surgery probably no, I think we're quite a close-knit surgery and like I said we make an effort to decompress and talk to each other a lot, I think that the main difference for me as a trainee has been that usually every week or every other week we would otherwise be going down for central teaching every Wednesday morning, and that's obviously not- not happened at all since August. We've had a fair few teachings on Teams and small group teachings, where you kind of log on every Wednesday morning and see everyone from that point of view, but it really sucks not ebing able to see other people who are kind of on your peer level, particularly when you're all siting exams and things like that, you know just not being able to interact with other people who are at your level. There was a lot of, kind of, sharing concerns and experiences and anxieties with people that we got in ST1 that was really healthy, you know just kind of hearing that everyone else was feeling exactly the same way as you know, you were feeling, was really nice? And as much as we try as best we can to replace that with Zoom and Teams, it's just- it's just not the same. Um, you know, I remember in ST1 after a small group teaching we would often-because we would often be in somebody's house, we would just always bring food and sit down and have lunch and stuff afterwards, and that kind of socia interaction I think is really missed.

Interviewer: The person I was interviewing just before you was another trainee, and they said the exact same thing. And it was the first time I'd had it raised. But yeah they said the same thing, and it sounds a little bit isolating. From the people that are at your level, I'm sure you've got other people from other levels in your team.

GPTr8: I mean I'm lucky because as an ST3 I know people from ST1 and 2, I can't imagine what it's like to have moved into an area, just started GP training as an ST1, and literally never having met anyone face-to-face who's at the same level as you, like that must be awful.

Interviewer: I wanted to ask, so you've mentioned this, has it affected your training in any other ways, would you say?

GPTr8: So, I mean obviously the exam I think is probably the biggest one, so as an ST3, I don't know how much you already know about GP training?

Interviewer: Yeah, uh a fair amount from these interviews!

GPTr8: Yeah so I've already sat my knowledge exam, so that wasn't a problem for me, I know people that have had to delay their knowledge, the first stage, which I think has been a real pain, I… still have to sit my, what would otherwise have been my CSA, face-to-face OSCE, but they've replaced it with a recorded assessment.

Interviewer: The RCA?

GPTr8: Exactly, yeah, and I'm actually the first trainee in my surgery to sit it, because the girl that was there before me last managed to get in for the last sitting of the CSA before they closed it in March! *(Laughs*). So, it's been a… that I think will have an impact on training, because it's just a completely different exam, and I'm actually finishing my submission next week, so I have no idea whether it's gone well or not! *(Laughing*)

Interviewer: Thank you for your time when you're in the middle of it!

GPTr8: Oh yeah, no, it's fine, I think I've got enough anyway, it's just that's… that side of it has been completely different ad I think the college has actually done quite well in adapting to all that. There are people who criticise the college, and there are parts of it to be criticised, but I think the adaptations to the exam have been pretty good and they've rolled it out really well, and the software we use is really good. Um, the other thing I'm worried about in terms of training is I have never had to do a back-to-back face-to-face surgery to time, so with 10 minute appointments, because the last time I had to do that I was an ST1 and I had 15-minute appointments, so I'm managing it at the moment with a telephone surgery, I have no idea whether I'm ready to consult at 10 minutes, you know, back-to-back 13 patients in a row, I have no idea whether I'd do it to time, whether I would cope, and who knows whether we're going to get back to that point before August. So I think that's probably my biggest concern at the moment.

Interviewer: That's reasonable, um, has it changed your view of GP practice, is this still something you want to go into?

GPTr8: Yeah, 100%, like I've- I've never been happy in any other hospital job that I've done, like the only time- and my husband says it as well- when I come home from work, I've ever felt like I've had a good day, or actually had a fulfilled day, or even enjoyed what I was doing, is when I was on the GP job, so 100% hasn't changed my mind. Um, I… I think, you know, from a- from a practice point of view, I don't think it's harmed the surgery to have to move a little bit more into telephone triage, and I think they're likely to keep at least a small aspect of that, because I think there are so many things that can be dealt with over the phone quite easily. Um, but, they will need to start making a move back to face-to-face, I think, as things open up.

Interviewer: Ok. Um, slightly more contentious, what is your opinion of the government response to Covid, in terms of the public health messages and policies, um, I'm sure you're- as a frontline staff, I'm sure you're fielding a lot of queries from patients.

GPTr8: Yeah, um, and it's interesting for me personally as well because I've got family that's pretty much all over the world. So my closest family, so my parents and my sister, they all live in **REDACTED country 1**, um, my sort of extended family beyond that, my mum's side are in **REDACTED country 2** and my dad's side are in **REDACTED country 3** so I've got quite a good global view of what's happening everywhere else! And it's made it so much more difficult to watch because everyone else's borders closed down so much more quickly, and I've got friends in **REDACTED country 4** who basically said, like, when there were two people that tested positive in their building, their whole building locked down, the government was at the door, delivered everyone PPE and cleaning supplies, really clear advice about what to do, and you know, I appreciate that we have a different scale to **REDACTED country 4** and to **REDACTED country 1**, don't get me wrong, but you know, we- my parents managed to come over and see me in September time, which was kind of just the golden window, the only time they were gonna be able to see me, and you know my parents I'm pretty sure had Covid at the beginning, um, as did my sister because she works in- as an ITU nurse in **REDACTED city**, and we think she probably gave it to them, but they had very classical symptoms, were ill for about, I think it probably took them about four or five weeks to recover, um, and… they're both like fit, well, 60-something year-old people, who they got quite a shock. That was probably the only reason I was happy for them to come over in the first place, because we kind of thought- well they've already had it, so they're not too much at risk, but they're both sensible, well-educated people- they were given literally no guidance when they flew into the country, it was like- okay, so where are you self-isolating? And they weren't really told what self-isolating means, nobody checked up on them, it was just- I couldn't believe it, and they obviously went back to **REDACTED** and they had people on the phone to them within the two weeks to check that they were actually staying within their house, and all of this- completely different- and all of this stuff about them imposing the hotel quarantine now? (*Laughs*). I mean like horse has already bolted guys! Long, long time ago! If you were going to do that, you needed to do it back in April, when we started seeing everyone flying back from their skiing holidays in Italy, I mean it's mental that we're doing it now. And we still see it- we had someone who walked into the surgery today, who had a- a son who had flown home from, um, Bulgaria to stay with his relatives, and had literally been in the country for less than a week, and showed up at our surgery with a registration form, and the receptionist is like- you're not meant to be here! You're meant to stay in your house and not have gone anywhere! And he had literally no idea.

Interviewer: Right.

GPTr8: So it's- I think it's obviously- the higher level implementation, like the hospital- the hotel quarantines, blah blah blah, and that kind of thing, not ideal, um, but also just the actual dissemination of information has not been brilliant.

Interviewer: Yeah. Thank you, that's a great answer, um, lots of comparisons drawn. Yeah thank you very much. This is a more sensitive question, so answer it as you wish, but has Covid had any impact on you personally, um, as a trainee going through what I imagine is a very stressful year?

GPTr8: Yeah, um… so I- I was meant to get worried last July! (*Laughs*). So we, we had our big wedding planned, and um, you know 150 planned to attend, and we obviously had to postpone that, but, you know, we were really lucky to get that tiny window where we went ahead with both sets of parents? So at that point we were allowed to have, I think, six people in a room, so we had literally- no, it must have been more than that at a wedding, I think you were allowed to have 20. Um, so we had literally both sets of parents, me and **REDACTED partner name** and our celebrants, and that was it! *(Laughs*). But, you know, we had postponed to this June, and we have to postpone again until 2022, so for us, at least we managed to get the marriage bit out the way, and we can have a big wedding, you know, further on down the line.

Interviewer: Yeah, without the stresses of the actual marriage!

GPTr8: Exactly, and you know there were so many things on the day, like, that I could get into, that I'm so glad I don't have to worry about! *(Laughing*). Now I can have a big wedding when it's a-go!

Interviewer: I hope it's everything you hope for and better!

GPTr8: Yeah and I think from a personal point of view, I think my husband and I, I think have quite stressful jobs, and we've always really hung onto, like, our little holidays? Like the little pockets of leave that you can get together and actually get away, and I think that's what we've really missed. On a personal level, it's just difficult to deal with a hard job with, like, nothing to look forward to.

Interviewer: I really hope that, obviously as everyone else does, I really hope that soon this starts to raise and you can do those things again. Hopefully soon.

GPTr8: Fingers crossed!

Interviewer: From what we've talked about today, are there any changes in general practice that you could see, or would like to see carried on in the future, and if so, how? And equally are there any that you would not like to see carried on!

GPTr8: So… I think- yeah I've obviously said this before but I think that a degree of remote consulting is useful, um, I think there ae some people that take really well to video consults and phone consults, particularly people who are working, or people who have childcare issues, or people who are young and don't necessarily need to come down to the surgery for whatever the problem is. So I think they're- I think personally we should retain a little of that. I know there are some surgeries who have a

very high volume lists that have always done telephone triage? And I think that works well for some certain populations. But it's just, it's a problem of access, you have to make sure that you're making it fair for everyone, and everyone knows how the system works, and that it doesn't disadvantage some people over others? Um, things that I wouldn't want to continue, is probably the obvious stuff, just like access to secondary care, so I think our- our routine stuff that we're referring, or investigating, you know I really miss being able to just give somebody an X-ray card and saying, just wander down there and get an X-ray whenever suits you, like having to wait for an X-ray to come through has really impacted what we're able to do. Um, you know, particularly for people that might have fractures or something, isn't always appropriate? You know, for something that might have happened a little while back, um, they would otherwise just be able to walk in and get the X-ray and come out. Instead you're having to expose them to all the people in A&E and you shouldn't really have to do that, so, um, it's- it's probably a fairly obvious answer but yeah, all that stuff, I can't wait to start getting a little more access back.

Interviewer: Great, thank you. Um, it's not super obvious- I think that's a really nice answer. And hopefully this research will go somewhere, and it will raise- because you and lots of other GPs have similar hopes for the future, so I hope that this goes somewhere. Um, what do you think we can learn from the pandemic, thus far, in general?

GPTr8: You know, I think it's similar to what, like, for example doctors in Africa probably say about post-Ebola, you know, you hope that we have learnt how to quickly roll out a Test & Trace, or a vaccine programme, or PPE, or when you should be locking down your borders or quarantining! *(Laughs*). You know, I think just the pandemic-readiness aspect of it? I, I really hope that we, or like the government, have learnt how best to roll that out, and, you know we don't know now if the vaccines are all going to be great, or if this is going to be the end of it, or whether we're actually going to continue to have mini spikes every winter over the next couple of years, like, we just don't have that information yet, and I really, really hope that the actual pandemic-readiness of the country has just got better, hopefully, fingers crossed! *(Laughs).*

Interviewer: I've eaten into your time little bit, so is there anything that we haven't spoken about today that is important to you that you would like to raise?

Pause.

Interviewer: We've covered quite a bit I think.

GPTr8: Yeah… I think we've pretty much covered it all, yeah.

Interviewer: Okay, great, I'm just going to stop the recording there.

Recording ends.
