# Supplementary material for: Primary health care during the COVID-19 pandemic: A qualitative exploration of the challenges and changes in practice experienced by GPs and GP trainees
Source: PLoS One. 2023 Feb 9;18(2):e0280733. doi: 10.1371/journal.pone.0280733 (PMC9910752; doi:10.1371/journal.pone.0280733)
Supplement: S1 Data — (ZIP) [file pone.0280733.s005.zip › GPTr9 Transcript.pdf]

GPTr9 Transcript

Interviewer: Could you tell me a bit about you experience in GP care, and tell me a bit about your practice maybe?

GPTr9: Um, so I've done the whole of my GP training less than full time. I started training in February 2015, before my second child was born, and like I say I've done all of it less than full time. The practice that I work in, the area that I work in, **REDACTED area name**, is kind of an ex-mining town, a quite socially deprived area, high rates of smoking, and very high rates of obesity, um… yeah, kind of difficult demographic in lots of ways, um, some problems with sort of reading and writing, and things like that as well, um… but um… not so much any problems with sort of intravenous drug use or anything like that, um, less so with that sort of things… um… so yeah, and that- so the practice that I work at, we've got about 9 and a half thousand patients, um, so it's kind of a medium sized practice, so yeah, that's a bit about that really.

Interviewer: Cool, and are you part-time now?

GPTr9: Yep, so I still work 60% 3 days a week, and I've done that all the way through, apart from in the hospital where they wouldn't let us do 3 days a week, we had to do 50%, so 3 days one week, 2 days the next week, and I had a job share so we kind of shared it equally between the two of us.

Interviewer: OK, cool. Um, so, to start, and it's quite a big question, but could you tell me about your experiences of Covid-19 professionally?

GPTr9: Hmm, so, when I was in the pandemic obviously last year, I was in a slightly different practice, I was at a practice called **REDACTED practice name 1**, which was in the same PCN, but it's about half a mile down the road, um, similar set of patients, um… but, um, it was a much nicer practice? (*Laughs*). So the team there was nicer I felt, and I felt a lot more supported, and we… when the pandemic first started, we introduced- or rather our partner introduced- a coffee break, which we never had before because we were too busy. So then we'd have a coffee break at 10:30 where all the doctors were socially distanced but we'd meet in the um, meeting room, which was quite big, so we'd all kind of sit, sort of 2 to 3 metres apart from each other, and just sort of talk about how we were feeling, about, um, kind of worries, um, and we also used that time- because at the time NHS England were sending out all the shielding letters, and we were having a lot of problems with that, because some were inappropriately coded, so we had time to sort of bring those kind of Covid-related queries, but also just to sort of check in with each other? Which was quite nice.

Interviewer: What did that sort of do for you and your team?

GPTr9: Um… It was really nice because I think it made us really bond more as a team and I really felt like I was being cared- like people really cared? And that is really important, um, I feel as any person, working in any job, whether you're a doctor or anything, but to really feel that the people you were working for really care about you as a person? Um… and so that was nice, I felt like we were all there, we'd just talk about, you know, the weekend, and obviously you can't really do anything, or, you know, stories from people's families or whatever, and sometimes we'd bring cakes in as well, um, especially on Wednesday, so we had cake Wednesday, one of the partner brought a cheesecake, and then I made some cakes as well.

Interviewer: That's a very nice feature. That sounds lovely. In your second practice you said the support was different, how was that?

GPTr9: So the support different in the fact that um… because I'd been at the practice- I'd never been at **REDACTED practice name 2** before, um… so I did a stint there just at the start of ST3, and then I went back to **REDACTED practice name 1** um in August of last year, and I'd been to **REDACTED practice name 1** before, so it was kind of, it wasn't great, I felt like there was a lot of pressure, um, to… It felt like a lot of the face-to-face was dumped on the registrars and none of the partners saw any face-to-face, because you know, the partners had to protect themselves, and the registrars was basically- and one of the partners even said to me, 'Oh I know it looks like you might be seen as cannon fodder', that's literally what she said, 'but that's not our intention'. And I was like oh, that's what it feels like! (*Laughs*). And myself and another trainee, who had both been at **REDACTED practice name 1** and had both moved to **REDACTED practice name 2**, both had some time off with stress, I had 2 weeks off and she had a month off, and neither of us never had stress off GP training or anything like that, so we both had some time off. So I think it's very interesting how being at one place during the pandemic, and then moving to another place, when actually in August, the pandemic, some of the stuff was at a bit of a lull… was actually not nice, um…

Interviewer: It seems like the environment has a massive effect on your experience.

GPTr9: Definitely, definitely.

Interviewer: It sounds like you were in a bit of a hot-water situation?

GPTr9: Yeah, but you know I got through it, and I'm still there now. You know I don't really love it, but I think it's a means to an end. And I just think, well, I've learned what kind of place can be good, and what kind of place can be supportive, and I just kind

of think to myself, well when I hopefully get to that stage and, if I ever do have any trainees, I feel like I'll have more insight about how to support them and how to basically be kind to people? (*Laughs*).

Interviewer: Learn from your teachers' mistakes!

GPTr9: Yeah… that's what I think, yeah.

Interviewer: When you, if you don't mind me asking, when you took the weeks off for the stress, that was in August I think you said, how was that? Did you feel-

GPTr9: So I took it in October I think. My colleague, she- she managed 2 or 3 weeks and she just went off for a month, think I managed until… middle of September, late September, and then I had 2 weeks between the end of September and the first week of October so I had to weeks off. Um… and then I had like, yeah, 'stress at work' on my note. So I tried to make it very obvious that that was what it was for, um, it was quite- it was not nice, but, yeah… I don't think- I couldn't have sustained- I just needed a break, because they were driving me a bit potty.

Interviewer: It sounds like a very tense environment, and you have the responsibility of your patients on top of it, so... And how has it been since, are you in the same practice still?

GPTr9: I'm still in the same practice, um… I think, um… because 2 of us have been off, they've had to change their ways slightly. Um… because obviously we've both reported it to the programme directors, so they have, um, changed it slightly in the fact that we no longer see the whole practice's face-to-face anymore, we can just see the majority- mostly our own face-to-face, which I think is fair. Um… there still are some other things they could do to improve, but I think that's sort of the main thing. Um… and, yeah, one of my other colleagues is still on a, like um, a fit note, so she's still got some of her hours kind of changed and things like that, um… but I didn't have that, so I'm managing, but I can kind of see the light now, and I'm like 'oh it's only til August, then I'll be gone!'

Interviewer: What's your plan for once you finish your ST3?

GPTr9: Um… to… hopefully have a few weeks off in August, um, 'cause of the kids and stuff, and childcare and school holidays, and then I'm going to hopefully get a job. So I've just been round looking at jobs, this week, um, yeah. Moving to **REDACTED area name**, so was in **REDACTED area name** and going to move across the border I think, to go to somewhere new.

Interviewer: Lovely, yeah! Would you say the pandemic has influenced your, um, your future career really? It sounds like you have learnt from it, in terms of what you like.

GPTr9: Um… I think it probably has influenced my future career, in that I'm much more open to the idea of moving now, whereas I wasn't before, previously I was quite fixed, like I'm definitely going to stay in one area, because I know that area and I'm comfortable with that area, and I thought- actually it's made me think that it would be best to be close to home for the kids, and actually that, you know, it can be flexible in the pandemic in the way that we do telephone consults, the way we do video consultations, you know I have been working from home on occasion, um, when we've had to split the practice up into bubbles, you know Bubble A and Bubble B come in on different days in case anyone had to isolate or anything like that. So I have had a laptop at home and on some days been working from home, so it's mad me think actually when I'm going in to do a job, that actually um, I could ask for that? I could say, actually, could I work from home one day a week? Which I would never have thought that a doctor could work from home, I was like how is that possible in doing our job. But, we can! And we've made it work, so actually, that's a positive.

Interviewer: Yeah completely, so you're doing- so you're doing one of your days, you're doing at home?

GPTr9: Some of the time, so we've got like 2 week rotas which change between, and then um, you know at the height of the pandemic we were definitely split into Group A and Group B, and the Group A and B didn't mix with each other, um, but now most of us had had our vaccine and the R number has gone right down, that we are kind of mixing back up again a bit, um, so that's changing, um, but that's how we were at the height of the pandemic.

Interviewer: That's an interesting concept, because I haven't- I mean you're probably my 7th or 8th person I've spoken to this week? And I haven't had that mentioned before, even though that sounds quite effective, so I'm really glad you said it because I'll ask about that more in future.

GPTr9: OK, that's good, you can write about that in your research!

Interviewer: Yeah definitely, so with remote working from home, that's coming with a bit of a shift to telemedicine, I'm guessing? How have you found the shift to telemedicine, and how have you balanced in person consultations? Obviously, a lot of in-person consultations for a bit!

GPTr9: Yeah I did a lot of everybody else's, which was horrible, because you don't know what's going on, you get half a story, so the patient's already spoken to somebody, and you're almost looking through the notes to think 'why have they brought him down? I wouldn't have brought them down if that was me'. You're almost looking for something to find, and you're like, I don't know what they were thinking! I don't know, I can't really, you know? They were- they were worried about this, and I'm looking at the patient thinking 'I don't know, I'm not worried' so you're almost second guessing, and it was really horrible, whereas if you've spoken to somebody on the phone, and you think, I'm worried about x, y, you bring them down and go it's not x, it's not y, whatever, this is what it is. It's a bit easier I think, because you've triaged it yourself?

Interviewer: So it was triaging by other doctors?

GPTr9: Yeah, so they were triaging into our spots, and then we were seeing their face-to-face and then our own as well, so we were seeing a lot more, whereas now, I just triage on the phone, or do a full consultation on the phone, and then if I think they need bringing in, then I'll see my own face-to-face, which is much better. Um, also in the practice, 'cause they were a little worried about the Covid situation, that we weren't allowed to see people in our own room. So we had designated green rooms, because the practice is quite big, it's a brand new building, so we had designated green rooms, and designated red rooms. The red room was for people that were possible Covid coming through the fire escape, straight in that room and straight out, and they had to wait in the car, but then green room was for like other people, and we all had to book a slot in the green room? So, you'd kind of be in your office somewhere, or maybe in a clinical room, but maybe upstairs, um… and uh, then you'd come down from your room upstairs, in like an office-type room, and then you'd book to see someone and we'd all kind of hot-desk in that room, so there was only ever 2 rooms that were having Covid patients in, so they would have a deep clean every day, but the other room wouldn't be cleaned? Other than the red room.

Interviewer: Yeah I've had um, similar things from other practices like the red zone, green zone thing. Did you find that was effective?

GPTr9: Um, I personally didn't like it, because I like to see people in my own room, where I know where all the equipment was, and I often find that going into the green rooms, I often have to make sure there was, like, thermometer covers… and, speculums… and that kind of thing, and often people were using them and then not stocking them up? Which if it's your room you'd think 'Oh I've used one of those' and go get one out of the cupboard or something like that. So I found that a bit frustrating, but I realised why we had to do it, but we've actually stopped doing that now, as of this week, we're gonna go back to just using our own clinical rooms, because we're just seeing an increase more in patients, and it's not practical, and people

were running late, and we were having more people in the waiting room, and we had concerns about Covid cross-contamination in the room, in the waiting room, so we're now just going back to using our own clinical rooms.

Interviewer: That's good I guess, because that's what you preferred? At the moment, how many of your appointments would you say are face to face?

GPTr9: Face to face… so in the morning I usually see probably 12… depends if I'm on an on-call day, if I'm on a non on-call day I see about 12 in the morning, telephones, and if I'm on an on-call in the morning, I could see like 18 or more, just depends, the list just kind of grows. But face to face out of those, I'd say that maybe, maybe 1 or 2, so, sort of, 10- 10 to 15 percent target really, for face to face.

Interviewer: Are you using texting? I know some people use a programme called AccuRx…

GPTr9: Yeah so we're using AccuRx, we use AccuRx to text sick notes, to, uh, text you know can you text me a picture of that rash, send it back, or, um, we can actually use a video consultation on AccuRx? So I've used that on occasion, for people saying like, 'oh my child's not very well'. And so rather than just bringing them straight down into the red room, if they're you know, a bit snotty or whatever, I just want to kind of check, you know, what they're up to. Sometimes a patient- a parent is not giving a kind of accurate summary over the telephone, they 'oh they're really poorly, they're not themselves, you know, they're really… I'm really worried about them'. And you do a video consultation and they're absolutely fine, running around the house, you know, throwing their toys everywhere, and you're like, actually we probably don't need to bring them down. Um, so I think, we were just being a bit cautious with how many we were bringing down to the red rooms. So, we were kind of being advised, you know obviously not in a baby, because you can't really assess a baby over video consultation, but certainly a pre-schooler or um, a school-aged child, you can, um, assess over video consultation.

Interviewer: How have you found that change to telemedicine in general then? How have you gotten used to these devices?

GPTr9: Um, it's been OK, it's been OK. I mean I've never used it before, um… I've been really surprised by how well the patients have taken to it. Um… they really like it! Really, really like it. Um, and like I say, I was shocked by that, and you know, I was thinking it's quite a deprived area, are people going to have access to the internet, are they going to have mobile phones, and things like that, but it's not really been a problem, and even with patients that are, you know, in their 70s and 80s, they shock me about how great they are with these devices! And I think, you know, I bet they'll just use their landline, but they- they want a telephone consultation, they want to use AccuRx and they a video consultation! Um, so that's been good, yeah.

Interviewer: Good, that's good to hear. Um, so how informed did you feel about the risk of Covid-19 to yourself and to your patients, and about how to look after your patients in the pandemic?

GPTr9: Um… informed. Probably not that well informed, I mean we had horror stories last year when we first started in the pandemic, we were panicking thinking that we were gonna have to go around, you know people would be dying in their phones, and that you know, we were gonna run out of all the end of life care drugs, because at the moment we issue, like, five vials of morphine, five vials of midazolam etc etc, for the people on end of life pathways, and we were thinking those are gonna have to be rationed, because you know, we can't just have five vials in everyone's house, we're gonna run out, from what they were saying… And then we were thinking we're gonna have to use these drugs that we're not so familiar, with like, alphentanyl and diamorphine, which aren't on our formulary, in case we can't get hold of them, and getting ourselves in like right circles with it, but actually, we didn't need to worry about any of that, but it wasn't really clear in general practice about what we were actually gonna be.

Interviewer: Did your role change?

GPTr9: Um… I think the role changed in that actually we were doing more telemedicine stuff, but not really the kind of palliative stuff, because that's just been the same really, um… that's not really changed greatly, and we've not been palliating people at home with Covid really.

Interviewer: OK. Have you um, have you found that you've been supported physically, so in terms of things like PPE, and also emotionally, so in terms of support as a clinician?

GPTr9: I think in terms of PPE, it's not been a problem. Um… I'm surprised I've not yet caught Covid, the amount of people that I've seen with it, I can't believe it. I've had my antibodies tested and they're negative, still can't believe it, but, um, but emotionally I think better at my previous practice, at this practice not so great, and I think they've not really, um, taken that into account really. I did, um, a friend of mine, that did the- that's also been off, she sent me some information about some Covid coaching that was available through NHS England, and I did sign up for that and I had 4 sessions, and that was really great, with a psychologist, and I thought that was really helpful to kind of, you know, help, sort of with the anxiety with going to work, um, and that kind of thing. But I don't know if I felt… yeah physically there's been a lot of help with PPE and, you know, segregating of zones and protocols and that kind of thing, but I think emotionally we could have done a bit better.

Interviewer: What would you advise maybe? I mean hindsight's a great thing, but…

GPTr9: I think yeah what we were doing at *REDACTED practice name 1* worked really well, just having a meeting together, with a few people, every day, just really makes you feel like people care? Just talking to each other really, and being there for each other.

Interviewer: Thank you, I think that's a really nice answer, and I think I ask them in the same question because I don't want it to seem like one's more important than the other, because I mainly, um, yeah the emotional support, working in a position like you have for a year. So yeah, thank you. So you had these sessions, do you think they're accessible normally for GPs?

GPTr9: I don't think they are normally, no I think they're a special NHS England thing that we got sent round on an email, to say, oh here's GP, NHS England support, and here's how you can access it, and it wasn't just for GPs, it was for anybody working in primary care, so GPs, nurses, receptionists, HCAs, anybody. Um, but I think if you were working in a hospital you'd have to access something else. Um, but it was done remotely, just like this on Zoom, we had 4 sessions, and yeah, it was good, it was helpful.

Interviewer: Good, I'm glad to hear it. How did you feel making decisions with the guidance that you did have?

GPTr9: Um… I think the hardest thing was that we were all worried about in the beginning was, how do you assess somebody's sats over the telephone, and there was this, um, I can't remember what it's called now, but someone sent some research around about some rubbish thing about, oh if they can hold their breath or something, or for so many seconds or something, um, it means that their sats are OK. All of this! And I remember reading it and thinking, we've got evidence that this is rubbish! Um… so I think that's what we were all mainly worried about, was how do you assess sats and breathlessness over the telephone, but I think now that we know a bit more about Covid that, you know, we just do a drive by in a car park and stick a sats probe on, or bring them down into the red room, or whatever, as long as you kind of can do that, and can satisfy yourself with 'oh probably they don't need admission currently', now we've got some sats probes that we can lend out to patients, but initially at the beginning of the pandemic we didn't have that availability. Um, the PCN did provide for our care homes and our learning disability homes a thermometer and a sats probe, but only for those, and not for general practice.

Interviewer: OK, so you have care homes associated with your practice?

GPTr9: Yeah, so in our primary care network, all our care homes that would be in that area, have been all like, rather than like each patient registered with whichever doctors, practice that they want to, and then all the doctors, like, you know are going to the same care home at lunch times, which seems totally bonkers, um, the care homes are all split up, and they were aligned to each GP practice? So we only had, like 4 aligned to our practice, one of which was a learning disability care home, one of which is a long-term mental health rehabilitation locked centre, so only 2 care of elderly, but those four, because they count as care homes, they all got given a sats probe and a thermometer with the PCN. So everybody, all the practices that were part of than PCN, aligned to their practice, they all got a thermometer and a sats probe each. But I don't know where the money came from, it just came from the PCN. Wherever the money from the PCN goes, it came from there.

Interviewer: Another entity.

GPTr9: Yeah!

Interviewer: How much do you interact with the PCN, do they inform or is it mostly resource-orientated?

GPTr9: It's…. mostly from the CCG actually that we get a lot of stuff from, not from the PCN, um… yeah mainly through them. We don't normally hear much through them, only from random things just turn up, like sort of equipment and stuff like that, and also our first contact physio and pharmacists come from the PCN.

Interviewer: Alright, thank you. So, I was going to ask about how common practice has changed for you, but you've obviously outlined a few key ones like triaging and telemedicine? Um, have you had any interaction with NHS 111 or secondary care that's been different to before?

GPTr9: Um… let me think. I've had a few, um, letters from secondary care which I've had to bounce back, which has been annoying, a gynaecologist asking me to prescribe some GnRH analogues, and uh, I had to write a letter back to say no, I can't, that's your remit, you need to prescribe that sorry, it's not on our formulary. A few things like, so… I had a patient that had bronchiectasis, um, that had been on the phone to a respiratory consultant and the respiratory consultant then a letter to us to say oh can you prescribe this ciploxacin antibiotic and it's a little bit like well… you 've spoken to the patient… so shouldn't you do the prescription? (*Laughs*).

Interviewer: Why wouldn't they do it themselves?

GPTr9: Oh because it's just easier to get a GP to do, isn't it? (*Laughs*).

Interviewer: That's frustrating.

GPTr9: Um, I did do it that time, because I felt like the respiratory doctors are so busy at the moment with the pandemic, I felt like I couldn't write a letter back to that, but I thought you know, if he keeps doing it I'll make a note of it and then I'll tell him (*laughs*).

Interviewer: Right, if he comes back again with it! Right, so you've described- have you had any new roles from secondary care, or are your patients still being referred in the same manner?

GPTr9: Um… ours are still being referred in the same manner, and they're being put on a waiting list if, um, their operations aren't going to go ahead, so we've not been told 'no don't refer', we've just been told 'your patients may go into a very, very big queue' and the patients have got to be made aware of that. And so, if I've been referring them about anything, I've just said you'll get a letter saying you've been accepted for an appointment, but you won't physically get an appointment because there are no appointments to give.

Interviewer: How are patients managing that, the longer waiting times?

GPTr9: Um, some are managing with it OK, and some are really grateful, some are every grump and very angry, you know 'why is my knee operation not being done' that sort of thing, and they don't understand- 'the theatre should be open now, and ITU is empty!' and all of this, and you've got to explain well actually, some of the nurses in the theatre might be isolating, or some people might be shielding, and you know it's not just a case of the numbers in ITU have gone down, there's other staffing implications. Trying to get them to understand that.

Interviewer: OK alright, um, it sounds like you have the right response ready now at this point, I guess after so long having to explain waiting times! In terms of the government response to Covid-19, how effective do you think they've been in controlling the pandemic, and also, in informing the public? Like, in terms of public health messages and policies?

GPTr9: Hmm…. I think they've done the best they could in a difficult situation, to be honest with you. I think the only thing that I would say about the public's kind of response is the public kind of thinking that GP surgeries are closed and haven't been working in the pandemic- the doors may be locked at certain times, but it doesn't mean that it's totally closed and the people

inside aren't doing any work. So I think that kind of public health message hasn't been great, but I think that overall, I mean I don't know if I could have done a better job, it's been really hard, and I think they've tried their best in very, very difficult situations. Um, I appreciate that perhaps we should have locked down earlier than we maybe should've done, that they were perhaps thinking about the long-term economy, and you know… it's very difficult very difficult situations, so I think perhaps they've done it 100%, but I don't think that I could've done it either, so, um, yeah I think they've tried their best.

Interviewer: Yeah, that's a fair response I think. It's quite a contentious question, but yeah. Um, I think it's interesting you say about the public not knowing about GPs being open, because I've heard this a few times now, would you think there's- if things were to be different, would it better if the public were more informed of how they can use their GPs?

GPTr9: Yeah! I think that would've been a good idea, because I've had quite a few people on telephone triage, when I've rang them up, they've said 'oh I rang 111 and they told me just to ring you' and I thought well why did you not just ring us to start with? You know that you're registered with us, just ring us! Like, why bother waiting for 111? It's 2 o'clock in the afternoon, of course just ring us! So yeah I've had- I've been a bit confused by that, and I think some patients, especially some round in our area that maybe that have got lower IQs and lower socioeconomic status, etc etc, maybe wouldn't have realised that, 'cause they might've just seen the door closed, and thought, oh, that means they're closed then. They wouldn't have thought, oh the lights are all on, I can see people inside the building, oh maybe I can still get an appointment, um it's just an automatic 'oh! They're closed!'.

Interviewer: And maybe, people are probably worried about burdening?

GPTr9: Yeah, I have to say though, during the first pandemic, the stuff that was coming through on the telephone was like really decent queries, like I've got this problem, I've got that problem, I've got a UTI, like I think, this is a GP, easy door problem. But now we're getting more people ringing about 'I've had this problem for 6 years doctor' and you're like, that's lovely, we are in the middle of a pandemic but we'll try our best! So I think people are coming out more of the woodwork, things that they've had for a long time that they're trying to get sorted.

Interviewer: Would you say that the general presentation of patients has changed much?

GPTr9: Since March, April of last year, I felt that the stuff that was coming out… not saying that people ringing now, there stuff isn't genuine, but people were ringing then with the real bread and butter of general practice, you know, of like, you know, really proper medical kind of things that you can really put in a nice simple box, and you can, you know, sort out. Whereas we're

getting again now the really difficult kind of family situations, social situations, you know people ringing the GP for that. You know, lots of questions at the moment about, you know, should I be shielding, should I not be shielding, employer related questions… All sort of different things which people weren't ringing about before, um, you know, 'I'm having a bad time at work' and asking you to get involved with things that are probably more for the government advice bureau and things like that, and just trying to direct them to that kind of place.

Interviewer: OK. Has your management of chronic care patients had to change at all?

GPTr9: Um, yes, I think at the practice I'm at now we haven't done people's uh chronic reviews, so there's a short cohort of people around 3 or 4 months last year that were missed, and then we're still catching up with smears as well, because there was a block of time that those were off, um, so- and we've still got a bit of a backlog. So I guess chronic care has… suffered really.

Interviewer: OK. So I mean you've told me a few anyway, but I was going to ask- are there any changes which you think should and should not be carried into the future? So… telemedicine or otherwise, is there anything you would say is beneficial and should stay, and will shape GP for the better?

GPTr9: I- I think that we will never go back. We will never go back to having a waiting room full of 15, 20 people- and you know it used to be so stressful when we were running late, because you might get somebody saying they were depressed, and they were gonna kill themselves, and you genuinely- it doesn't matter how amazing and great you are, I cannot do that in 10 minutes, you've got to show empathy, you've got to show kindness, and you've really, you know, you've got to get them on board, and you can invariably run late into the next appointment, and then the next appointment, and then you'd have 3 or 4 people on your screen who were waiting, and it's just the most stressful thing. And so, we'll never have that now, because we'll never have people able to just make an appointment flat-off, and I think we'll always have telephones interspersed with face to face, so I don't think we'll ever have a full waiting room like that again.

Interviewer: OK, well that makes sense!

GPTr9: Yeah it does! It's time utilisation. You can spend 2 minutes with somebody on the phone and then 10 minutes with somebody on the phone, and you'd be waiting sometimes in your room, you'd see loads of people back to back, then you'd have a few DNAs and you'd just be sat there, waiting in your room, whereas now, you can just ring people, so it's fine!

Interviewer: I guess, does it give you a bit more control over your work as well?

GPTr9: Definitely more control, and it's much more convenient for the clinician as well definitely. Yeah and some people- I find with my depressions that the first or second appointments, they do need a bit longer, but after that if you're just catching up with them, it can be shorter. So…

Interviewer: How is it managing patients, for example presenting as depressed, over the phone in comparison to in person?

GPTr9: Um, I think that we'll never go back to seeing many people like that, face to face, because I think you can gain quite a lot from that still over the telephone, because it's all mainly in the history, and it's all mainly in what they're saying to you, so I think seeing them face to face doesn't add a massive amount to that, um, I think if you put the time in to listen properly on the telephone, and to be attentive to their kind of cues, then, I think that that, you know, that's ok I think. You know those are kind of the best ones to do on the phone.

Interviewer: Really?

GPTr9: Yeah. They feel more natural on the phone, you know because you're just chatting to somebody about how they feel, and you know that sort of thing as opposed to like their shoulder, like 'oh can you move it, can you move it to the top, can you do this with it, can you do that?' whereas talking to someone about how they feel, or what's happening or who's around them, I think… And I supposed the red flags you have to really directly ask them, whereas sometimes if you were seeing someone in practice the red flags could kind of be inferred, because you could see them in front of your face, but you definitely have to check that, and do really good safety netting at the end, just to make sure that, you know, to protect yourself and patient really.

Interviewer: Is that something you've, um, had, any sort of advice on, or is that something you've had to teach yourself as you've gone through?

GPTr9: I think because I did my exam where I did the second part of the MRCGP where they did the CSA and then did the recorded clinical segment, so then we were doing all of that as part of recording for our exam, so yeah I think it was just part of that really.

Interviewer: That's good I guess! Um, so, my final question for you is what do you think we can learn from the pandemic?

GPTr9: I think things that we could learn would be.. well… all the sort of telemedicine stuff which everyone's really happy about going forward, um, I think other things that we could learn about is, um, actually spending time, just to kind of check up on each other, just to make sure everyone's OK, and having that time each day to have a short coffee break all together. Um, and also the other thing that we've learnt is the amount of home visits that we have has gone down, which has got to be a good thing, so I'm happy for that! (*Laughs*).

Interviewer: I'm glad to hear it!

Recording ends.
