# Supplementary material for: Primary health care during the COVID-19 pandemic: A qualitative exploration of the challenges and changes in practice experienced by GPs and GP trainees
Source: PLoS One. 2023 Feb 9;18(2):e0280733. doi: 10.1371/journal.pone.0280733 (PMC9910752; doi:10.1371/journal.pone.0280733)
Supplement: S1 Data — (ZIP) [file pone.0280733.s005.zip › GPTr10 Transcript.pdf]

GPTr10 Transcript

Interviewer: Right okay so to start, can you tell me a little bit about your experience in general practice before the pandemic.

GPTr10: Uhh yep, so I did four months of general practices as an F2 probably about four years ago, or something, um... which was kind of my first real flavour of general practice, which was obviously well before the pandemic, um, and... you know it was up in the **REDACTED city name**, um... so probably quite different to my experiences that I've had in **REDACTED city name**, it was very much, you know, the usual, patients coming in sitting in the waiting room all that kind of stuff. I started my ST2 GP placement in February of last year, so just before the pandemic started and had probably about a month to six weeks of more traditional general practice, while I was sort of doing my induction and just getting into things, so patients coming in as normal, kind of having that kind of experience that we associate with going to see the doctor. Um... which was kind of all fine and all kind of what I was expecting it to be... didn't really do anything over the telephone, um, more or less everything was kind of done face-to-face. Which was fine.

Interviewer: Cool, have you been in any practices since, or is that the current practice you're in?

GPTr10: So that was a different practice to where I am now, so I did six months between February and August in a different practice that's actually really close by, and now I've been at this place for about seven months now.

Interviewer: Can you tell me about your practice demographic for the one you're in now?

GPTr10: Yeah so it's a really mixed demographic in **REDACTED area name** in **REDACTED area name** so it's, um... fairly diverse in terms of background and ethnicity. We've got um, kind of housing estates and lots of blocks of flats above our building, so there's quite a lot of deprived patients, but we also have quite a lot of uh... middle class, um, older generation patients, um, it- on our books in our practice, so we have quite a lot of private referrals and patients with varying expectations, so very, very varied in terms of backgrounds and kind of social caste and economic stuff I guess?

Interviewer: Alright, thank you. So could you tell me about your experience of the Covid-19 pandemic professionally?

GPTr10: Um yeah so... um... was initially just very... sort of, uh... there was a lot of uncertainty at work, about, um, what we could or couldn't do, what we should or shouldn't be doing, about bringing patients in, not bringing patients in, and I think probably around the kind of March, April time in the first wave, there was a very sort of scary really couple of weeks, where we were trying to triage patients over the phone for breathlessness and there was this big, you know, 12 hour wait for an

ambulance and just really like not knowing what we should be doing with patients with Covid- with Covid problems, um, and it being very, very difficult to keep up with what was going on in the different hospitals. We sit between three different local hospitals, and so- that's probably a continued theme, it's quite difficult just to keep up with everything, as- as a trainee, you know, you're trying to learn how to be a GP and also keep on top of learning new medical things and stuff and having Covid on top of that was quite, um... overwhelming I guess at times. So definitely initially it was a lot of, um... uncertainty about how to approach Covid itself and about how to approach the rest of medicine basically on the phone? So, most of my ST2 was done pretty much all on the phone, um, and- and that's kind of continued and now started to ease off a little bit and we're finding the balance between seeing some patients and doing other bits over the phone, um, so yeah.

Interviewer: Okay, you spoke about being overwhelmed from the sort of changes, did you have much guidance for those?

GPTr10: Um, so... probably not sort of national guidance about how we should all be approaching things, when I was in the first wave, I was working in a really big practice which has got like 20, 25, GPs? So I felt like I had a lot of support as a trainee and as a member of the team, we were all very much in it together, we had like a briefing twice a day, um, there was really good sort of camaraderie and we did lots of sort of wellbeing stuff and, um, looked out for each other, so I think there was a good- a good feeling of you can ask someone if you're not sure and it's OK to not be sure, because this is new territory. But I think, certainly initially there wasn't fantastic- and I still don't think there is- guidance about how to be safely triaging patients over the phone, I think, probably because there isn't really a way to do it. Um... and that I think was quite difficult at the start.

Interviewer: Yeah I can imagine! Um, when you say there isn't really a way to do it, what do you mean by that?

GPTr10: So I think in the- in the first wave there was this big thing about using the ROTH score, um... and sort of counting... getting patients to count and how far can they count, and does that mean they're too breathless, or not breathless enough. And then, after about two weeks there was this big thing about the ROTH score's not safe, you shouldn't be using it, and everyone thinking oh my God, I spoke to so many patients last week, and thought they were fine because I was doing what I was told to do, um... so that was quite difficult, quite stressful. And I think we all kind of realised that the reality is that every patient is different, and you know there's going to be lots of confounding factors on the phone, you know language barrier, age, expectation, etc. Um... so I think that has played into, you know, where some patients are getting better care than others maybe, but yeah.

Interviewer: Yeah completely, that's very sensible sounding definitely. Could you tell me about the support you had in terms of, um, PPE and sort of public health measures?

GPTr10: Umm, yeah, so, PPE I think has been fairly basic from general practice but probably... adequate so we... if we were seeing patients, we would have access to, uh, apron, gloves, mask and a visor if we wanted to use a visor? And we would be encouraged to do that and are still using those things, you know, now if we feel that we need to, um... and I think we- we had a reasonable amount of guidance as to what we should be doing um, at the time I suppose. We also have lots of things like you know, do you feel that you're high-risk, filling in your how- how- you know, your own personal details for, like, risk, and if you felt uncomfortable doing stuff, then you didn't- wouldn't have to do stuff etc, um so I think in terms of that I feel like iIve been looked after. Um, so I think that's kind of all been okay.

Interviewer: Okay, that's good. Did that come from your practice, the um, evaluation things?

GPTr10: Um... I have done one- at least one of the practices I've worked at, if not both, and I also have been sent one by my kind of employer, so the local trust that actually pays me.

Interviewer: Right, OK. Sure, cool thank you. How did you feel making decisions for patients with the guidance that you were being given?

GPTr10: Um, so I think sometimes definitely uncomfortable with, um, managing Cov- definitely initially Covid patients over the phone, um... because I don't think we really had any strong guidance about how to do that. As soon as we had access to things like Covid hubs or, there was- the ambulance service wasn't completely overwhelmed, then things start- have started to feel more comfortable and now we have a much better system in place for- for being able to examine patients and for being able to get pulse oximeters to patients and, you know, having a low threshold to just send them in if we need to um, I think, also from having to do telephone... like- triage, or telephone appointments for patients, especially as a fairly junior trainee at the time, um, sometimes that was really difficult and it's very difficult to make a decision about whether a patient needs to come in to see you or the patient needs to go to hospital or whether a patient is just being a bit dramatic and actually just is fine. And I think even the more senior doctors found that difficult, because they weren't used to doing everything over the phone everyone's used to happening at face-to-face interaction, where you can just see someone's fine immediately or, hang on you're not fine and so that was difficult and stressful, and I don't think I don't think there is guidance to be able to do that it's more, you know, you kind of need to learn... learn how to do that, pick it up over time, which is difficult mid-pandemic I guess.

Interviewer: Yeah that sounds very difficult to just pick up, um, how are you managing now a year into using telemedicine?

GPTr10: Um, so I feel like okay. To be fair I'm quite like a chatty person and I quite like the phone, so I think some people found it more difficult because they- they need that face-to-face interaction sort of thing, whereas I quite like talking to people on the phone um, and... you know, I quite like having someone on loudspeaker or having a headset on and just be able to sort of almost relax a little bit. From a training point of view it's also quite useful to be able to look stuff up on the computer, you can do notes, at the same time, you can be quite time efficient, um... and I think I've got a better grasp of which patients need to come in to see me, be it because they're medically unwell or be it because they're very anxious or be it because I can just tell I'm not going to get a good end result if I don't do that, and I think now we're in a much better position to be able to do it more safely because numbers are under control, and at this practice patients are being vaccinated which are a lot of the patients that we would want to see anyway, um... so I think we've, like, gotten used to having to negotiate those issues, um, so I don't feel uncomfortable as such, with that side of things, in general. In general okay.

Interviewer: Ok great thank you. Um... so in terms of general practice you've spoke about telemedicine, has your role as a GP changed in any other way?

GPTr10: So I suppose um... some of the things that have been... different- I think we've had to take a lot of burden away from secondary care where secondary care has just been completely shut down, um, so we've been in... I suppose the term that we sometimes use in general practices is ‘holding’, we've been holding lots of patients both their physical health that sometimes looked after, at least in part, by secondary care, and for mental health, um which is kind of our job anyway, but obviously there's been an enormous influx of, um... patients struggling. And so I think there's been quite a lot of that burden transferred onto general practice obviously we've had to absorb doing the vaccination stuff, which is something that we're just finding people in time to, kind of, to be able to do, um, so, you know, those are kind of some of the things that have been a bit more difficult, um, I suppose just trying to generally be a bit more practical around, um, you know around doing certain bits on the phone, and then saying why don't you drop this test in here and then we'll have a catch up then, and having to be a bit more tactical about the way we approach, um, consulting and about, um, with patients and things. Um, (*unintelligible*) saying someone's- ok they're not- they're not comfortable to come in, or they're not comfortable to have treatment until they've had two doses of this or whatever so having to have that extra element of, kind of, compromise to... to their care really.

Interviewer: That's interesting, um yeah, the mitigating of patient nerves, yeah. With the responsibilities from secondary care, when you said you are holding patients what does that actually involve, how would that change your care of the patient?

GPTr10: Um, so I think lots of patients, um... just full stop not being seen by their specialist? So I mean counting- we've got lots of patients on very complex medications, you know rheumatology patients on biologic treatment, um... lots of patients on

biologic treatment, things like that, that we're then having to sort of do their blood tests and be able to maybe interpret those blood tests, even though they're not things that we would usually do, um, and also, I think, just picking up just like the actual care aspects those patients would expect to see their cardiologist or their respiratory doctor every three to six months, and then they haven't seen them for a year and a half, so, you know, maybe normally they have an ECHO every six months, but they can't have that and then they start to get symptoms and there's no way for them to have an ECHO, and they can't get hold of their cardiologist and the sort of complexity of patients care is massively rebounding on us in general practice, where we're then having to make decisions and... out of things that might be further out of our comfort zone. And I think the logistics of getting things done, like doing the routine monitoring and things like that has been- has been really stressful, has been really difficult to get access to X-rays and ECGs and those sorts of, like, basic tests that normally are very, very easy for us to access.

Interviewer: Okay. That's a lot of extra work as a trainee as well! Um, yeah I can imagine it being a very busy time. Are referrals starting to ease now- the waiting times?

GPTr10: Um... I mean I think probably not to be honest with you, I don't know but in the news today the waiting times are at an all-time high um... so I think some patients it's very- very dependent on what they've been referred for- something, you know- I think patients understand that they're in the big queue if they're waiting for a hip replacement, and they've tried the conservative things they know that what they need is a hip replacement and they know that we as general practice have absolutely no sway over that. It's not really urgent-urgent, but it is going to impact on their quality of life. I think, where it's been more difficult is those patients that are quite reliant on their specialist for various other things, not being able to get access to them, um, and a lot of it kind of just falling back onto us. And yeah as a trainee when you don't have that- the wealth of experience and things that has been quite- that has been difficult and stressful.

Interviewer: I understand. You spoke about the vaccination program, could you tell me about that in your practice, have you been involved in that at all?

GPTr10: Yeah, so we have... we- I'm in a practice, um... there's like three practices that come together to form a sort of- a group really, and our group plus probably another couple of groups have... are part of like the vaccination program within **REDACTED city name**. And so, there's a local centre that's down by the train station that used to be used as, um, it was like a wheelchair centre, um, for patients with mobility needs, and it was turned into a Covid hub during the first wave to assess patients, so it's got a big- it's quite a big space, um, but it's now been turned into the vaccination hub, so we have... got a care coordinator at our practice who's a fairly new member of staff who's been working really hard to get our vaccination- our- patients vaccinated, um, and is involved in booking and speaking to patients about their concerns. We have obviously spoken, you know, spoke to

lots of patients about their own concerns about vaccination and grouping, and all that sort of stuff, um... and then the vaccinations themselves have been fairly continuous I think we're doing quite well in our area? And our practice usually contributes a couple of members of staff like once a week on a Thursday. So I've been down once and probably gonna go down again in the next couple of weeks um... and that's actually really nice because it's a bit different, everyone's very happy to be, um, vaccinated it's, you know, people are very thankful and there's generally not as much complexity in terms of, you know, it's fairly straightforward who can- can and can't have a vaccination and there's someone there to ask if there's someone complicated so it's actually quite easy, um, which is quite nice. Yeah so that's been really good and something quite, you know, it's obviously been very positive in a- in a difficult time.

Interviewer: That's really nice to hear. It's quite exciting to be part of something like this, that will probably be a historic thing.

GPTr10: Hmm yeah, definitely, definitely!

Interviewer: Yeah. You spoke a lot about peer support in your last practice, I was wondering if Covid has changed your relationship with your patients or your colleagues in your current practice?

GPTr10: Um, I think, as- I think definitely we... especially in my previous practice, it did make everybody gel together because we initially were then kind of all consulting in one big room all together, it's a bit like a call centre, there was a lot more asking people about things, and looking out for others and making sure everyone was okay. Um, so I think probably as a whole it has brought people together quite well and I think this is probably similarly in the practice I'm at now, although it's much smaller. Um, I think with patients, I think, it- as I was saying before about kind of holding patients that have got maybe mental health difficulties or struggling with anxiety and lots of that is often around, um, Covid, I think we are in a strong position from general practice to be able to reassure, and to be able to, you know, help patients and some patients have really... appreciated and hopefully benefited from that. Um, I suppose we've missed out on that face-to-face, you know, relationship building sort of stuff with patients and as a patient for example today that I've been, um... trying to help out with his sort of social situation, it's not actually very medical at all, but um, he's been sort of kicked out of his house with his daughter and it's unfortunate, um but he's also got quite badly controlled diabetes and various other things and I spoke to him on the phone about three or four times, he's often crying, and he came in to see me today as he wants to do something for his diabetes, and it was just really interesting, where it was like, oh, I've, you know, finally putting a face to a name kind of thing because I've never actually met him. Um... and it's interesting how it just feels that that's not the norm anymore, when obviously previously it used to be.

Interviewer: Yeah.

GPTr10: So I think yeah... it's kind of a bit swings and round abouts, some things maybe we've all grown closer with and some patients have maybe benefited where they can't get down to the practice normally anyway so over the phone has been fine, but some people have probably missed out, um... and it's been more difficult to establish a good, you know, kind of working relationship with them.

Interviewer: I guess accessibility depends on the patient, yeah. Um, it must be nice when you finally get to meet them.

GPTr10: Yeah definitely, totally, it's- it is really, really nice.

Interviewer: Yeah, yeah. So… quite a big question, but what is your opinion of the government response to Covid-19 in terms of um, public health measures and policies.

GPTr10: Ooh, um, I mean I think it has been really, really difficult um... you know, it's completely uncharted territory, and I think- I don't envy anyone having to make decisions about what to do and what people can do.

Interviewer: Yeah.

GPTr10: I suppose, um... Looking now with hindsight it does feel that maybe we've been a little bit too soft and- and not- not gone early enough with some of the lockdown stuff, um and I do- you know as someone that's been on like the tube and on public transport, like throughout the whole thing, it is sometimes quite frustrating to see how many people very blatantly flaunting the rules and not wearing masks and are fairly obviously not going to work, not doing the things that they should be doing, and then you do wonder whether a little bit more strength in that area may have, uh... reduced the burden of the pandemic on people and you know, maybe saved lives, I don't know. Um I think you know overall there's been fairly good communication about stuff that's been briefings, there's been, you know, let's try and keep people aware of what's going on, which has been good um... And you know, there has been, you know clapping for NHS and that kind of stuff, which was probably- you know, which was nice at the time, and it was nice that there was some recognition for, you know, frontline workers, which has perhaps, you know, faded away a little bit now, which is not surprising, but um, yeah I dunno, I suppose I've probably been almost too busy to have like a real... sort of much time to really reflect on it.

Interviewer: Yeah, fair enough.

GPTr10: And then there's so much to it isn't there, there's lots of different areas that have been affected both professionally and personally I guess.

Interviewer: Yeah it's a huge question to ask, because when you really start unpacking it, you could just keep going if you were to look at all angles, but no I appreciate that answer, thank you. I live in London too and in summer I was thinking the same thing on the tube! So a more sensitive question, but personally has there been any impact of Covid, has it had any effect on your life?

GPTr10: So I haven't actually had Covid itself, and my family and friends and things have stayed well, touch wood, um so from a, sort of, physical point of view it hasn't massively impacted on me, but it has been, like it has been exhausting, um, and I think as a trainee what people maybe forget is that we still have to do exams, I've just been doing some absolute nonsense recording exam thing, um, for which there hasn't been very good support, not very good information etc about, and it's been a massive stress for basically anyone that's a trainee at the moment, and I think people forget that as trainees we're dealing with quite a lot anyway, and that we do a lot of clinical sessions compared to the vast majority of GPs. And we have to do portfolio work and we have to do exams and the exams got pushed back because of Covid, and so everyone's trying to squeeze doing lots into a very small amount of time? And no one's been able to go on holiday or see their friends and family very much, and all of that kind of, then results in basically burnout, and I feel at the moment that I've- I've hit that level. Fortunately, I've got a week off next week which I'm very much looking forward to, and will hopefully feel a bit more energetic, but um, it has really highlighted that need to look after yourself, um, and you know, I think people have been really good at thinking about wellbeing and whatnot, but I think it has been really difficult as a trainee you have more pressures on you, you're less, you know, you're less mature as, uh, knowing what- what you need to do to look after yourself, um, and so some of that has been quite difficult and I definitely have felt burned out on numerous occasions, and like you know I really need a break kind of thing.

Interviewer: Yeah, no I understand, but I'm really sorry to hear that. Thank you being so candid, um... how are you doing then?

GPTr10: Yeah, I mean fine, I've just been- it's all kind of like come to a bit of a point this week because I've had to do this hand-in for the exam stuff, um... and... I'm usually a very, like, chipper person at work, and you know, generally quite a nice person to be around, but I just felt like it's made me be a bit miserable and you know, part of that's just life it's not fun doing exams and whatnot, but I think there's a general consensus feeling amongst most people in my position that we haven't been given very good information or support around things and, unfortunately, such is medicine that t's all sort of very much comes from the hierarchy, and you know... there's- what might have been nice was for someone to say you guys have had a really difficult time, how can we make this easier for you, how could we make this, you know, um, less stressful so that you feel that you can get the

best out of your training and I don't think that's happened at all. Um... and that then unfortunately just has a massive impact on your, kind of, quality of life and sometimes also on your outlook of medicine as a career, um... which is a bit of a shame, but I think it also then all just, you know, it's a bit of a vicious circle, you then get a bit stressed, and a bit tired, and don't sleep very well and then, you get a bit angry at life and a bit angry at medicine and whatever, and all you really need is just to chill out for a week, and probably not do very much because I can't do very much but um, but yeah I think that's probably been the biggest challenge, certainly this, like- this year, having been in lockdown.

Interviewer: Yeah it's been such a long time now, I think it's inevitable that you're going to have some kind of burnout at some point but I appreciate you talking to me today when you're in your final push before the week off, but I hope that week off-

GPTr10: That's fine, it's only one more day now!

Both laugh.

Interviewer: I hope the week off is amazing, just loads of lie-ins and doing nothing.

GPTr10: Yes, definitely.

Interviewer: Yeah, on that note, I want to ask how do you think the pandemic has affected your training? You spoke about the recorded exams which I've had other trainees also say they're also struggling with, has it affected do you think your GP training or your outlook on GP?

GPTr10: Um yeah, I mean I think it definitely has, I think we're in a slightly unique position where I haven't done, or at least not recently I haven't done very much of GP- what GP was like before so I've got very used to telephone consulting and I feel relatively comfortable with that now, um... And I think hopefully going forward, we will be able to pave the way for doing some telephone consulting and some face to face and a nice mix, um, of those two things. I think in terms of my training, yet it definitely has made thing... um... more difficult, and I think I've probably had less exposure to certain things that would have been useful to me, even, you know- just like really basic things like looking at skin lesions or rashes and things like that, we haven't really had patients coming in about those sorts of problems, and maybe some things around timekeeping, having patients waiting in the waiting room, being able to deal with that, which is a big part of general practice. Whether that will be a big part of general practice going forward is difficult to know. Um, and... and yeah I suppose you know, having exams pushed back because of Covid and then trying to squeeze a lot into one period of time and that stuff, um, has probably just made me

feel like I'm not enjoying my training as much, definitely over the last few months, I don't think this recorded thing is an especially good way to, uh, you know, to check that you're good enough to be a GP but I appreciate that it's difficult for them, and they have to do something. Um, but as I suggested before I do think there could be a little bit more thought to, you know, the circumstances of us as trainees that are tired and have been slogging away really... But equally there's some positives, you know, there's things like, um, being able to look at e-consults and look at photos and things with other members of staff beforehand, which have been good learning opportunities, um... and like I say being able to look stuff up as you go along is really good, um, which- which I've really, kind of, appreciated so, um... that's- that's probably been useful, I suppose overall you just kind of take what you get, you don't really have a choice, just try and make the best of it and go from there.

Interviewer: Yeah, I guess that is true. Um hopefully... I've had- you're the 10th- I think you're actually the last person I have an interview with! But I really hope this research goes somewhere, because your experiences reflect a lot of what I've heard already, plus new things, but it would be nice to put that together and have somebody actually see, um, what it's been like for GP trainees. It's especially interesting that all of your exposure has been pandemic exposure? Like your learning for consulting is quite interesting. But yeah, thank you for your answer. Um, sort of touched on it already, but I normally like to ask about what changes you think should be carried on into the future of general practice? And equally, what changes you would like to not see carried on into the future?

GPTr10: Hmm so- so I think there definitely is a place for telephone consulting, um... and I like doing some stuff over the phone, I think a lot of things can be achieved over the phone. It can be way more convenient for lots of patients, and we should try and take that into account, it also takes the pressure off us people, waiting in the waiting room, so I think trying to keep some things on the phone? But finding a way to make that convenient for us as doctors and for patients as well.

Interviewer: Yeah.

GPTr10: That would be good... I think... I'm trying to think what would not be good, what do I not want to continue, um... There tends to be a lot of duplication with, if you speak to them on the phone and then you say 'Okay, you need to now come in and I need to examine you', and then you end up kind of going through the whole thing again. If there were a way to make things a little bit more streamlined?

Interviewer: Yeah, so sort of the triaging process?

GPTr10: Yeah I think that's really, really difficult to do, because I think some practices have left that in the hands of the doctors, and some have tried to train up admin and reception staff to do things, there's invariably going to be people that fall through the cracks or it doesn't quite work out for. Um, but it can be quite frustrating at the moment when patients are sort of almost demanding a face-to-face appointment when it's not necessarily appropriate, and I think sometimes, you know, you kind of want to have control over that at least at the moment, I don't know um... but yeah I think definitely trying to keep things like e-consult and um, the telephone triage stuff is good, e-consult is definitely really good for learning for trainees and for students and things, um... But hopefully being able to reint- sort of reintegrate the face-to-face stuff, maybe with people- less time of people hanging around in the waiting room, I don't know, finding a way to split that up? I don't know, I wouldn't want to be the one that's trying to do that, but...

Both laugh.

Interviewer: No they're good ideas and suggestions to go towards that so thank you, um and yeah massive job to reorganise but it's going to have to happen I guess. Um, what do you think the main challenge is going to be to GP care going forwards, sort of coming out of the pandemic?

GPTr10: Um, so I think just, obviously there's going to be this huge backlog of secondary care stuff and of, you know, this worry about missed diagnosis and missed cancer diagnosis- diagnoses, and all these sorts of things, I think there'll probably be some backlash from that, um... and- and there's going to be lots of things that we'll just have to physically catch up on, lots of long term care for patients with diabetes, high blood pressure, blah blah blah, um, has not been done very well through the pandemic 'cause it's very difficult, um... so I think there'll be that big, kind of, catch-up process. I suspect there'll be like an ongoing kind of burden with mental health because there's going to be big unemployment, patients that will have unfortunately lost relatives and loved ones and things, um, I think it's likely to have complicated, uh, life for a lot of people, and I think probably mental health will continue to be a big area and a big challenge yeah, um... and I think also just about us as GPs wanting to make sure that we still enjoy the job, um... and being able to find the balance and I know when I- at the very first part of the pandemic some of the more senior GPs already were like this isn't what I signed up for, this isn't- this isn't what I want to be doing. So I suppose having to find the balance so that you are doing something that you find, you know, you enjoy, and fulfilling etc, so I think there's some challenge to be found in that, um, and at the moment, having you know, been slogging through ST3, it just feels like you want to do as few sessions as possible, which is obviously not what you want to be feeling, but sometimes it just feels like you know that's... that's what you want, you just you just want to be able to relax a bit more.

Interviewer: Well you're human. I hope that the week helps and then things do start to give you time to enjoy life outside of GP a bit more, but um yeah, thank you. I um, I... feel like we covered quite a bit of GP care today. Is there anything that we haven't spoken about that is important to you, about your experience from the pandemic?

GPTr10: Um, not- not specifically I don't think, or probably not unique to me anyway, everyone's had their own kind of journey through stuff I guess anyway, um but no probably not specifically.

Interviewer: OK all right, no worries! So last question, what do you think we can learn from the pandemic?

GPTr10: So I suppose there's again there's loads of levels to that question isn't there, there's lots of things that you feel like we could learn as a society, about where our limits are, what we're good at doing what we're obviously not very good at doing um... and... you know, maybe also from a, uh... you know, government, or whoever's in charge point of view about willingness to act and to be decisive or be very indecisive, unfortunately. Um... I think medically and from general practice we probably learned that we can do a lot of things remotely and we can still provide a good level of care for the vast, vast majority of patients over the phone and by being a bit tactical with how we do investigations and things um... and that if anything, probably it just makes us better at our job by, you know, having to be that little bit more thorough over the phone, um, and kind of learning to be very good at telephone triage and good at using- using the resources around us.

Interviewer: Yeah.

GPTr10: But, also, that that comes with a bit of a price in terms of being tired and it being a bit scary and a bit exhausting. Hopefully we've all learned a little bit about ourselves, in terms of, um, you know where our own limits lie, how we can relax, how we look after ourselves better, look after our colleagues better, um and that kind of stuff, 'cause I think it has been really difficult for a lot of people, so an endless number of lessons to learn, no doubt. But hopefully come through a bit stronger.

Interviewer: I think a lot of those lessons, um, reflect on people from all walks of life going through the pandemic so that's a really nice answer, thank you. That's everything I've got to ask, I'll stop recording here.

Recording ends.
