# Supplementary material for: Primary health care during the COVID-19 pandemic: A qualitative exploration of the challenges and changes in practice experienced by GPs and GP trainees
Source: PLoS One. 2023 Feb 9;18(2):e0280733. doi: 10.1371/journal.pone.0280733 (PMC9910752; doi:10.1371/journal.pone.0280733)
Supplement: S1 Data — (ZIP) [file pone.0280733.s005.zip › GPTr7 Transcript.pdf]

GPTr7 Transcript

Interviewer: So to start off, could you tell me a little bit about your general experience in general practice, pre-pandemic, sort of where you've worked and what you've done?

GPTr7: So I think pre pandemic, um, as a GP trainee I hadn't really had any experience of GP, so I think that's- that's kind of the experience that I've had has been very strange because it's been only really in the pandemic, so I didn't previously do a GP job, um, my previous jobs as part of GP training were in OBS &GYNAE and palliative care and I've just finished a job in A&E in February (*laughs, makes prayer hands*).

Interviewer: Right, phew!

GPTr7: I'm quite happy to be getting out really. Yeah, good timing. And I started my GP jobs in February 2020. So, depends actually when you'd define the pandemic as starting.

Interviewer: Yeah tricky one, March, I guess? But- so have you been in multiple GP practices or just in one GP practice?

GPTr7: Yep, so I started in February 2020 at my ST2- that was my ST2- so I had six months in my old practice, **REDACTED practice name**, which is part of an MP, and then I moved to **REDACTED practice name** surgery in August.

Interviewer: Okay. What's the demographic like of your practice?

GPTr7: Um... they're a little bit different as- my current practices are quite varied, they're both quite- quite white practices, quite a lot of English as their first language, um... They're- my current practice is probably slightly more affluent than my first practice? Um, my first practice was kind of around Stirchley kind of area.

Interviewer: Um so... how prepared did you feel for the pandemic? Could you tell me about your professional experience of Covid-19?

GPTr7: I felt very underprepared and very- I found it very difficult as a GP trainee to know really what- what my role was? And what I was learning... and I think the longer it's done on the more I've kind of become concerned about the fact that all of my experience in GP is this pandemic, and I didn't really know what: normal general practice is, I've gotta say. So back in February, when it first started, I supposed I'd had about a month of kind of settling in, I'd had a couple of weeks of kind of shadowing and

settling in, and then moved onto kind of half hour, very long, appointments, and I was just getting into the swing of seeing patients and getting used to general practices- quite change of lifestyle, then it all became telephone and... My first practice we didn't see anyone in the practice face to face at all. Um, everyone was seen at a different practice, so it was one GP from my practice would go over there, which they didn't- they didn't really make me or ask me to do so, I just was on the phone for everyone. I found it really difficult to know how to- how to adjust to that really because I think GP is such a change in pace and- and responsibility from hospital job, but then doing it all over the phone without any kind of reassurance is- is very odd.

Interviewer: Yeah, um, how- have you had any guidance for doing that? How has your, sort of, learning over telemedicine been?

GPTr7: It's definitely been learning on the job, and I think that, um, I'd be interested to- to see your research at the end to know how the GPs feel about it, because a lot of the GPs I spoke to feel really out of their comfort zone, um, and I think that's hard when you're then being supported and guided by people who are also feeling quite- it's out of everyone's knowledge really so, um, I remember back in the beginning of the pandemic, um, there was a day when, for some reason, there were no GPs in the practice, so I kind of got sent home, um, because there was no one to supervise me, but I had a tutorial with my GP supervisor, my clinical supervisor, who kind of went through the very basics of telemedicine, with me? Kind of, checking people's identities and basics things you could- you could ask them, and no one had- it had been a couple of weeks in, and no one had really done that, so I felt like I was really just thrown in at the deep end with it initially.

Interviewer: How have you found the process of um, sort of risk stratifying? Did you say you don't really see any patients in person, or was that just in the first practice...?

GPTr7: Yeah, that was just in my first practice, so at my new practice that I've been with since August, it's- it's a bit easier because you- you can invite people in and see them in your own appointments, and I think we've probably all become a bit more confident over time, in terms of seeing people in practice and patients have become... at the beginning of the pandemic no one wanted to come in, no one wanted to leave their house, no one wanted to call you about anything.

Interviewer: Okay.

GPTr7: Um, it was very odd the patients you were getting- we were getting called about, everyone just sort of had a cough and cold and everyone had sort of Covid-y symptoms. Whereas now it's more like- well, I'm told, it's more like normal GP with kind of a big range of things and you're still having to invite people in and do all that, so I think that's a bit easier, I think risk stratification is (*unintelligible*).

Interviewer: Did you say it's really difficult?

GPTr7: Yeah, I think particularly with kind of children and um, and older people who might again- it might be harder for them to get into the practice, so we are doing some home visits now or- which is much better as well, so you can kind of go out and comfort yourself as much as the patient. (*laughs*)

Interviewer: Have you felt protected for your in-person and home visits, um, in terms of PPE?

GPTr7: Um, I feel like I've been given access to as much PPE as has been given to the medical profession. I think it's- it's hard in general practice because I think that we are really sheltered from what's actually going on in hospitals, I think, as a GP trainee you kind of see what your hospital colleagues are going through and it's- it's so different, we can sit behind our phones and be really quite sheltered so I mean, I think probably the whole procurement of PPE has been a little bit inadequate, but I think that actually as GPs, we- we're not necessarily in the worst position.

Interviewer: Yeah I understand. Yeah, it's quite- quite empathetic of you, yeah, and I guess it depends, how much you're seeing people, but do you... do you work from home when you're doing your phone calls, or is it video calls and phone calls? Sorry.

GPTr7: Phone calls.

Interviewer: Have you used anything like AccuRx or any text messaging devices?

GPTr7: Yes, text- AccuRx has been absolutely the best it's absolutely brilliant, um, brilliant kind of thing to have access to, I think? I can see myself using that forever, even when I'm seeing people face to face. Cause actually I’m quite interested in, um, in- well I'm interested in Global Health, which I'll talk to you about at the end, because I'm interested to hear about your course, but um... I'm interested in kind of making the practice greener and in, kind of, all of that kind of thing as well, I think, avoiding colour printing random stuff for our patients to take home and probably not paying attention to, is- um, is great, and you can send them a text message and when you've got proof that you've done it, so you've got proof of your communication with the patient, and the- the vast majority of patients, particularly under the age of 50, but even people in the 60s and 70s, actually, the way that they access most health information is through their smartphone so... it's great being able to send things directly that you know that they can then look at later.

Interviewer: That's a great point and I don't think anyone's actually pointed that out, that yeah you've got the evidence of consultations. Um... how well informed did you feel that your patients were about the pandemic and about why they had to switch to telemedicine?

GPTr7: Um... very- I suppose in terms of- it's very rare, that you get patients who don't really understand that there's a pandemic, I think- I think people don't feel like it's a consultation is a lot of the problem. And it can be really frustrating when you get to the end of a consultation, and they say, 'ooh, when are you going to be able to- when you get to be able to do consultations again' and you think, well we've just spent 15 minutes and we've had a consultation, and you've got a plan and…

Both laugh.

GPTr7: It’s like, what's your understanding of what's happened here? (*laughs*)

Interviewer: Why do you think that is, that they don't feel they've had a proper consultation?

GPTr7: I think it's particularly with the older generation, I think it's so therapeutic them being seen I think it's the- them getting out the house, having that, kind of familiar, going to that practice that they've been registered for 30 years with, and having someone kind of look at them face-to-face, I feel like they feel like things haven't really happened- particularly if there hasn't been an outcome, like, I've issued you a prescription. Which isn't in a massive- I mean I'd say probably 50% of my consultations I don't end up issuing a prescription or ordering a test or doing anything, it's more advice and discussion, um, which, I think that you can get away with when you've kind of laid a hand on them, but when you haven't done that, it's more tricky.

Interviewer: Okay, thank you that's, um, yeah well-explained, that makes sense to me thank you. How have you felt making decisions with the guidance that you've been given, um, I'm sure you've got patients asking you questions about Covid or you're having to make decisions you wouldn't normally have done over the phone if that makes sense? How have you managed that?

GPTr7: I think it's been- it's been quite shifting the guidance that you're given, which I think was really difficult, particularly at the beginning, um, of, what you should- what you should do to assess people? There was this whole... there was this whole fad of doing the ROTH score? I don't know if you've heard of that.

Interviewer: Yeah, um, a few people have mentioned that, yeah.

GPTr7: We (*unintelligible*) 20 and I tried it a few times and it was always a complete disaster, because patients will not understand what you meant and then... Yeah so, and- and there was also this 'you must, kind of, record this' and then actually there was no evidence behind it, it didn't really do anything. And then there was, kind of the opening of the red centres, which took several months really to start off. So I remember back last March, April, you had people you weren't really sure what to do with them, rather than, say... well you've got to go to A&E, which feels really unsatisfying. Um, I think, as we've gone on, things have become more organized, um, I think that- that knowing... around, kind of, Covid diagnosis, I think it's much easier now, everyone can get test and people don't have to have a specific reason to get a test, everyone can just get them whenever they want, but I think even- even a few months back at the end of last year, there were- there were times when you thought, well, this patient really needs a test to exclude Covid, because we were aware that there were all these cases being diagnosed off the back of a sore throat or feeling a bit tired, um, but there was this kind of 'computer says no' of cough, fever, cough, loss of breath- um, loss of taste then you can't have Covid tests which seems ridiculous?

Interviewer: Yeah, um now that we know so much more about it, um, a few of the GPs have mentioned the same thing, that it just was very exclusive criteria.

GPTr7: Mm, and I think in terms of other things, I think hospitals have been generally quite- quite forgiving in terms of, often you're sending off referrals and saying actually I've not seen the patient, but this is- this is the kind of test result that means I think we need an urgent referral, or, um, kind of saying... that you're- you're not doing things in the normal the normal pattern maybe? Um, but if you- if you know that they're going to need a referral anyway, then I think generally hospitals are being quite understanding where they can with urgent referrals.

Interviewer: OK. Um, you mentioned red zones, could you tell me a bit more about that?

GPTr7: Yeah, so they're kind of the red sites that have set up, kind of at **REDACTED hospital name**, and so actually originally the problem was that they set- they set one up for- for the NEC, um, and it was a site where you could refer patients to be seen and they'd get an appointment and then go around the sort of port-a-cabins in their car, um, and be assessed by a GP there and they could decide whether or not they needed to a hospital. The problem that we had, particularly in my first practice so in **REDACTED practice name** was that actually a lot of our patients didn't drive... A lot of our patients certainly couldn't afford a taxi which would have been 25, 30 pounds and then, can you pay for a taxi just to drive around and wait for- I don't really- I don't think it'd be that appropriate to get a taxi, but if you don't have access to any transport then it's really, really restrictive, um, especially with the not particularly affluent population, so we were really stuck in a position where we couldn't really assess people with Covid? Other than a phone conversation and then go to A&E if you sound like you're going to drop dead (*laughs*).

Um, but there was no other way to do it, and I think we've been a bit more flexible in our- in my next practice, so before they- so they've set up a red site now which is at **REDACTED hospital name**, which is a bit closer, bit more feasible for patients to get to, again it's a slightly different patient population, so they probably be more able to get there, probably more likely to have access to a car, but they have also set up some other things that have been really helpful so they've set up pulse oximetry at home.

Interviewer: Okay, how do patients access that?

GPTr7: We refer people now, um, so anyone over 65 you can refer, or younger than 65 with health conditions, you can refer them to be sent a pulse oximeter and they get called on day, two and three and four, I think, and then, once a week for a couple of weeks, just to check their SATs, and they get given quite clear guidance.

Interviewer: That must be useful for chronic patient management too...

GPTr7: Oh so it's just for Covid really, for people you're worried about with Covid, just for during the acute bit where you're worried their SATs might drop. But I think it's an interesting- an interesting part of that, in terms of you mentioned chronic diseases, I think patients have become a lot more- a lot more willing to think about monitoring their chronic disease themselves?

Interviewer: Oh really?

GPTr7: We've had quite a few COPD patients kind of pick up a pulse oximeter and buy one, and- and have it at home, which is actually great because it means that you can do so much more monitoring remotely.

Interviewer: Yeah, yeah that sounds really, really useful yeah! If patients could access something like that then, um, yeah definitely. I um, I wanted to ask about you said sort of patient presentations changing, but... how does that change sort of your care of patients, and so, for example, chronic patients, um, yeah how's your, sort of, patient care changed for you?

GPTr7: I supposed that- that how it's changed is an interesting one, 'cause I haven't really done the normal...

Interviewer: No I suppose that's fair enough!

GPTr7: I suppose from a chronic patient perspective, I think, initially, they were- everyone was petrified, particularly people with, with kind of COPD or long-term oxygen and things were all just incredibly worried, and they wouldn't have wanted a home visit, even if you'd offered it, because they were all just sat in their homes, not really seeking, I mean, not really seeing any one, and often kind of requesting lots of steroids and antibiotics, and I'm sure other people have told you about the massive inhaler rush at the beginning of Covid?

Interviewer: Yeah one person did mention that.

GPTr7: Everyone- everyone wanted inhalers people who haven't had- had had asthma when they were six and now are 30, saying ‘actually I want my inhaler just in case’.

Interviewer: Right.

GPTr7: Shortages and things of inhalers, because that- there was really kind of a bit of a panic.

Interviewer: Yeah- you're- you're right that a few people have described a panic with a similar thing, and then a lull afterwards, when, um, everything was gone and then it was just people looking afterwards.

GPTr7: Yeah.

Interviewer: Yes, thank you for raising that. Um, this- again this might be difficult to answer because you're saying, because GP is new to you, but from what you've experienced and what you know, how has the role of a GP changed in patient management?

GPTr7: I suppose I think in a lot of ways I think it's, I think, particularly now, things are, things are almost more normal now. So we went through the first phase, when everyone just had coughs and colds and people were staying away with kind of their fungal toenails and things. Then there was the middle phase, when it was summer and Covid kind of went away a bit and everyone- there was- it was almost quite quiet really... and now we're back- seem to back to almost more of the kind of, um, even keel with it. Um, I think patients certainly still seem to feel that we're the first port of call. I think patients are more scared to go to A&E. So, potentially are- are cooling, a cooling kind of the GP more, rather than going to A&E, I'm not sure how that equates to A&E numbers, I suspect it doesn't equate to any numbers at all and they're still absolutely rammed as they always would be, um, but I think that anecdotally, patients seem to be quite nervous if going to A&E. And nervous generally, of going to hospital really. A lot of patients seem quite reassured by the fact that- that well, they don't want to come into the practice and

they say well, 'I'm good and I don't really want to come in and kind of put myself at risk', so I think patient- a lot of patients have adapted quite well to the telemedicine side, and kind of understand that, understand that this is now how care is being delivered, so I, I think in a lot of ways, and we're still doing, we're still doing the gatekeeper role and still being a point of assessment for patients, I think it's just managing our own frustration and sometimes feeling like we can't offer... the service that we'd like to. Um... or sometimes feel like you can't quite manage people how you'd like to.

Interviewer: In what way, do you mean, um, that you'd like to?

GPTr7: I suppose, for example, I had a clinic on Monday, and I actually, sort of, over half of the patients needed a face-to-face appointment, and it felt very inefficient for me to speak to them on the phone and then have to book them in for a face-to-face later in the week... and actually none of them were- none of them were people who had anything kind of desperately wrong with them, nothing was urgent or.. or kind of... life threatening that I needed to see that minute, it was all just kind of actually I don't feel reassured without having listened to your chest, or, or checked your blood pressure, um... I think just trying to... trying to kind of come up with a triaging system whereby maybe we could give more guidance to receptionists on how to get people in, and, I think we're just not quite there yet, we're not quite there with how we actually manage our time?

Interviewer: Okay, um, at the end I would like to ask about future changes to GP learning from this, so I'll definitely ask you about that again, because, um, that's a great idea and raises the theme I've heard a little bit about triage and the uses of triaging, when it's used right, obviously. But yeah, thank you for that. Um, the last, sort of, question on this note is that I wanted to ask how has it changed your relationship with patients or the relationship you'd expect to have if you'd had previous GP experience?

GPTr7: I think this is a really interesting question because I think it's definitely... changed it and I think one of the things that I've noticed- so at the moment I'm- I'm trying to sit the RCA as well, so that's kind of the- the GP exam that's the kind of end-of-training exam, so- which records consultations, so I'm trying to kind of get all that that sorted as well, so I've been kind of jumping through the exam hoops through the pandemic as well, um, I think one of the things I found quite difficult is when you're having a frustrating day, um, or a difficult day, or have a difficult patient, I think sometimes having that lack of connection and having just that kind of phone call, you can find yourself- I don't want to say losing compassion, because it sounds- it sounds really cold, but it's harder to access that compassion, I think, when you don't have someone sat in front of you. And it's much easier to net frustration build if they're not- if they're not telling you the right history, and if they're difficult historian, or they've got connection issues or they're not really- not really paying attention which sometimes happens as well, and you can hear them, kind of, doing the dishes in the background or...

Interviewer: Very frustrating.

GPTr7: And I'm thinking, ‘every time you walk into that corner your signal goes and I can't hear you so just stay still’! And when you think about it kind of logically, I mean there's lots going on in their lives, and then this, it's- it's possible for- it's possible to kind of empathise with that situation, but I think it can get really frustrating on the phone particularly (*unintelligible*) clinic, and it can be difficult, sometimes to- to get beyond that frustration with... with a patient... difficult communication.

Interviewer: Thank you that's a very candid answer and I think reasonable! You're trying to do your job and, um, and you're adjusting as well to telephone calls, so yeah, thank you, I don't think it sounds bad to say.

GPTr7: I think it isn't as much- when you see a person face-to-face it's easier, just to kind, uh, I mean it was interesting to speak to my colleagues about it because they know, they- a lot of the partners at my practice have been there for kind of 20, 30 years, so they know all the patients, and when they call them, they’ve got a face in mind and they've got a person in mind, a real person? Whereas I've just got a voice at the end of the line, and I think for the patient, they've also got me as, almost like I'm in a call centre? And I don't think they necessarily... I don't want to say, not respect- they don't give me the kind of attention necessarily that they would do if they could imagine me as a doctor and have a connection with me? I feel like you lose that a little bit, and you lose- you lose soft stuff, because you lose that kind of connection, you lose that- that mutual respect that I think you really need to have an effective consultation and you really lose clinical things as well, so thinking about- particularly about patients and their weight, we were discussing this in our tutorial the other day, about patients who may be coming with shortness of breath or... gastro-oesophageal reflux or chest pain and you don't- you lose all of the visual clues so are they short of breath when they walk into the waiting room? Are they morbidly obese? Which obviously, you then address straight away and be thinking oh, maybe this is kind of a big factor but, you've got to kind of go out of the notes and say 'oh well when was their weight last recorded, oh that was 10 years ago, is it different now?' And then you have to ask them about it. and you don't have that kind of mental picture in the same way.

Interviewer: And I guess it must be- well saying this as a student, I can imagine it would be a bit easier to forget to ask things that you would- because you're so used to picking up on a complete picture of a person? Um, it's hard to ask about things like, sweat, or it's quite hard to ask about the little things isn't it?

GPTr7: Or do you do ask about it and it's so painfully laborious, sort of them saying 'oh I've got pain in my arm, oh it's around my elbow and it's around the...' and- and actually in real life, they'd just say I've got pain here (*indicates to forearm*) and it goes

here, and I mean you can arrange a video call, but the video calls are... they're a mine-field in themselves with bad connections, fuzziness, and all kinds of craziness. And so, sometimes people try to explain things over the phone, when you know that- that face to face it would take three seconds, it is- is quite frustrating.

Interviewer: Um, that's a very fair point, thank you. You were speaking about your colleagues that have been in the practice, for a long time, has it changed your relationship with your colleagues, or have you felt supported through the pandemic socially?

GPTr7: Um yeah definitely I have been really lucky with the practice I've been, in- I've been really well supported.

Interviewer: Right.

GPTr7: It throws lots of interesting things as a trainee, I think, um, because the way you practice is different- it's- it's interesting being able to sit on the phone and- and Google something at the same time, which you can do. You can Google a guideline if you're not sure! I mean you can do that if you've got a patient in with you, and there any ways to do that, but I think you're much more likely to do it when the patient can't see you. So it kind of makes you a bit lazier in a way, maybe? Also I found particularly at the beginning, I think I've had to work quite hard to get myself out of this habit, particularly at the beginning, when I was feeling less confident, I would just say to the patient 'I don't know I'll go ask my colleague and call you back' and its, for people I wasn't sure about which is- it's absolutely reasonable to do as an ST2 when you're- when you're completely out of your depth and don't know what's going on but, it- it does, in some ways it's easier to- to discuss cases with colleagues, I think, because they don't have patients coming in and out and neither do you, and patients are kind of happy to have a call back. So I think it's, I supposed it's changed with respect to that, but I think it's- it's something that I've come to see tried to do less, and I think in some ways the RCA has helped a bit with that because you're recording so you can't go kind of googling necessarily... hacking away at the keyboard, um...

Interviewer: It's funny how GPs say... GPs who are quite a bit older, saying 'yeah I do get to Google' quite a lot! But they say it almost in a confessional way, like 'now I'm anonymous, I'm googling' and that- that's what they're there, but I understand what you mean, that yeah, there's a bit more pressure on some of you to perform in a different way I guess, yeah.

GPTr7: Yeah, it's a performance. (*Unintelligible*)

Interviewer: Sorry what was that?

GPTr7: Have you used NICE CKS guidelines before?

Interviewer: I was a NICE student Ambassador once...

Both laugh.

Interviewer: It was just something to do, I went to a conference, but that- but I do really like them, I find them really useful as a student and like using them.

GPTr7: I still use them as a GP! (*laughs*)

Interviewer: That's good to know, I can keep doing it then! Um, it's a slightly more contentious question, but how do you feel about the government response to Covid-19 in terms of, not just controlling the pandemic, but public health measures and policies? Because I understand as a GP, patients will sort of use you as a port-of-call when they're having queriers, um, so what's your experience been of that? What's your opinion of that?

GPTr7: Um I think often patients haven't understood isolation guidelines, I think that particularly initially, I was often quite surprised at the number of people who hadn't really absorbed the isolation guidelines. I think it's- I think it's really difficult because there are patients that won't listen, regardless of how much you tell them, they won't listen. So you can still speak to patients, I mean nearly a year in, and they say oh I've had a cough for three weeks and- and they haven't had a Covid test, or haven't even thought about it, but they really are in the minority and, they're the kind of patients that you think are always going to ignore these kind of things they just don't- don't think of the rules um, kind of applying to them. I think the guidelines were- the most contentious guidelines, the most difficult guidelines, I think that we found difficult were... have been, kind of, around shielding... particularly initially, people wanting to be on the shielding list, people not being sure if they should be on the shielding list, um, and patients- patients requesting to be on the shielding list, particularly in the first lockdown because they got them access to things like delivery slots for shopping, um, and um, kind of pharmacy deliveries and things when these things were quite limited. Um, so that was quite difficult, I think, for the GPs in my practice to manage, um, and I certainly had quite a lot of questions as well about that. Um, and then guidelines around kind of specific circumstances particular around, kind, of pregnancy and work, I feel like they- the evidence-the guidance kind of shifted around quite a bit, around that, and I'm not surprised that patients didn't feel like they- didn't really know what was going on, because quite often I didn't feel like I necessarily knew what to tell them.

Interviewer: Yeah.

GPTr7: And I think pregnant people were obviously quite- understandably quite anxious about whether or not to work and what- what was expected of them.

Interviewer: Okay, um, how did you manage scenarios like this, and then- did you have the delayed referral times, did you experience that?

GPTr7: Yeah.

Interviewer: Have you managed these things and explain to patients, and have you had to do anything differently to look after them whilst waiting?

GPTr7: Um... I think- I think patients are used to long waiting times, I think, patients are potentially more forgiving at the moment of long waiting times! Um, because they understand that everyone's really busy, and the vast majority of patients really understand that at the minute. Because some patients, I think you certainly see patients who are struggling with their mental health, I think, waiting for referrals, so for example, I had a patient that had, that was literally sat on the bed in hospital waiting to go down for his cancer operation that was then cancelled, and then he had a second date that was then cancelled the night before, so there are patients who are really, really being put through it really. I mean this chap was amazingly, kind of, resilient to it I thought, that we're still struggling with that kind of uncertainty around it, and I think it can be frustrating, as the GP when you can't really- you and I understand why that's happening and why they can't do it, but I mean, you really kind of feel for your patients, but there's not- there's not a lot we can really do...

Interviewer: Yeah.

GPTr7: It's been quite a lot of delay in like physio referrals and things as well, so... I guess coming back to like AccuRx and things, we're sending patients a lot of exercise resources and, I think patients have become a bit more resourceful in themselves in terms of realising that actually they- they can kind of get engaged with that too.

Interviewer: That's a positive, I guess. It's a slightly more sensitive question to ask, so answer how you will, but has the pandemic had any effect for you personally?

GPTr7: Well, I wasn't supposed to be here this year, so I was on the GP global health fellowship.

Interviewer: Oh yeah!

GPTr7: So that would've have been nice but, yeah, that didn't happen, so I ended up as a bit of a surprise to my ST practice because I wasn't supposed to be there until 2021.

Both laugh.

GPTr7: Which was a bit of a problem for them, because they took on an extra trainee, I wasn’t going to be there. So there was three registrars at the practice which is quite a lot really, um, but yeah, the only thing that didn't get cancelled was my partner's sabbatical year, so he's got a random year off work and- and I've been thrown into ST3. I'm supposed to be sitting a diploma in Tropical Medicine hygiene as well, which also, was just kind of delayed and I'm probably going to put it off now, for another year, because I've had all my GP exams but that's caused a lot of problems, I think, whilst everyone moans that the GP exams actually they've been quite good in keeping them going I think, I'm grateful that I've been able to- continue to use this year as part of my training, rather than have to stay in training for another year or delay my training, because I can't sit the exams that I need to.

Interviewer: Okay, and that's- I think you've had a bit of a tough one, so that's a very nice answer. Well, how are you doing, considering that you've had your year changed?

GPTr7: Yeah it's alright, I mean I'd rather not be- rather not be doing the RCA and thing, I think that's quite tough to do at the moment. It really is- it's a tricky exam as it is, it is, I think, but, I think when you don't have all that kind of support of seeing friends and family, and being in another lockdown, it's a bit tedious. But I think all the plans that I've had to cancel, were things that had actually just become untenable in a pandemic, I wouldn't particularly like to be in South Africa at the moment, really, because I think that they're probably in much worse condition than we are and, unfortunately, are probably likely to be for long time, given the government's attitude towards aid supporting other countries' pandemic responses so, I think that yeah, it's gonna- it's... globally, I think... it's going to be a problem for a long time isn't it.

Interviewer: Well I hope that it happens for you, at some point, uh, and you've got something nice to look forward to! And at least these exams are done but they yeah they don't- they don't sound pleasant. (*laughs*) Um, do you think there are any

changes which you like to see carried on into future practice, so I know you were talking earlier about some ideas you had. What would you advise if given, given the voice to advise. What would you say you've learned from this?

GPTr7: Given the power! I think, teleconsulting can- does have its place and can be really useful. A lot of the time it's- it's- it's really efficient, you can get things done really quickly you don't eat to end up converting it to a face-to-face call, um, you can- the patient can book it in at their convenience which is again a double edged sword, because some patients just, kind of, see you as, kind of, a convenience store of general practice so- but I think that teleconsulting and being confident with that is great, and I think I will always be more confident now with teleconsulting because I've spent so much of my training doing it, um, so for out of hours training for- for GP training, you have to do a certain amount of out-of-hours and that's the Badger out-of-hours, which is all tele- it's kind of like a telephone conference centre, um and you go and do your out-of-hours, and I think back in the past trainees used to find it quite difficult because they'd never done teleconsulting before, so it was all very new and different, whereas for me it's just the day job, that's what I do at the time, so I do find it- I find that I can manage most things over the phone and I'm quite aware, now, when I need to bring people in and- and do that kind of thing, so I think, uh, keeping some telephone consulting things. But I don't know how you build a triage system that works to mean that you're not wasting your time, double- double, kind of, consulting people? I think telephone consulting is really difficult with kids, I think most of the time you want to see kids, so potentially having kind of automatic bookings for under-5s. Um... I wonder what the effect would be of giving patients the choice? Um, I wonder if all patients would say they want a face-to-face, or actually whether or not people, particularly younger people who are at work, would say actually I quite like a telephone consultation. Particularly for a mental health follow up, that- that really is just kind of a, 'how are you doing, and should we continue your medication', it could be really appropriate to have a telephone consultation. And I think the speed with which AccuRX has come to be used is- is great, and I think that it's brilliant from a green aspect in terms of communication with patients? Um, and in terms of our ability to- to request things like photographs, it's really helpful, and do video consults if you need to say, I think that would definitely be something that I hope stays.

Interviewer: Great Thank you that's a great answer. Okay great. Thank you that's a great answer, um lots to think about thank you. And um, yeah, a few things I've heard from other trainees, so it's really interesting to hear your insight definitely. Um, my final question would be about how you think the pandemic has influenced your training, I know that, um, well you've mentioned a few things, but if you could tell me how has it affected your training?

GPTr7: Hmm yeah, so I think I've kind of mentioned that it's- it's changed my exams, um, it's so, it's changed- a completely different exam structure, it's meant that- that all of my training has been telephone, pretty much all my GP training has been i the pandemic. I'm pretty sure I'm going to qualify in August having never having done a full face-to-face GP clinic? (*laughs*).

Which I find kind of horrifying really, but actually speaking to my colleagues, they say that they don't really see it going back to being that... particularly much in the future. I think it's looking at picking up different skills so, I still see a huge range of things, I'm still managing a lot- of a lot of presentations and seeing a wide range of patients that just in different ways, I think the world of GP has changed, and, from what people say to me they think it's going to change back particularly quickly. So I suppose my thought to myself, has been would I rather be in my position, where my last year and a half of training is just telephone consulting, or would I rather have just qualified then only had six months of being a GP and then suddenly going to telephone consulting being a fully qualified GP? I think that almost would be worse really. I feel in some ways, being a trainee you can be quite protected, and you can- you can get quite a lot of support that- it's just- it's just very, very different, and I think that we will always be kind of the- the Covid- Covid cohort I suppose. That will always be my- my experience of GP training, but I think the thing about GP is that that you don't qualify and then become an expert. It's bit different to hospital medicine in that respect I suppose. They've got this whole concept of 'first five GPs' now where you get- you can get extra training and support in your first five years, and I would hope that- that they adapt that to some extent to help with the Covid- the Covid cohort who've we've had a really different training experience.

Interviewer: Um, has it influenced your view of general practice, are you still looking forward to going into it, is that your plan?

GPTr7: Yeah, it's definitely still my plan. Um, I think my current feelings towards general practice are more influenced by the RCA, I think, which I find really doesn't suit my temperament at all, I think it's a, it's an exam that really- that really kind of colours your day to day practice because you spend your whole day to searching for kind of perfect recordings that don't exist, um, but I think- I think it's at times, I think the frustration that- the main frustrations that I've had is when I've found it hard to connect with patients, and I think it's always a joy to see patients in the practice, and I think the hope that I've got is that, as we go forward, um... We will be able to do that a bit more and we'll be able to continue seeing patients because it's just more interesting. You lose all of the fun of it. Talking to a parent with a toddler on the phone is fine, and you can get all the information you need, but you don't get to see a cute toddler and play with them and, kind of, interact with them, and have all of that kind of banter with older people, and you don't get that and you do really miss out on it, I think it's- it's where the- where the joy comes in, in medicine I think.

Interviewer: Thank you. Is there anything that we haven't spoken about today, anything that has been important to you and your experience?

GPTr7: Um... I don't so, I supposed, I supposed with the training aspect of I suppose- I'm stuck on a diatribe- I don't know if the other trainees you've spoken to you have mentioned kind of their- their- their teaching experience in terms of...

Interviewer: Very variable, how's your been?

GPTr7: So it's been put on to kind of... teams Microsoft teams and initially there was a long time where it kind of wasn't and it was all a bit vague, um, and I think it is- I think it's been really, um... difficult I think the quality- the quality of its been (unintelligible) anyway I think, um, but I think it's been more difficult on teams, where we've had people who've obviously not been very familiar with how online teaching works and... um... I feel like in some ways it's kind of skated around the difficulties of things like telephone consulting. Our teaching, for some reason is all trainees together, so I feel really sorry for the trainees who are in hospital for the first 18 months, um, of their training, because their teaching just isn't really relevant to them, and I think they're having such a difficult time in hospitals, at the moment, that they- that they, kind of, can't really have that mental space to think about 'oh, actually, how would I do this in general practice'? And um, when you're in dental practice, kind of the, what you're trying to do is get through and... and pass your exams and... work out how to do a telephone consultation where the patient doesn't just ramble on for 15 minutes and you don't get say anything, and... I didn't know. I think it's been difficult. I think, teaching online has not been the best and you've missed out- the biggest problem is you've missed out on the networking with other trainees.

Interviewer: Yeah, actually no one's mentioned that before and that's a great point.

GPTr7: Because that was the best thing about the teaching, was the tea-break. Unfortunately, when you get to chat to other trainees and- and find out- because GP's quite isolated in a lot of ways, um...

Interviewer: Sure, yeah I was wondering about that because, yeah 'cause GP could be a little bit lonely I'd imagine. That's why I asked about your colleague support really, because I wondered if there was still people to sort-of socialize with, who are going through the same thing, but I guess... I really hope that's recovered soon and there's some, sort of, yeah.

GPTr7: Hmm. I think going through the exam has always been helpful because a little group of us who are doing the exam at the same time have kind of got together to- to discuss that, but I think when I was an ST2 it wasn't really happening so much. If you're just your one trainee GP at your practice and there's no other trainees there, it can be a bit difficult to... to... And if you don't really have many friends in GP practice, it can be difficult to... to have that kind of acquaintance connection? Because you're not like the kind of friends, where you'd text each other about how you're feeling, but you'd have a chat, a tea break, and kind of have a bit of a moan, or a bit of- a bit of an outlet, and I think- I think yeah, I think probably the- the lack of teaching, I probably missed- I probably missed that especially initially the most.

Interviewer: Yeah. Thank you. That’s a really nice candid answer and something that I'll ask future trainees a little bit more about as well, so thank you very much. Um, OK, I feel like I've taken so much of your time! I'm going to stop recording.

Recording ends.
