# Supplementary material for: Primary health care during the COVID-19 pandemic: A qualitative exploration of the challenges and changes in practice experienced by GPs and GP trainees
Source: PLoS One. 2023 Feb 9;18(2):e0280733. doi: 10.1371/journal.pone.0280733 (PMC9910752; doi:10.1371/journal.pone.0280733)
Supplement: Attachment — Submitted filename: Response to Reviewers.docx [file pone.0280733.s006.docx]

**Response To Reviewers**

Reviewer 1: Sanjida Arora

Reviewer 2: Anonymous

|  | Element of paper | Reviewers’ feedback | Authors’ response |
| --- | --- | --- | --- |
| Journal Requirements | *PLOS ONE requirements* | Please ensure that your manuscript meets PLOS ONE’s style requirements, including those for file naming | To do in submission |
|  | *Financial disclosure* | Please state what role the funders took in the study.  If the funders had no role, please state: ""The funders had no role in study design, data collection and analysis, decision to publish, or preparation of the manuscript.""  If this statement is not correct you must amend it as needed.  Please include this amended Role of Funder statement in your cover letter; we will change the online submission form on your behalf. | Thank you, we have ensured this is in our cover letter to be updated for the online submission form. |
|  | *Funding statement* | We note that you have provided funding information that is not currently declared in your Funding Statement. However, funding information should not appear in the Acknowledgments section or other areas of your manuscript. We will only publish funding information present in the Funding Statement section of the online submission form.  Please remove any funding-related text from the manuscript and let us know how you would like to update your Funding Statement. | Thank you for alerting us to this error. We have removed the funding-related text from the manuscript and would like to keep the Funding Statement the same, as described in the cover letter. |
|  | *Data availability statement* | You have not specified where the minimal data set underlying the results described in your manuscript can be found. PLOS defines a study's minimal data set as the underlying data used to reach the conclusions drawn in the manuscript and any additional data required to replicate the reported study findings in their entirety. All PLOS journals require that the minimal data set be made fully available  In your revised cover letter, please address the following prompts:  a) If there are ethical or legal restrictions on sharing a de-identified data set, please explain them in detail (e.g., data contain potentially sensitive information, data are owned by a third-party organization, etc.) and who has imposed them (e.g., an ethics committee). Please also provide contact information for a data access committee, ethics committee, or other institutional body to which data requests may be sent.  b) If there are no restrictions, please upload the minimal anonymized data set necessary to replicate your study findings as either Supporting Information files or to a stable, public repository and provide us with the relevant URLs, DOIs, or accession numbers.  We will update your Data Availability statement on your behalf to reflect the information you provide. | We have attached our data as supplementary files (21 transcripts), however we are currently exploring utilising a data repository, and will soon let you know if this is successful and we wish to transfer the data over. Hopefully this is OK - we should know this week. We have described this in our cover letter. |
|  |  |  |  |
|  | *Direct billing option* | Please note that in order to use the direct billing option the corresponding author must be affiliated with the chosen institute. Please either amend your manuscript to change the affiliation or corresponding author, or email us with a request to remove this option. | The corresponding author, Minka Grut, is affiliated with the chosen institute, the University of Birmingham. Please let us know if we have not selected this as the institute! |
|  | *Ethics statement* | Your ethics statement should only appear in the Methods section of your manuscript. Please ensure that your ethics statement is included in your manuscript. | We have now ensured that it is only in the Methods section, thank you. |
| Reviewers Responses to Questions | *Is the manuscript technically sound, and do the data support the conclusions?* | Partly | N/A |
|  |  | Partly | N/A |
|  | *Have the authors made all data underlying the findings in their manuscript fully available?* | No | We have now made the data available through |
| Review Comments to the Author | *General explanatory* | The author needs to define "general practice". What does general practice entail in context of primary healthcare? | We have now defined general practice in the introduction and hope that this better contextualises the experiences of our participants. |
|  |  | Line 67- please explain what is shielding | We have now clarified what is meant by shielding, thank you for noticing this. |
|  | *Methodology* | Sampling methodology and size:   - An average of 36 minutes for interviews seems inadequate to capture broad areas mentioned in interview guidelines and the nuanced aspects given that there was no prior relationship between researcher and participants. - The authors could have increased the sample size, though for qualitative study, even lesser would be good, provided there is richness in the data. | - We agree that an increased sample size might have been beneficial to the study. We were satisfied that the data set we produced was very rich due to both the forthcoming nature of the participants, and the directing help of our topic guide. - Due to the time constraints at the time, when this was originally a university dissertation, it was not possible to hold interviews of a much longer time period. However, it is worth mentioning that the 36 minute average was because some interviews were much shorter (for example, a participant would have had a unique experience (e.g. Taking time out during some of the pandemic) that were hard to control for but meant less questions were applicable), and some were much longer. |
|  |  |  |  |
|  |  |  |  |
|  |  |  |  |
|  |  | What was the rationale of including GP trainees? | - Thank you for questioning this. We have defined and clarified their inclusion in the manuscript, and hope that this better explains the chosen cohort (and additionally the meaning of ‘GP trainee’ for readers outside of the UK). - We included GPs in this cohort because their responsibilities and thus pandemic experience are very similar to that of fully trained GPs. - Furthermore, we wished to determine if practising in exceptional circumstances such as COVID-19 had affected their training or their views towards becoming a GP. This relates to job retention and alerts the reader to any support that the new cohort of GPs might need, to compensate for any missed learning experiences. |
|  |  | What was the content of the social advertisement developed for recruitment? Were these also reviewed by ethics committee? | We have attached the social media advertisement as supplementary material for your convenience. It was not reviewed by the ethics committee, however its content was described in detail, and approved by the committee. We have now clarified this in the ‘Methods’ section. |
|  |  | - Though authors have listed as a limitation, the channel of recruitment and the incentives provided, could have resulted in somewhat a homogenous and self-selected group. - Were participants informed a-priori that they would receive a 30GBP voucher for participation? Even if not, was it possible that others came to know of it (snowballing) which influenced self-selection? | Interviews were informed of the £30 voucher through the social media advertisements; the decision for this was three-fold:   - Firstly, we too anticipated a strong chance of others coming to know about the voucher before their interviews. We felt it was better to ensure that the participants had had access to the same level of information, and thus decided to advertise the voucher beforehand. - Additionally, we had a limited study period and limited resources with which to conduct the study (due to its being conducted as a component of a university dissertation), an incentive with the use of social media allowed for us to recruit an adequate number of participants - Most importantly, we felt that it was most important, even with the limitations it created, that participants were compensated, both for their time and for the emotional duress of recounting what was an incredibly stressful time for GPs. At the time of the study, we were still fluctuating in and out of a lockdown period, so it was very fresh for participants. - Thankfully, we do not believe it greatly influenced who participated, as the majority were very motivated by the opportunity to get to speak about their time in the pandemic and have their experiences shared. |
|  |  | - Line 128- Participants were selected based on professional role, urban/rural- what were these roles? Was any comparison done based on roles or urban-rural divisions? - I would be interested to understand what kind of saturation in data did the researchers reach that they felt this was a good number to stop data collection. For example, even though there were both urban and rural based practitioners (adopted as a sampling strategy to provide diversity), there is no comparison or comment made on these lines in findings/discussion. | - We have now included the professional roles in Table 2 and defined their meaning in the table footnotes. We had 1 GP partner, 1 GP who was a partner then a locum in the pandemic period, 1 locum GP, 1 GP who was locum then salaried, and finally 6 salaried GPs. We additionally had the 10 GP trainees. - In terms of differences between professional roles, the key finding was the unique experiences of locums, who described a lack of job security in comparison to salaried/partner GPs, and more loneliness than those within a consistent practice setting. However, the locum cohort was very small, and with a vast amount of findings and space constraints, we felt that this was less significant than other findings that were explored in the results/discussion. - In terms of practice settings, we did not observe any prominent differences, although again the sample size was not large and therefore might not have illuminated more subtle differences. - Past this, unfortunately within the time constraints it was not possible to perform a full comparison of how the GP roles, and their practice settings, influenced the data. Our main intention was to achieve variation in order to ensure that the insights we received were not limited to one group, and that they are hopefully representative of the wider UK population of GPs. - Please see below regarding data saturation. |
|  |  | Was coding of transcripts done simultaneously as more interviews were being conducted? Or was done after all interviews were over? What kind of theoretical saturation was achieved? | - Yes, they were done simultaneously, which we have described in ‘Data Collection’. Codes were developed into themes and analysed once the interviews were completed. - Data saturation was reached when no new codes were appearing, ie. the point of ‘information redundancy’. This point was determined through discussion within the coding team of three members, and we conducted two further interviews to confirm data saturation. We have amended our description in ‘Participants and Recruitment’ to clarify this process. - Whilst each interview continued to introduce a unique insight, our intention was to focus upon the most frequently appearing codes for analysis. We acknowledge that this is a nuanced term, particularly in the context of qualitative data, and have clarified within the ‘Participants and Recruitment’ section. |
|  | *Conceptualisation* | Results / Themes   - The weakness of the paper is not having a set of well-defined and theoretically backed research questions beyond stating the obvious ‘what the experiences of the GP & GP trainees on are…”. - Much of the findings are mere descriptions and even the presentation is based on broad descriptive categories than ‘themes’. - For instance the fifth theme is called “suggestions” which by itself cannot be considered as a theme. Similarly the fourth theme on changes to teaching and support could actually be combined with theme 1 as they both speak to need for effective support and guidance. - Each theme in the paper received more of a ‘touch and go’ treatment and remain isolated from each other. May be the paper should have focused just on the telemedicine aspect or on the personal/professional dilemmas. | - At the time of conducting this study, it was the first of its kind, and so was entirely novel to the evidence base. For this reason, we could not predict which elements of the pandemic would be most salient - indeed until we began interviews, we had not even realised the vast uptake of telemedicine. We therefore utilised an inductive approach rather than a deductive approach. - A broad investigative scope allowed the participants to guide us towards what had had the most impact on them. Our pilot interview was useful in refining our topic guide; however, we could not eliminate questions further than this for the concern that they might be more important in the following interviews.   Themes:   - Thank you for your feedback on this, it has prompted reflection upon the intention of our study and how the GPs’ voices have been conveyed. In response, we have changed some of our theme names to hopefully incorporate the sentiments of our participants, rather than give descriptive names for broad categories. - We acknowledge that our themes might feel slightly disjointed and agree that perhaps this is a result of having covered such a large topic area gleaning a lot of important results. However, we believe that a strength of this study lies in its capturing of the overall effect Covid had on GP and GP trainees. We hope that this is where it will be most beneficial to the evidence base, by allowing GPs themselves to highlight the most salient issues that arose as a result of the pandemic. - Thank you for drawing such interesting parallels between Theme 1 and 4 regarding support and guidance, we hadn’t considered this! We have decided it is important for theme 4 to be kept separate from theme 1, because despite the similarities in terms of support and guidance, the important distinction for us is the homage to how affected GP training was – including how being a trainee altered their experience of the pandemic in comparison to trained GPs, the support they needed that was specific to their progression as doctors, and the particular emotional duress that arose as a result. |
|  |  | The paper could have elaborated more on how the NHS functions and the role of GPs and general issues faced by the health workforce of that cadre. This would also include on how other building blocks of the health system (financing, leadership & governance, information systems, access to medicines) interact with health workforce to affect health service delivery. In the current version, the paper doesn’t connect the findings to the health systems. |  |
|  | *Findings/discussion* | The only in-depth exploration that the authors undertake is the use of tele medicine. However even there, no new dimension or an argument is not emerging from the study. | - Thank you for alerting us to this, hopefully we can explain leading on from our answer above. When we began conducting this study at the beginning of 2021, there was a limited evidence-base to draw from, so as described above we had a rather broad topic guide and results. - At the time of the study, we had fairly little data on the shift towards telemedicine, as an example, within the practice, and so compiling our participants’ views and wants with regards to its future in general practice felt like a salient point to discuss. - Furthermore, the GP opinions were and are in consensus of existing evidence, so we believe the relevance is that it agrees and thus supports the evidence base. |
|  |  | - The authors claim to have used recursive thematic analytical style. While it is relevant for a research topic of this nature, the thick description that is required for qualitative research is missing in the findings. Central to any qualitative research is the ‘emic’ perspective, but in this paper, there is nothing much discussed about the participants’ contexts except for what they themselves have ‘reported’ as an experience/opinion about the pandemic. - For an overwhelming catastrophe of this nature, the emotions of the providers seem to be missing. Probably the researchers were not trained enough to elicit such responses, or these arise due to lack of personalization in telephonic nature of interviews compared to face to face. - The interview guidelines also reflect that each topic was not sufficiently exhausted and moved from one broad area to another. I could get some glimpses of it, such as when the providers mentioned about guilt of delayed referrals, etc. - I am also curious to know if the respondents mentioned about their own families as it would have been an important aspect of the GP’s making meaning of the pandemic, themselves, their lives and their patients. Though the guidelines mention a set of questions to explore this, nothing has come out in findings. - In addition, I felt the researcher-author’s voice(s) were also missing in the discussion and the paper is almost reduced to a ‘report’ than a research finding. | - We are sorry that the emotions of the providers have not been allowed to show through in our findings, particularly because they were kind enough to show these in the interviews. Quotations were limited in the interest of covering the large amount of topic areas, however clearly the balance has become skewed towards the takeaways from the interviews. We have re-focussed our findings section so that whilst the productive outcomes from the interviews can be discussed, the central emotion and poignant reflections of the participants has not been lost. We have done this particularly through using more quotations. - We agree that the technology barrier was a limitation, and additionally this was my (first author) first time conducting a study and qualitative interviews. I’m grateful for your feedback, and will subsequently work on my interview technique and in particular eliciting the emotions of interviewees. - In terms of the participants speaking about the personal effect of Covid on themselves and their families, indeed we did have a few participants kindly share the difficulties that they faced. Our decision not to include them as a theme was not because they weren’t important but rather due to the lack of data, as many participants deflected from this topic or misunderstood it. Again, this is likely due to interviewing technique and is something I will definitely practice and study further. - We have re-inserted a quote from an earlier revision (please see GPTr6 in ‘Reduced Support‘). Whilst there might not be sufficient data for a full discussion or theme, we agree it is important to allow these voices to come through to truly illuminate the stressful time that GPs managed. |
|  |  | Quotations from providers are few and do not capture diversity of experiences. | - As above, we have taken this on board and used more quotations. |
|  |  | Lack of support from higher ups and delayed referrals are not sufficiently analysed. Only authors would know if there is more analysis that can be brought in or not. | - We have refocussed and developed our analysis to encompass support from higher ups *and* from SC within the paragraph ‘Support’. We believe these are similar concepts and naturally lead on from each other to complement each other well within the analysis. |
|  |  |  |  |
|  |  | There is very little connection between what was set ought, what was found and what was concluded. For instance, Telemedicine is being suggested for fulfilling “patient preferences and needs” while the entire paper has been on provider experiences. | - Having reflected upon this feedback, and considering our intention in the writing, we must agree that the focus of the participant experience has been lost in places to the patient experience. In many ways, this is central to the provider experience, as a patient’s takeaway from a consultation and wants//needs of their GP can so influence the GPs’ experience. - However, we wish to clarify that our focus was the GPs, and thus we have adjusted the writing, particularly in the discussion, to draw any mentions of the patient experience (which is, of course, only from what we know from our GP interviews and thus is not a strong enough descriptor of patient experiences anyway) back to what this meant for the GPs. Where it hasn’t seemed very relevant to the goal of the study, we have omitted. |
|  |  | The need for training of GPs on effective health communication or the need for leadership skills which comes out as a finding in the previous section strangely does not find place in conclusion! More details need to be given for what exactly authors mean by a stronger team based support. | - We have adapted our conclusion to allow more of a personal voice to come through and hopefully to show what the main takeaways of this study were. - We recognise the lack of clarity when describing ‘team-based support’ and have omitted this phrase, in favour of the description within the leading sentence of the conclusion and the discussion topic ‘Support’. |
|  |  | The scope of future has been explored only for vaccine, what about other aspects of general practice? | - Thank you for this feedback on such a critical part of the paper. The key implications for future practice that we have explored include the development of a teleconsulting hybrid, facilitating specialist advice for GPs to keep patients in the community, and the advantages of strengthening support within GP practices in the future. To incorporate your feedback, we have adjusted the end of the introduction to direct the readers towards the future implications that we explore within the discussion. |
|  |  | Would be useful to lay down the scope of telemedicine in context of different healthcare needs. for example- medical abortion and telemedicine | - Thank you for this feedback, we agree it certainly deserves a full exploration and is relevant to the outcomes of this qualitative study. Due to the space constraints, and receiving your helpful feedback, we have deemed that the novel takeaways from this study require a more focused reflection upon in the discussion and the conclusion, and so we have not been able to contextualise telemedicine far out of the obvious primary care uses it has had, however we hope that it’s prominence in the study does prompt readers to explore the scope of telemedicine outside of the writing. |
